# Rh^III^‐Catalyzed C−H Activation of Aryl Hydroxamates for the Synthesis of Isoindolinones

**DOI:** 10.1002/chem.202002384

**Published:** 2020-07-21

**Authors:** Saad Shaaban, Caitlin Davies, Christian Merten, Jana Flegel, Felix Otte, Carsten Strohmann, Herbert Waldmann

**Affiliations:** ^1^ Max-Planck-Institute of Molecular Physiology Department of Chemical Biology Otto-Hahn-Strasse 11 44227 Dortmund Germany; ^2^ Technical University Dortmund Faculty of Chemical Biology Otto-Hahn-Strasse 4a 44227 Dortmund Germany; ^3^ Ruhr University Bochum Organic Chemistry II Universitätsstrasse 150 44801 Bochum Germany; ^4^ Technical University Dortmund Department of Inorganic Chemistry Otto-Hahn-Strasse 6 44227 Dortmund Germany

**Keywords:** C−H functionalization, isoindolinones, isoindolobenzazepines, rhodium, styrenes

## Abstract

Rh^III^‐catalyzed C−H functionalization reaction yielding isoindolinones from aryl hydroxamates and *ortho*‐substituted styrenes is reported. The reaction proceeds smoothly under mild conditions at room temperature, and tolerates a range of functional groups. Experimental and computational investigations support that the high regioselectivity observed for these substrates results from the presence of an *ortho*‐substituent embedded in the styrene. The resulting isoindolinones are valuable building blocks for the synthesis of bioactive compounds. They provide easy access to the natural‐product‐like compounds, isoindolobenzazepines, in a one‐pot two‐step reaction. Selected isoindolinones inhibited Hedgehog (Hh)‐dependent differentiation of multipotent murine mesenchymal progenitor stem cells into osteoblasts.

## Graphical abstract

**Isoindolinones**: The first Rh^III^‐catalyzed C−H functionalization toward the synthesis of isoindolinones with styrenes is presented. The use of styrenes with a coordinating side arm together with OBoc‐aryl hydroxamates completely switched the regioselectivity. Mechanistic investigations supported the β‐hydride elimination pathway. This method gave an access to NPs scaffold (isoindolobenzazepines). Some of the synthesized derivatives inhibited Hedgehog (Hh)‐dependent differentiation of multipotent murine mesenchymal progenitor stem cells into osteoblasts.

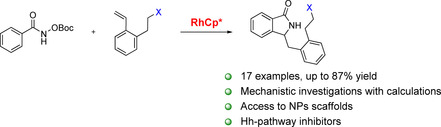

Transition‐metal‐catalyzed C−H bond transformations exploiting different metals have emerged as powerful synthetic methods.^1^ Among them, cyclopentadienyl rhodium complexes (RhCp*L_n_) have been utilized for the C−H functionalization of aromatic compounds employing different directing groups.^2^ Since the first report by Miura *et al*.,^3^ RhCp* complexes have proven to be efficient catalysts for the functionalization of C−H bonds adjacent to benzamides. A variety of annulation partners have been employed in C−C and C−X bond formation.^4^ In particular, the respective coupling of *N‐O*‐aryl hydroxamates and alkenes (predominantly styrenes) has successfully been used to synthesize dihydroisoquinolones (Scheme 1 a),^5^ including enantioselective transformations.^6^ However, the synthesis of isoindolinones, that is, the five‐membered ring analogues of the dihydroisoquinolones, by RhCp*‐mediated C−H functionalization has been explored less frequently. Thus, Rovis *et al*. used diazo compounds as annulation partners (Scheme 1 b‐left),^7^ and isoindolinones could also be formed with electron‐deficient alkenes as coupling partners.^8^ The latter reaction favors the formation of the Heck‐type intermediate *via* β‐hydride elimination followed by a Michael‐type addition. However, these transformations require electron deficient alkenes and elevated temperatures and as a result, their applicability is limited (Scheme 1 b‐right). Direct formation of five‐membered isoindolinones from aryl hydroxamates by coupling with styrenes, that is, the regioisomeric transformation for the formation of dihydroisoquinolones (see Scheme 1 c), has not been described. We envisioned that such a transformation might be realized by attachment of a coordinating substituent to the *ortho* position of the styrene. Coordination of this group to the Rh catalyst in the expected seven‐membered ring intermediate would favor the β‐hydride elimination over the reductive pathway. Subsequent cyclization would then enable the formation of the isoindolinone (Scheme 1 c).^9^

**Scheme 1:**
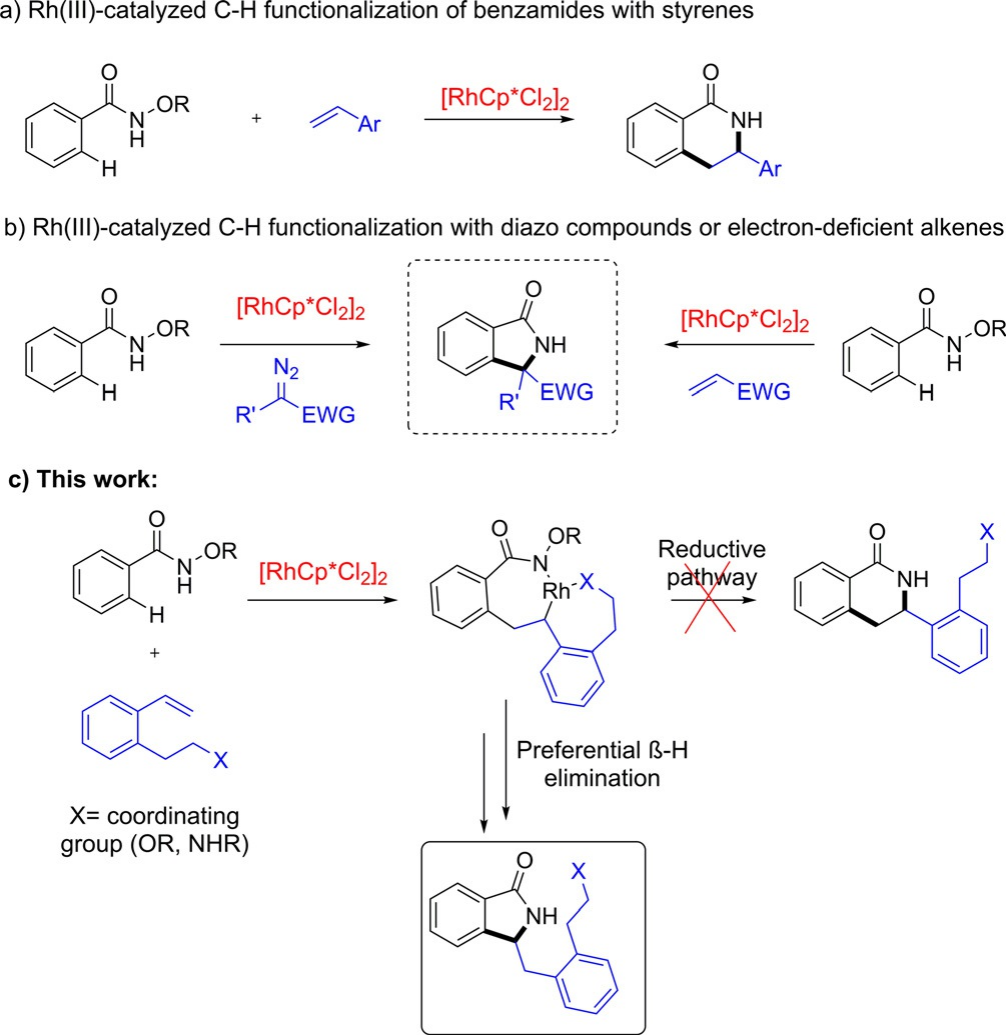
Rh^III^‐catalyzed C−H functionalization of aryl hydroxamates: a) with styrenes; b) with diazo compounds or alkenes with an EWG; and c) our work with styrenes bearing a coordinating side‐arm in the *ortho* position.

In initial experiments, the reaction of Piv‐protected benzamide **1 a** with styrene **2 a** bearing an alkyl‐mesylate (R=CH_2_CH_2_OMs) tether was explored (Scheme 2). Gratifyingly, the resulting reaction yielded isoindolinone **C** as the major product, albeit with formation of the six‐membered ring product **A**. In all previous reports on related reactions, *ortho*‐substituted styrenes have not been used,^10^ such that, subsequently we further investigated the influence of the *ortho*‐substituent of the styrene on the transformation. To this end, the reaction was performed using the OPiv‐hydroxamate **1 a** with styrenes bearing Br and Me groups in the *ortho* position. However, none of these styrenes afforded the five‐membered ring isoindolinone. Instead, a mixture of the six‐membered ring regioisomers **A** and **B** were obtained. In accordance with previous reports,5a, 5c styrene led to the formation of **A** as the sole product. You *et al*. reported recently, the formation of the five‐membered ring with any styrenes using OBoc‐hydroxamates,^9^ and we wondered whether this would also be the case here. Therefore, the same reactions (Scheme 2) were explored with OBoc‐phenyl‐hydroxamate **1 b**. In the presence of this substituent the styrenes afforded exclusively product **C** in high yield. Only styrene forms minor amounts of the six‐membered ring regioisomer **A**. These results show that the OBoc‐phenyl‐hydroxamate **1 b** plays a crucial role in the formation of the five‐membered ring adduct. In addition, the finding that only the mesylate‐protected styrene with the OPiv‐phenyl‐hydroxamate **1 a** yielded the desired isoindolinone **C**, supports the hypothesis that a coordinating group in the *ortho*‐position may favor an alternative reaction pathway.

**Scheme 2:**
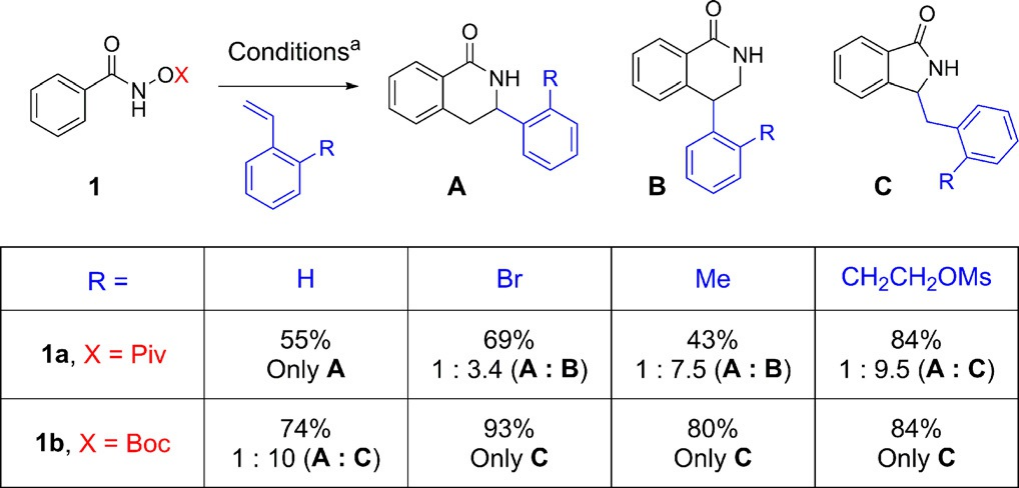
Effect of *ortho*‐substituent of the styrene moiety and the OX‐protecting group. Reactions were performed using [RhCp*Cl_2_]_2_ (1.0 mol %), CsOPiv (25 mol %) in MeOH [0.1 m] at RT for 14 h. Yields were determined by ^1^H NMR spectroscopy by using 1,3,5‐trimethoxybenzne as internal standard. Ratios were determined from the ^1^H NMR spectrum of crude mixture.

Optimization of the reaction conditions were carried out. The use of 1.0 mol % of [RhCp*Cl_2_]_2_ with a catalytic amount of potassium acetate KOAc in methanol at room temperature resulted in the formation of desired product **C** in 74 % isolated yield (see the Supporting Information for details). With the optimized conditions in hand, we then explored the scope of this transformation (Scheme 3). A variety of alcohol and amine protecting groups (OMs, NHTs, OAc and OTIPs) on the *ortho*‐substituted styrene were tested and were well tolerated, providing the five‐membered ring products in good yields ranging from 58 % to 87 % (**3 a**, **c**–**e**). Interestingly, the reaction also proceeded well when the free alcohol (**3 b**) was used. Different functional groups on the aryl hydroxamates (Cl, F, Me, OMe and NO_2_) were tolerated to yield the desired adducts in good yields (**3 f**–**k**, **3 n**,**o**). Whereas the *meta*‐methyl derivative yielded only a single regioisomer in high yield (**3 l**), its chloro‐analogue gave a 1:1.3 mixture of regioisomers (**3 m** and **3 m′**).^11^ The nitro‐substituted benzamide **3 p** afforded the product in lower yield. For this product the isoindolinone structure was confirmed by means of crystal structure analysis (see the Supporting Information).

**Scheme 3:**
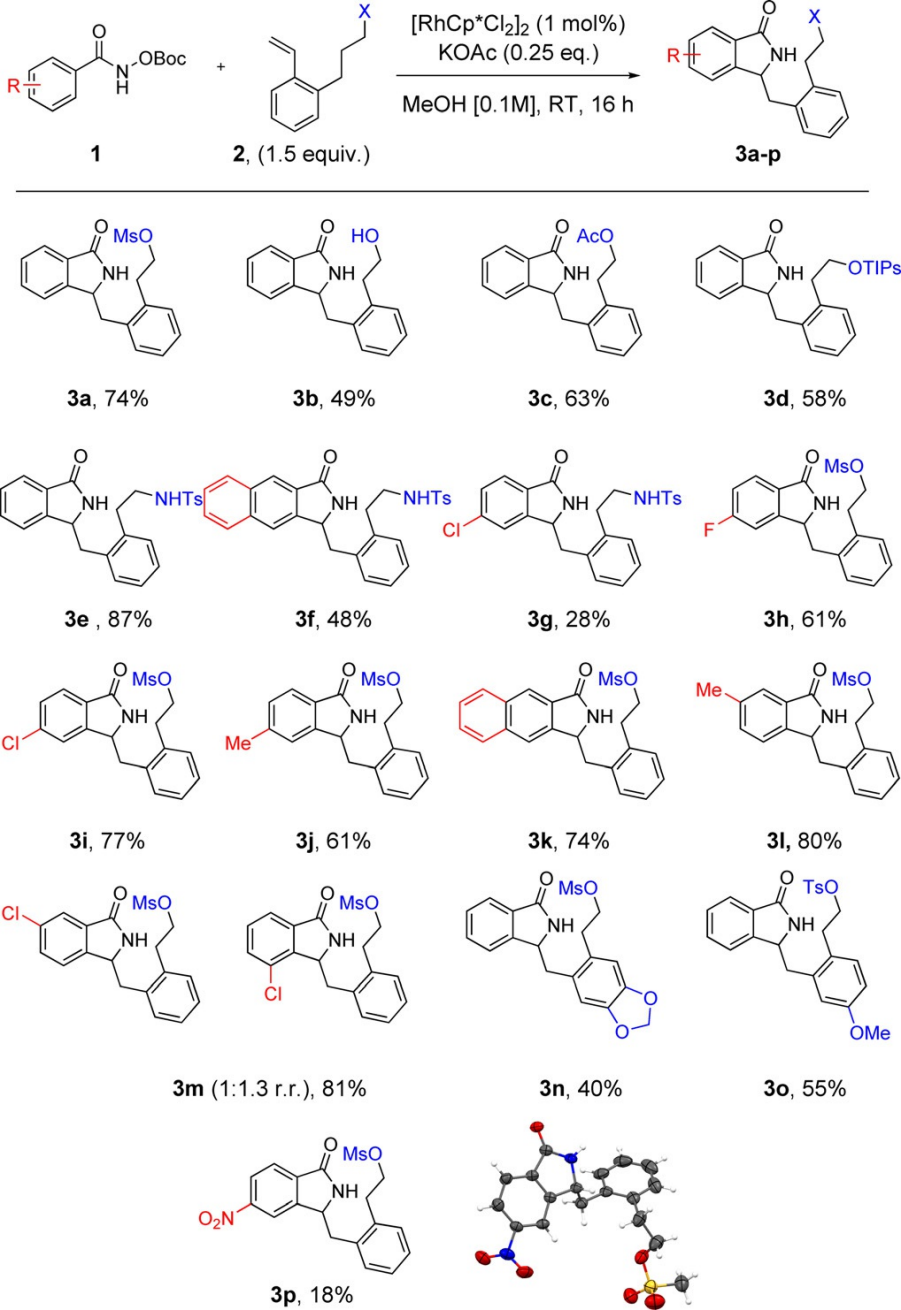
Scope of isoindolinone substrates.

For conversion of the isoindolinones into further compound classes, we explored whether the coordinating group in the final isoindolinone scaffold could be treated as a leaving group for ring closure. We developed a one‐pot‐two‐step reaction, where C−H functionalization is followed by subsequent treatment of the reaction mixture with base to afford isoindolobenzazepine products **4**. This scaffold is found in an alkaloid family isolated from *Berberis darwinii*.^12^ After brief optimization of the second step (see the Supporting Information for details), a series of these NP‐like compounds were synthesized (Scheme 4). Multiple aryl hydroxamates with electron donating and electron withdrawing groups were successfully converted into the desired isoindolobenzazepines (**4 a**–**r**) in moderate to very good yields. The *para*‐chloro product (**4 g**) allowed for structural confirmation by X‐Ray analysis (see the Supporting Information). Bromo‐ and iodo‐derivatives (**4 f** and **4 i**) were also tolerated providing opportunities for further synthetic elaboration. In addition, *meta*‐substituents (**4 m** and **4 n**) and di‐substitutions provided good yields of the desired products. Electron donating and withdrawing groups on the styrene also worked well affording the products in moderate yields (**4 o**, **4 q** and **4 r**). In particular, the CF_3_‐styrene provided the desired compound (**4 p**) in excellent yield.

**Scheme 4:**
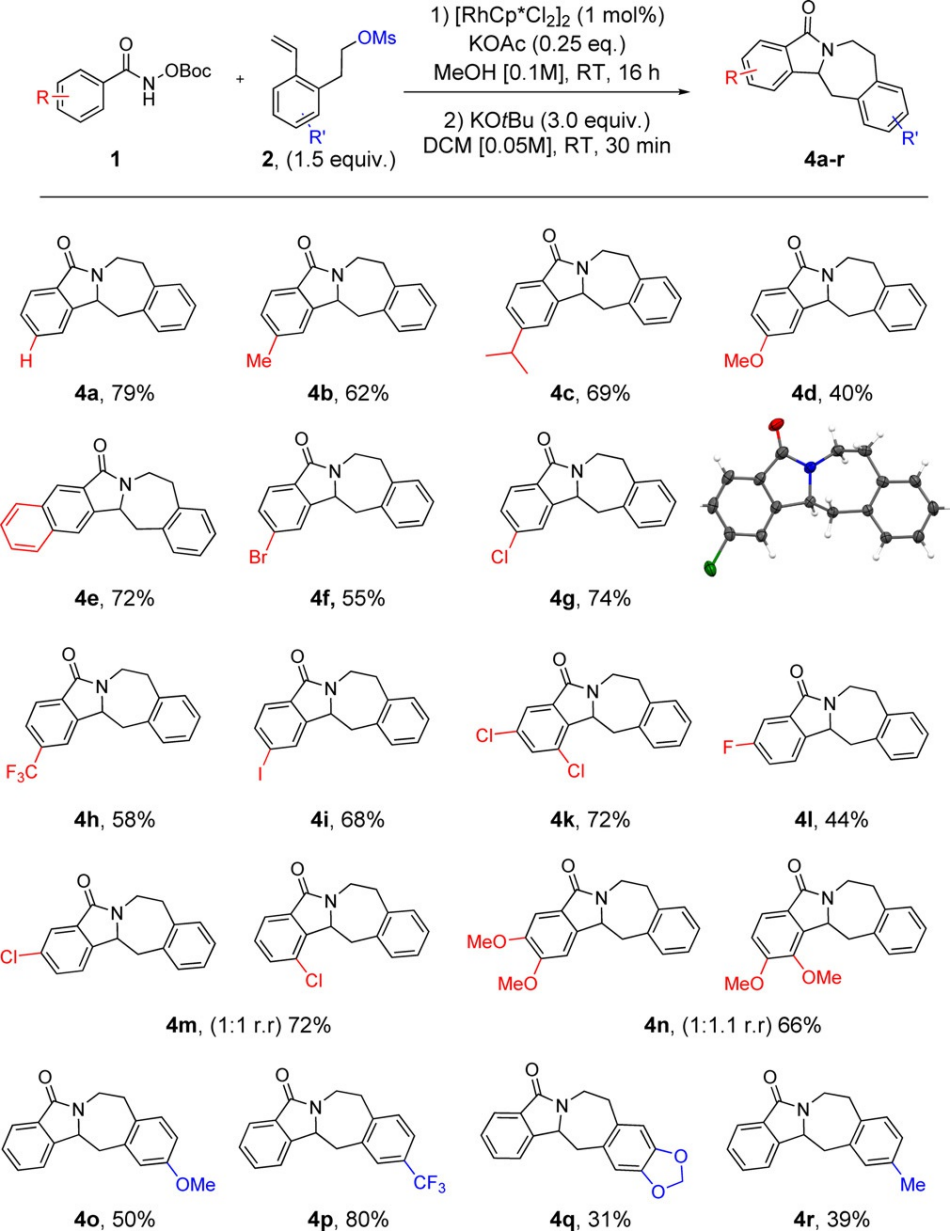
Scope of isoindolobenzazepine substrates.

To gain insight into the reaction mechanism, we performed the C−H functionalization using deuterium labelled styrene **2′** (Scheme 5, eq. 1). The reaction led to the formation of the desired product **3 q** with complete deuterium incorporation (from the styrene). In addition, submission of starting materials similar to the β‐hydride elimination intermediate to the optimized reaction conditions did not yield the cyclization product (Scheme 5, eqs. 2 and 3). These findings supported the notion that the reaction does not proceed *via* a Michael type addition which is in agreement with the fact that RhCp* does not catalyze a hydroamination reaction.^13^

**Scheme 5:**
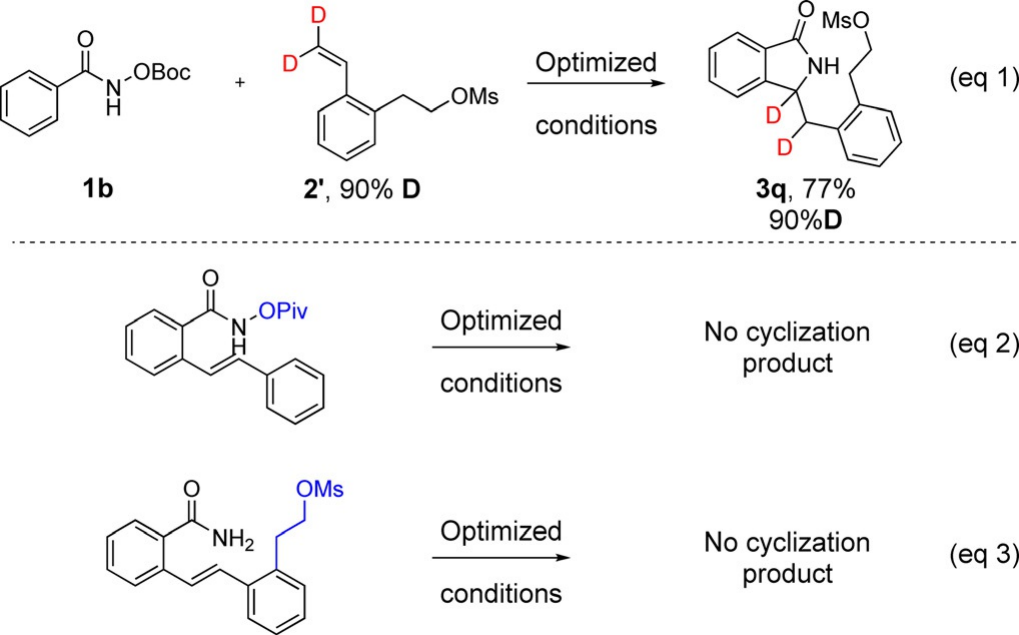
Mechanistic investigations.

Based on these findings and previous reports,5a, 14 we propose that the reaction starts with the formation of the active catalyst **I**. Oxidative addition affords Intermediate **II**. Insertion of the styrene would then give intermediate **III** which might undergo reductive elimination to dihydroisoquinolone **A**.14a However, in this case, intermediate **III** preferentially favors the β‐hydride elimination pathway leading to rhodium hydride species **IV**. This would then undergo migratory insertion into the olefin to yield the six‐membered ring rhodacycle **V**. Subsequent reductive elimination followed by N−O bond cleavage should afford the desired isoindolinone **3** (Scheme 6). For a deeper understanding of this mechanism, a computational analysis of the key intermediates using the *OPiv*‐aryl hydroxamate was performed (see the Supporting Information for details; Scheme 7). Notably, it indicates that the formation of key intermediate V* from III* is energetically favored over the pathway leading to dihydroisoquinolone **A**.

**Scheme 6:**
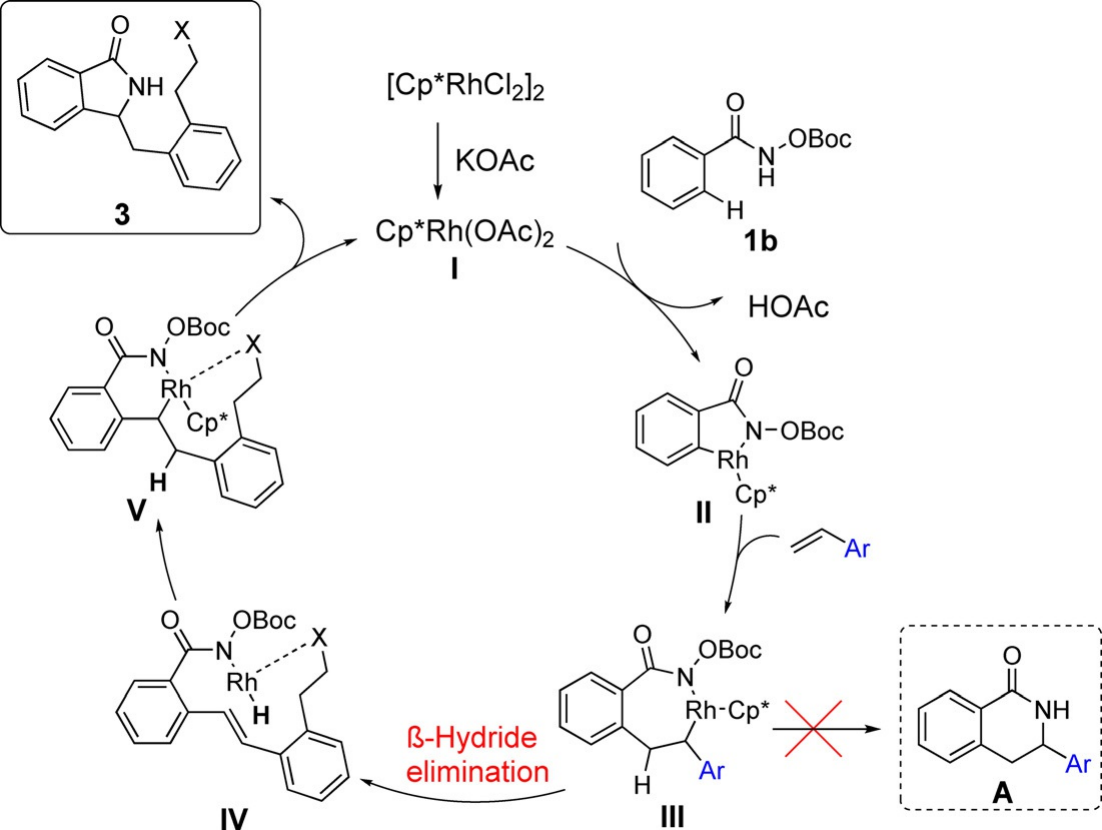
Proposed mechanism based on experimental findings.

**Scheme 7:**
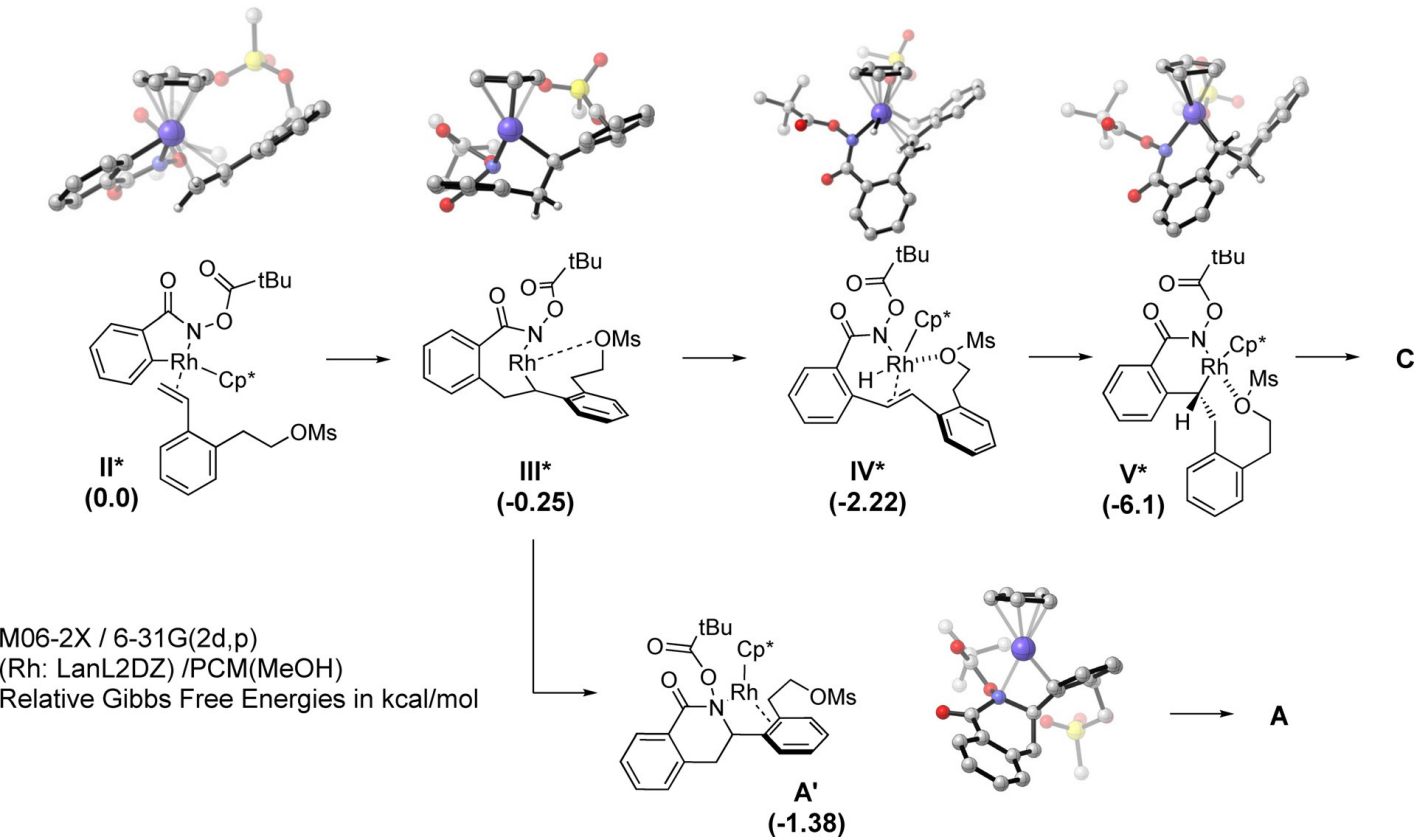
Calculated energies for the proposed intermediates with OPiv‐hydroxamate.

Isoindolinones are endowed with different bioactivities.^15^ Therefore the synthesized compounds were subjected to several cell‐based assays monitoring signal transduction through different cancer‐related pathways, as well as cellular processes such as autophagy. Interestingly, several derivatives inhibited Hedgehog (Hh)‐dependent differentiation of multipotent murine mesenchymal progenitor stem cells into osteoblasts.

Hh signaling is essential during embryonic development and is of importance for stem cell homeostasis and tissue regeneration.^16^ Abnormal regulation of Hh signaling is involved in severe birth defects and various types of cancer, including basal cell carcinoma and medulloblastoma.16a, 17 Consequently, the identification of Hh signaling inhibitors is of high interest. To investigate the effect of selected isoindolinones and isoindolobenzazepines on Hh signaling, a Hh‐dependent osteoblast differentiation assay using C3H10T1/2 cells was employed for primary screening.^17^, ^18^ The Hh pathway was activated using the Smoothened agonist purmorphamine. Active Hh signaling leads to differentiation of mesenchymal stem cells into osteoblasts and, thereby to expression of the osteoblast‐specific marker alkaline phosphatase. The activity of this enzyme serves as a measure of Hh‐dependent osteoblast differentiation.^19^ Remarkably, some isoindolinone derivatives inhibited this process with half‐maximal inhibitory concentrations (IC_50_) of 1.1±0.5 μm (**3 m**) and 2.1±1.1 μm (**3 g**; Figure 1 and the Supporting Information).

**Figure 1:**
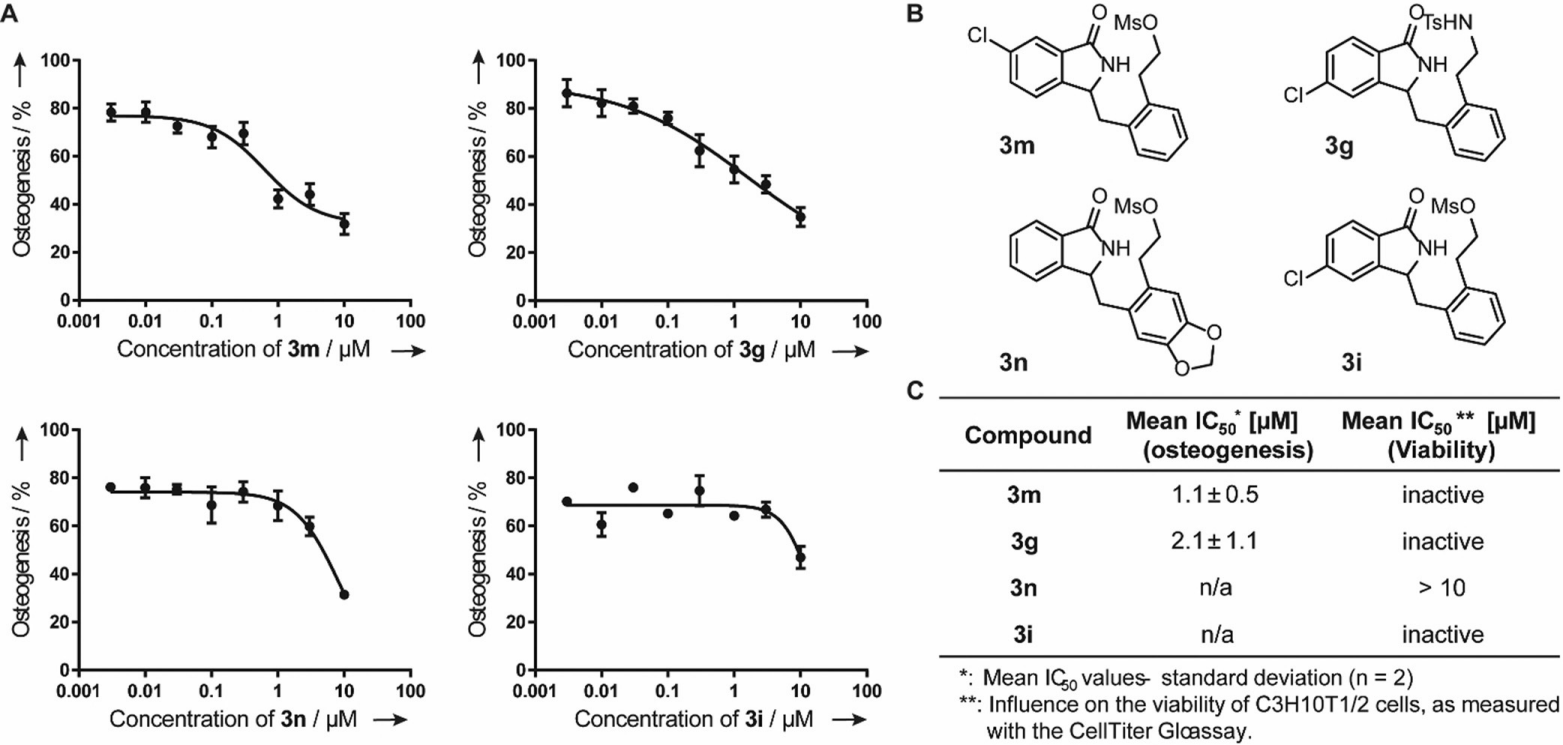
Influence of isoindolinones on Hh‐dependent osteoblast differentiation of C3H10T1/2 cells. A) C3H10T1/2 cells were treated with 1.5 μm purmorphamine and different concentrations of the compounds or DMSO as a control. After 96 h, the activity of alkaline phosphatase was detected by using a chemiluminescent substrate. The DMSO purmorphamine control was set to 100 %. Data are mean values ±SD and representative of two biological replicates, each performed in three technical replicates. B) Chemical structures of the isoindolinones **3 m**,**g**,**n**, and **3 i**. C) IC_50_ values of the isoindolinones, obtained from the respective dose‐response curve shown in (a) and the respective viability assays. To determine IC_50_ values, threefold dilutions starting from 10 μm were used. Compounds listed as “inactive” showed no effect at the tested concentrations in the respective assay and, therefore, no IC_50_ was determined.

In conclusion, we described the regiodivergent synthesis of isoindolinones *via* a Rh^III^‐catalyzed C−H functionalization of aryl hydroxamates with *ortho*‐substituted styrenes. Both, the OBoc‐protecting group and the presence of an *ortho*‐substituent with a coordinating substituent on the styrene proved to favor the formation of the five‐membered ring. This method also provides an alternative route for the synthesis of isoindolobenzazepine derivatives, the underlying scaffold of a family of NPs. Mechanistic experiments including DFT calculations support a mechanistic hypothesis for product formation *via* β‐hydride elimination followed by the formation of a six‐membered ring rhodacycle. Selected isoindolinones proved to be inhibitors of Hh‐dependent osteoblast differentiation.

## Conflict of interest

The authors declare no conflict of interest.

## Supporting information

As a service to our authors and readers, this journal provides supporting information supplied by the authors. Such materials are peer reviewed and may be re‐organized for online delivery, but are not copy‐edited or typeset. Technical support issues arising from supporting information (other than missing files) should be addressed to the authors.

Supplementary
