# Supplementary material for: RhIII‐Catalyzed C−H Activation of Aryl Hydroxamates for the Synthesis of Isoindolinones
Source: Chemistry. 2020 Jul 21;26(47):10729–34. doi: 10.1002/chem.202002384 (PMC7496876; doi:10.1002/chem.202002384)
Supplement: Supplementary file 1 — Supplementary [file CHEM-26-10729-s001.pdf]

Chemistry–A European Journal

Supporting Information

Rh^{III}-Catalyzed C–H Activation of Aryl Hydroxamates for the Synthesis of Isoindolinones

Saad Shaaban+,[a] Caitlin Davies+,[a, b] Christian Merten,[c] Jana Flegel,[a, b] Felix Otte,[d] Carsten Strohmann,[d] and Herbert Waldmann*[a, b]

Table of Contents

Table of Contents 1
1. General Information 2
2. Reaction Optimization 3
2.1 Solvent Screen 3
2.2 Base Screen 3
2.3 Cyclization step screen 4
3. General procedures and analytical data 5
3.1 General Procedure for the synthesis of *OBoc*-aryl-hydroxamates 1 5
3.2 General Procedure for the synthesis of styrenes 2 5
3.3 General Procedure for the synthesis of 3-Isoindolinones 3 8
3.4 General Procedure for the synthesis of Isoindolobenzazepine scaffolds 4 15
4. X-ray Structure Analyses 23
5. Biological data 25
6. DFT Calculations 27
7. References 39
8. NMR Spectra 40

SUPPORTING INFORMATION

1. General Information

Unless otherwise noted, all commercially available compounds were used as provided without further purification. Solvents for chromatography were technical grade.

Analytical thin-layer chromatography (TLC) was performed on Merck silica gel aluminium plates with F-254 indicator. Compounds were visualized by irradiation with UV light or potassium permanganate staining. Column chromatography was performed using silica gel Merck 60 (particle size 0.040-0.063 mm).

^{1}H-NMR, ^{13}C-NMR and ^{19}F-NMR were recorded on a Bruker DRX400 (400 MHz), Bruker DRX500 (500 MHz), INOVA500 (500 MHz) and Bruker DRX700 using $CDCl_3$ or CD_2Cl_2 as solvent. Data are reported in the following order: chemical shift (δ) values are reported in ppm with the solvent resonance as internal standard ($CDCl_3$: δ = 7.26 ppm for ^{1}H, δ = 77.16 ppm for ^{13}C, CD_2Cl_2: δ = 5.32 ppm for ^{1}H, δ = 54.00 ppm for ^{13}C). Multiplicities are indicated br s (broad singlet), s (singlet), d (doublet), t (triplet), q (quartet), m (multiplet); coupling constants (J) are given in Hertz (Hz).

High resolution mass spectra (HR-MS) were recorded on an LTQ Orbitrap mass spectrometer coupled to an Accela HPLC-System (HPLC column: Hypersyl GOLD, 50 mm x 1 mm, particle size 1.9 µm, ionization method: electron spray ionization). Fourier transform infrared spectroscopy (FT-IR) spectra were obtained with a Bruker Tensor 27 spectrometer (ATR, neat) and are reported in terms of frequency of absorption (cm^{-1}).

Acid chlorides were either commercially available or made from their corresponding carboxylic acid following the reported procedure. [1] 2-Bromostyrenes were also commercially available or made from their corresponding 2-Bromobenzadehyde following a modified version of the reported procedure. [2]

2. Reaction Optimization

2.1 Solvent Screen

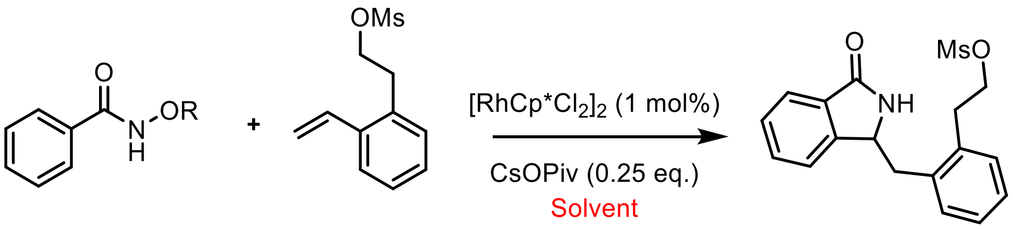

Entry	R	Solvent	Total yield (%)[a]	Ratio C: A
**1**	Piv	MeOH	84	8.3 : 1
**2**	Piv	DCM	46	5.6 : 1
**3**	Piv	MeCN	27	8.0 : 1
**4**	Piv	HFIP	0	—
**5**	Piv	Toluene	71	10.8 : 1
**6**	Piv	IPA	88	8.7 : 1
**7**	Piv	Hexafluorobenzene	48	5 : 1
**8**	Piv	MeOH (1.2 styrene)	86	8.6 : 1
**9**	Piv	MeOH (1.5 styrene)	93	6.8 : 1
**10**	Piv	MeOH (40 °C)	69	12.8 : 1
**11**	Boc	MeOH (2.0 styrene)	70	>99:1
12	**Boc**	**MeOH (1.5 styrene)**	**69[b]**	**>99:1**
**13**	Boc	MeOH (40 °C)	71	>99:1
**14**	Boc	TFE	73	>99:1
**15**	Boc	DCM	43	>99:1
**16**	Boc	TFE/DCM (1:1)	41	>99:1

[a] Determined by NMR using 1,3,5-trimethoxybenzene as internal standard (1.0 equiv). [b] Isolated yield

2.2 Base Screen

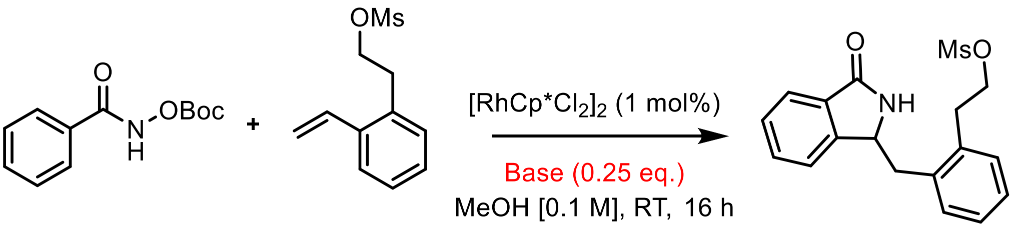

Entry	R	Solvent	Base	Yield (%)[a]
**1**	Boc	MeOH	CsOPiv	63
**2**	Boc	MeOH	KOPiv	60
**3**	Boc	MeOH	K_2CO_3	19
**4**	Boc	MeOH	CsOAc	77
**5**	Boc	MeOH	KOAc	84
**6**	Boc	MeOH	--	0

2.3 Cyclization step screen

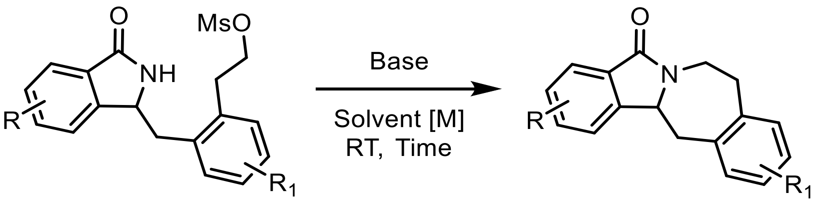

Entry	Solvent	Temp. (° C)	Base	Base equiv.	Conc. [M]	Time (mins)	Yield (%)[a]
**1**	THF	RT	KOtBu in THF	2.0	0.05	10	50
**2**	MeCN	RT	KOtBu	2.0	0.05	10	28
**3**	DCM	RT	KOtBu	2.0	0.05	10	53
**4**	DMSO	50	KOtBu	2.0	0.05	10	-
**5**	THF	60	KOtBu in THF	2.0	0.05	10	10
**6**	THF	RT	KOtBu in THF	1.0	0.05	10	29
**7**	DCM	RT	KOtBu	1.0	0.05	10	32
**8**	DCM	RT	KOtBu	2.0	0.10	10	60
**9**	DCM	RT	KOtBu	2.0	0.25	10	63
10	**DCM**	**RT**	**KOtBu**	**3.0**	**0.05**	**30**	**67[b]**
**11**	DCM	RT	KOtBu	4.0	0.05	10	52

[a] Determined by NMR using 1,3,5-trimethoxybenzene as internal standard (1.0 equiv). [b] Isolated yield

3. General procedures and analytical data

3.1 General Procedure for the synthesis of *OBoc*-aryl-hydroxamates 1

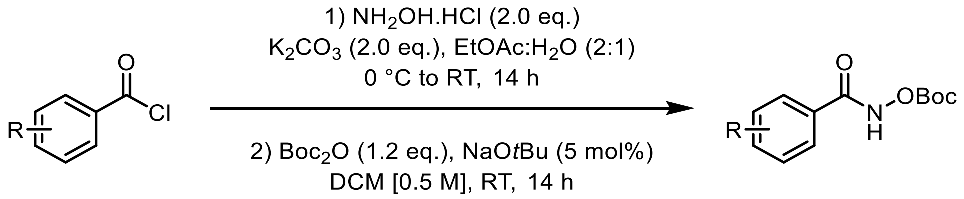

Following a modified procedure by Fagnou *et al.* [3], a flask was charged with hydroxylamine hydrochloride (1.5 equiv.), potassium carbonate (2.0 equivalent) and solvent (EtOAc/water; 2:1). The reaction was cooled to 0 °C with vigorous stirring before dropwise addition of acid chloride (1 equiv.) dissolved in EtOAc (2 ml). The reaction was stirred at RT for 14 h. The reaction mixture was diluted with water and extracted with EtOAc (2 x 20 ml). The combined organic layers were washed with brine (10 ml), dried over anhydrous sodium sulfate and concentrated *in vacuo*. The crude hydroxamic acid was used in the next step without further purification.

The hydroxamic acid (1.0 equiv.), NaO*t*Bu (5 mol%) and di-*tert*-butyl dicarbonate (1.2 equiv.) were dissolved in DCM [0.5 M]. The mixture was stirred at RT for 14 h. The reaction mixture was quenched with saturated $NaHCO_3$ solution (10 ml) and extracted with DCM (2x 10 ml). The organic layer was dried over Na_2SO_4, concentrated *in vacuo,* and then purified by silica gel chromatography (Pent/EtOAc, 5:1) to give the title products **1**.

All the aryl-hydroxamates are reported and their characterization matches the literature. [4]

3.2 General Procedure for the synthesis of styrenes 2

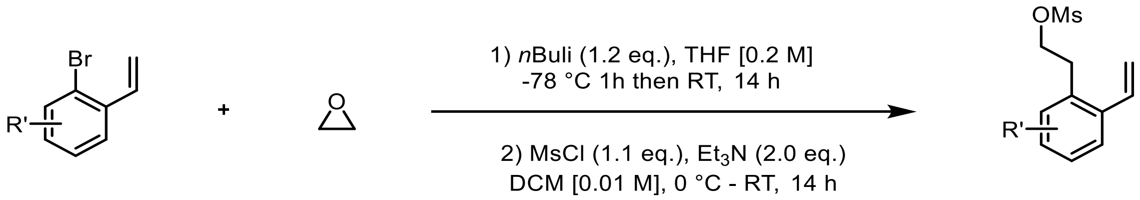

According to the reported procedure [5], a 100 mL round bottom flask was charged with 2-bromostyrene (1.0 eq.) and THF [0.2M]. The solution was then cooled to -78 °C and *n*-BuLi (1.2 eq., 1.6 M solution in hexanes) was added dropwise. The resulting mixture was then stirred at -78 °C for 1 h. Ethylene oxide (3.0 eq., 2.5 M solution in THF) was then added to the reaction mixture and stirred for an additional 0.5 h at -78 °C. The reaction was then warmed to RT and stirred for 14 h. A saturated solution of NH_4Cl (10 mL) was added, and the aqueous layer washed with Et_2O (3 x 25 mL). The combined organic layers were

dried over Na_2SO_4, filtered and concentrated *in vacuo* to afford the corresponding alcohol which was used in the next step without further purification.

The resulting alcohol (1.0 equiv.) and triethylamine (1.1 equiv.) were dissolved in DCM [0.01M] and cooled to 0°C. Then MsCl (1.1 equiv.) was added dropwise and the reaction was stirredat RT for 12h. The solvent was removed under reduced pressure and the crude material was purified by silica gel chromatography (Pent/EtOAc, 4:1) to afford the title styrenes **2**.

Some of the styrenes are reported and our characterization matches the literature. [6]

2-vinylphenethyl methanesulfonate (2a)

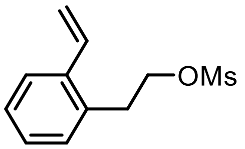

Colourless oil, 78% yield. **^{1}H-NMR (400 MHz – $CDCl_3$):** δ 7.56 – 7.46 (m, 1H), 7.29 – 7.25 (m, 1H), 7.25 – 7.22 (m, 1H), 7.21 – 7.18 (m, 1H), 6.97 (dd, *J* = 17.3, 11.0 Hz, 1H), 5.68 (dd, *J* = 17.3, 1.3 Hz, 1H), 5.37 (dd, *J* = 11.0, 1.3 Hz, 1H), 4.36 (t, *J* = 7.2 Hz, 2H), 3.15 (t, *J* = 7.2 Hz, 2H), 2.83 (s, 3H); **^{13}C-NMR (101 MHz – $CDCl_3$):** δ 137.3, 134.0, 133.4, 130.5, 128.2, 127.7, 126.4, 117.0, 69.6, 37.4, 33.1; **HR-MS:** calc. for $[M+H]^+$ $C_{11}H_{15}O_3S$: 227.07364, found: 227.07423

5-methyl-2-vinylphenethyl methanesulfonate (2b)

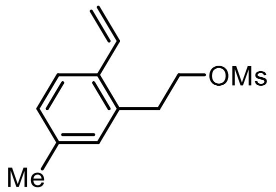

Colourless oil, 70% yield. **^{1}H-NMR (500 MHz - CD_2Cl_2):** δ 7.43 (d, J = 7.9 Hz, 1H), 7.11 – 7.04 (m, 1H), 7.03 (d, J = 1.7 Hz, 1H), 6.95 (dd, J = 17.3, 10.9 Hz, 1H), 5.65 (dd, J = 17.3, 1.3 Hz, 1H), 5.32 – 5.29 (m, 1H), 4.32 (t, J = 7.2 Hz, 2H), 3.10 (t, J = 7.2 Hz, 2H), 2.82 (s, 3H), 2.32 (s, 3H); **^{13}C-NMR (126 MHz - CD_2Cl_2):** δ 138.52, 134.71, 134.22, 133.90, 131.57, 128.81, 126.42, 116.00, 70.54, 37.60, 33.38, 21.31; **HR-MS:** calc. for $[M+H]^+$ $C_{12}H_{17}O_3S$: 241.08929, found: 241.08939.

2-(6-vinylbenzo[d][1,3]dioxol-5-yl)ethyl methanesulfonate (2c)

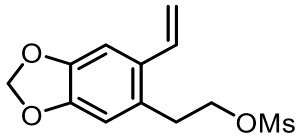

Colourless oil, 40% yield. **^{1}H-NMR (500 MHz - CD_2Cl_2):** δ 6.99 (s, 1H), 6.86 (dd, J = 17.2, 10.9 Hz, 1H), 6.66 (s, 1H), 5.95 (s, 2H), 5.55 (dd, J = 17.2, 1.1 Hz, 1H), 5.26 (dd, J = 10.9, 1.1 Hz, 1H), 4.30 (t, J = 7.2 Hz, 2H), 3.06 (t, J = 7.2 Hz, 2H), 2.89 (s, 3H); **^{13}C-NMR (126 MHz - CD_2Cl_2):** δ 147.6, 147.4,

133.4, 130.9, 127.3, 115.2, 110.2, 106.0, 101.3, 69.6, 37.5, 33.0; **HR-MS:** calc. for $[M+H]^+$ $C_{12}H_{15}O_5S$: 271.06347, found: 271.06357.

4-(trifluoromethyl)-2-vinylphenethyl methanesulfonate (2d)

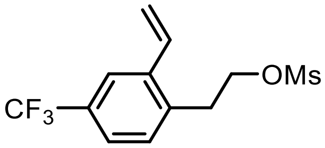

Colourless oil, 52% yield. **^{1}H-NMR (400 MHz - CDCl$_3$):** δ 7.66 (s. 1H), 7.43 (d, J = 7.9 Hz, 1H), 7.03 (d, J = 1.7 Hz, 1H), 6.95 (dd, J = 17.3, 10.9 Hz, 1H), 5.65 (dd, J = 17.3, 1.3 Hz, 1H), 5.32 – 5.29 (m, 1H), 4.32 (t, J = 7.2 Hz, 2H), 3.10 (t, J = 7.2 Hz, 2H), 2.82 (s, 3H); **^{13}C-NMR (101 MHz - CDCl$_3$):** δ 137.96, 137.23, 132.85, 130.78, 129.90, 124.55, 124.52, 123.29, 123.26, 118.80, 68.54, 37.48, 32.93.; **^{19}F-NMR (470 MHz, CDCl$_3$):** δ = -62.68.

2-(vinyl-2,2-d*2*)phenethyl-d*2* 4-methylbenzenesulfonate (2')

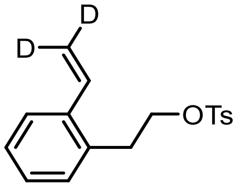

Colourless oil, 75% yield *(90% D)*. **^{1}H-NMR (500 MHz - CDCl$_3$):** δ 7.66 – 7.59 (m, 2H), 7.34 (dd, J = 7.6, 1.5 Hz, 1H), 7.24 – 7.17 (m, 2H), 7.14 (td, J = 7.5, 1.4 Hz, 1H), 7.10 (td, J = 7.4, 1.5 Hz, 1H), 7.01 (dd, J = 7.5, 1.4 Hz, 1H), 6.76 – 6.70 (m, 1H), 4.12 – 4.01 (m, 2H), 2.97 (t, J = 7.5 Hz, 2H), 2.37 (s, 3H); **^{13}C-NMR (126 MHz - CDCl$_3$):** δ 144.66, 144.62, 137.09, 133.72, 133.69, 133.61, 133.37, 133.17, 132.98, 130.27, 129.83, 129.82, 129.77, 127.97, 127.88, 127.84, 127.44, 127.17, 126.20, 126.19, 70.19, 69.78, 66.80, 32.80, 32.10, 21.63, 14.73.

3.3 General Procedure for the synthesis of 3-Isoindolinones 3

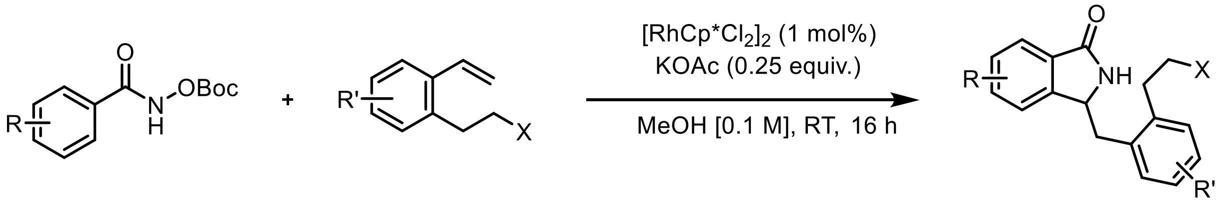

In a dram vial equipped with a stirring bar was added [RhCp*Cl_2]$_2$ (1.0 mol%), KOAc (0.025 mmol, 0.25 eq.), *N*-((*tert*-butoxycarbonyl)oxy)aryl-hydroxamate substrate (**1**) (0.1 mmol, 1.0 eq.) and dry MeOH [0.1 M]. The mixture was stirred for 30 seconds before the addition of the alkene substrate (**2**), (0.15 mmol, 1.5 eq.). The reaction was left to stir at room temperature for 16 h. The solvent was then evaporated under reduced pressure and the crude mixture was purified by silica gel chromatography using DCM to DCM: DMA* (10:1) as eluent to afford the title 3-Isoindolinones **3**.

*DMA: is a mixture of DCM:MeOH:NH_3 (100:5:0.15).

2-((3-oxoisoindolin-1-yl)methyl)phenethyl methanesulfonate (3a)

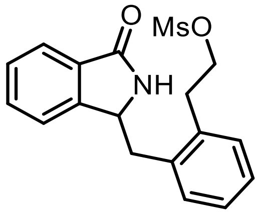

Colourless oil, 74% yield. **^{1}H-NMR (500 MHz - $CDCl_3$):** δ 7.86 (d, *J* = 7.3, 1.0 Hz, 1H), 7.58 (td, *J* = 7.5, 1.2 Hz, 1H), 7.51 (t, 1H), 7.39 (d, *J* = 7.6, 0.9 Hz, 1H), 7.30 – 7.26 (m, 4H), 6.40 (br s, 1H, NH), 4.80 (dd, *J* = 9.7, 4.9 Hz, 1H), 4.43 – 4.29 (m, 2H), 3.36 (dd, *J* = 14.0, 4.9 Hz, 1H), 3.13 (t, 2H), 2.91 (s, 3H), 2.81 (dd, *J* = 9.2, 4.8 Hz, 1H); **^{13}C-NMR (125 MHz - $CDCl_3$):** δ 170.36, 146.80, 135.87, 134.68, 132.18, 131.73, 130.66 (2C), 128.74, 128.00, 127.98, 124.16, 122.91, 69.71, 57.64, 38.18, 37.60, 32.75; **FT-IR:** ṽ = 3245, 2514, 2159, 2030, 1976, 1690, 1468, 1346, 1168, 947 cm^{-1}; **HR-MS:** calc. for $[M+H]^+$ $C_{18}H_{20}O_4NS$ 346.1107, found: 346.1108.

3-(2-(2-hydroxyethyl)benzyl)isoindolin-1-one (3b)

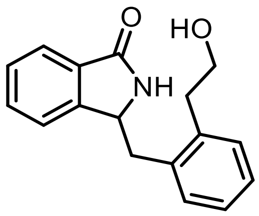

Red solid, 49% yield. **^{1}H-NMR (400 MHz - $CDCl_3$):** δ 7.80 (dt, J = 7.5, 1.0 Hz, 1H), 7.60 – 7.40 (m, 2H), 7.34 (d, J = 7.6 Hz, 1H), 7.26 – 7.13 (m, 4H), 7.11 (s, 1H), 4.77 (dd, J = 9.6, 4.7 Hz, 1H), 3.86 (tp, J = 7.7, 3.6 Hz, 2H), 3.27 (dd, J = 14.0, 4.7 Hz, 1H), 2.98 – 2.75 (m, 3H); **^{13}C-NMR (100 MHz - $CDCl_3$):** δ 161. 51, 147.07, 137.32, 135.97, 131.90, 130.52, 130.38, 130.15, 128.84, 128.46, 127.55, 127.43,

126.96, 123.98, 122.68, 63.17, 58.25, 38.16, 35.14; **FT-IR:** ṽ = 3059, 2348, 1680, 1542, 1492, 1449, 1153, 1094, 1062, 1011, 856 cm^{-1}; **LC-MS:** calc. for $[M+H]^+$ $C_{17}H_{17}O_2N$ 268.1, found: 268.1.

2-((3-oxoisoindolin-1-yl)methyl)phenethyl acetate (3c)

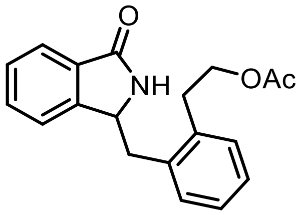

Red oil, 63% yield. **^{1}H-NMR (400 MHz - $CDCl_3$):** δ 7.79 (d, *J* = 7.3, 1.0 Hz, 1H), 7.48 (td, *J* = 7.5, 1.2 Hz, 1H), 7.42 (t, 1H), 7.30 (d, *J* = 7.7 Hz, 1H), 7.20 – 7.11 (m, 4H), 6.47 (br s, 1H, NH), 4.73 (dd, *J* = 9.5, 4.9 Hz, 1H), 4.17 – 4.11 (m, 2H), 3.32 (dd, *J* = 14.0, 4.9 Hz, 1H), 2.91 (t, 2H), 2.75 (dd, *J* = 9.2, 4.8 Hz, 1H), 1.93 (s, 3H); **^{13}C-NMR (101 MHz - $CDCl_3$):** δ171.08, 170.33, 146.88, 135.92, 135.70, 131.95, 131.73, 130.46, 130.40, 128.83, 128.51, 127.64, 127.44, 127.39, 124.01, 122.78, 119.50, 64.50, 57.62, 38.16, 31.94, 20.98; **FT-IR:** ṽ = 3045, 2486, 2159, 1976, 1690, 1468, 1289, 1163, 973cm^{-1}; **LC-MS:** calc. for $[M+H]^+$ $C_{19}H_{19}O_3N$ 309.3, found: 309.3.

3-(2-(2-((triisopropylsilyl)oxy)ethyl)benzyl)isoindolin-1-one (3d)

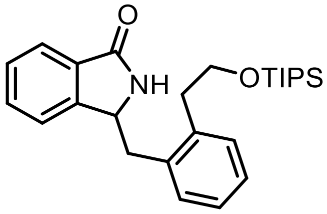

Red oil, 58% yield. **^{1}H-NMR (500 MHz - $CDCl_3$):** δ 7.83 – 7.78 (m, 1H), 7.57 – 7.39 (m, 3H), 7.39 – 7.11 (m, 4H), 6.31 (s, 1H), 4.76 (dd, J = 10.1, 4.9 Hz, 1H), 3.81 (t, J = 7.0 Hz, 2H), 3.32 (dd, J = 13.8, 4.8 Hz, 1H), 2.86 (td, J = 6.9, 2.1 Hz, 2H), 2.71 (dd, J = 13.9, 10.1 Hz, 1H), 1.05 – 0.96 (m, 3H), 0.99 – 0.78 (m, 18H); **^{13}C-NMR (125 MHz - $CDCl_3$):** δ 170.21, 147.00, 137.74, 135.64, 131.97, 131.57, 130.68, 130.02, 129.17, 128.85, 128.51, 127.47, 126.89, 124.06, 122.66, 64.50, 57.79, 38.41, 36.15, 17.93, 17.71, 13.67, 11.89.; **FT-IR:** ṽ = 3023, 2861, 1683, 1545, 1294, 1175, 1065, 1013, 849 cm^{-1}; **LC-MS:** calc. for $[M+H]^+$ $C_{26}H_{37}O_2NSi$ 432.2, found: 432.2.

4-methyl-N-(2-((3-oxoisoindolin-1-yl)methyl)phenethyl)benzenesulfonamide (3e)

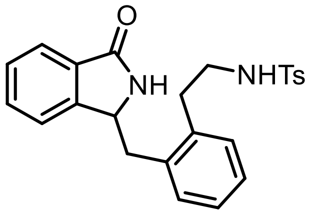

Red oil, 87% yield. **^{1}H-NMR (400 MHz - CDCl$_3$):** δ 7.74 (dd, J = 7.0, 1.3 Hz, 1H), 7.64 – 7.52 (m, 2H), 7.41 (dtd, J = 22.8, 7.4, 1.2 Hz, 3H), 7.21 (d, J = 7.4 Hz, 1H), 7.16 – 7.08 (m, 2H), 7.12 – 7.01 (m, 3H), 6.88 (s, 1H), 5.56 (t, J = 6.2 Hz, 1H), 4.62 (dd, J = 8.9, 5.6 Hz, 1H), 3.12 (dd, J = 13.8, 5.6 Hz, 1H), 3.09 – 2.96 (m, 2H), 2.91 – 2.75 (m, 2H), 2.71 (dd, J = 13.9, 8.9 Hz, 1H), 2.28 (s, 3H); **^{13}C-NMR (101 MHz - CDCl$_3$):** δ 170.65, 146.91, 143.36, 137.04, 136.57, 135.48, 132.09, 131.90, 131.66, 130.61, 130.10, 129.71, 128.81, 128.44, 127.65, 127.42, 127.24, 127.00, 123.91, 122.95, 57.68, 53.47, 43.86, 38.01, 33.17, 21.49; **FT-IR:** ṽ = 3592, 3057, 1681, 1486, 1320, 1152, 1017, 866, 815 cm^{-1}; **LC-MS:** calc. for $[M+H]^+$ $C_{24}H_{24}O_3N_2S$ 421.1, found: 421.1.

4-methyl-N-(2-((3-oxo-2,3-dihydro-1H-benzo[f]isoindol-1-yl)methyl)phenethyl)benzenesulfonamide (3f)

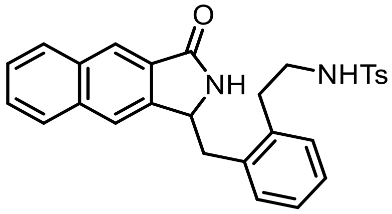

Red solid, 48% yield. **^{1}H-NMR (500 MHz - CDCl$_3$):** δ 8.33 (d, J = 4.8 Hz, 1H), 7.94 (d, J = 8.2 Hz, 1H), 7.81 (d, J = 8.2 Hz, 1H), 7.76 (s, 1H), 7.70 – 7.61 (m, 4H), 7.59 – 7.48 (m, 3H), 7.19 – 7.03 (m, 5H), 4.89 (d, J = 7.0 Hz, 1H), 3.21 (dd, J = 13.7, 5.8 Hz, 1H), 3.18 – 3.04 (m, 2H), 2.92 (dt, J = 55.9, 7.9 Hz, 3H), 2.27 (s, 3H).); **^{13}C-NMR (125 MHz - CDCl$_3$):** δ 170.61, 143.35, 141.50, 136.97, 136.72, 135.27, 132.94, 130.83, 130.17, 129.70, 129.63, 128.96, 128.26, 128.09, 127.79, 127.22, 127.01, 126.61, 124.63, 122.15, 58.03, 43.95, 38.54, 33.18, 29.73, 21.47; **FT-IR:** ṽ = 3630, 3057, 2549, 2148, 1685, 1492, 1321, 1207, 1073, 1017, 956, 905, 814 cm^{-1}; **LC-MS:** calc. for $[M+H]^+$ $C_{28}H_{26}O_3N_2S$ 471.1, found: 471.1.

N-(2-((6-chloro-3-oxoisoindolin-1-yl)methyl)phenethyl)-4-methylbenzenesulfonamide (3g)

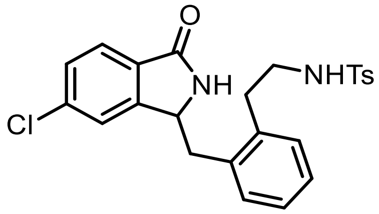

Yellow oil, 28% yield. **^{1}H-NMR (500 MHz - CDCl$_3$):** δ 7.74 (d, J = 8.1 Hz, 1H), 7.69 (d, J = 8.1 Hz, 2H), 7.46 (dd, J = 8.0, 1.7 Hz, 1H), 7.32 (d, J = 1.7 Hz, 1H), 7.25 – 7.19 (m, 4H), 7.16 (ddd, J = 15.4, 6.8, 2.1 Hz, 2H), 6.70 (s, 1H), 5.07 (s, 1H), 4.70 (dd, J = 9.4, 5.4 Hz, 1H), 3.21 (dd, J = 14.0, 5.4 Hz, 1H), 3.15 (dt, J = 9.8, 7.3 Hz, 2H), 2.88 (t, J = 7.4 Hz, 2H), 2.78 (dd, J = 14.0, 9.3 Hz, 1H), 2.38 (s, 3H); **^{13}C-NMR (125 MHz - CDCl$_3$):** δ 168.34, 147.35, 142.51, 137.43, 135.94, 135.41, 134.01, 129.54, 129.17, 129.02, 128.73 (2C), 128.13, 126.92, 126.42, 125.97 (2C), 124.20, 122.40, 56.29, 42.79, 36.86, 32.19,

20.47; **FT-IR:** ṽ = 3182, 2514, 2360, 2159, 2030, 1977, 1692, 1327, 1155, 925 cm^{-1}; **HR-MS:** calc. for $[M+H]^+$ $C_{24}H_{24}O_3N_2SCl$ 455.1190, found: 455.1189.

2-((6-fluoro-3-oxoisoindolin-1-yl)methyl)phenethyl methanesulfonate (3h)

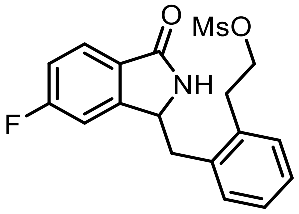

Colourless oil, 61% yield. **^{1}H-NMR (500 MHz - $CDCl_3$):** δ 7.83 (dd, J = 8.4, 5.0 Hz, 1H), 7.33 – 7.23 (m, 4H), 7.19 (td, J = 8.7, 2.2 Hz, 1H), 7.06 (dd, J = 8.2, 2.2 Hz, 1H), 6.39 (br s, 1H, NH), 4.77 (dd, J = 9.6, 5.0 Hz, 1H), 4.43 – 4.31 (m, 2H), 3.32 (dd, J = 14.0, 5.1 Hz, 1H), 3.13 (t, J = 7.8, 6.8 Hz, 2H), 2.93 (s, 3H), 2.87 – 2.77 (m, 1H); **^{13}C-NMR (125 MHz - $CDCl_3$):** δ 169.29, 166.46, 164.46, 149.23, 135.45, 134.68, 130.70, 128.15, 128.07, 127.80, 126.24, 116.57, 110.43, 69.64, 57.26, 38.08, 37.66, 32.81; **^{19}F-NMR (470 MHz, $CDCl_3$):** δ = -106.13 (q, J = 7.6); **HR-MS:** calc. for $[M+H]^+$ $C_{18}H_{19}O_4NFS$ 364.1013, found: 364.1013.

2-((6-chloro-3-oxoisoindolin-1-yl)methyl)phenethyl methanesulfonate (3i)

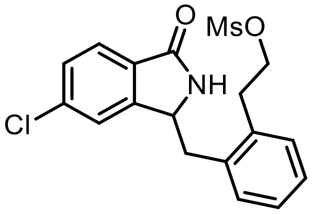

Yellow solid, 77% yield. **^{1}H-NMR (500 MHz - $CDCl_3$):** δ 7.78 (d, *J*=8.1 Hz, 1H), 7.48 (dd, *J*=8.1, 1.7 Hz, 1H), 7.39 (d, *J*=1.7 Hz, 1H), 7.34 – 7.27 (m, 4H), 6.27 (br s, 1H, NH), 4.78 (dd, *J*=9.9, 4.9 Hz, 1H), 4.43 – 4.35 (m, 2H), 3.34 (dd, *J*=14.0, 4.9 Hz, 1H), 3.14 (t, *J*=7.3 Hz, 2H), 2.94 (s, 3H), 2.84 – 2.78 (m, 1H); **^{13}C-NMR (125 MHz - $CDCl_3$):** δ 169.04, 148.21, 138.50, 135.31, 134.59, 130.58, 130.52, 130.12, 129.26, 128.07, 127.97, 125.32, 123.34, 69.48, 57.13, 37.97, 37.56, 32.65; **FT-IR:** ṽ = 3199, 2507, 2360, 2159, 2029, 1977, 1708, 1376, 1162, 946 cm^{-1}; **HR-MS:** calc. for $[M+H]^+$ $C_{18}H_{19}O_4NSCl$ 382.0685, found: 382.0688.

2-((6-methyl-3-oxoisoindolin-1-yl)methyl)phenethyl methanesulfonate (3j)

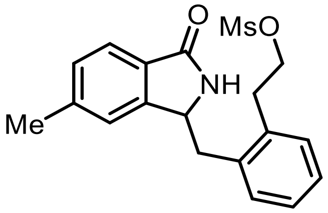

Colourless oil, 61% yield. **^{1}H-NMR (500 MHz - $CDCl_3$):** δ 7.73 (d, J=7.8 Hz, 1H), 7.33 – 7.30 (d, 1H), 7.28 (m, 4H), 7.21 (d, J=1.6 Hz, 1H), 6.25 (br s, 1H, NH), 4.74 (dd, J=10.0, 4.6 Hz, 1H), 4.37 (m, 2H), 3.35 (dd, J=14.0, 4.6 Hz, 1H), 3.13 (t, J=7.3 Hz, 2H), 2.91 (s, 3H), 2.75 (dd, J=14.0, 10.0 Hz, 1H), 2.47 (s, 3H); **^{13}C-NMR (125 MHz - $CDCl_3$):** δ 170.47, 147.27, 142.97, 136.02, 134.68, 130.63, 130.59, 129.74, 129.11, 127.99, 127.94, 123.92, 123.35, 69.73, 57.48, 38.26, 37.57, 32.69, 22.13; **FT-IR:** ṽ = 3232, 2513, 2159, 2029, 1977, 1690, 1618, 1346, 1168, 947 cm^{-1}; **HR-MS:** calc. for $[M+H]^+$ $C_{19}H_{22}O_4NS$ 360.1264, found: 360.1264.

2-((3-oxo-2,3-dihydro-1H-benzo[f]isoindol-1-yl)methyl)phenethyl methanesulfonate (3k)

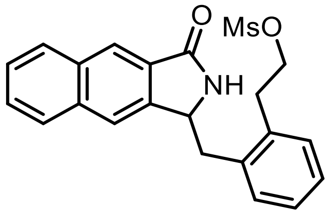

Yellow oil, 74% yield. **^{1}H-NMR (500 MHz - $CDCl_3$):** δ 8.40 (s, 1H), 8.03 (d, J = 8.1 Hz, 1H), 7.92 (d, J = 8.1 Hz, 1H), 7.82 (s, 1H), 7.62 (td, J = 8.2, 6.8, 1.4 Hz, 1H), 7.58 (td, J = 8.1, 6.8, 1.4 Hz, 1H), 7.31 (d, J = 2.5 Hz, 4H), 6.56 (br s, 1H, NH), 4.97 (dd, J = 9.7, 4.9 Hz, 1H), 4.47 – 4.30 (m, 2H), 3.46 (dd, J = 14.0, 4.9 Hz, 1H), 3.23 – 3.11 (m, 2H), 2.92 (dd, 1H), 2.91 (s, 3H); **^{13}C-NMR (125 MHz - $CDCl_3$):** δ 170.13, 141.45, 135.67, 135.32, 134.64, 133.08, 130.63, 130.58, 129.67, 129.18, 128.26, 128.09, 127.96 (2C), 126.68, 124.64, 121.93, 69.62, 57.56, 38.78, 37.49, 32.67; **FT-IR:** ṽ = 3189, 2928, 2522, 2360, 2159, 2029, 1977, 1690, 1639, 1342, 1178 cm^{-1}; **HR-MS:** calc. for $[M+H]^+$ $C_{22}H_{22}O_4NS$ 396.1264, found: 396.1260.

2-((5-methyl-3-oxoisoindolin-1-yl)methyl)phenethyl methanesulfonate (3l)

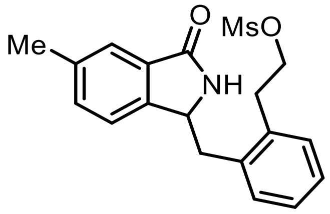

Colourless oil, 80% yield. **^{1}H-NMR (500 MHz - $CDCl_3$):** δ 7.64 (d, J = 1.7 Hz, 1H), 7.36 (dd, J = 7.7, 1.6 Hz, 1H), 7.29 – 7.26 (m, 1H), 7.26 – 7.23 (m, 3H), 7.21 (d, J = 7.7 Hz, 1H), 6.58 (br s, 1H, NH), 4.75 (dd, J = 9.5, 5.2 Hz, 1H), 4.34 (m, 2H), 3.28 (dd, J = 14.0, 5.2 Hz, 1H), 3.10 (t, J = 7.3 Hz, 2H), 2.89 (s, 3H), 2.81 (dd, J = 14.0, 9.4 Hz, 1H); **^{13}C-NMR (125 MHz - $CDCl_3$):** δ 170.61, 144.14, 138.82, 135.93, 134.72, 133.16, 131.82, 130.65, 130.60, 127.94, 127.89, 124.32, 122.64, 69.71, 57.57, 38.24, 37.57, 32.72, 21.48; **FT-IR:** ṽ = 3189, 2522, 2362, 2159, 2029, 1977, 1690, 1343, 1168, 947 cm^{-1}; **HR-MS:** calc. for $[M+H]^+$ $C_{19}H_{22}O_4NS$ 360.1264, found: 360.1264.

2-((5-chloro-3-oxoisoindolin-1-yl)methyl)phenethyl methanesulfonate (3m)

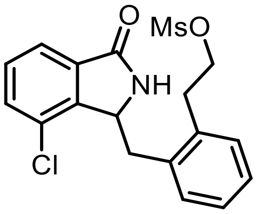

A mixture of regioisomers (1:1.3) were isolated as a colourless oil (81%).

Major isomer: **^{1}H-NMR (500 MHz - CDCl$_3$):** δ 7.75 (d, J = 7.5 Hz, 1H), 7.59 (d, J = 7.9 Hz, 1H), 7.48 (t, J = 7.7 Hz, 1H), 7.27 (m, 3H), 7.24 (m, 1H), 6.47 (br s, 1H, NH), 4.83 (dd, J = 10.4, 3.1 Hz, 1H), 4.43 (t, J = 7.1 Hz, 2H), 3.98 (dd, J = 14.2, 3.1 Hz, 1H), 3.22 (m, 2H), 2.92 (s, 3H), 2.56 (dd, J = 14.1, 10.3 Hz, 1H); **^{13}C-NMR (125 MHz - CDCl$_3$):** δ 167.75, 142.53, 134.60, 133.80, 133.17, 132.48, 131.69, 129.52, 129.29, 128.42, 126.87, 126.82, 121.64, 68.63, 56.31, 36.37, 34.61, 31.36; **FT-IR:** ṽ = 3198, 2515, 2360, 2159, 2028, 1977, 1693, 1344, 1166, 947 cm^{-1}; **HR-MS:** calc. for [M+H]$^{+}$ $C_{18}H_{18}O_4NSCl$ 382.0685, found: 382.0688.

Minor isomer: **^{1}H-NMR (500 MHz - CDCl$_3$):** δ 7.80 (d, J = 1.9 Hz, 1H), 7.52 (dd, J = 8.1, 2.0 Hz, 1H), 7.27 (m, 3H), 7.24 (m, 2H), 6.63 (br s, 1H, NH), 4.78 (dd, J = 9.4, 5.3 Hz, 1H), 4.36 (m, 2H), 3.30 (dd, J = 14.0, 5.3 Hz, 1H), 3.11 (t, J = 7.3 Hz, 2H), 2.92 (s, 3H), 2.84 (dd, J = 14.4, 9.1 Hz, 1H); **^{13}C-NMR (125 MHz - CDCl$_3$):** δ 167.81, 143.75, 134.36, 133.87, 133.56, 131.13, 129.59 (2C), 129.56, 126.96, 126.89 – 126.88 (m), 123.16, 123.15, 68.53, 56.27, 36.94, 36.50, 31.67; **FT-IR:** ṽ = 3198, 2515, 2360, 2159, 2028, 1977, 1693, 1344, 1166, 947 cm^{-1}; **HR-MS:** calc. for [M+H]$^{+}$ $C_{18}H_{19}O_4NSCl$ 382.0685, found: 382.0688.

2-(6-((3-oxoisoindolin-1-yl)methyl)benzo[d][1,3]dioxol-5-yl)ethyl methanesulfonate (3n)

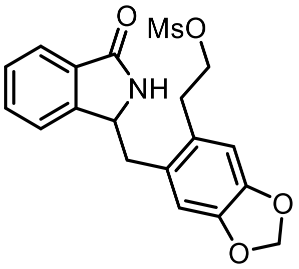

Colourless oil, 40% yield. **^{1}H-NMR (500 MHz - CDCl$_3$):** δ 7.86 (d, J = 7.5, 1.2 Hz, 1H), 7.59 (t, J = 7.5, 1.2 Hz, 1H), 7.51 (t, J = 7.5 Hz, 1H), 7.41 (d, J = 7.6, 1.0 Hz, 1H), 6.73 (d, J = 2.0 Hz, 2H), 6.41 (br s, 1H, NH), 5.98 – 5.96 (m, 2H), 4.75 (dd, J = 9.7, 4.9 Hz, 1H), 4.35 – 4.23 (m, 2H), 3.25 (dd, J = 14.2, 5.0 Hz, 1H), 3.02 (t, J = 7.3 Hz, 2H), 2.96 (s, 3H), 2.71 (dd, J = 14.2, 9.7 Hz, 1H); **^{13}C-NMR (125 MHz - CDCl$_3$):** δ 169.24, 146.21, 146.19, 145.55, 131.14, 130.46, 127.81, 127.64, 126.65, 123.08, 121.73, 109.19, 109.14, 100.35, 68.50, 56.82, 36.94, 36.55, 31.64; **FT-IR:** ṽ = 3182, 2516, 2159, 2030, 1977, 1694, 1502, 1485, 1367, 1276, 1166, 1039, 976 cm^{-1}; **HR-MS:** calc. for [M+H]$^{+}$ $C_{19}H_{20}O_6NS$ 390.1007, found: 390.1005.

4-methoxy-2-((3-oxoisoindolin-1-yl)methyl)phenethyl 4-methylbenzenesulfonate (3o)

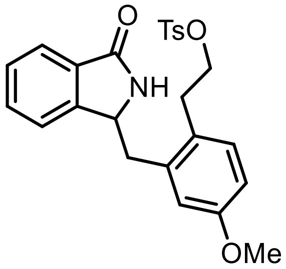

Pink oil, 55% yield. **^{1}H-NMR (600 MHz - $CDCl_3$):** δ 7.81 (d, J = 7.5, 1.2 Hz, 1H), 7.60 (d, J = 7.4 Hz, 2H), 7.52 (t, J = 7.5, 1.2 Hz, 1H), 7.42 (t, J = 7.5 Hz, 1H), 7.29 – 7.18 (m, 1H), 7.16 – 6.99 (m, 2H), 6.81 (d, J = 2.0 Hz, 2H), 6.73, (d, J = 7.5 Hz, 1H), 6.66 (br s, 1H, NH), 4.69 (dd, J = 9.7, 4.9 Hz, 1H), 4.07 – 4.05 (m, 2H), 3.72 (s, 3H), 3.58 (dd, J = 14.2, 5.0 Hz, 1H), 3.02 (t, J = 7.3 Hz, 1H), 2.89 – 2.68 (m, 2H), 2.32 (s, 3H); **^{13}C-NMR (167 MHz - $CDCl_3$):** δ 170.52, 158.90, 146.68, 144.86, 136.67, 132.91, 132.41, 131.59, 131.04, 129.83, 128.85, 128.74, 127.76, 127.44, 126.49, 124.18, 122.84, 115.80, 113.20, 70.21, 58.12, 55.35, 38.07, 31.65, 31.45, 29.71, 21.63; **FT-IR:** ṽ = 2923, 1684, 1609, 1466, 1291, 1188, 1096, 1010, 897, 814 cm^{-1}; **LC-MS:** calc. for $[M+H]^+$ $C_{25}H_{25}O_5NS$ 452.3, found: 452.3.

2-((6-nitro-3-oxoisoindolin-1-yl)methyl)phenethyl methanesulfonate (3p)

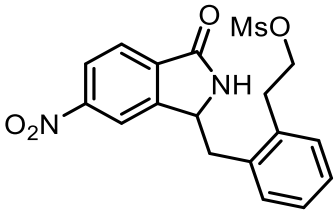

Yellow solid, 18% yield. **^{1}H-NMR (500 MHz - $CDCl_3$):** δ 8.39 (dd, J = 8.2, 2.0 Hz, 1H), 8.29 – 8.25 (m, 1H), 8.01 (d, J = 8.3, 0.6 Hz, 1H), 7.37 – 7.28 (m, 3H), 7.26 – 7.23 (m, 1H), 6.50 (br s, 1H, NH), 4.91 (dd, J = 9.7, 5.0 Hz, 1H), 4.45 – 4.37 (m, 2H), 3.43 (dd, J = 14.0, 5.1 Hz, 1H), 3.16 (t, J = 7.3 Hz, 2H), 2.97 (s, 3H), 2.89 – 2.85 (m, 1H); **^{13}C-NMR (125 MHz - $CDCl_3$):** δ 167.60, 150.44, 147.44, 137.06, 134.80, 134.61, 130.68, 130.59, 128.33, 128.10, 125.24, 124.32, 118.67, 69.44, 57.41, 37.83, 37.62, 32.73; **FT-IR:** ṽ = 3192, 2929, 2523, 2360, 2159, 2030, 1977, 1715, 1690, 1525, 1342, 1163, 976 cm^{-1}; **HR-MS:** calc. for $[M+H]^+$ $C_{18}H_{19}O_6N_2S$ 391.0959, found: 391.0958.

2-((3-oxoisoindolin-1-yl-1-*d*)methyl-d)phenethyl-*d*2 4-methylbenzenesulfonate (3q)

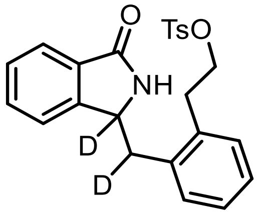

Colorless oil, 67% yield. **^{1}H-NMR (600 MHz - CDCl$_3$):** δ 7.76 (d, J = 7.5, 1.2 Hz, 1H), 7.59 (d, J = 7.4 Hz, 2H), 7.49 – 7.39 (m, 2H), 7.19 – 7.08 (m, 6H), 6.57 (br s, 1H, NH), 4.14 – 4.04 (m, 2H), 3.12 (s, 1H), 2.96 – 2.92 (m, 2H), 2.35 (dd, J = 14.2, 5.0 Hz, 1H), 2.32 (s, 3H); **^{13}C-NMR (151 MHz - CDCl$_3$):** δ 170.47, 146.67, 144.90, 144.66, 135.62, 134.64, 133.59, 133.16, 132.90, 132.82, 131.97, 131.70, 130.45, 130.38, 130.27, 129.86, 129.79, 128.78, 128.55, 127.97, 127.87, 127.82, 127.75, 127.73, 127.65, 127.44, 126.19, 123.97, 122.82, 119.31, 77.29, 69.99, 69.81, 66.84, 32.78, 32.28, 21.63, 14.73; **FT-IR:** ṽ = 3061, 1684, 1692, 1597, 1493, 1354, 1188, 1095, 1010, 960, 815 cm^{-1}; **LC-MS:** calc. for [M+H]$^+$ $C_{24}H_{21}D_2O_2NS$ 424.1, found: 424.1.

3.4 General Procedure for the synthesis of Isoindolobenzazepine scaffolds 4

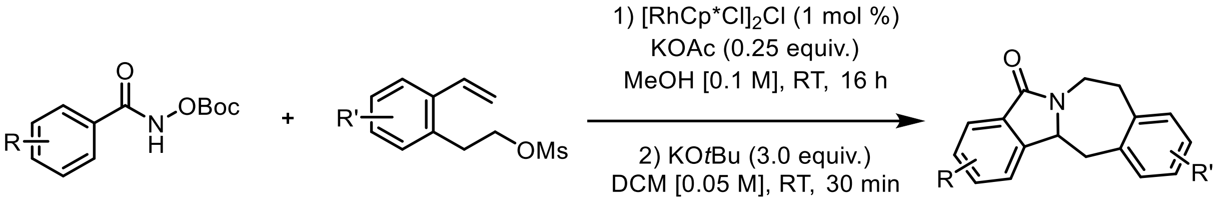

In a dram vial equipped with a stirring bar was added [RhCp*Cl$_2$]$_2$ (1.0 mol%), KOAc (0.0025 mmol, 0.25 eq.), *N*-((*tert*-butoxycarbonyl)oxy)aryl-hydroxamate substrate (**1**) (0.1 mmol, 1.0 eq.) and dry MeOH [0.1 M]. The mixture was stirred for 30 seconds before the addition of the alkene substrate (**2**), (0.15 mmol, 1.5 eq.). The reaction was left to stir at room temperature for 16 h. The solvent was then evaporated under reduced pressure. Dry DCM [0.05 M] was added to the crude mixture followed by *t*BuOK (0.30 mmol, 3.0 eq.). The reaction mixture was stirred for 30 minutes at room temperature. The reaction was then quenched with H_2O and extracted with DCM (2 x 5 mL). The organic layer was washed with brine, dried over $MgSO_4$, filtered, and the solvent was evaporated. The crude material was purified by silica gel chromatography (Pent/EtOAc, 4:1) to afford the title compounds **4**.

8,13-dihydro-6,13-methanodibenzo[c,f]azonin-5(7H)-one (4a)

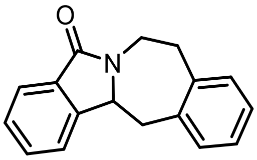

White solid, 79% yield. **^{1}H-NMR (500 MHz - CDCl$_3$):** 7.82 (d, *J* = Hz, 1H), 7.55-7.48 (m, 2H), 7.43 (t, *J* = Hz, 1H), 7.62-7.22 (m, 1H), 7.19-7.13 (m, 3H), 4.80-4.72 (m, 1H), 4.38 (d, *J* = Hz, 1H), 3.22 (d, J = Hz, 1H), 3.00-2.81 (m, 4H); **^{13}C-NMR (125 MHz - CDCl$_3$):** 167.1, 144.9, 141.4, 137.8, 131.9, 131.6, 129.9 (2C),128.4, 127.4, 123.8, 122.2, 61.3, 42.3, 41.3, 36.2; **FT-IR:** ṽ = 2910, 2508, 2159, 2029, 1976, 1679, 1451 cm^{-1}; **HR-MS:** calc. for [M+H]$^+$ $C_{17}H_{16}ON$ = 250.1226 found: 250.1227.

2-methyl-7,8,13,13a-tetrahydro-5H-benzo[4,5]azepino[2,1-a]isoindol-5-one (4b)

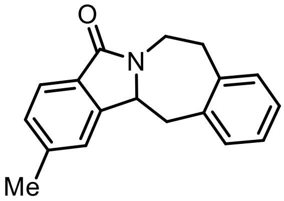

White solid, 62% yield. **^{1}H-NMR (500 MHz - $CDCl_3$):** 7.77 (d, *J* = 7.7 Hz, 1H), 7.36 (s, 1H), 7.32 – 7.28 (m, 2H), 7.26 – 7.19 (m, 3H), 4.86 – 4.74 (m, 1H), 4.42 – 4.35 (d, 1H), 3.27 (dd, *J* = 14.6, 1.7 Hz, 1H), 3.05 – 2.88 (m, 4H), 2.50 (s, 3H); **^{13}C-NMR (125 MHz - $CDCl_3$):** 167.20, 145.28, 142.19, 141.50, 137.94, 129.88 (2C), 129.42, 127.43, 127.05, 123.59, 122.63, 60.99, 42.68, 41.17, 36.38, 21.98; **FT-IR:** ṽ = 2921, 2513, 2159, 2030, 1976, 1676, 1415 cm^{-1}; **HR-MS:** calc. for $[M+H]^+$ $C_{18}H_{18}ON$ = 264.1382 found: 264.1384.

2-isopropyl-7,8,13,13a-tetrahydro-5H-benzo[4,5]azepino[2,1-a]isoindol-5-one (4c)

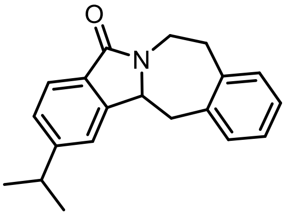

White solid, 69% yield. **^{1}H-NMR (500 MHz - $CDCl_3$):** 7.80 (d, *J* = 7.8 Hz, 1H), 7.39 (d, *J* = 1.4 Hz, 1H), 7.36 (dd, *J* = 7.8, 1.4 Hz, 1H), 7.34 – 7.31 (m, 1H), 7.26 – 7.21 (m, 3H), 4.81 (dd, *J* = 11.6, 5.5 Hz, 1H), 4.44 – 4.35 (m, 1H), 3.29 (dd, *J* = 14.6, 1.7 Hz, 1H), 3.04 – 2.98 (m, 3H), 2.96 – 2.88 (m, 2H), 1.33 (d, *J* = 6.9 Hz, 6H); **^{13}C-NMR (125 MHz - $CDCl_3$):** 167.18, 153.26, 145.27, 141.53, 137.99, 129.90, 129.87, 129.82, 127.42, 127.04, 127.00, 123.70, 119.93, 61.13, 42.69, 41.18, 36.42, 34.69, 24.09 (2C); **FT-IR:** ṽ = 2962, 2514, 2159, 2029, 1977, 1671, 1417 cm^{-1}; **HR-MS:** calc. for $[M+H]^+$ $C_{20}H_{22}ON$ = 292.1695 found: 292.1697.

2-methoxy-7,8,13,13a-tetrahydro-5H-benzo[4,5]azepino[2,1-a]isoindol-5-one (4d)

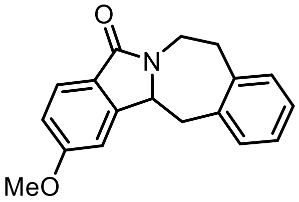

White solid, 40% yield. **^{1}H-NMR (500 MHz - $CDCl_3$):** 7.80 (d, *J* = 8.3 Hz, 1H), 7.31 (dd, *J* = 5.3, 3.5 Hz, 1H), 7.25 (dd, *J* = 5.6, 3.3 Hz, 2H), 7.24 – 7.20 (m, 1H), 7.03 (d, *J* = 2.1 Hz, 1H), 7.01 (dd, *J* = 8.3, 2.2 Hz, 1H), 4.83 – 4.75 (m, 1H), 4.38 (d, *J* = 10.9 Hz, 1H), 3.92 (s, 3H), 3.24 (dd, *J* = 14.7, 1.7 Hz, 1H), 3.06 – 2.97 (m, 2H), 2.96 – 2.89 (m, 2H); **^{13}C-NMR (125 MHz - $CDCl_3$):** 165.92, 161.85, 146.08, 140.47, 136.80, 128.86 (2C), 126.43, 126.00, 124.14, 123.56, 113.61, 106.32, 59.88, 54.72, 41.77, 40.16, 35.42;

FT-IR: ṽ = 2919, 2515, 2159, 2027, 1666, 1610, 1414 cm^{-1}; **HR-MS:** calc. for $[M+H]^+$ $C_{18}H_{18}O_2N$ = 280.1332 found: 280.1334.

8,9,14,14a-tetrahydro-6H-benzo[f]benzo[4,5]azepino[2,1-a]isoindol-6-one (4e)

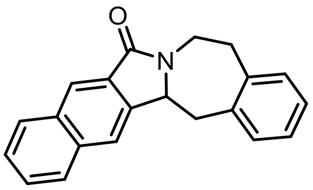

Yellow powder, 72% yield. **^{1}H-NMR (500 MHz - $CDCl_3$):** 8.40 (s, 1H), 8.03 (d, 1H), 7.99 (s, 1H), 7.96 (d, *J* = 8.2, 1.3 Hz, 1H), 7.64 – 7.52 (m, 2H), 7.38 – 7.32 (m, 1H), 7.31 – 7.27 (m, 1H), 7.26 – 7.21 (m, 2H), 4.95 – 4.79 (m, 1H), 4.60 (d, *J* = 11.1, 1.4 Hz, 1H), 3.40 (dd, *J* = 14.7, 1.8 Hz, 1H), 3.13 – 2.92 (m, 4H); **^{13}C-NMR (125 MHz - $CDCl_3$):** 165.87, 140.34, 139.15, 136.86, 134.14, 132.16, 128.91, 128.81, 128.75, 128.53, 127.07, 126.64, 126.43, 126.05, 125.40, 122.96, 120.09, 60.01, 42.44, 40.44, 35.32; **FT-IR:** ṽ = 2923, 2519, 2031, 1976, 1686, 1418 cm^{-1}; **HR-MS:** calc. for $[M+H]^+$ $C_{21}H_{18}ON$ = 300.1382 found: 300.1386.

2-bromo-7,8,13,13a-tetrahydro-5H-benzo[4,5]azepino[2,1-a]isoindol-5-one (4f)

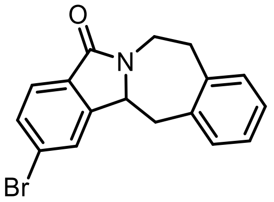

White solid, 55% yield. **^{1}H-NMR (700 MHz - $CDCl_3$):** δ 7.68 – 7.64 (m, 2H), 7.56 (dd, J = 8.0, 1.6 Hz, 1H), 7.22 (dd, J = 5.3, 3.5 Hz, 1H), 7.20 – 7.12 (m, 3H), 4.76 – 4.68 (m, 1H), 4.38 – 4.33 (m, 1H), 3.18 (dd, J = 14.7, 1.8 Hz, 1H), 2.98 – 2.91 (m, 2H), 2.91 – 2.82 (m, 2H); **^{13}C-NMR (167 MHz - $CDCl_3$):** δ 166.21, 146.67, 141.25, 137.33, 131.92, 130.95, 129.92, 127.64, 127.21, 126.26, 125.65, 125.28, 60.76, 42.43, 41.31, 36.16, 29.71; **FT-IR:** ṽ = 3053, 2913, 2159, 1683, 1493, 1363, 1277, 1166, 1050, 994, 893, 828 cm^{-1}; **LC-MS:** calc. for $[M+H]^+$ $C_{17}H_{14}ONBr$ = 330.1 found: 331.1.

2-chloro-7,8,13,13a-tetrahydro-5H-benzo[4,5]azepino[2,1-a]isoindol-5-one (4g)

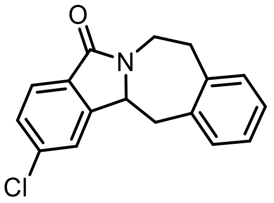

Yellow solid, 74% yield. **^{1}H-NMR (600 MHz - $CDCl_3$):** δ 7.74 (d, J = 8.1 Hz, 1H), 7.52 – 7.48 (m, 1H), 7.41 (dd, J = 8.1, 1.8 Hz, 1H), 7.27 – 7.10 (m, 4H), 4.73 (ddt, J = 11.1, 9.3, 4.6 Hz, 1H), 4.40 – 4.34 (m, 1H), 3.19 (dd, J = 14.6, 1.7 Hz, 1H), 3.00 – 2.80 (m, 4H); **^{13}C-NMR (151 MHz - $CDCl_3$):** δ 166.13,

146.46, 141.25, 137.94, 137.34, 130.47, 129.93, 129.07, 127.64, 127.20, 125.07, 122.70, 60.80, 42.44, 41.33, 36.18, 29.72, 14.08; **FT-IR:** ṽ = 3054, 2918, 2160, 1683, 1452, 1306, 1166, 1027, 258, 894, 834 cm^{-1}; **LC-MS:** calc. for $[M+H]^+$ $C_{17}H_{14}ONCl$ = 284.3 found: 284.3.

2-(trifluoromethyl)-7,8,13,13a-tetrahydro-5H-benzo[4,5]azepino[2,1-a]isoindol-5-one (4h)

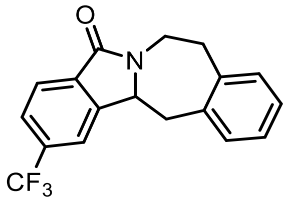

Yellow solid, 58% yield. **^{1}H-NMR (500 MHz - $CDCl_3$):** δ 7.93 (d, J = 7.8 Hz, 1H), 7.77 (s, 1H), 7.71 (dd, J = 7.9, 1.5 Hz, 1H), 7.44 (td, J = 7.0, 1.7 Hz, 1H), 7.26 (dd, J = 5.3, 3.6 Hz, 3H), 4.81 – 4.70 (m, 1H), 4.45 (dd, J = 11.0, 1.8 Hz, 1H), 3.26 (dd, J = 14.6, 1.8 Hz, 1H), 3.04 – 2.79 (m, 4H); **^{13}C-NMR (125 MHz - $CDCl_3$):** δ 165.81, 145.22, 141.15, 137.15, 135.17, 133.97, 133.85, 133.71, 133.45, 129.99, 129.96, 128.11, 127.74, 127.68, 127.30, 125.88, 125.86, 124.93, 124.47, 122.76, 119.48, 119.45, 119.41, 116.99, 61.18, 42.30, 41.47, 37.29, 36.06; **^{19}F-NMR (470 MHz - $CDCl_3$):** δ -62.20; **LC-MS:** calc. for $[M+H]^+$ $C_{18}H_{14}OF_3N$ = 318.1 found: 318.1.

2-iodo-7,8,13,13a-tetrahydro-5H-benzo[4,5]azepino[2,1-a]isoindol-5-one (4i)

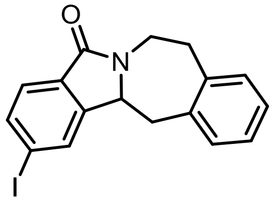

White solid, 68% yield. **^{1}H-NMR (600 MHz - $CDCl_3$):** δ 7.96 – 7.93 (m, 1H), 7.84 (dd, J = 8.0, 1.4 Hz, 1H), 7.61 (d, J = 7.9 Hz, 1H), 7.32 – 7.27 (m, 1H), 7.26 – 7.20 (m, 3H), 4.85 – 4.73 (m, 1H), 4.45 – 4.36 (m, 1H), 3.25 (dd, J = 14.7, 1.8 Hz, 1H), 3.06 – 2.97 (m, 2H), 2.97 – 2.88 (m, 2H); **^{13}C-NMR (151 MHz - $CDCl_3$):** δ 166.38, 146.71, 141.26, 137.75, 137.36, 131.56, 131.53, 129.92 (2C), 127.63, 127.20, 125.32, 98.37, 60.61, 42.41, 41.25, 36.16; **FT-IR:** ṽ = 2896, 2352, 2115, 1997, 1674 cm^{-1}; **LC-MS:** calc. for $[M+H]^+$ $C_{17}H_{14}ONI$ = 376.1 found: 376.1.

1,3-dichloro-7,8,13,13a-tetrahydro-5H-benzo[4,5]azepino[2,1-a]isoindol-5-one (4k)

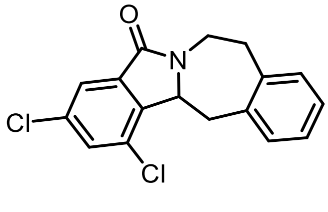

Yellow solid, 58% yield. **^{1}H-NMR (700 MHz - $CDCl_3$):** 7.69 (d, J = 1.7 Hz, 1H), 7.47 (d, J = 1.7 Hz, 1H), 7.26 – 7.21 (m, 1H), 7.20 – 7.12 (m, 3H), 4.76 – 4.68 (m, 1H), 4.41 (dd, J = 10.3, 1.7 Hz, 1H), 3.70

(dd, J = 14.5, 1.7 Hz, 1H), 3.01 – 2.94 (m, 2H), 2.91 – 2.83 (m, 1H), 2.66 (dd, J = 14.5, 10.3 Hz, 1H); **^{13}C-NMR (176 MHz - $CDCl_3$):** 164.71, 141.02, 140.05, 137.31, 135.70, 135.61, 131.96, 130.07, 129.80, 129.77, 127.69, 127.25, 122.80, 60.89, 53.43, 41.65, 39.47, 35.94, 29.71; **FT-IR:** ṽ = 3042, 2917, 1688, 1450, 1319, 1230, 1102, 959, 890 cm^{-1}; **LC-MS:** calc. for $[M+H]^+$ $C_{17}H_{13}OCl_2N$ = 318.2 found: 318.2.

3-(fluoro)-7,8,13,13a-tetrahydro-5H-benzo[4,5]azepino[2,1-a]isoindol-5-one (4l)

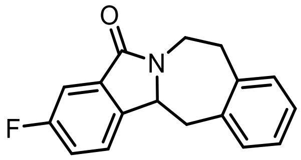

Off-white solid, 44% yield. **^{1}H-NMR (700 MHz - $CDCl_3$):** 7.69 (d, J = 7.4 Hz, 1H), 7.49 (td, J = 7.8, 4.5 Hz, 1H), 7.33 (dd, J = 5.3, 3.5 Hz, 1H), 7.26 – 7.23 (m, 3H), 7.24 – 7.20 (m, 1H), 4.82 (ddd, J = 9.9, 5.5, 1.5 Hz, 1H), 4.59 (dd, J = 10.9, 1.7 Hz, 1H), 3.55 (dd, J = 14.6, 1.7 Hz, 1H), 3.07 – 3.00 (m, 2H), 2.99 – 2.92 (m, 1H), 2.89 (dd, J = 14.6, 10.8 Hz, 1H); **^{13}C-NMR (176 MHz - $CDCl_3$):** 164.96 (d, J = 2.3 Hz), 156.69 (d, J = 250.6 Hz), 140.21, 136.57, 134.13 (d, J = 4.2 Hz), 129.63 (d, J = 6.7 Hz), 129.46 (d, J = 16.0 Hz), 129.20, 128.76, 126.54, 126.14, 118.76 (d, J = 3.7 Hz), 117.51 (d, J = 20.1 Hz), 58.20 (d, J = 2.6 Hz), 40.43, 39.73, 35.14; **^{19}F NMR (470 MHz, $CDCl_3$)** δ = -120.59 (dd, J = 9.3, 4.4 Hz); **HR-MS:** calc. for $[M+H]^+$ $C_{18}H_{15}OFN$ = 268.1132 found: 268.1134.

3-(chloro)-7,8,13,13a-tetrahydro-5H-benzo[4,5]azepino[2,1-a]isoindol-5-one (4m)

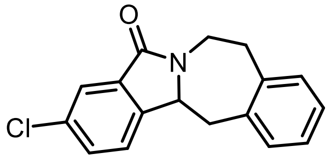

White solid, 36% yield. **^{1}H-NMR (500 MHz - $CDCl_3$):** 77.80 (dd, J = 7.4, 1.0 Hz, 1H), 7.54 (dd, J = 7.9, 1.0 Hz, 1H), 7.45 (dt, 1H), 7.34 (dd, J = 5.0, 3.7 Hz, 1H), 7.26 – 7.20 (m, 3H), 4.89 – 4.77 (m, 1H), 4.52 (dd, J = 10.3, 1.4 Hz, 1H), 3.84 (dd, J = 14.5, 1.5 Hz, 1H), 3.12 – 2.99 (m, 2H), 2.99 – 2.88 (m, 1H), 2.75 (dd, J = 14.4, 10.3 Hz, 1H); **^{13}C-NMR (126 MHz - $CDCl_3$):** 165.80, 142.99, 141.21, 137.36, 134.74, 133.77, 131.70, 129.90, 129.87, 127.59, 127.14, 123.98, 123.36, 60.88, 42.41, 41.33, 36.11; **FT-IR:** ṽ = 2918, 2159, 2027, 1977, 1675 cm^{-1}; **HR-MS:** calc. for $[M+H]^+$ $C_{17}H_{15}ONCl$ = 284.0836 found: 284.0839.

1-(chloro)-7,8,13,13a-tetrahydro-5H-benzo[4,5]azepino[2,1-a]isoindol-5-one (4m')

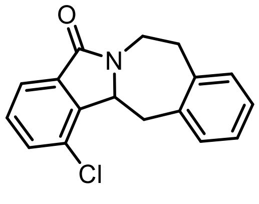

White solid, 36% yield. **^{1}H-NMR (500 MHz - $CDCl_3$):** 7.74 (dd, J = 7.4, 1.0 Hz, 1H), 7.48 (dd, J = 7.9, 1.0 Hz, 1H), 7.39 (t, J = 7.6 Hz, 1H), 7.28 (dd, J = 5.2, 3.6 Hz, 1H), 7.21 – 7.14 (m, 3H), 4.82 – 4.70 (m,

1H), 4.46 (dd, J = 10.4, 1.5 Hz, 1H), 3.77 (dd, J = 14.5, 1.6 Hz, 1H), 3.09 – 2.93 (m, 2H), 2.93 – 2.82 (m, 1H), 2.69 (dd, J = 14.4, 10.3 Hz, 1H); **^{13}C-NMR (126 MHz - $CDCl_3$):** 165.88, 141.71, 141.16, 137.69, 134.44, 132.18, 130.11, 130.06, 129.73, 129.10, 127.53, 127.15, 122.39, 61.07, 41.47, 39.53, 36.06; **FT-IR:** ṽ = 2929, 2159, 2028, 1976, 1686 cm^{-1}; **HR-MS:** calc. for $[M+H]^+$ $C_{17}H_{15}ONCl$ = 284.0836 found: 284.0840.

2,3-(dimethoxy)-7,8,13,13a-tetrahydro-5H-benzo[4,5]azepino[2,1-a]isoindol-5-one (4n)

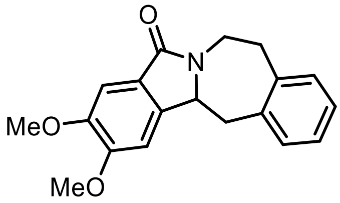

White solid, 27% yield. **^{1}H-NMR (700 MHz - $CDCl_3$):** 7.36 (s, 1H), 7.31 (m, 1H), 7.23 (m, 3H), 7.00 (s, 1H), 4.82 – 4.74 (m, 1H), 4.36 (dd, J = 11.0, 1.8 Hz, 1H), 4.02 (s, 3H), 3.96 (s, 3H), 3.25 (dd, J = 14.7, 1.8 Hz, 1H), 3.06 – 2.98 (m, 2H), 2.97 – 2.87 (m, 2H); **^{13}C-NMR (176 MHz - $CDCl_3$):** 167.39, 152.68, 149.96, 141.50, 138.40, 137.77, 129.91, 129.86, 127.46, 127.05, 124.22, 105.47, 104.36, 60.88, 56.38, 56.33, 42.75, 41.31, 36.38; **FT-IR:** ṽ = 2924, 2159, 2029, 1977, 1663, 890 cm^{-1}; **HR-MS:** calc. for $[M+H]^+$ $C_{19}H_{20}O_3N$ = 310.1437 found: 310.1441.

1,2-(dimethoxy)-7,8,13,13a-tetrahydro-5H-benzo[4,5]azepino[2,1-a]isoindol-5-one (4n')

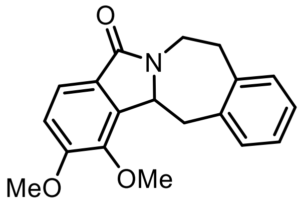

White solid, 39% yield. **^{1}H-NMR (700 MHz - $CDCl_3$):** 7.59 (d, J = 8.2 Hz, 1H), 7.33 – 7.30 (m, 1H), 7.25 – 7.19 (m, 3H), 7.05 (d, J = 8.2 Hz, 1H), 4.81 – 4.74 (m, 1H), 4.50 (dd, J = 10.6, 1.6 Hz, 1H), 4.01 (s, 3H), 3.96 (s, 3H), 3.64 (dd, J = 14.5, 1.7 Hz, 1H), 3.08 – 2.97 (m, 2H), 2.91 (dd, J = 13.3, 5.5 Hz, 1H), 2.80 (dd, J = 14.4, 10.6 Hz, 1H); **^{13}C-NMR (176 MHz - $CDCl_3$):** 166.66, 155.36, 143.93, 141.48, 138.40, 136.91, 130.14, 129.73, 127.34, 127.01, 125.61, 119.65, 113.03, 60.68, 59.84, 56.25, 41.37, 40.75, 36.40; **FT-IR:** ṽ = 2925, 2159, 2029, 1977, 1689 cm^{-1}; **HR-MS:** calc. for $[M+H]^+$ $C_{19}H_{20}O_3N$ = 310.1437 found: 310.1439.

11-methoxy-7,8,13,13a-tetrahydro-5H-benzo[4,5]azepino[2,1-a]isoindol-5-one (4o)

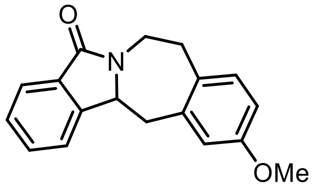

White solid, 50% yield. **^{1}H-NMR (600 MHz - CDCl$_3$):** 7.88 (d, J = 7.6, 1.2 Hz, 1H), 7.60 (td, J = 7.3, 1.2 Hz, 1H), 7.56 (dtd, J = 7.6, 1.1 Hz, 1H), 7.49 (t, J = 7.3 Hz, 1H), 7.13 (d, J = 8.2 Hz, 1H), 6.87 (d, J = 2.7 Hz, 1H), 6.77 (dd, J = 8.2, 2.7 Hz, 1H), 4.83 – 4.74 (m, 1H), 4.44 (d, 1H), 3.84 (s, 3H), 3.23 (dd, J = 14.6, 1.7 Hz, 1H), 3.04 – 2.83 (m, 4H); **^{13}C-NMR (151 MHz - CDCl$_3$):** 166.08, 157.44, 143.81, 137.98, 132.48, 130.94, 130.50, 129.82, 127.41, 122.77, 121.03, 115.14, 110.62, 60.14, 54.32, 41.75, 40.57, 34.32; **FT-IR:** ṽ = 2923, 2360, 2159, 2030, 1977, 1689 cm^{-1}; **HR-MS:** calc. for [M+H]$^+$ $C_{18}H_{18}O_2N$ = 280.1332 found: 280.1333.

11-(trifluoromethyl)-7,8,13,13a-tetrahydro-5H-benzo[4,5]azepino[2,1-a]isoindol-5-one (4p)

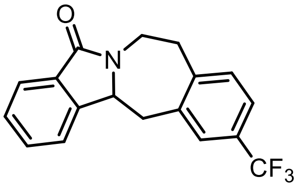

Yellow solid, 80% yield. **^{1}H-NMR (500 MHz - CDCl$_3$):** 7.83 (dt, J = 7.6, 1.0 Hz, 1H), 7.55 (td, J = 7.4, 1.2 Hz, 1H), 7.54 – 7.46 (m, 2H), 7.48 – 7.36 (m, 2H), 7.27 (d, J = 7.8 Hz, 1H), 4.83 – 4.73 (m, 1H), 4.39 (dd, J = 11.1, 1.7 Hz, 1H), 3.30 (dd, J = 14.8, 1.8 Hz, 1H), 3.04 – 2.85 (m, 4H); **^{13}C-NMR (126 MHz - CDCl$_3$):** 167.15, 145.41, 144.46, 138.58, 131.85, 131.74, 130.32, 129.51, 129.25, 128.70, 126.69, 126.66, 125.17, 124.39, 124.36, 123.98, 122.10, 60.72, 42.48, 40.76, 36.25; **^{19}F-NMR (470 MHz, CDCl$_3$):** δ = -62.34; **LC-MS:** calc. for [M+H]$^+$ $C_{18}H_{14}OF_3N$ = 318.1 found: 318.1.

5,6,12b,13-tetrahydro-8H-[1,3]dioxolo[4'',5'':4',5']benzo[1',2':4,5]azepino[2,1-a]isoindol-8-one (4q)

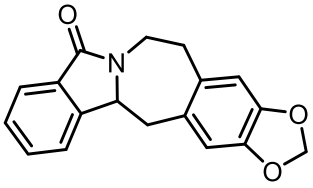

White solid, 31% yield. **^{1}H-NMR (500 MHz - CDCl$_3$):** 7.88 (d, J = 7.5, 1.0 Hz, 1H), 7.59 (td, J = 7.4, 1.2 Hz, 1H), 7.54 (dd, J = 7.5, 1.0 Hz, 1H), 7.49 (t, 1H), 6.80 (s, 1H), 6.72 (s, 1H), 5.96 (dd, 2H), 4.83 – 4.73 (m, 1H), 4.41 (d, 1H), 3.18 (dd, J = 14.8, 1.8 Hz, 1H), 3.04 – 2.90 (m, 2H), 2.89 – 2.80 (m, 2H); **^{13}C-NMR (126 MHz - CDCl$_3$):** 167.10, 146.43, 146.16, 144.73, 134.82, 131.98, 131.56, 130.90, 128.46, 123.81, 122.06, 110.42, 110.40, 101.11, 61.19, 42.19, 41.31, 35.96; **FT-IR:** ṽ = 2930, 2522, 2363, 2159, 2028, 1977, 1698, 1038 cm^{-1}; **LC-MS:** calc. for [M+H]$^+$ $C_{18}H_{15}O_3N$ = 294.0 found: 294.0.

11-methyl-7,8,13,13a-tetrahydro-5H-benzo[4,5]azepino[2,1-a]isoindol-5-one (4r)

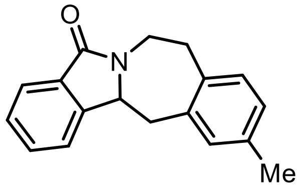

White solid, 39% yield. **^{1}H-NMR (600 MHz - CDCl$_3$):** 7.88 (d, J = 7.5, 1.0 Hz, 1H), 7.59 (td, J = 7.4, 1.1 Hz, 1H), 7.55 (d, J = 7.5, 1.1 Hz, 1H), 7.49 (t, J = 7.4, 0.8 Hz, 1H), 7.20 (d, J = 7.4 Hz, 1H), 7.05 (d, J = 7.9 Hz, 2H), 4.82 (d, J = 1.2 Hz, 1H), 4.42 (d, 1H), 3.27 (dd, J = 14.7, 1.8 Hz, 1H), 3.06 – 2.95 (m, 2H), 2.94 – 2.82 (m, 2H), 2.35 (s, 3H); **^{13}C-NMR (151 MHz - CDCl$_3$):** 167.11, 144.94, 141.26, 137.11, 134.74, 132.01, 131.52, 130.74, 129.87, 128.40, 127.55, 123.79, 122.08, 61.39, 42.15, 41.28, 36.29, 20.98; **FT-IR:** ṽ = 2920, 2159, 2029, 1977, 1680 cm^{-1}; **HR-MS:** calc. for $[M+H]^+$ $C_{18}H_{18}ON$ = 264.1382 found: 264.1385.

4. X-ray Structure Analyses

Data collection was conducted on a Bruker D8 Venture four-circle diffractometer by Bruker AXS GmbH using a PHOTON II CPAD detector by Bruker AXS GmbH. X-ray radiation was generated by microfocus sources IμS and IμS 3.0 Cu or Mo by Incoatec GmbH with HELIOS mirror optics and a single-hole collimator by Bruker AXS GmbH.

For the data collection, the programs APEX 3 Suite (v.2018.7-2) with the integrated programs SAINT (integration) and SADABS (adsorption correction) by Bruker AXS GmbH were used. Using Olex2, [7] the structures were solved with the ShelXT[8] structure solution program using Intrinsic Phasing and refined with the XL[9] refinement package using Least Squares minimization.

Crystal data and structure refinement for compound **3p** (CCDC 1990749) and **4g** (CCDC 1990750) with 50% ellipsoid probability level.

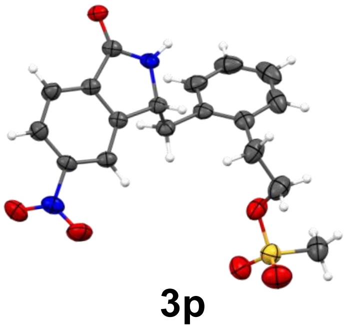

3p

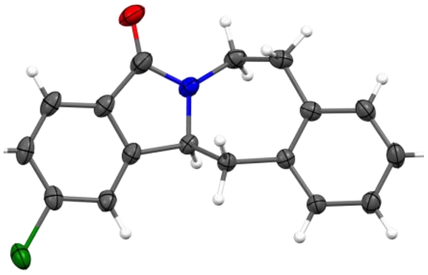

4g

**Compound**	**3q**	**4g***
Empirical formula	$C_{18}H_{18}N_2O_6S$	$C_{17}H_{14}ClNO$
Formula weight	390.40	283.74
Temperature/K	100.0	100.0
Crystal system	monoclinic	monoclinic
Space group	$P2_1/n$	$P2_1/c$
a/Å	14.8594(8)	12.3505(13)
b/Å	5.4431(3)	9.3353(9)
c/Å	21.6605(12)	12.2839(11)
α/°	90	90
β/°	90.003(2)	105.592(4)
γ/°	90	90
Volume/Å^3	1751.93(17)	1364.2(2)
Z	4	4

ρ_{calc}g/cm^3	1.480	1.382
μ/mm^{-1}	2.002	0.274
F(000)	816.0	592.0
Crystal size/mm^3	0.542 × 0.107 × 0.04	0.466 × 0.311 × 0.1
Radiation	CuKα (λ = 1.54178)	MoKα (λ = 0.71073)
2Θ range for data collection/°	7.214 to 159.562	5.548 to 59.998
Index ranges	-18 ≤ h ≤ 18, -6 ≤ k ≤ 6, -27 ≤ l ≤ 26	-17 ≤ h ≤ 17, -13 ≤ k ≤ 13, -17 ≤ l ≤ 16
Reflections collected	28346	29747
Independent reflections	3739 [R_{int} = 0.0493, R_{sigma} = 0.0288]	3982 [R_{int} = 0.0313, R_{sigma} = 0.0210]
Data/restraints/parameters	3739/0/245	3982/0/245
Goodness-of-fit on F^2	1.064	1.062
Final R indexes [I>=2σ (I)]	R_1 = 0.0714, wR_2 = 0.2227	R_1 = 0.0456, wR_2 = 0.1121
Final R indexes [all data]	R_1 = 0.0748, wR_2 = 0.2263	R_1 = 0.0531, wR_2 = 0.1175
Largest diff. peak/hole / e Å^{-3}	0.67/-0.81	0.39/-0.41

*Disorder omitted for clarity.

5. Biological data

Reagents

Purmorphamine was purchased from Cayman Chemical (#10009634) and DMSO from (Sigma Aldrich, #67685). The compound collection was synthesized as described in the paper. All other reagents used are mentioned in the respective protocols below, with the corresponding sources.

Cell lines

The murine mesenchymal stem cell line C3H10T1/2 (ATCC, CCL-226) was cultured in Dulbecco's Modified Eagle's medium (DMEM, high glucose, PAN, #P04-03550) supplemented with 10% of fetal calf serum (FCS, Fisher Scientific, #10136253, heat inactivated), 1 mM sodium pyruvate (PAN, #P04-43100) and 2 mM L-glutamine (PAN, #P0480100). The cells were cultured at 37°C and 5% CO_2 in a humidified atmosphere. Checks for mycoplasma contaminations were performed regularly and cells were always found to be free of any contaminations.

Osteoblast differentiation and viability assay

The osteoblast differentiation assay and the viability assay were performed using C3H10T1/2 cells. [10] For the screening and IC_{50} determinations 800 C3H10T1/2 cells per well were seeded in white 384-well plates. After incubation overnight, cells were treated with 1.5 µM purmorphamine and different concentrations of the compounds or DMSO as a control. After 96 h the cell culture medium was aspirated and the commercial luminogenic ALP substrate CDP-Star (Roche, #11685627001) was added. The cells were incubated for one hour at room temperature and in absence of light. Afterwards the luminescence signal was read. To identify and exclude toxic compounds, which would also lead to a reduced luminescent signal, cell viability measurements were conducted in parallel. For this purpose, C3H10T1/2 cells were seeded and treated as described above. The cellular ATP content was determined as a measure of cell viability using the Cell Titer Glo reagent (Promega). Compounds were considered as hit compounds if they caused at least 50% reduction in the luminescent signal in the osteoblast differentiation assay while retaining cell viability ≥ 80% at a concentration of 10 µM. To determine IC_{50} curves for hit compounds, three-fold dilution curves starting from 10 µM, were used. Calculations of the IC_{50} values were conducted, using GraphPad Prism 7. (GraphPad Software, USA).

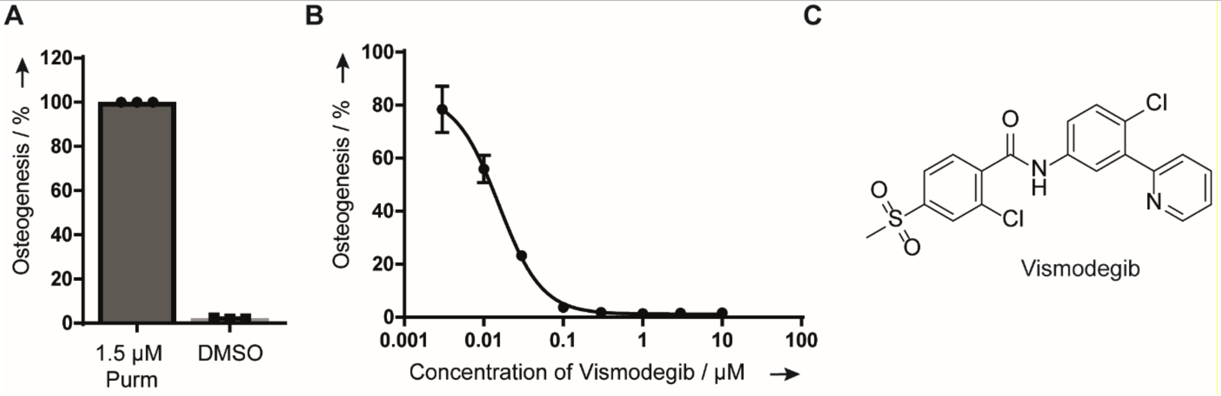

Figure 1: Hh-dependent osteoblast differentiation of C3H10T1/2 cells. A/B: C3H10T1/2 cells were treated with 1.5 µM purmorphamine or DMSO as a control (**A**) or with 1.5 µM purmorphamine and different concentrations of the known Hh inhibitor vismodegib (**B**). After 96 h, the activity of alkaline phosphatase was detected using a chemiluminescent substrate. The purmorphamine control was set to 100%. Data are mean values ± SD and representative of three biological replicates, each performed in three technical replicates. **C:** Chemical structure of the known Hh inhibitor Vismodegib.

6. DFT Calculations

Geometry optimizations and frequency analysis were carried out using Gaussian 16 Rev. B[11] employing tight convergence criteria using the M06-2X functional. [12] The 6-31G(2d,p) basis set was used for all elements except for Rh, for which LabL2DZ including pseudo potentials was used. The solvent environment was simulated by the IEFPCM of methanol as implemented in the software package.

Cartesian coordinates

*Structure II**

C	-1.61797100	-1.77769800	2.33470600
C	-1.33605000	-0.39123300	2.55196900
C	-0.49547900	-2.34232600	1.69196800
C	0.53203400	-1.33640400	1.59338900
C	0.01267600	-0.14723700	2.12719300
H	-0.41510200	-3.36029900	1.33658600
H	1.50230200	-1.46399400	1.13421800
H	0.50249300	0.81405300	2.12528400
C	-3.64462100	0.98435200	-0.25669700
C	-3.34696400	-0.29535900	0.21621700
C	-4.39413900	-1.13835900	0.57906000
C	-5.71232600	-0.68575200	0.49058300
C	-5.99825400	0.60042500	0.03712400
C	-4.95642900	1.43835700	-0.34334300
C	-2.49605600	1.83031800	-0.68999300
H	-4.20226200	-2.15053900	0.92107000
H	-6.52199000	-1.34883800	0.77848900
H	-7.02594500	0.94038900	-0.02464500
H	-5.13622100	2.44303900	-0.71334600
O	-2.57247300	3.00050000	-1.04649600
N	-1.37657500	1.07300600	-0.65143700
O	-0.21779000	1.76518400	-0.99856800

C	0.17444700	2.71389400	-0.10539300
O	-0.27918400	2.80518600	1.00163800
Rh	-1.36417200	-0.71092300	0.36405300
C	-0.58185000	-1.58293900	-1.67729100
C	-1.94192400	-1.76268200	-1.56782100
C	0.44731000	-2.62271300	-1.45141900
C	1.81196900	-2.26863800	-1.40513600
C	0.07953000	-3.96386700	-1.30256300
C	2.75098900	-3.26855000	-1.15369800
C	1.02922700	-4.94838800	-1.06542800
H	-0.96521600	-4.24539000	-1.37702200
C	2.37050300	-4.59597800	-0.97982300
H	3.80312400	-3.02066000	-1.07792300
H	0.72202300	-5.98164100	-0.95076000
H	3.12477700	-5.35036500	-0.78610600
H	-2.54305800	-2.28438700	2.56364300
H	-1.99660700	0.33185400	3.00719900
C	1.15810800	3.68706000	-0.73669600
C	2.07944700	3.01257200	-1.75570500
H	2.70785700	2.27013000	-1.25966100
H	1.51389100	2.52656000	-2.55332200
H	2.73051400	3.76950700	-2.20356900
C	1.98084600	4.35557000	0.36404900
H	2.61190400	5.13344000	-0.07602800
H	1.32875900	4.81456000	1.11003100
H	2.62212200	3.62375100	0.86155900
C	0.27396100	4.73234300	-1.44429300
H	-0.37129800	4.25656600	-2.18587500
H	-0.36364700	5.24935200	-0.72188800
H	0.91359000	5.46946800	-1.93891300
C	2.22334500	-0.82585300	-1.62372200
H	1.59649400	-0.15378300	-1.02502600
H	2.04989000	-0.55091400	-2.67275800

C	3.67337700	-0.51493600	-1.31475500
H	4.37030000	-1.17261000	-1.83459000
H	3.92308400	0.51195300	-1.59219800
O	3.94659500	-0.70928200	0.09810500
S	3.84256300	0.61796100	0.97986200
O	4.87987700	1.52817900	0.58179500
O	2.48304600	1.09726800	0.94097300
C	4.19265100	-0.09990000	2.54760600
H	4.17441000	0.71993700	3.26591700
H	3.41384900	-0.82865100	2.76948500
H	5.17847400	-0.55936800	2.50202900
H	-0.24895600	-0.66563100	-2.15265000
H	-2.38254400	-2.70792500	-1.27024300
H	-2.60500500	-1.04920500	-2.03922600

*Structure III**

C	1.71934000	-0.54507800	2.85494600
C	0.37000800	-0.80278700	2.70086900
C	1.98740200	0.80936900	2.39018500
C	0.76037700	1.40246000	2.04009100
C	-0.23804100	0.37483500	2.09863600
H	2.95305300	1.29464400	2.38453600
H	0.62098600	2.40908700	1.67157700
H	-1.29030300	0.49220100	1.87289400
C	2.45335700	-2.12105900	-0.82902500
C	2.97773000	-0.84619900	-1.14325700
C	4.31316000	-0.58642000	-0.78441400
C	5.08947100	-1.52964500	-0.13233600
C	4.55862200	-2.78734800	0.16241500
C	3.25412500	-3.07935600	-0.19546800
C	1.02246500	-2.52832100	-1.10856600
H	4.75583500	0.36323700	-1.06908800

H	6.11681800	-1.29760100	0.12477300
H	5.16818900	-3.53480700	0.65753900
H	2.82334700	-4.05524200	0.00001300
O	0.71691000	-3.64051300	-1.52256400
N	0.21052000	-1.52210100	-0.75768100
O	-1.14481700	-1.84388200	-0.84823600
C	-1.57244500	-2.75435200	0.05963800
O	-0.90778400	-3.12953300	0.98689300
Rh	1.15650300	-0.18284500	0.56987300
C	1.17846500	0.99122100	-1.13178500
H	0.19116600	0.89244300	-1.57614400
C	2.20469400	0.19370000	-1.96097800
H	1.68159700	-0.32527500	-2.76724800
H	2.93310300	0.86932400	-2.41839500
C	1.52281500	2.42010100	-0.86298500
C	0.58519100	3.45932100	-1.03207700
C	2.80695500	2.75815100	-0.41112300
C	0.95300000	4.77222800	-0.72488100
C	3.16145800	4.06563500	-0.11364600
H	3.53383000	1.96574500	-0.26363900
C	2.22609500	5.08525500	-0.26704500
H	0.21997200	5.56298000	-0.85618900
H	4.16263600	4.28742500	0.23961800
H	2.48597000	6.11246900	-0.03755700
H	2.47315300	-1.25011800	3.17700800
H	-0.13156600	-1.74497000	2.85938600
C	-2.95868200	-3.27145800	-0.29422800
C	-3.86620700	-2.15307400	-0.81649700
H	-3.98739100	-1.36683800	-0.06653000
H	-3.45929400	-1.70676700	-1.72699200
H	-4.85130700	-2.57027000	-1.04700300
C	-3.57190600	-3.92066200	0.94581200
H	-4.54581900	-4.34768200	0.69034400

H	-2.92888500	-4.71619400	1.32744400
H	-3.71594000	-3.18479200	1.74225900
C	-2.74077300	-4.32699600	-1.39310500
H	-2.26553500	-3.88022200	-2.26934400
H	-2.10238300	-5.13774100	-1.03158900
H	-3.70695900	-4.74749200	-1.68750900
C	-0.83079000	3.20476400	-1.50003500
H	-0.85950400	2.51434200	-2.34787100
H	-1.28654100	4.14325600	-1.82577800
C	-1.65020200	2.62707900	-0.36053600
H	-1.22735400	1.68075500	-0.01340800
H	-1.70553200	3.33476900	0.47419500
O	-2.98290900	2.36951400	-0.85455500
S	-4.00673000	1.86644000	0.25900100
O	-3.41154000	0.75412000	0.95766900
O	-4.44881200	2.99036400	1.03565300
C	-5.28790900	1.31218600	-0.81203900
H	-6.08121700	0.92875500	-0.17007100
H	-5.63446500	2.16544100	-1.39311700
H	-4.88332200	0.52696300	-1.44892900

Structure A’

C	-2.84676300	2.79123800	1.00644200
C	-1.49340400	3.12972900	1.40579800
C	-3.36743800	1.88283700	1.93098100
C	-2.28503200	1.52260700	2.80939000
C	-1.17466500	2.40512300	2.54957000
H	-4.35569200	1.44646000	1.92789100
H	-2.35046600	0.82321200	3.63205600
H	-0.23163800	2.40810400	3.07823100
C	-1.95294900	-1.49558700	-2.26280000

C	-1.31357700	-2.59360600	-1.68216500
C	-1.95243600	-3.82956000	-1.70240600
C	-3.21032000	-3.95531600	-2.28456900
C	-3.83878500	-2.85385800	-2.86531900
C	-3.20220700	-1.62003200	-2.86676900
C	-1.23905700	-0.19723100	-2.30631200
H	-1.46690300	-4.69141800	-1.25647800
H	-3.70521700	-4.92024400	-2.28618200
H	-4.81764700	-2.96131600	-3.31762700
H	-3.65963200	-0.74902100	-3.32315800
O	-1.29095600	0.57093100	-3.23011800
N	-0.47140500	0.09761400	-1.13938800
O	0.56998900	0.99242700	-1.44729000
C	0.23189700	2.31092000	-1.52179100
O	-0.89188000	2.71363300	-1.48033300
Rh	-1.61271500	0.76428500	0.87788200
C	0.14147000	-1.03030800	-0.34991300
H	1.18988800	-0.74689000	-0.28183700
C	0.05274300	-2.36997300	-1.09232300
H	0.80261200	-2.38442100	-1.89290900
H	0.30632300	-3.17226300	-0.39367800
C	-0.48580100	-1.04078400	1.03624200
C	0.35435900	-1.07758800	2.21608600
C	-1.86921500	-1.39666900	1.16824300
C	-0.18239100	-1.45130700	3.41741300
C	-2.36565300	-1.80745400	2.44948700
H	-2.44654400	-1.70075000	0.30410700
C	-1.54800500	-1.83575100	3.53967900
H	0.45138400	-1.45317800	4.29962000
H	-3.39968800	-2.12855300	2.52672700
H	-1.92608400	-2.15714300	4.50419200
H	-3.36219400	3.20904500	0.15323500
H	-0.85270100	3.82291800	0.88016400

C	1.49841800	3.14614200	-1.61538900
C	2.25570300	2.97678900	-0.28647100
H	1.61281700	3.21409500	0.56833900
H	2.62251700	1.95273000	-0.17962100
H	3.11125500	3.65836100	-0.27103200
C	1.09696200	4.60461200	-1.82539800
H	1.99738200	5.22069700	-1.89558900
H	0.51962700	4.72428000	-2.74575900
H	0.49022900	4.97083900	-0.99319600
C	2.37136000	2.65125400	-2.77726700
H	2.69826800	1.62293000	-2.61024300
H	1.83033900	2.70125600	-3.72681400
H	3.25702200	3.28862300	-2.85380300
C	1.81070800	-0.68923500	2.12770800
H	2.16068700	-0.34456100	3.10449900
H	1.95198600	0.13497400	1.42031600
C	2.67487000	-1.86823400	1.69876400
H	2.69151400	-2.64292500	2.46674700
H	2.31693300	-2.31775400	0.76707600
O	4.05131500	-1.45709200	1.54164600
S	4.52815800	-1.03042500	0.08467500
O	3.42537900	-0.41501200	-0.60973300
O	5.74066000	-0.29955900	0.29218400
C	4.88596000	-2.58279500	-0.67403800
H	5.23655800	-2.36601500	-1.68313200
H	5.65967600	-3.07364300	-0.08600800
H	3.97229800	-3.17617800	-0.70796900

*Structure IV**

C	-0.20029300	0.81729700	2.81912600
C	0.99303300	0.65651500	2.02572900
C	-0.50910300	-0.44818700	3.40010100

C	0.40202800	-1.39749800	2.86950800
C	1.36005800	-0.69091300	2.05784700
H	-1.33168700	-0.65744000	4.06770900
H	0.41769900	-2.45477600	3.09041000
H	2.17434200	-1.13967900	1.50479900
C	-3.44017100	-0.60202400	-1.09059500
C	-3.06053200	-1.85444600	-0.56613800
C	-3.98462500	-2.90399800	-0.60032200
C	-5.24796300	-2.74934100	-1.15649900
C	-5.61231200	-1.52176600	-1.69462500
C	-4.70974300	-0.46724400	-1.65523600
C	-2.62919700	0.67982600	-1.09880900
H	-3.69521300	-3.86626300	-0.18952400
H	-5.93850000	-3.58525000	-1.16781800
H	-6.59325200	-1.38117800	-2.13418400
H	-4.97048500	0.50264600	-2.06040700
O	-2.96092700	1.61226700	-1.82977000
N	-1.58506200	0.72553200	-0.25831100
O	-0.88425500	1.93251900	-0.35955100
C	-1.44565000	2.97561500	0.29687000
O	-2.41466800	2.87730900	0.99667300
Rh	-0.84472700	-0.53979900	1.18062700
C	-0.52331600	-1.88203000	-0.61000300
C	-1.72883200	-2.20931900	0.00079500
C	0.75675200	-2.60679000	-0.46072700
C	1.88140400	-2.20066000	-1.20472400
C	0.88152700	-3.68441000	0.42634600
C	3.10033200	-2.84854900	-0.99217600
C	2.09820700	-4.31956800	0.62185000
H	0.01717300	-4.02416500	0.98686400
C	3.22050900	-3.89200500	-0.08295400
H	3.96800100	-2.51659600	-1.55457500
H	2.16964800	-5.14512100	1.32094200

H	4.17936200	-4.37562100	0.06572700
H	-0.73189100	1.74250200	2.98971900
H	1.47616800	1.43013200	1.44676800
C	-0.67244100	4.25439000	0.00921200
C	0.81612200	4.04588200	0.32274100
H	0.96795900	3.81798300	1.38304300
H	1.23677000	3.23364300	-0.27666100
H	1.36641100	4.96335900	0.09381900
C	-1.24496800	5.37642400	0.87258400
H	-0.70336500	6.30483700	0.67104400
H	-2.30362700	5.53464800	0.65450700
H	-1.14835300	5.14065600	1.93575000
C	-0.85148500	4.58028100	-1.48225600
H	-0.42822000	3.79039900	-2.10648300
H	-1.91051500	4.68741800	-1.73290300
H	-0.34338700	5.52174500	-1.71063200
H	-2.29976700	-0.71321700	1.54903400
H	-0.58231600	-1.19481900	-1.44644600
H	-1.76099800	-3.06909500	0.66310900
C	1.85268400	-1.06994300	-2.20959400
H	0.97226100	-1.12446700	-2.85792400
H	2.72709200	-1.16352800	-2.85478000
C	1.86254100	0.31947500	-1.57346300
H	0.90243000	0.57435500	-1.12069200
H	2.10678700	1.08380700	-2.31753100
O	2.78754800	0.39758600	-0.46660100
S	4.33652400	0.63206100	-0.75221000
C	4.43743100	2.39232100	-0.83356500
H	3.82489000	2.74007000	-1.66548900
H	4.08799300	2.79282200	0.11737200
H	5.48516800	2.64154800	-1.00245000
O	4.99413100	0.16665900	0.43193500
O	4.67481900	0.07764500	-2.03430500

*Structure V**

C	1.04187800	0.12986600	3.02642200
C	-0.15174400	-0.69640100	2.85191000
C	0.67049800	1.49340000	2.79380200
C	-0.63619800	1.47082700	2.27588200
C	-1.16593800	0.11633500	2.38313000
H	1.31717800	2.35525800	2.86400300
H	-1.17662000	2.32145100	1.87964800
H	-2.14808700	-0.18578600	2.04565100
C	3.37434900	-0.69896800	-1.00659000
C	3.39481700	0.58849300	-0.44241200
C	4.62899300	1.24719400	-0.36490900
C	5.80226700	0.66899600	-0.83178200
C	5.76683500	-0.59926600	-1.40347300
C	4.55602200	-1.27082300	-1.48458300
C	2.15871700	-1.57943300	-1.10299100
H	4.65835900	2.24352800	0.06759600
H	6.73971000	1.20880900	-0.75056900
H	6.67424800	-1.06233400	-1.77442900
H	4.49498500	-2.26345700	-1.91445200
O	2.18384500	-2.62563300	-1.74114300
N	1.07678900	-1.14452800	-0.41210700
O	0.01002000	-2.05355400	-0.50992100
C	0.05943800	-3.09121700	0.35083400
O	0.95972800	-3.27731700	1.12398100
Rh	0.69109200	0.30807600	0.95534900
C	1.49030800	2.07010300	-1.16730700
C	2.18339300	1.34831600	0.01868400
C	0.33517400	2.94256800	-0.71185500
C	-0.97932900	2.79760200	-1.18395700
C	0.60010200	3.92606500	0.24912600

C	-1.98975400	3.58936800	-0.62269700
C	-0.40584000	4.71921600	0.78357100
H	1.62397000	4.07127600	0.58285200
C	-1.71875300	4.53466000	0.35810400
H	-3.00526900	3.46042900	-0.98291700
H	-0.16728600	5.47291900	1.52609200
H	-2.52143400	5.13653500	0.76972200
H	1.99442400	-0.21851800	3.39890500
H	-0.16968000	-1.77161900	2.95724200
C	-1.21999700	-3.91013700	0.25800500
C	-2.29964600	-3.10115900	0.99866900
H	-2.06904100	-3.01661500	2.06564500
H	-2.38046600	-2.09268600	0.58071700
H	-3.26639500	-3.60649800	0.90473900
C	-1.00079400	-5.25650500	0.94429700
H	-1.93350500	-5.82737000	0.93601900
H	-0.23399700	-5.83903900	0.42615100
H	-0.68313900	-5.11771400	1.97979800
C	-1.62983900	-4.10714100	-1.20626000
H	-1.83801800	-3.15065100	-1.69140200
H	-0.83953600	-4.61042400	-1.77080400
H	-2.53135700	-4.72566200	-1.24933800
H	2.52482000	2.10894700	0.72512100
H	1.16788100	1.33167800	-1.90684900
H	2.24814400	2.69559500	-1.66139000
C	-1.36447500	1.89031100	-2.33625700
H	-0.60988800	1.93671400	-3.12917100
H	-2.29446800	2.26887500	-2.76345300
C	-1.54499100	0.40986300	-2.01015200
H	-0.59158300	-0.09332300	-1.84909800
H	-2.06144200	-0.09732200	-2.83085600
O	-2.25205900	0.17297000	-0.77087500
S	-3.83637900	0.27833500	-0.73512400

C	-4.37835000	-1.20149200	-1.53349300
H	-4.01889600	-1.20764400	-2.56194400
H	-4.00717600	-2.05686600	-0.97159200
H	-5.46814000	-1.16974000	-1.51776000
O	-4.15061700	0.20844800	0.66355800
O	-4.26913800	1.40162800	-1.51936900

7. References

[1] S. Samanta, T. L. Lim, Y. Lam. *Chem. Med. Chem*. **2013**, 8, 994.

[2] Y. Hu, Y. Xie, Z. Shen, H. Huang, *Angew. Chem. Int. Ed.* **2017**, *56*, 2473

[3] N. Guimond, S. I. Gorelsky, K. Fagnou. *J. Am. Chem. Soc.* **2011** *133* 6449.

[4] a) B. Ye, N. Cramer. *Science*. **2012**, 338, 504; b) E. A. Trifonova, N. M. Ankudinov, A. A. Mikhaylov, D. A. Chusov, Y. V. Nelyubina, D. S. Perekalin, *Angew. Chem. Int. Ed.* **2018**, *57*, 7714.

[5] Y. Kita, T. Yata, Y. Nishimoto, M. Yasuda**,** *J. Org. Chem.* **2018,** *83*, 740

[6] a) A. Saxena, F. Perez, M. J. Krische, *J. Am. Chem. Soc.* **2015**, *137*, 5883; b) E. S. Sherman, P. Fuller, D. Kasi, S. R. Chemler, *J. Org. Chem.* **2007**, *72*, 3896.

[7] O. V. Dolomanov, L.J. Bourhis, R.J. Gildea, J.A.K. Howard, H. Puschmann, OLEX2: A complete structure solution, refinement and analysis program, *J. Appl. Crystallogr.* **2009**, *42*, 339.

[8] G.M. Sheldrick, SHELXT - Integrated space-group and crystal-structure determination, *Acta Crystallogr. Sect. A Found. Crystallogr.* **2015**, *71*, 3.

[9] G.M. Sheldrick, A short history of SHELX, *Acta Crystallogr. Sect. A Found. Crystallogr.* **2008**, *64*, 112.

[10] X. Wu, S. Ding, Q. Ding, N. S. Gray, P. G. Schultz. *J. Am. Chem. Soc.* **2002**, *124*, 14520.

[11] M. J. Frisch, G. W, Trucks, H. B. Schlegel, G. E. Scuseria, M. A. Robb, J. R. Cheeseman, G. Scalmani, V. Barone, G. A. Petersson, H. Nakatsuji, X. Li, M. Caricato, A. V. Marenich, J. Bloino, B. G. Janesko, R. Gomperts, B. Mennucci, H. P. Hratchian, J. V. Ortiz, A. F. Izmaylov, J. L. Sonnenberg, D. Williams-Young, F. Ding, F. Lipparini, F. Egidi, J. Goings, B. Peng, A. Petrone, T. Henderson, D. Ranasinghe, V. G. Zakrzewski, J. Gao, N. Rega, G. Zheng, W. Liang, M. Hada, M. Ehara, K. Toyota, R. Fukuda, J. Hasegawa, M. Ishida, T. Nakajima, Y. Honda, O. Kitao, H. Nakai, T. Vreven, K. Throssell, J. A. Montgomery Jr., J. E. Peralta, F. Ogliaro, M. J. Bearpark, J. J. Heyd, E. N. Brothers, K. N. Kudin, V. N. Staroverov, T. A. Keith, R. Kobayashi, J. Normand, K. Raghavachari, A. P. Rendell, J. C.; Burant, S. S. Iyengar, J. Tomasi, M. Cossi, J. M. Millam, M. Klene, C. Adamo, R. Cammi, J. W. Ochterski, R. L. Martin, K. Morokuma, O. Farkas, J. B. Foresman, D. J. Fox, D. J. Gaussian, *16 Rev. B.01, Wallingford, CT*, **2016**.

[12] Y. Zhao, D. G. Truhlar, The M06 suite of density functionals for main group thermochemistry, thermochemical kinetics, noncovalent interactions, excited states, and transition elements: two new functionals and systematic testing of four M06-class functionals and 12 other functionals. *Theor. Chem. Acc.* **2008**, *120*, 215.

8. NMR Spectra

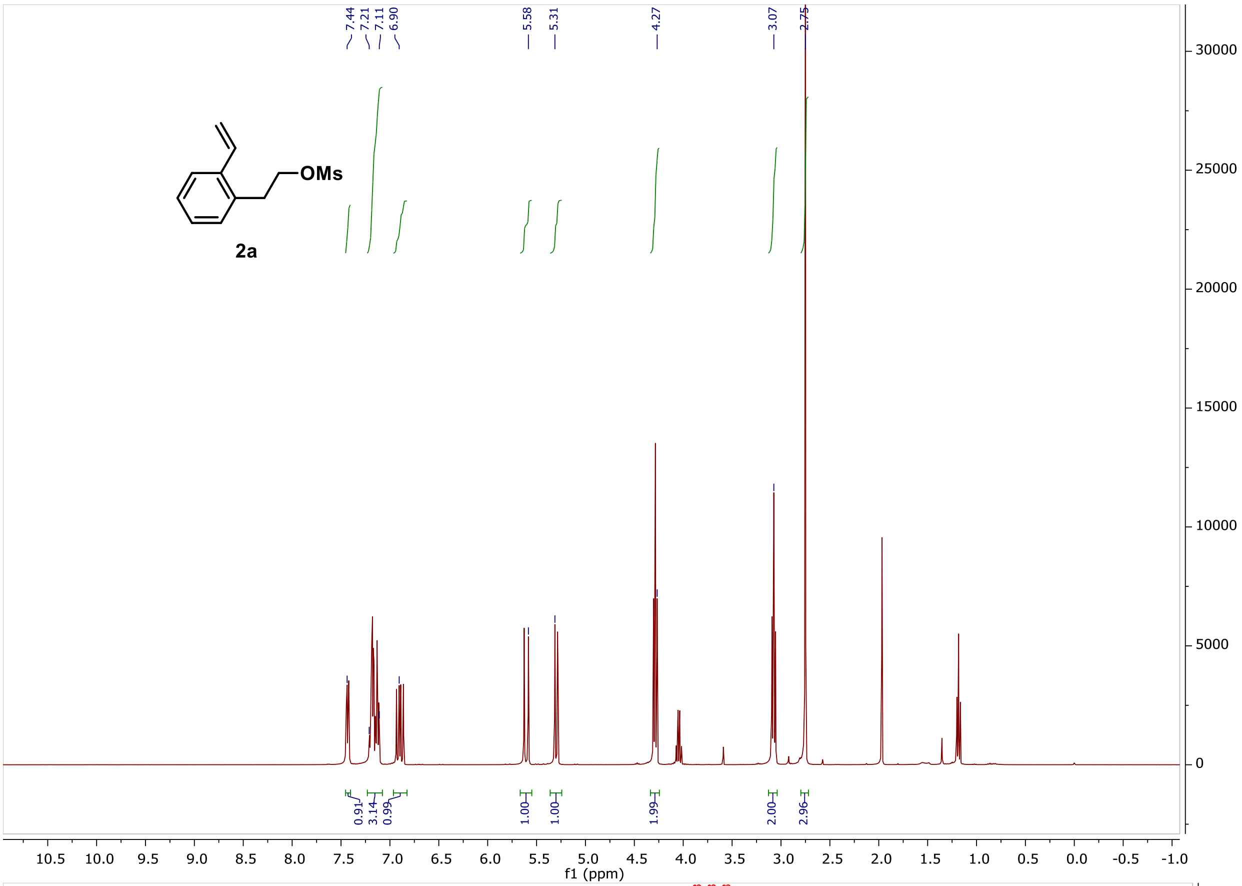

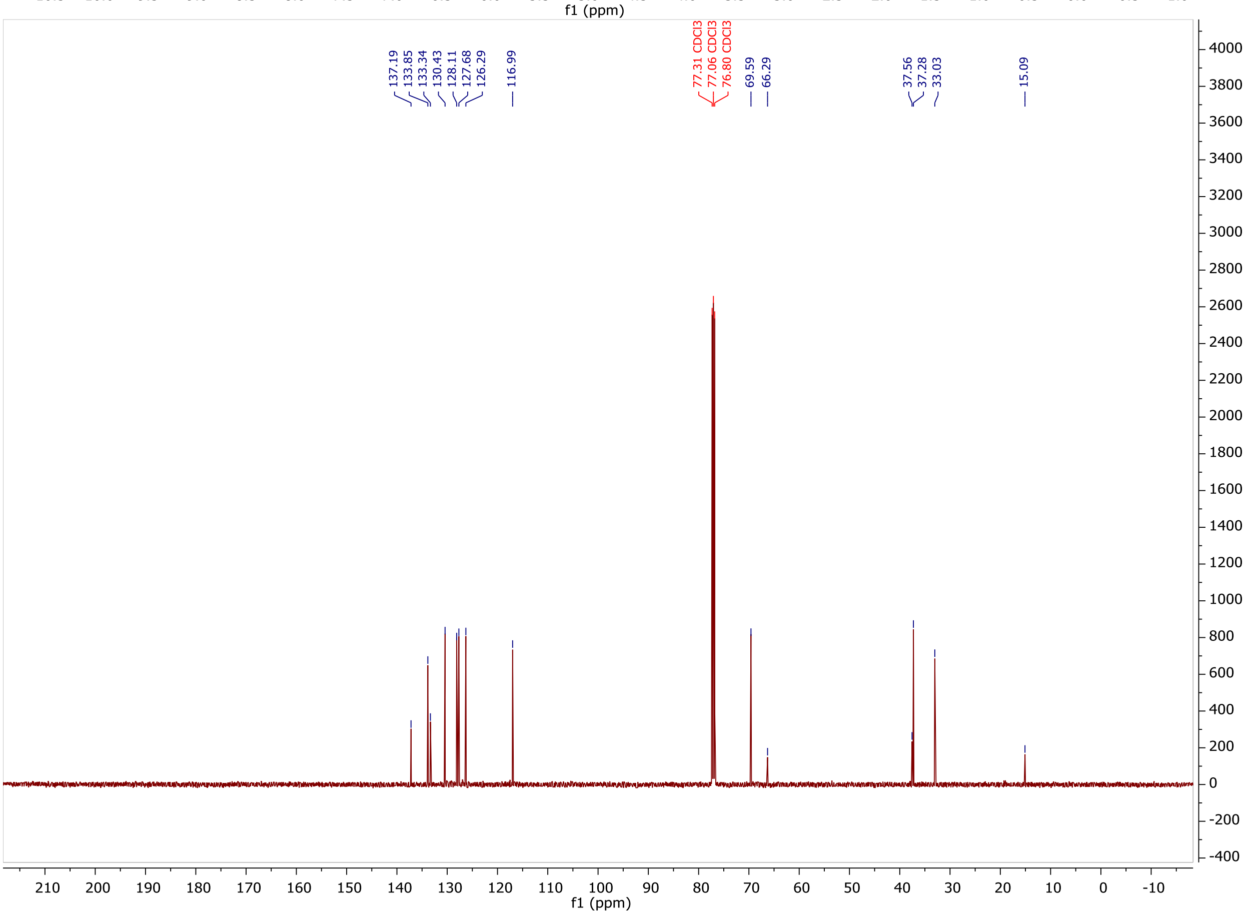

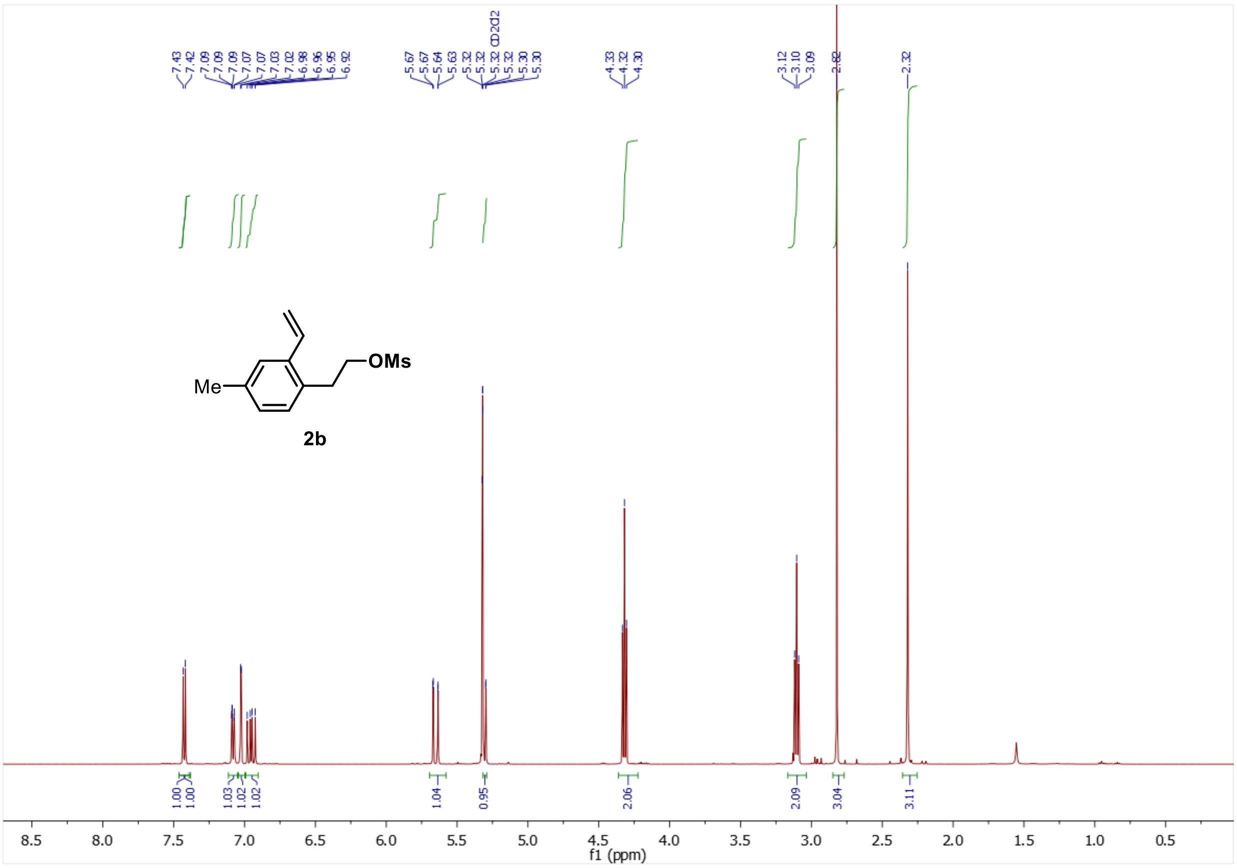
7.43
7.42
7.09
7.09
7.09
7.07
7.07
7.03
7.02
6.98
6.96
6.95
6.92
5.67
5.67
5.64
5.63
5.32
5.32
CD2Cl2
5.32
5.32
5.30
5.30
4.33
4.32
4.30
3.12
3.10
3.09
2.82
2.32
Me
OMs
2b
1.00
1.00
1.03
1.02
1.02
1.04
0.95
2.06
2.09
3.04
3.11
8.5
8.0
7.5
7.0
6.5
6.0
5.5
5.0
4.5
4.0
3.5
3.0
2.5
2.0
1.5
1.0
0.5
f1 (ppm)

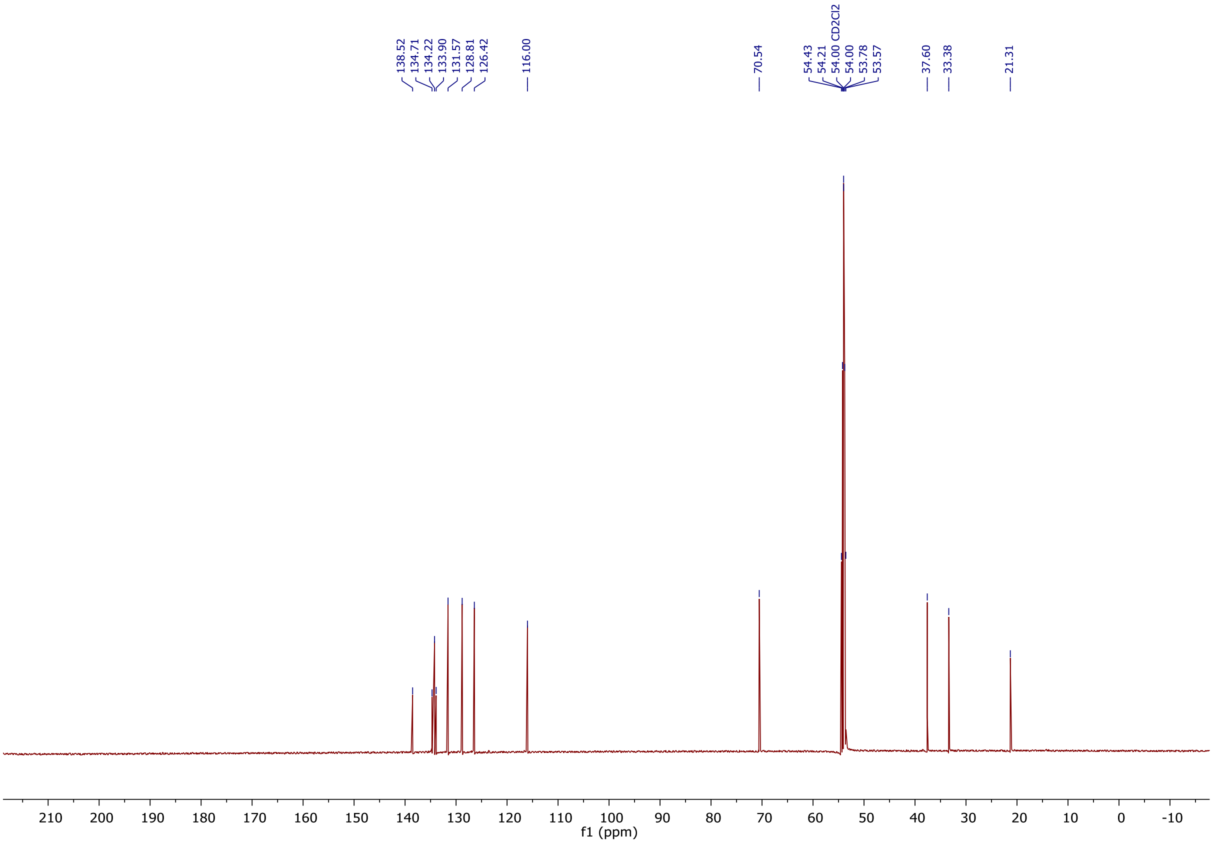
138.52
134.71
134.22
133.90
131.57
128.81
126.42
116.00
70.54
54.43
54.21
54.00 CD2Cl2
54.00
53.78
53.57
37.60
33.38
21.31
210
200
190
180
170
160
150
140
130
120
110
100
90
80
70
60
50
40
30
20
10
0
-10
f1 (ppm)

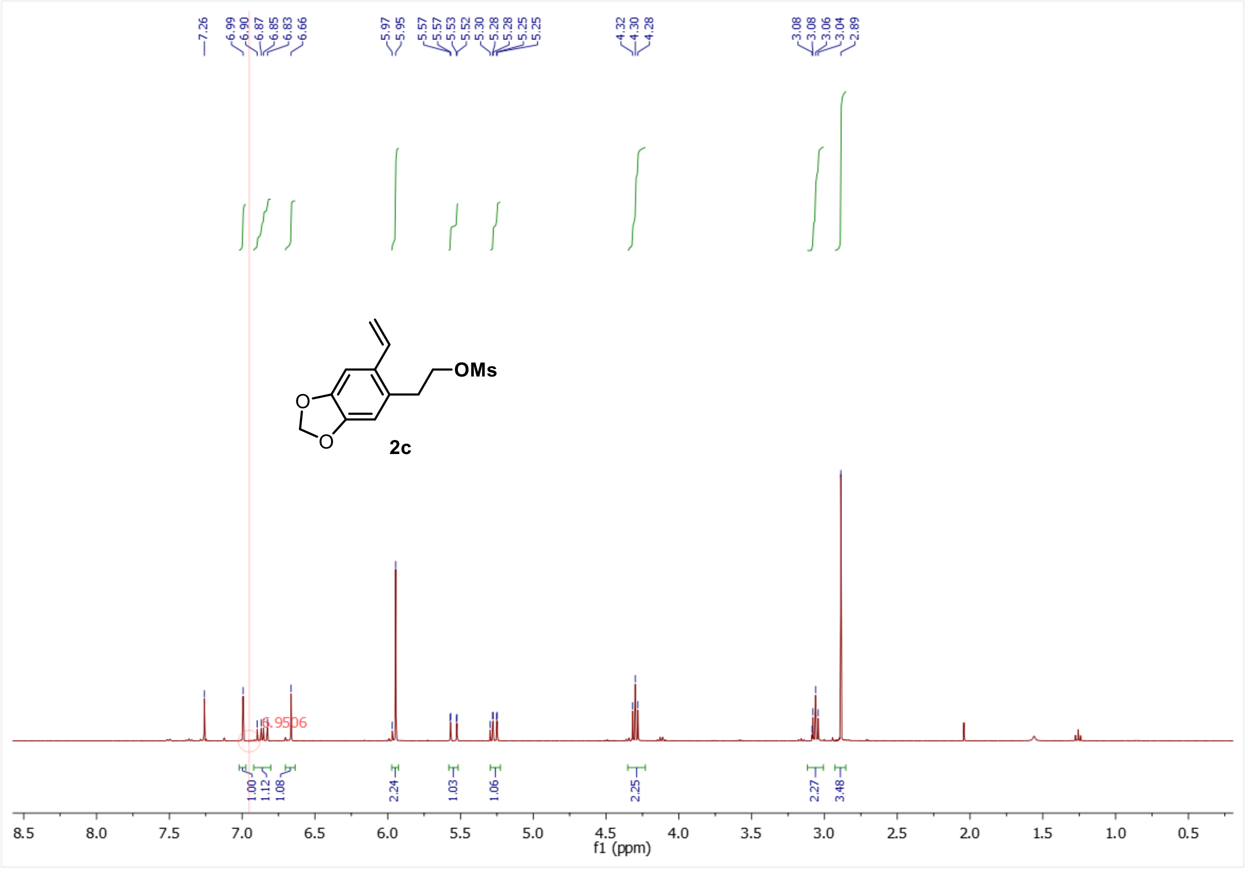
7.26
6.99
6.90
6.87
6.85
6.83
6.66
5.97
5.95
5.57
5.57
5.53
5.52
5.30
5.28
5.28
5.25
5.25
4.32
4.30
4.28
3.08
3.08
3.06
3.04
2.89
OMs
O
O
2c
1.00
1.12
1.08
2.24
1.03
1.06
2.25
2.27
3.48
f1 (ppm)

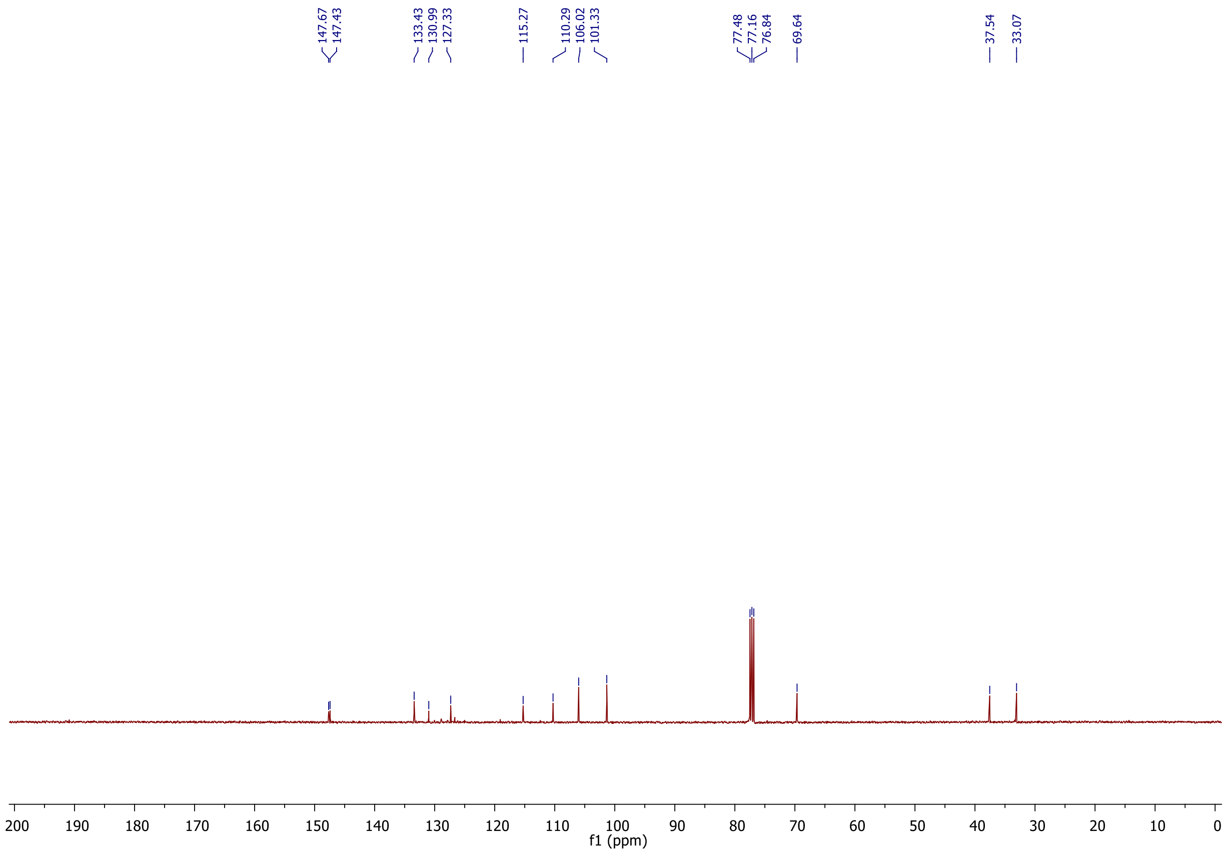
147.67
147.43
133.43
130.99
127.33
115.27
110.29
106.02
101.33
77.48
77.16
76.84
69.64
37.54
33.07
f1 (ppm)

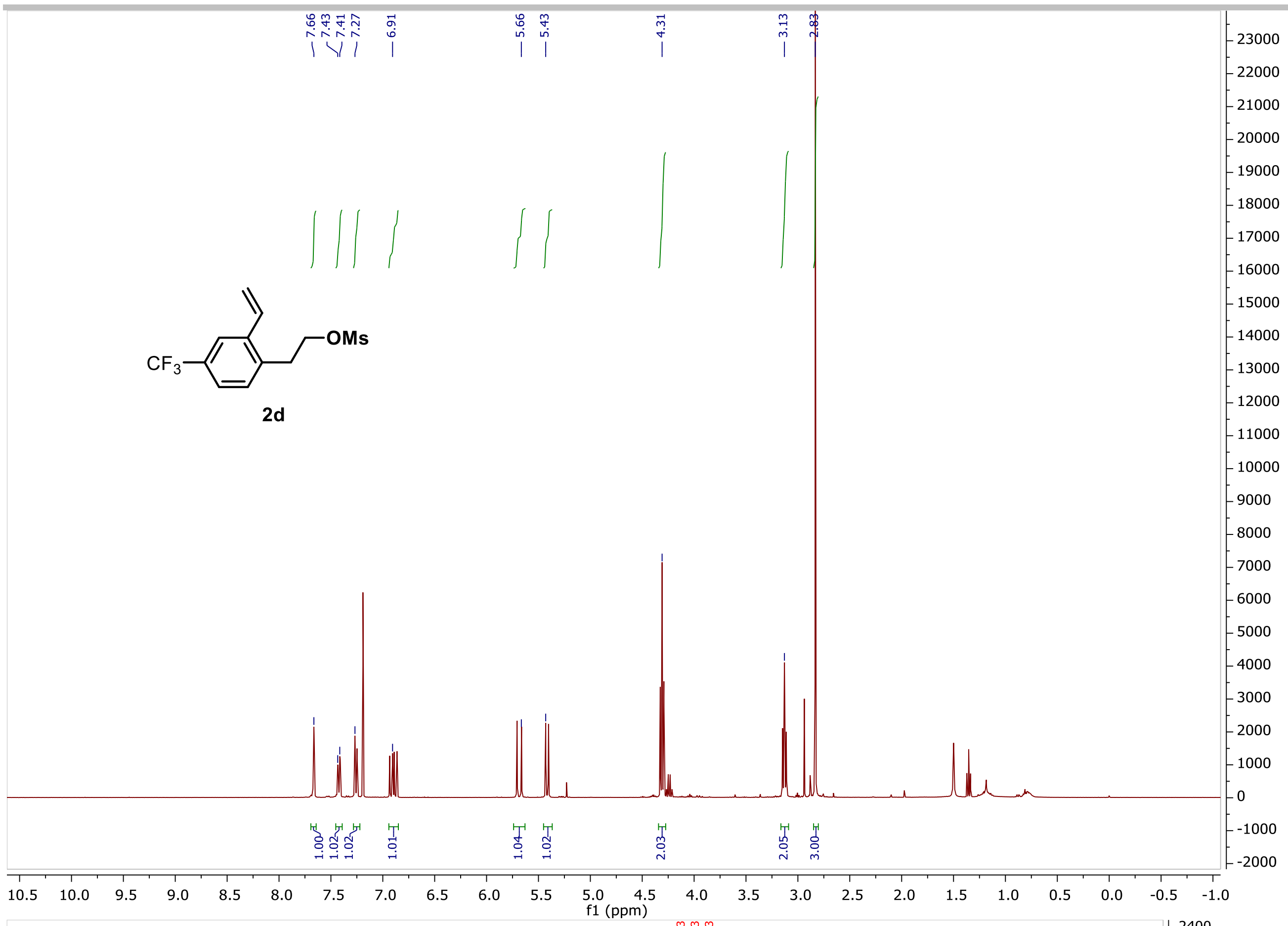
7.66
7.43
7.41
7.27
6.91
5.66
5.43
4.31
3.13
2.83
CF3
OMs
2d
1.00
1.02
1.02
1.01
1.04
1.02
2.03
2.05
3.00
f1 (ppm)

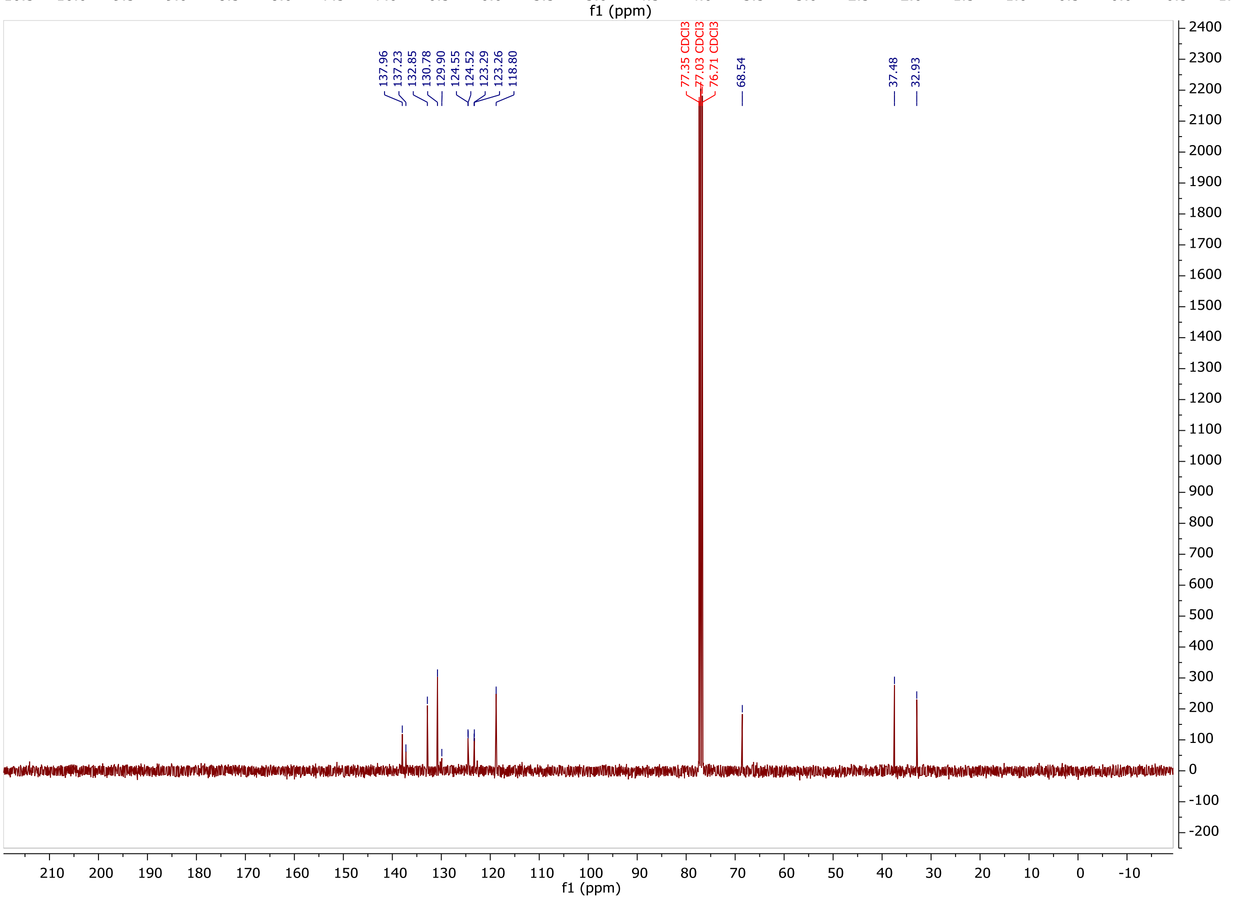
137.96
137.23
132.85
130.78
129.90
124.55
124.52
123.29
123.26
118.80
77.35 CDCl3
77.03 CDCl3
76.71 CDCl3
68.54
37.48
32.93
f1 (ppm)

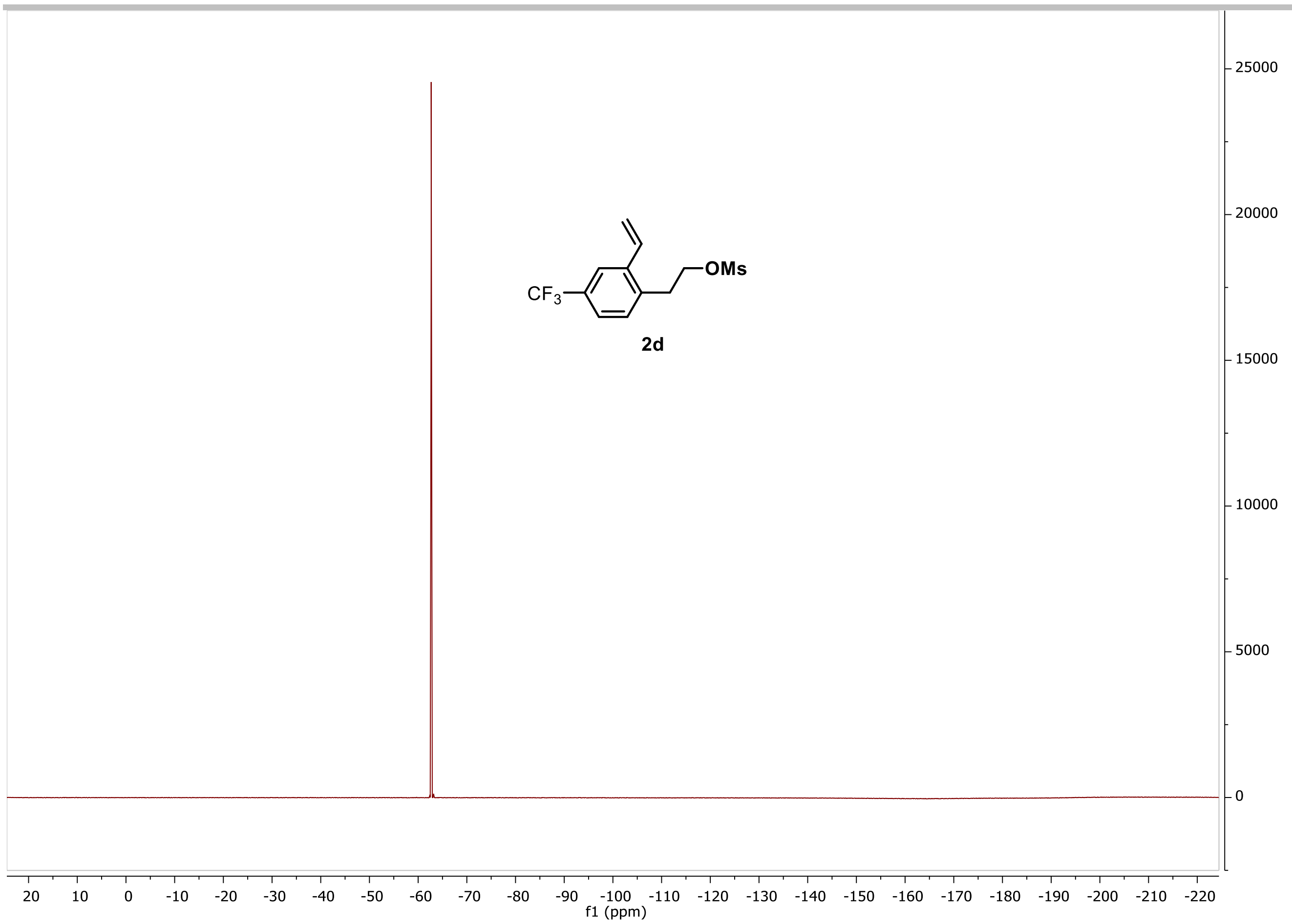

OMs
CF3
2d
25000
20000
15000
10000
5000
0
20 10 0 -10 -20 -30 -40 -50 -60 -70 -80 -90 -100 -110 -120 -130 -140 -150 -160 -170 -180 -190 -200 -210 -220
f1 (ppm)

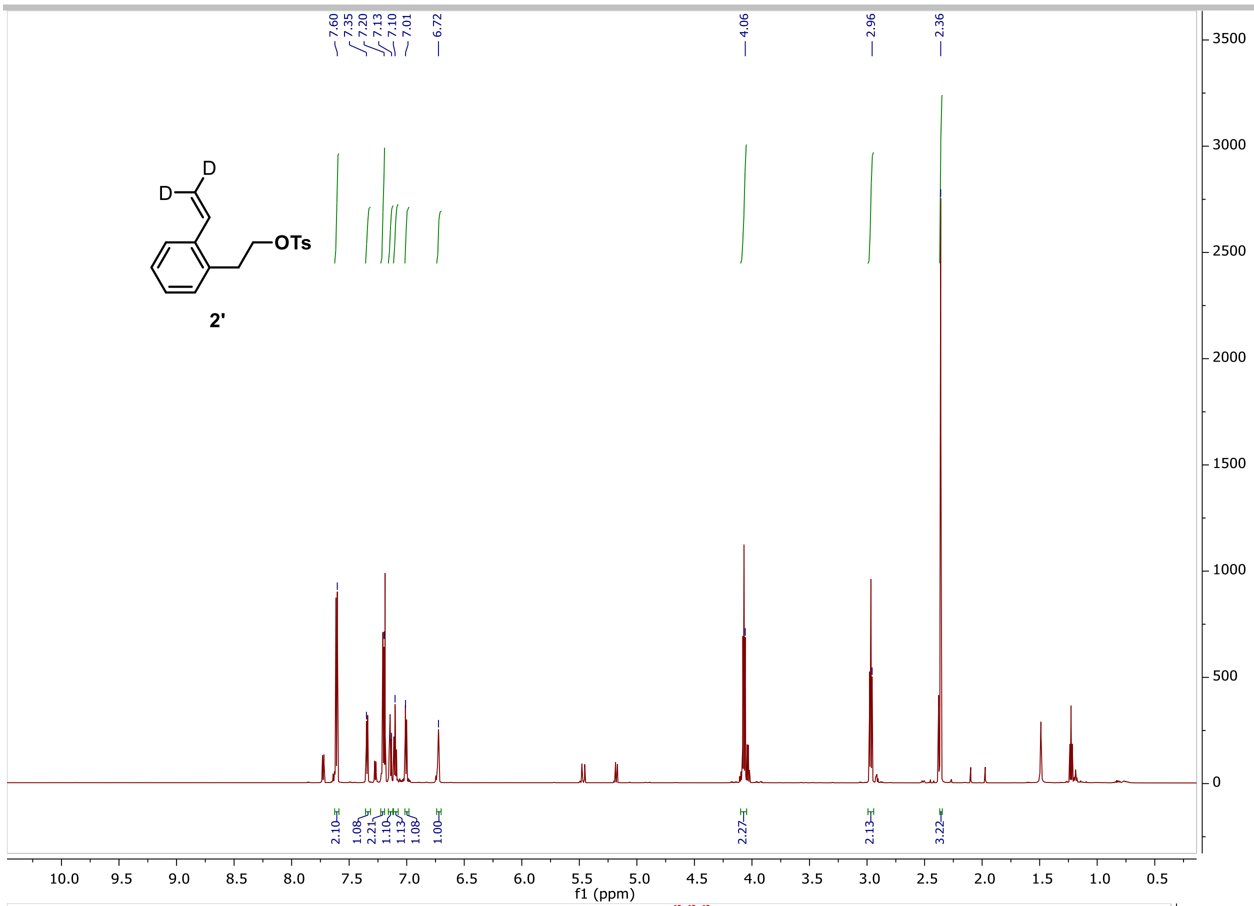
7.60
7.35
7.20
7.13
7.10
7.01
6.72
4.06
2.96
2.36
D
D
OTs
2'
2.10
1.08
2.21
1.10
1.13
1.08
1.00
2.27
2.13
3.22
f1 (ppm)

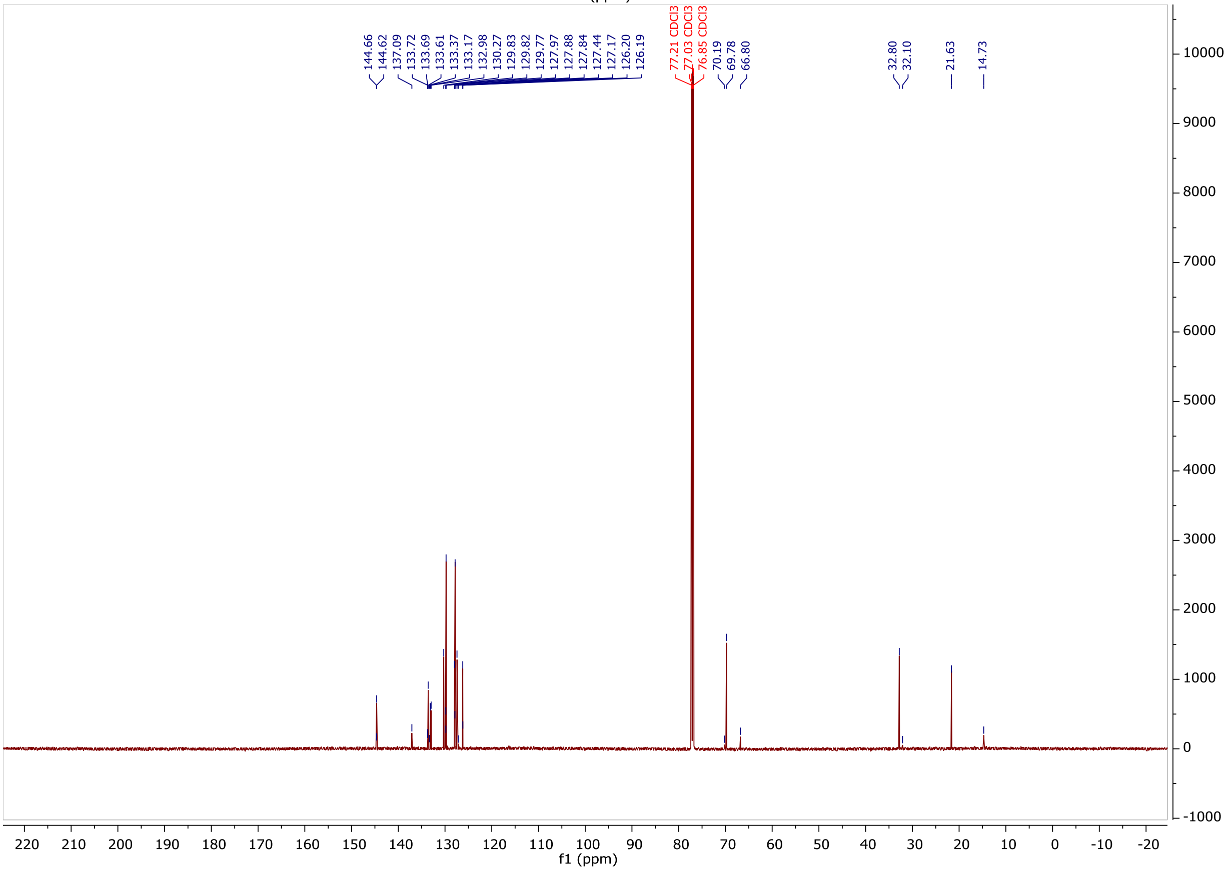
144.66
144.62
137.09
133.72
133.69
133.61
133.37
133.17
132.98
130.27
129.83
129.82
129.77
127.97
127.88
127.84
127.44
127.17
126.20
126.19
77.21 CDCl3
77.03 CDCl3
76.85 CDCl3
70.19
69.78
66.80
32.80
32.10
21.63
14.73
f1 (ppm)

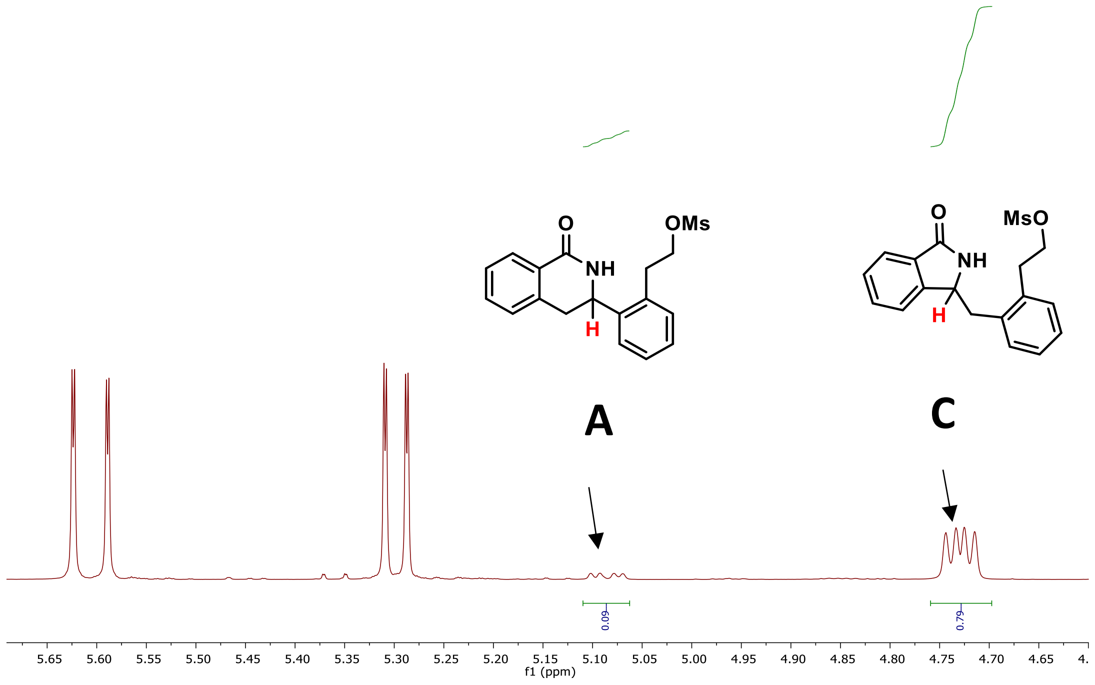
OMs
O
NH
H
A
MsO
O
NH
H
C
0.09
0.79
5.65 5.60 5.55 5.50 5.45 5.40 5.35 5.30 5.25 5.20 5.15 5.10 5.05 5.00 4.95 4.90 4.85 4.80 4.75 4.70 4.65
f1 (ppm)

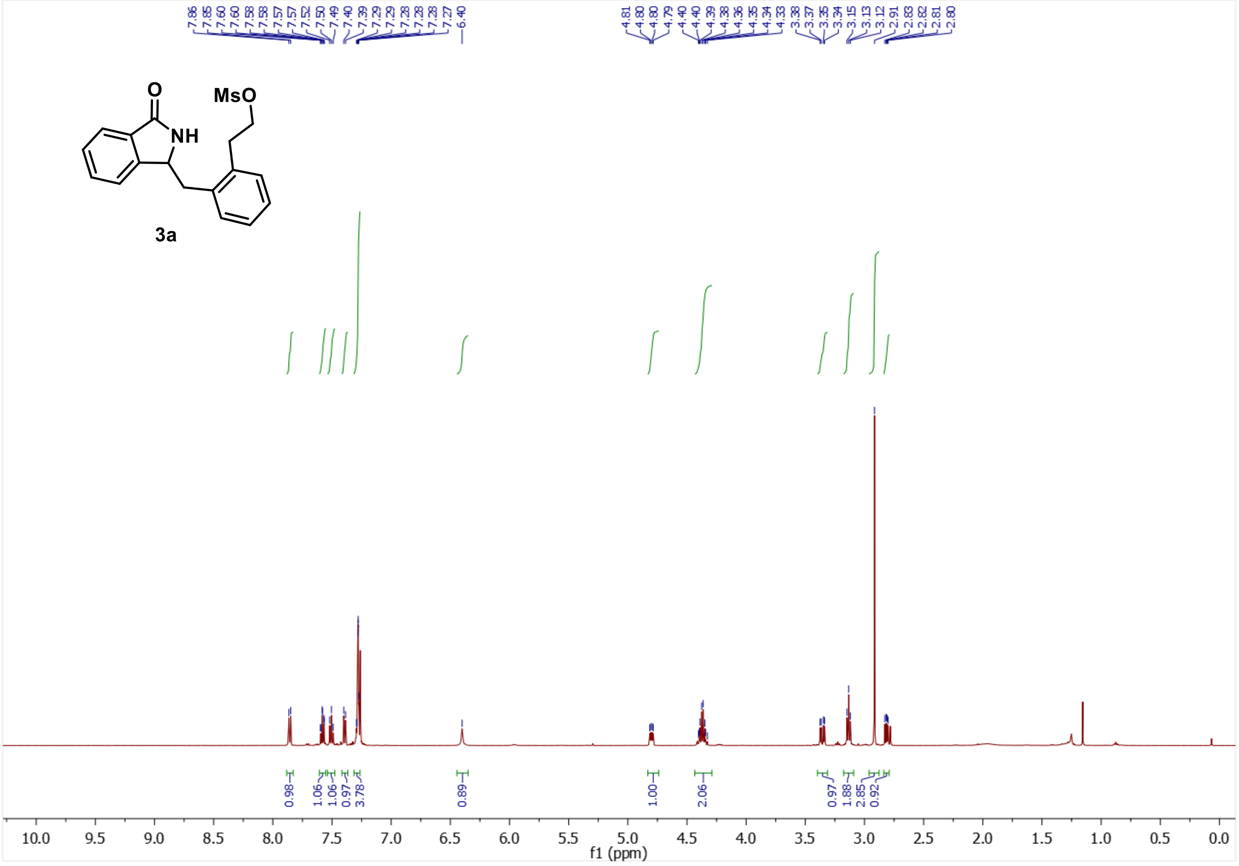

7.86 7.85 7.60 7.60 7.58 7.58 7.57 7.57 7.52 7.50 7.49 7.40 7.39 7.29 7.29 7.28 7.28 7.28 7.28 7.27 6.40
4.81 4.80 4.80 4.79 4.40 4.40 4.39 4.38 4.36 4.35 4.34 4.33 3.38 3.37 3.35 3.34 3.15 3.13 3.12 2.91 2.83 2.82 2.81 2.80
O
NH
MsO
3a
0.98 1.06 1.06 0.97 3.78 0.89 1.00 2.06 0.97 1.88 2.85 0.92
10.0 9.5 9.0 8.5 8.0 7.5 7.0 6.5 6.0 5.5 5.0 4.5 4.0 3.5 3.0 2.5 2.0 1.5 1.0 0.5 0.0
f1 (ppm)

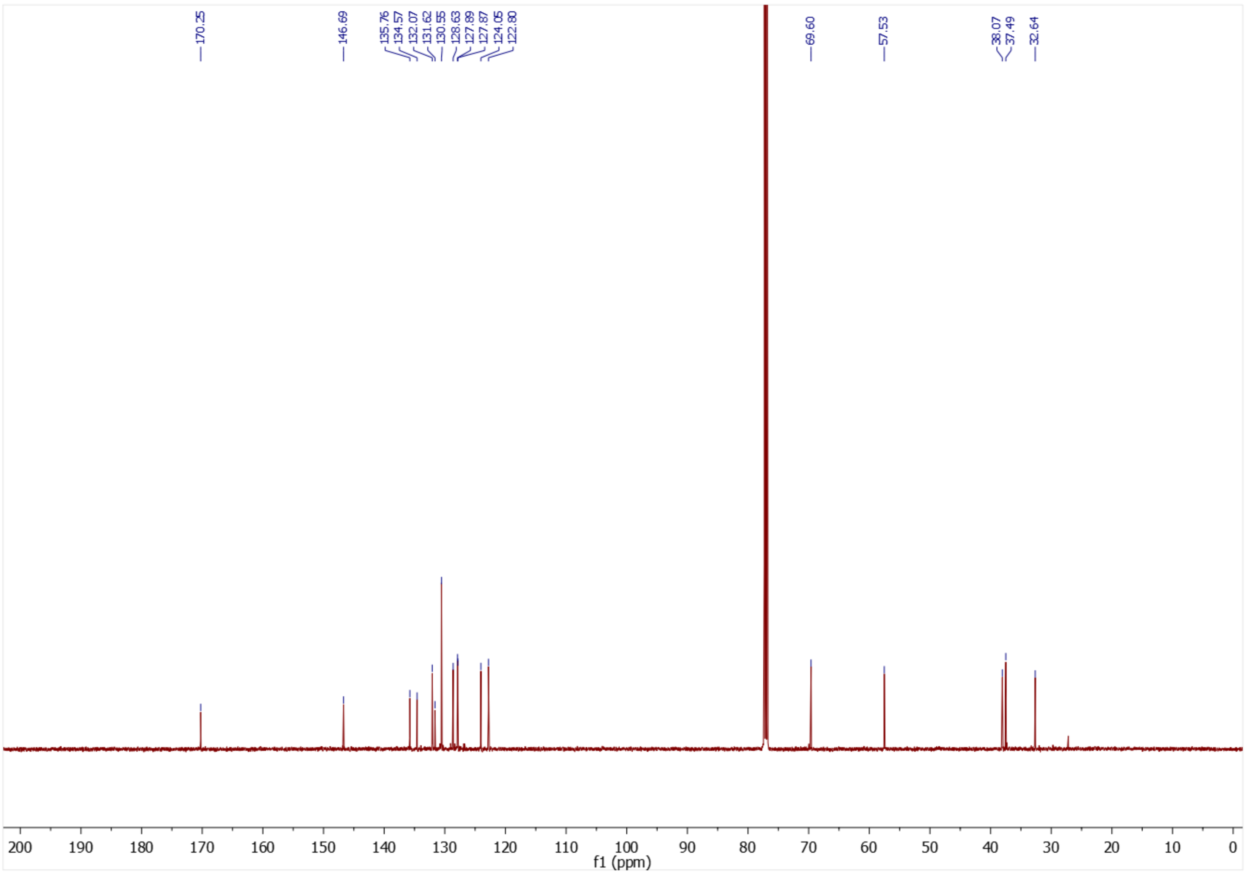

170.25
146.69
135.76 134.57 132.07 131.62 130.55 128.63 127.89 127.87 124.05 122.80
69.60
57.53
38.07 37.49
32.64
200 190 180 170 160 150 140 130 120 110 100 90 80 70 60 50 40 30 20 10 0
f1 (ppm)

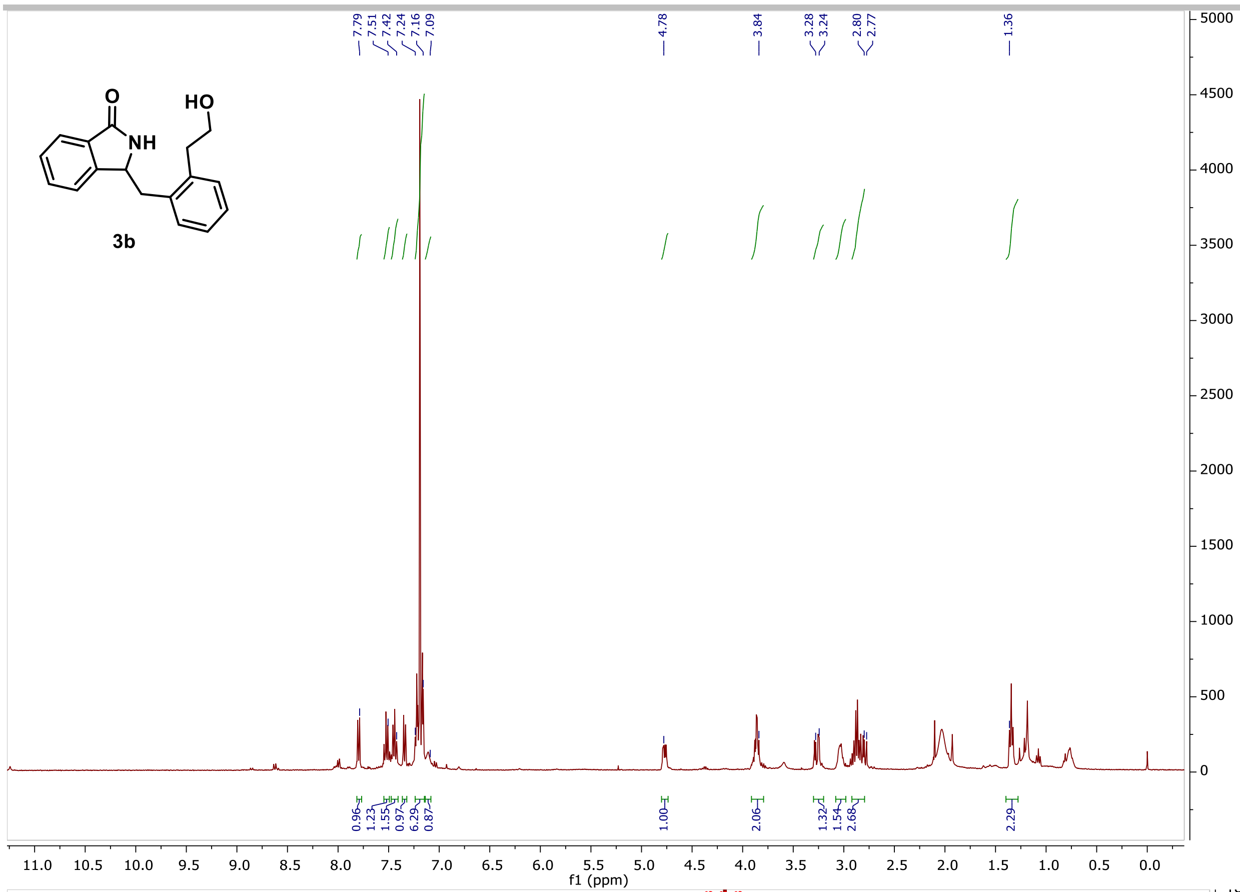
7.79
7.51
7.42
7.24
7.16
7.09
4.78
3.84
3.28
3.24
2.80
2.77
1.36
O
NH
HO
3b
0.96
1.23
1.55
0.97
6.29
0.87
1.00
2.06
1.32
1.54
2.68
2.29
f1 (ppm)

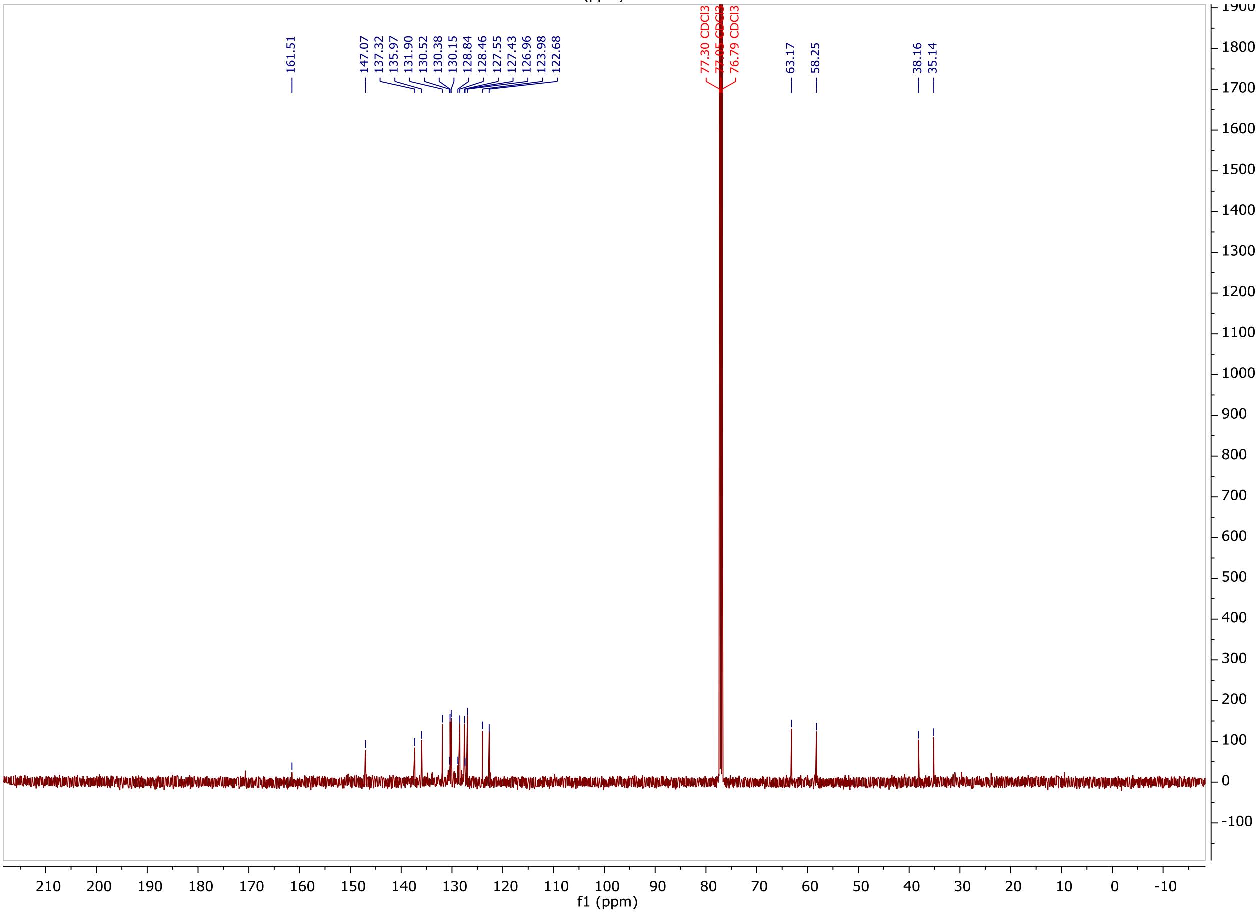
161.51
147.07
137.32
135.97
131.90
130.52
130.38
130.15
128.84
128.46
127.55
127.43
126.96
123.98
122.68
77.30 CDCl3
77.05 CDCl3
76.79 CDCl3
63.17
58.25
38.16
35.14
f1 (ppm)

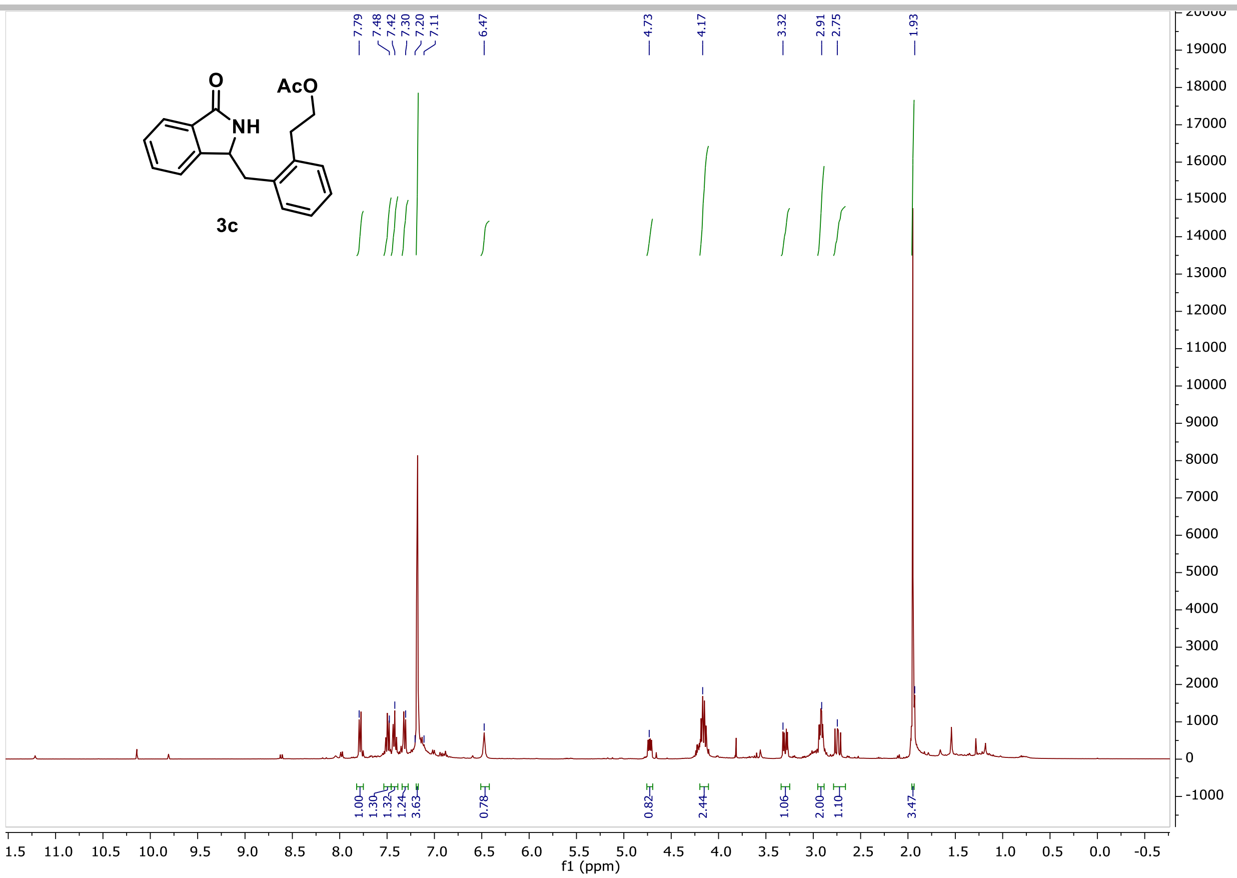
7.79
7.48
7.42
7.30
7.20
7.11
6.47
4.73
4.17
3.32
2.91
2.75
1.93
O
AcO
NH
3c
1.00
1.30
1.32
1.24
3.63
0.78
0.82
2.44
1.06
2.00
1.10
3.47
f1 (ppm)

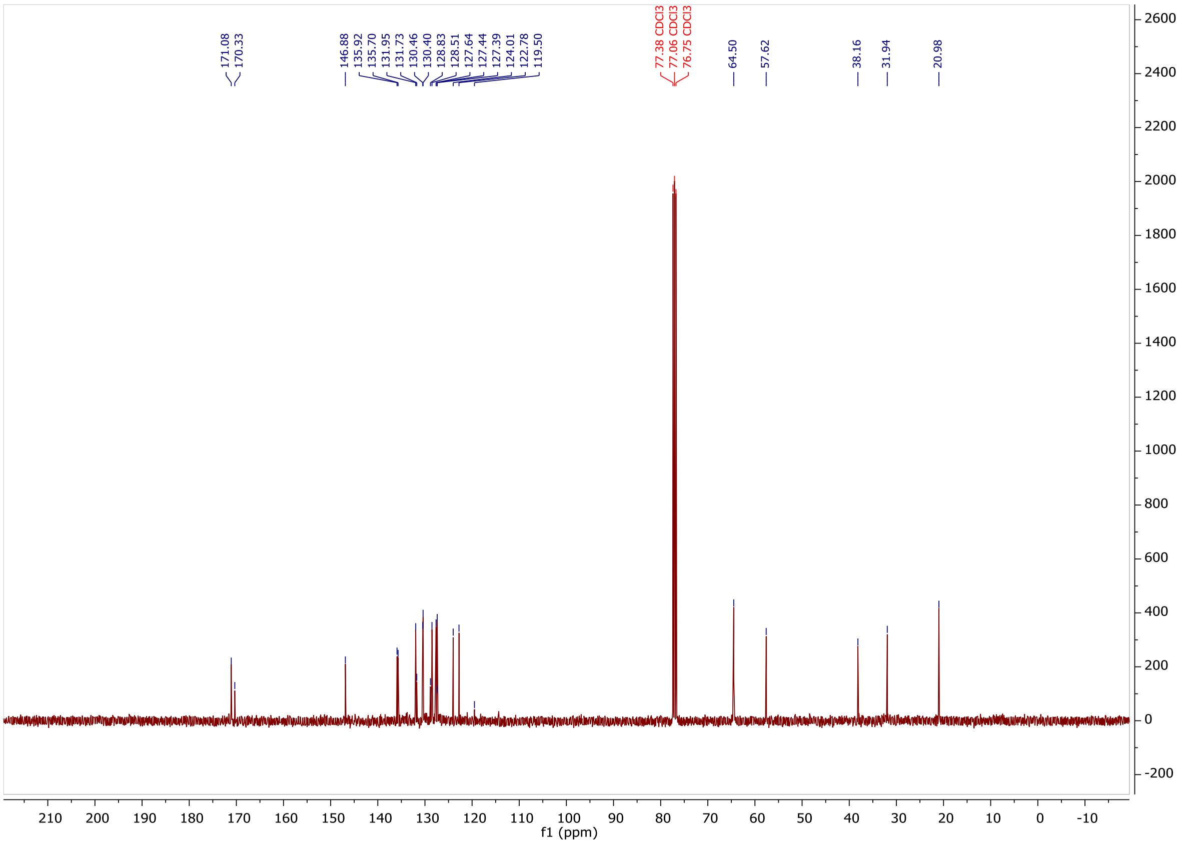
171.08
170.33
146.88
135.92
135.70
131.95
131.73
130.46
130.40
128.83
128.51
127.64
127.44
127.39
124.01
122.78
119.50
77.38 CDCl3
77.06 CDCl3
76.75 CDCl3
64.50
57.62
38.16
31.94
20.98
f1 (ppm)

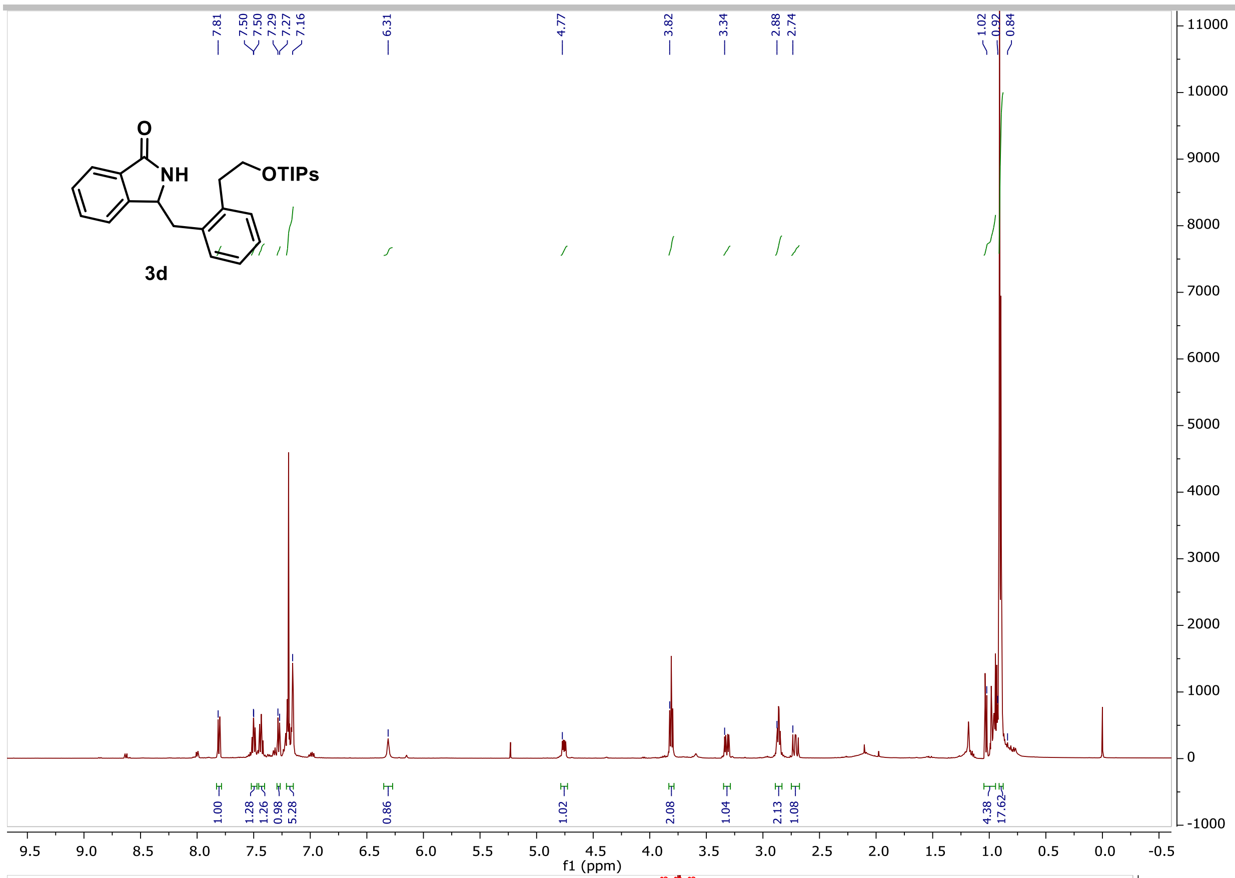
7.81
7.50
7.50
7.29
7.27
7.16
6.31
4.77
3.82
3.34
2.88
2.74
1.02
0.92
0.84
NH
O
OTIPs
3d
1.00
1.28
1.26
0.98
5.28
0.86
1.02
2.08
1.04
2.13
1.08
4.38
17.62
f1 (ppm)

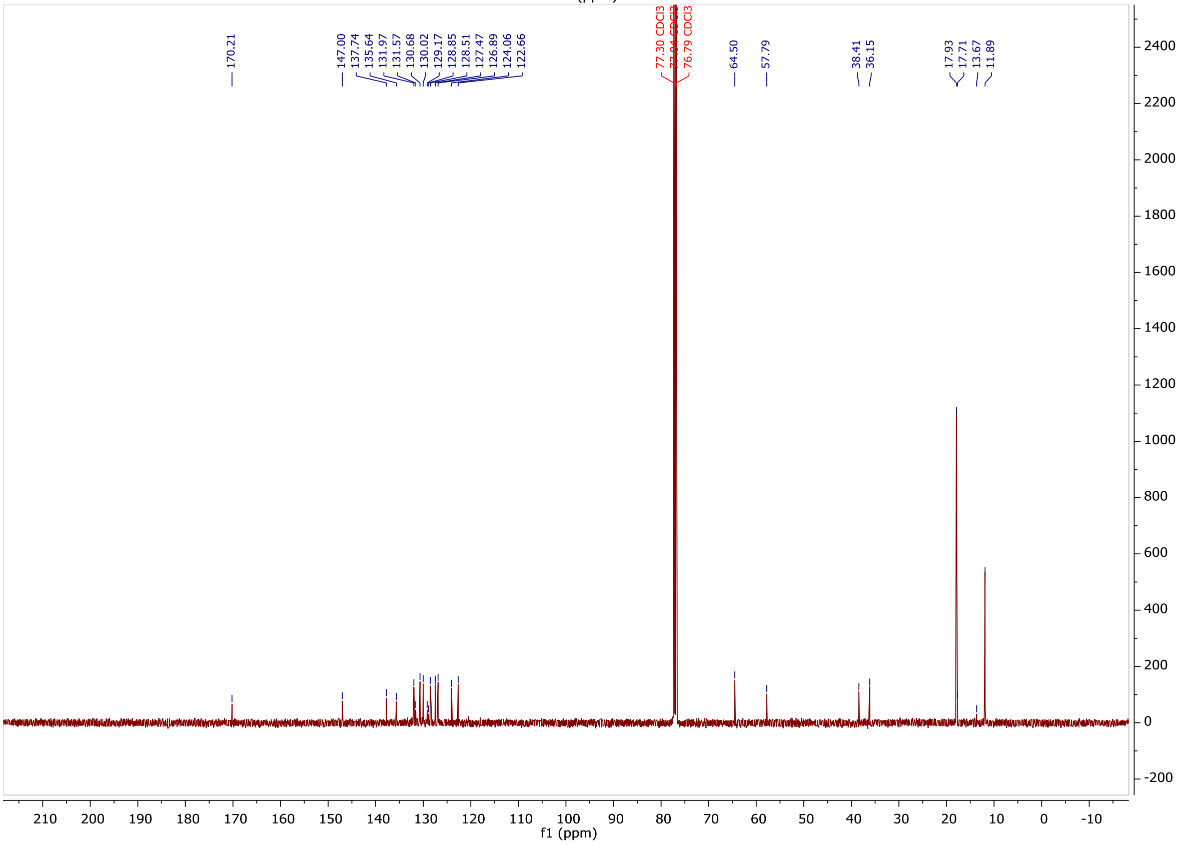
170.21
147.00
137.74
135.64
131.97
131.57
130.68
130.02
129.17
128.85
128.51
127.47
126.89
124.06
122.66
77.30 CDCl3
76.79 CDCl3
64.50
57.79
38.41
36.15
17.93
17.71
13.67
11.89
f1 (ppm)

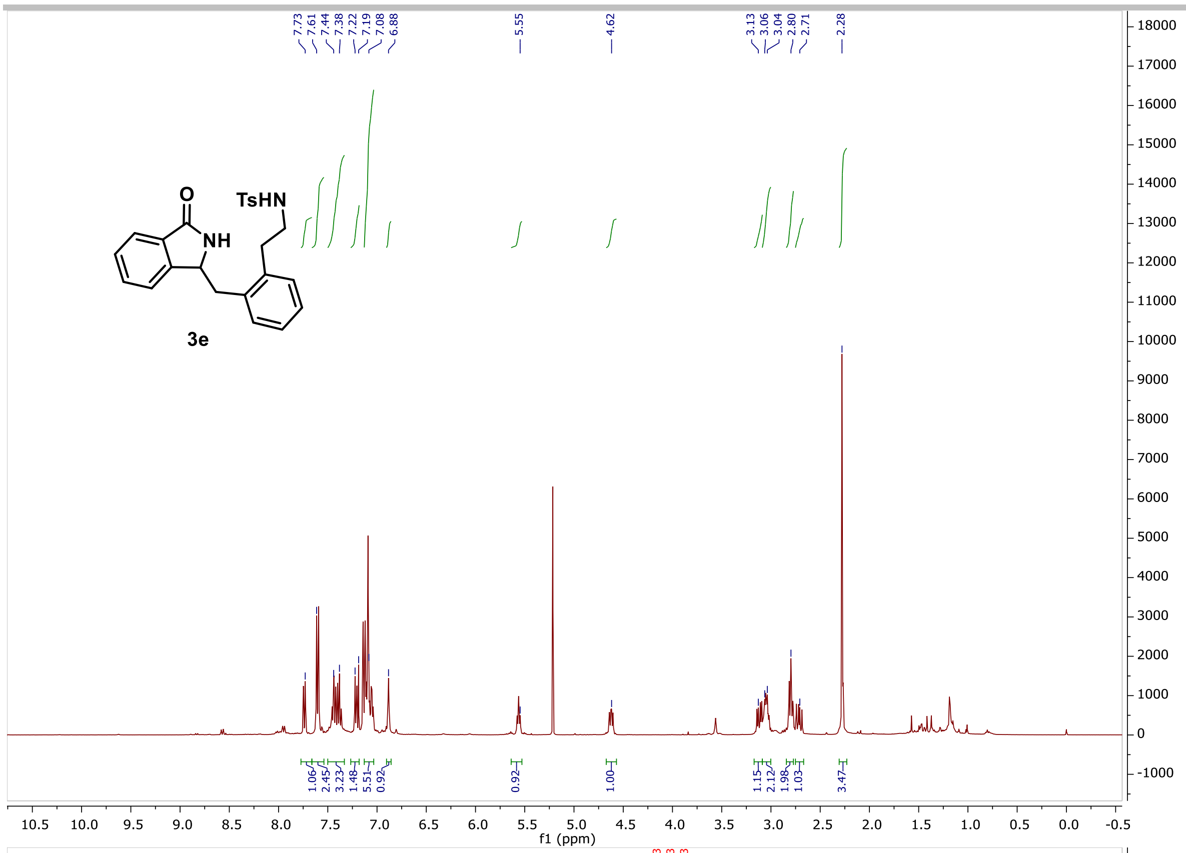
7.73
7.61
7.44
7.38
7.22
7.19
7.08
6.88
5.55
4.62
3.13
3.06
3.04
2.80
2.71
2.28
TsHN
O
NH
3e
1.06
2.45
3.23
1.48
5.51
0.92
0.92
1.00
1.15
2.12
1.98
1.03
3.47
f1 (ppm)

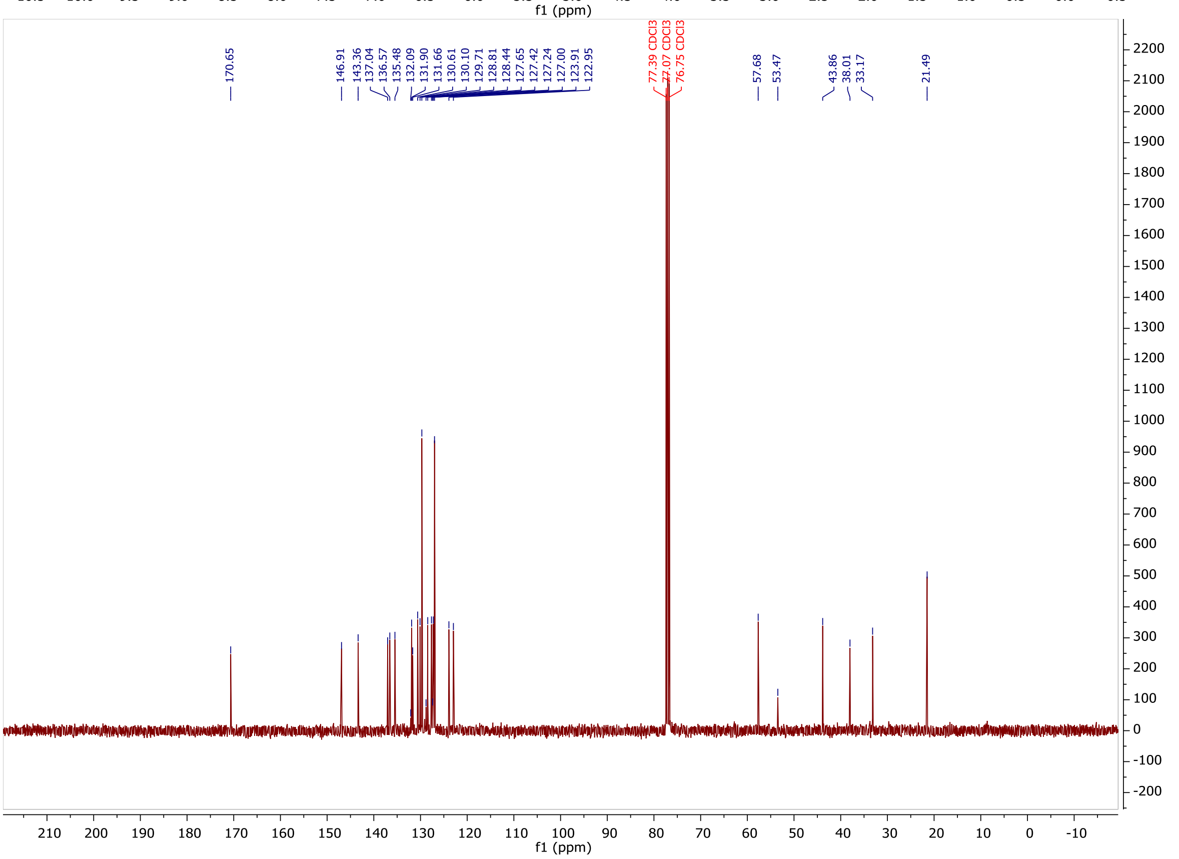
170.65
146.91
143.36
137.04
136.57
135.48
132.09
131.90
131.66
130.61
130.10
129.71
128.81
128.44
127.65
127.42
127.24
127.00
123.91
122.95
77.39 CDCl3
77.07 CDCl3
76.75 CDCl3
57.68
53.47
43.86
38.01
33.17
21.49
f1 (ppm)

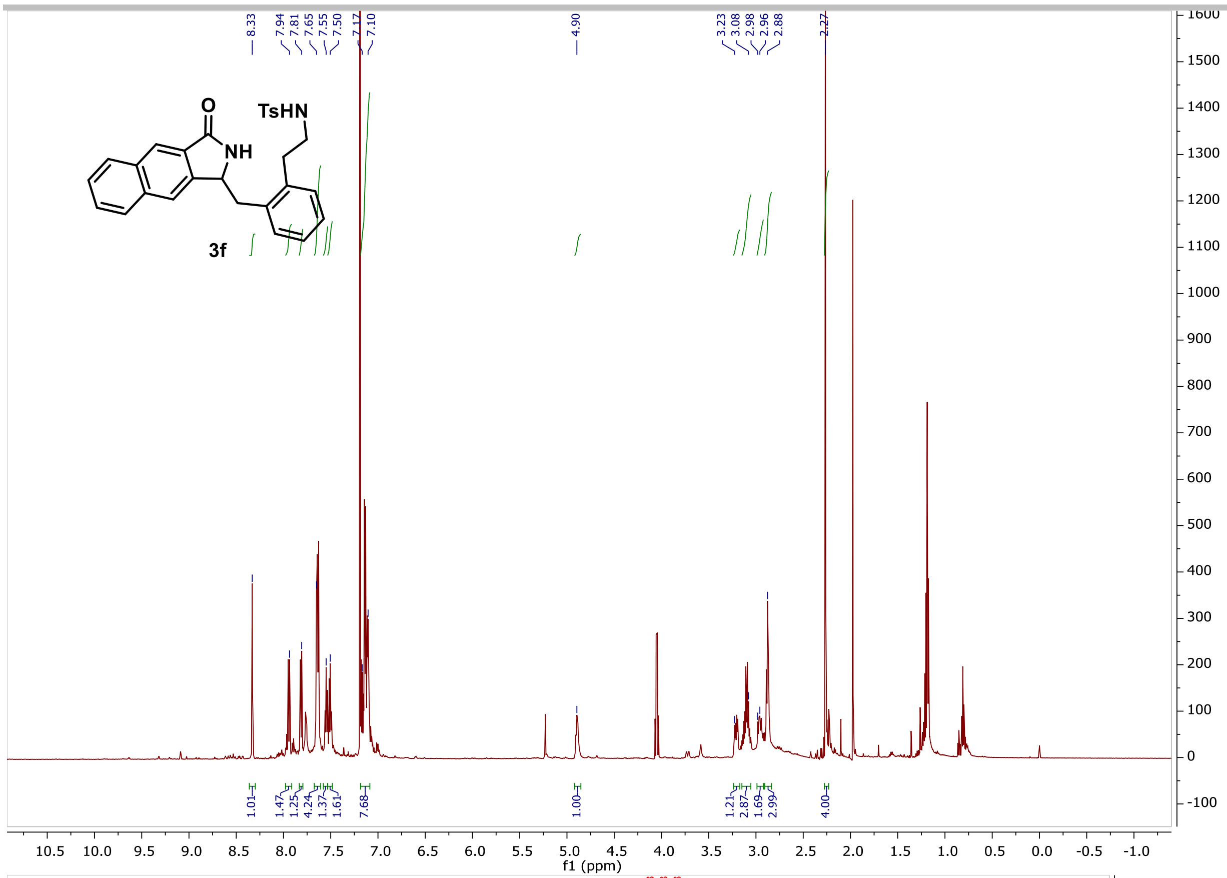
8.33
7.94
7.81
7.65
7.55
7.50
7.17
7.10
4.90
3.23
3.08
2.98
2.96
2.88
2.27
TsHN
O
NH
3f
1.01
1.47
1.25
4.24
1.37
1.61
7.68
1.00
1.21
2.87
1.69
2.99
4.00
f1 (ppm)

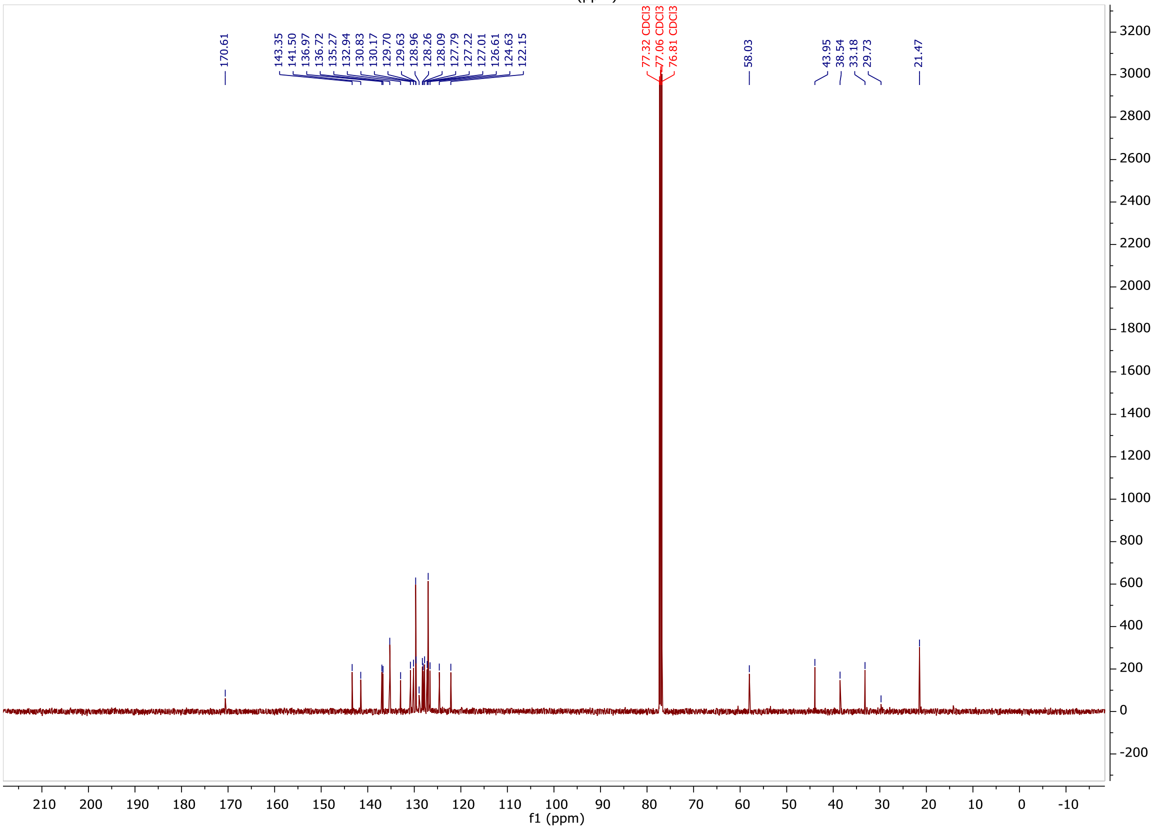
170.61
143.35
141.50
136.97
136.72
135.27
132.94
130.83
130.17
129.70
129.63
128.96
128.26
128.09
127.79
127.22
127.01
126.61
124.63
122.15
77.32 CDCl3
77.06 CDCl3
76.81 CDCl3
58.03
43.95
38.54
33.18
29.73
21.47
f1 (ppm)

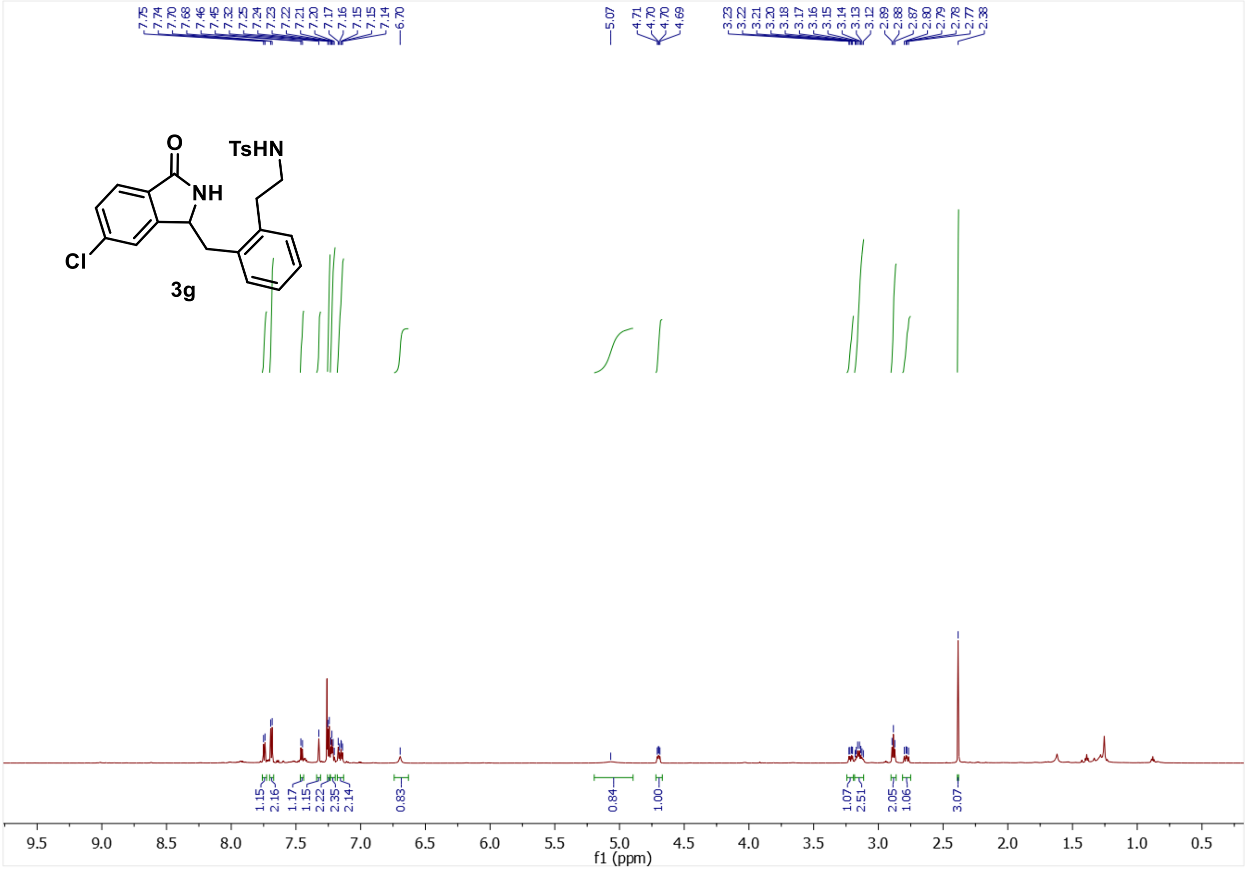
O
TsHN
NH
Cl
3g
7.75 7.74 7.70 7.68 7.46 7.45 7.32 7.25 7.24 7.23 7.22 7.21 7.20 7.17 7.16 7.15 7.15 7.14 6.70
5.07 4.71 4.70 4.70 4.69
3.23 3.22 3.21 3.20 3.18 3.17 3.16 3.15 3.14 3.13 3.12 2.89 2.88 2.87 2.80 2.79 2.78 2.77 2.38
1.15 2.16 1.17 1.15 2.22 2.35 2.14 0.83 0.84 1.00 1.07 2.51 2.05 1.06 3.07
9.5 9.0 8.5 8.0 7.5 7.0 6.5 6.0 5.5 5.0 4.5 4.0 3.5 3.0 2.5 2.0 1.5 1.0 0.5
f1 (ppm)

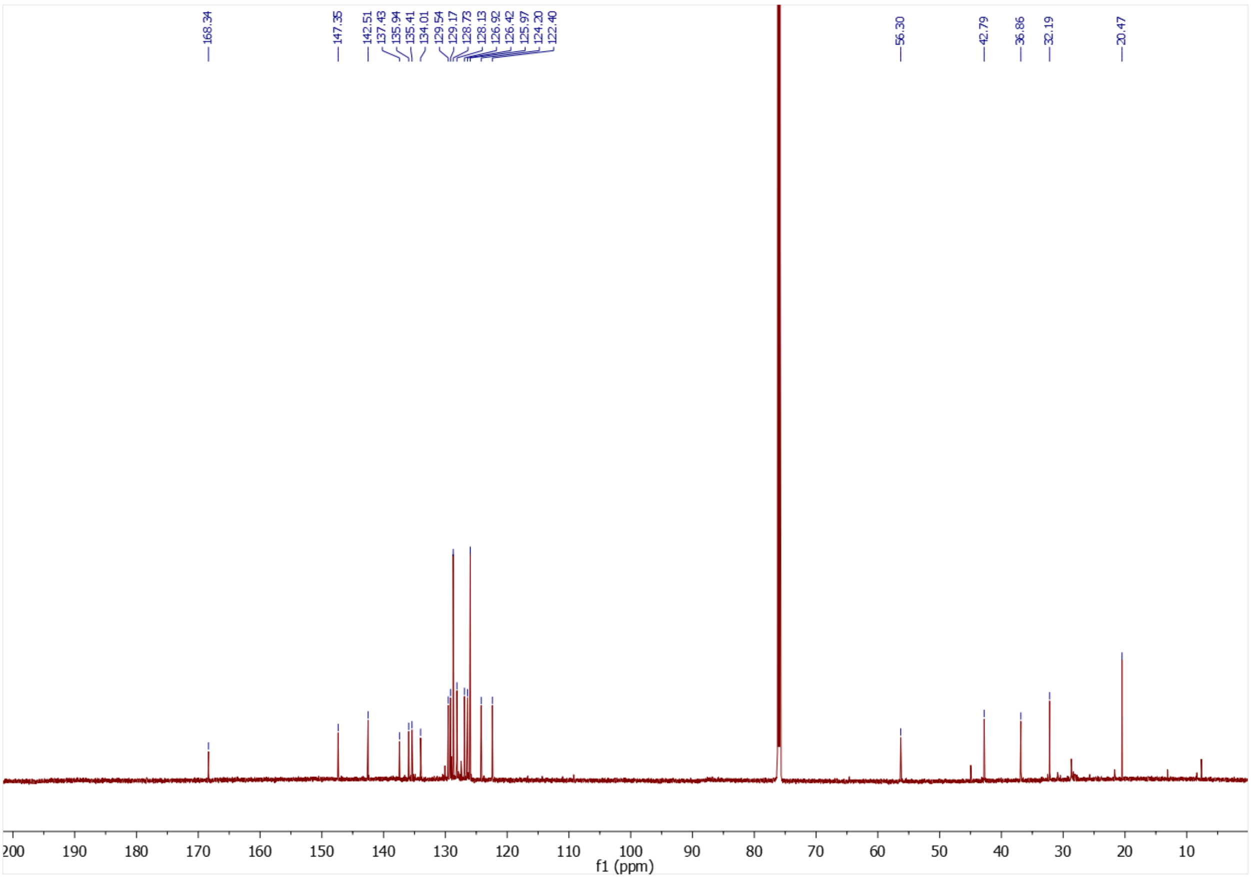
168.34
147.35 142.51 137.43 135.94 135.41 134.01 129.54 129.17 128.73 128.13 126.92 126.42 125.97 124.20 122.40
56.30
42.79
36.86
32.19
20.47
200 190 180 170 160 150 140 130 120 110 100 90 80 70 60 50 40 30 20 10
f1 (ppm)

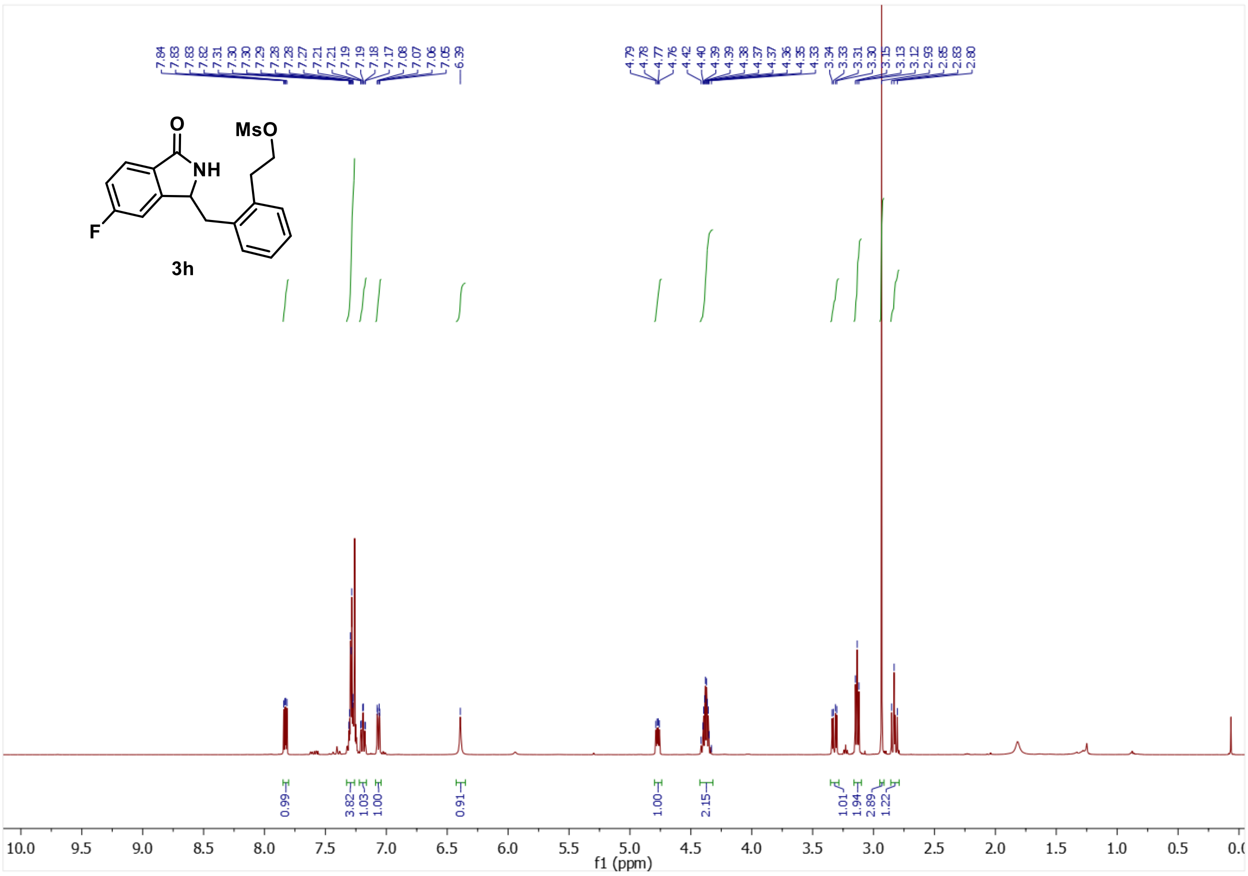
7.84
7.83
7.83
7.82
7.31
7.30
7.30
7.29
7.28
7.28
7.27
7.21
7.21
7.19
7.19
7.18
7.17
7.08
7.07
7.06
7.05
6.39
4.79
4.78
4.77
4.76
4.42
4.40
4.39
4.39
4.38
4.37
4.37
4.36
4.35
4.33
3.34
3.33
3.33
3.31
3.30
3.15
3.13
3.12
2.93
2.85
2.83
2.80
O
MsO
NH
F
3h
0.99
3.82
1.03
1.00
0.91
1.00
2.15
1.01
1.94
2.89
1.22
10.0 9.5 9.0 8.5 8.0 7.5 7.0 6.5 6.0 5.5 5.0 4.5 4.0 3.5 3.0 2.5 2.0 1.5 1.0 0.5 0.0
f1 (ppm)

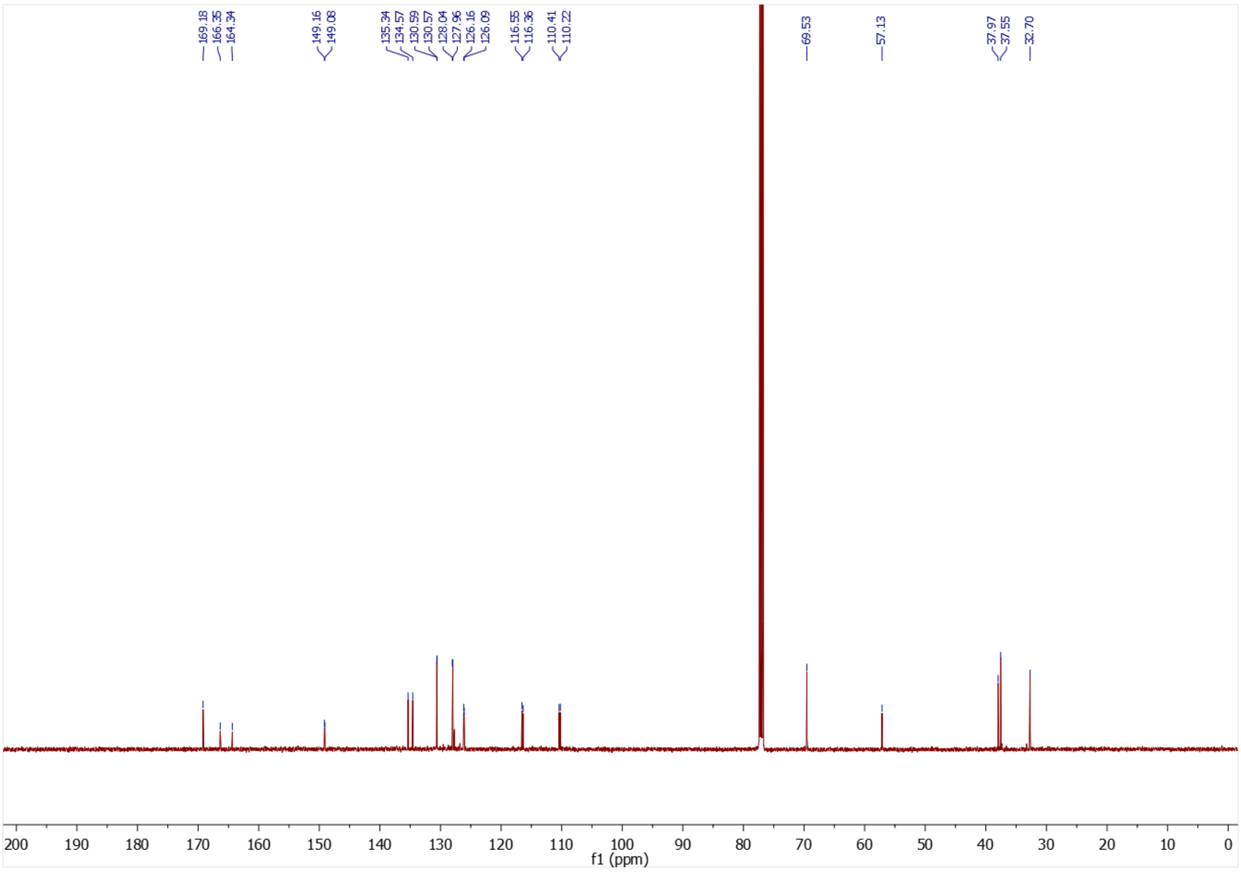
169.18
166.35
164.34
149.16
149.08
135.34
134.57
130.59
130.57
128.04
127.96
126.16
126.09
116.55
116.36
110.41
110.22
69.53
57.13
37.97
37.55
32.70
200 190 180 170 160 150 140 130 120 110 100 90 80 70 60 50 40 30 20 10 0
f1 (ppm)

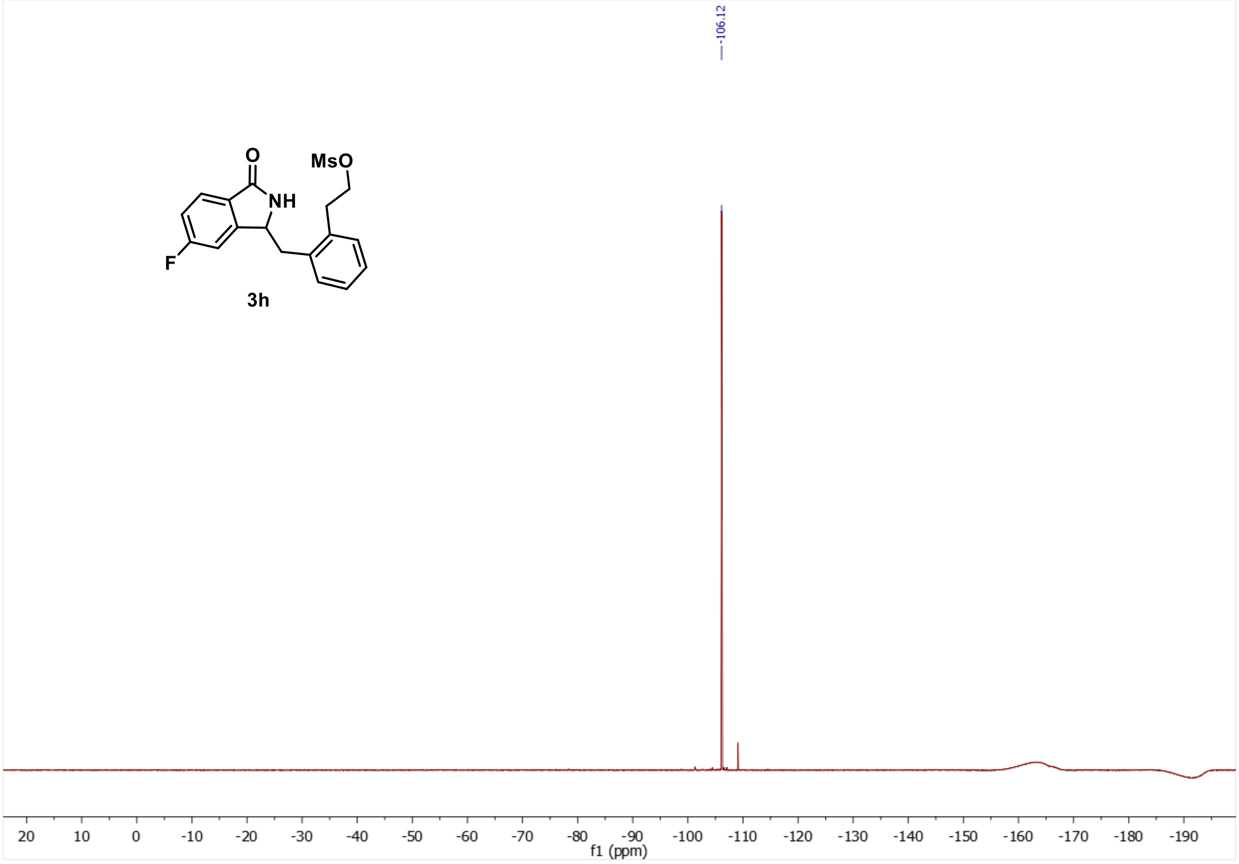
-106.12
O
MsO
NH
F
3h
20
10
0
-10
-20
-30
-40
-50
-60
-70
-80
-90
-100
-110
-120
-130
-140
-150
-160
-170
-180
-190
f1 (ppm)

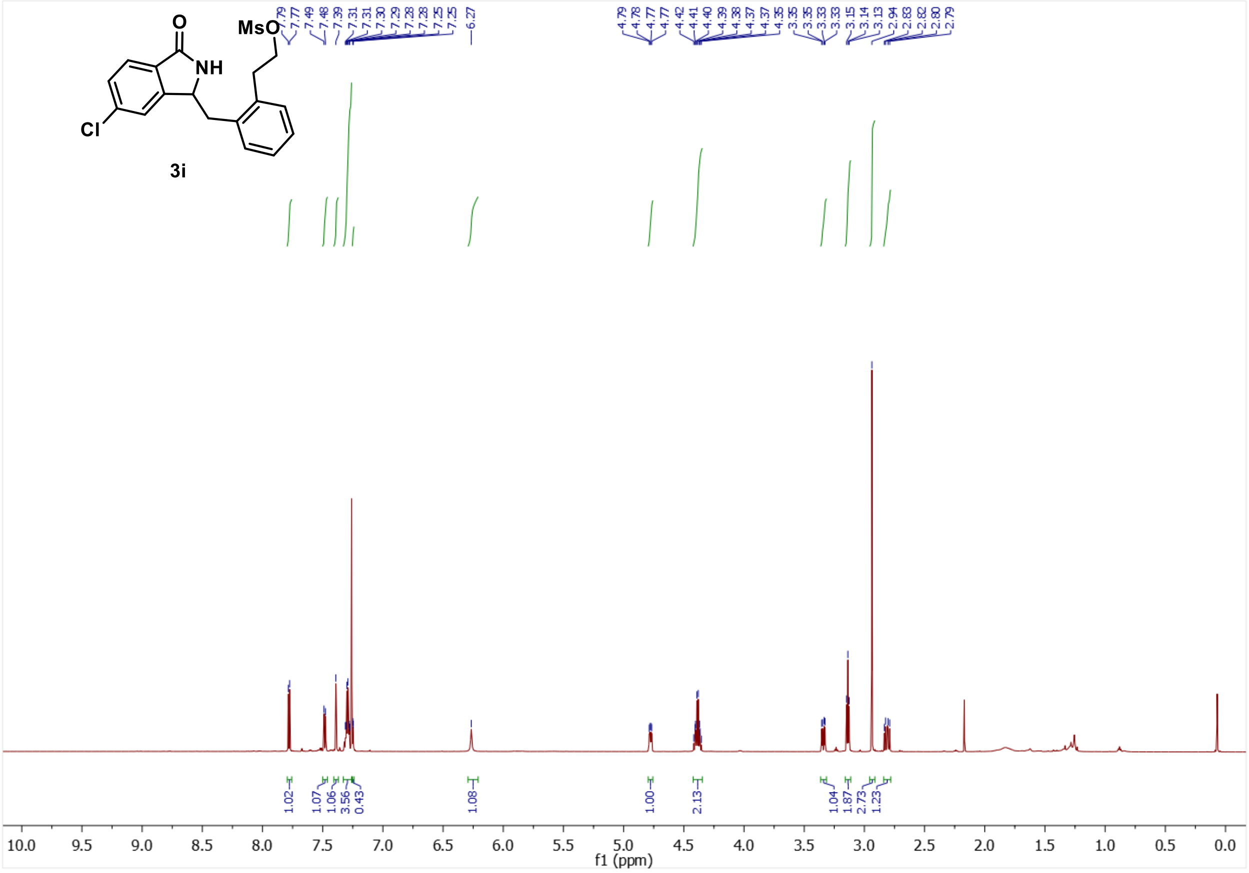
O
NH
MsO
Cl
3i
7.79
7.77
7.49
7.48
7.39
7.31
7.31
7.30
7.29
7.28
7.28
7.25
7.25
6.27
4.79
4.78
4.77
4.77
4.42
4.41
4.40
4.39
4.38
4.37
4.37
4.35
3.35
3.35
3.33
3.33
3.15
3.14
3.13
2.94
2.83
2.82
2.80
2.79
1.02
1.07
1.06
3.56
0.43
1.08
1.00
2.13
1.04
1.87
2.73
1.23
10.0 9.5 9.0 8.5 8.0 7.5 7.0 6.5 6.0 5.5 5.0 4.5 4.0 3.5 3.0 2.5 2.0 1.5 1.0 0.5 0.0
f1 (ppm)

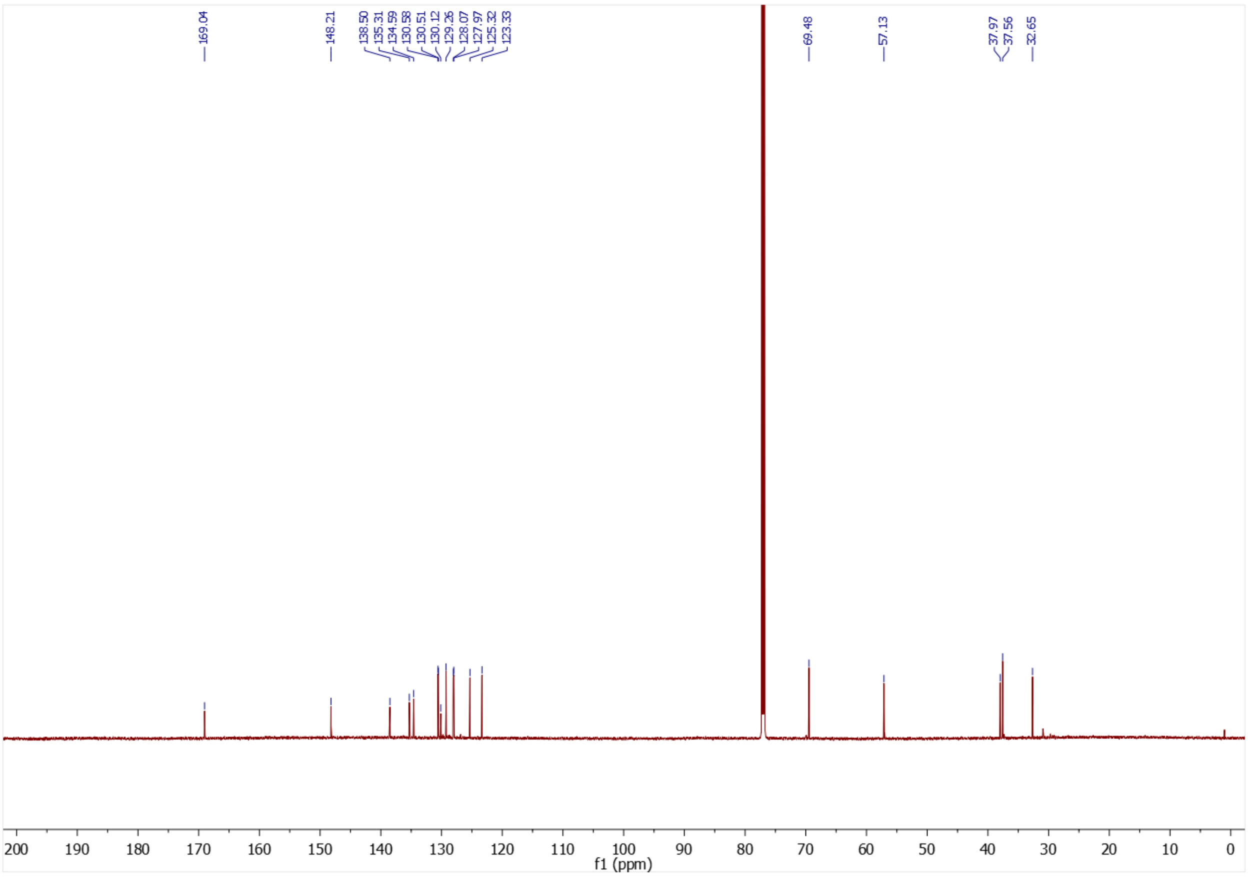
169.04
148.21
138.50
135.31
134.59
130.58
130.51
130.12
129.26
128.07
127.97
125.32
123.33
69.48
57.13
37.97
37.56
32.65
200 190 180 170 160 150 140 130 120 110 100 90 80 70 60 50 40 30 20 10 0
f1 (ppm)

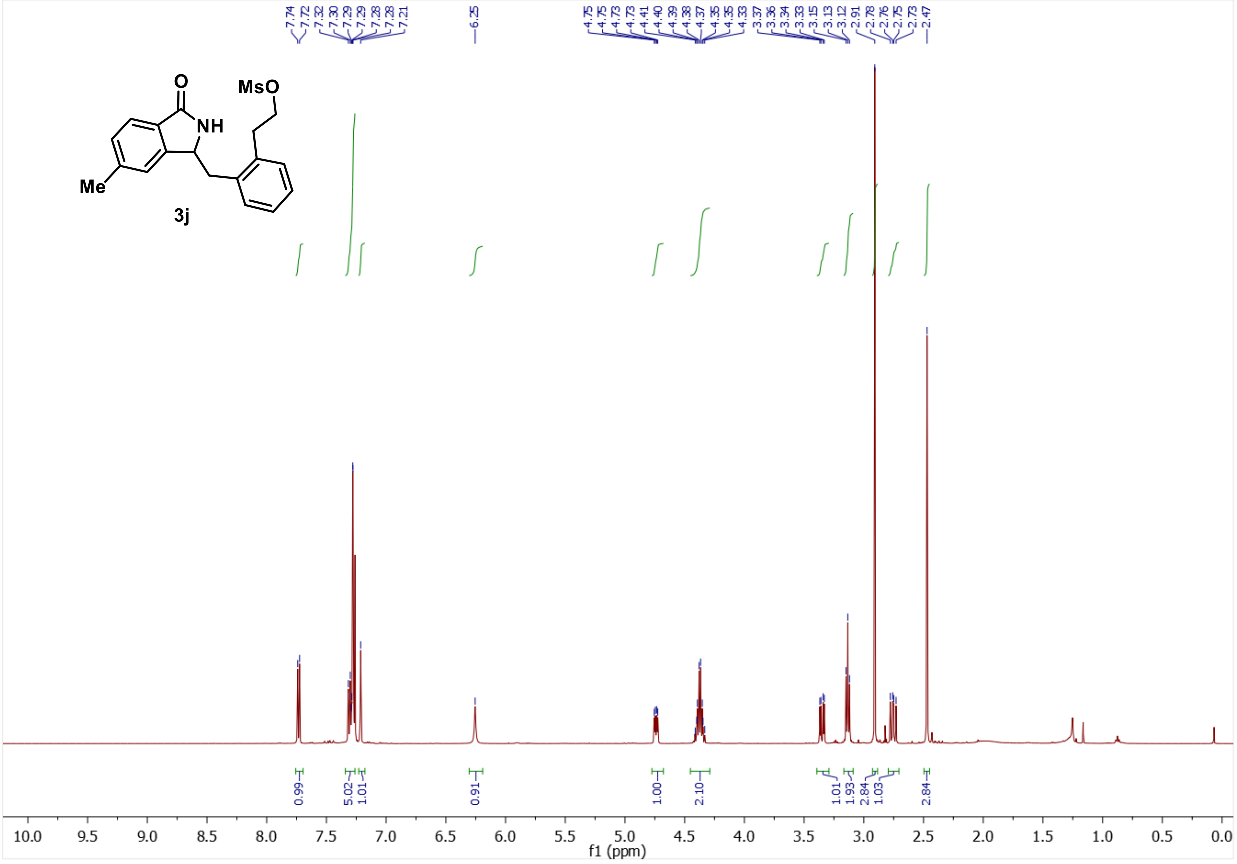

MsO
O
NH
Me
3j
7.74
7.72
7.32
7.30
7.29
7.29
7.28
7.28
7.21
6.25
4.75
4.75
4.73
4.73
4.41
4.40
4.39
4.38
4.37
4.35
4.35
4.33
3.37
3.36
3.34
3.33
3.15
3.13
3.12
2.91
2.78
2.76
2.75
2.73
2.47
0.99
5.02
1.01
0.91
1.00
2.10
1.01
1.93
2.84
1.03
2.84
f1 (ppm)

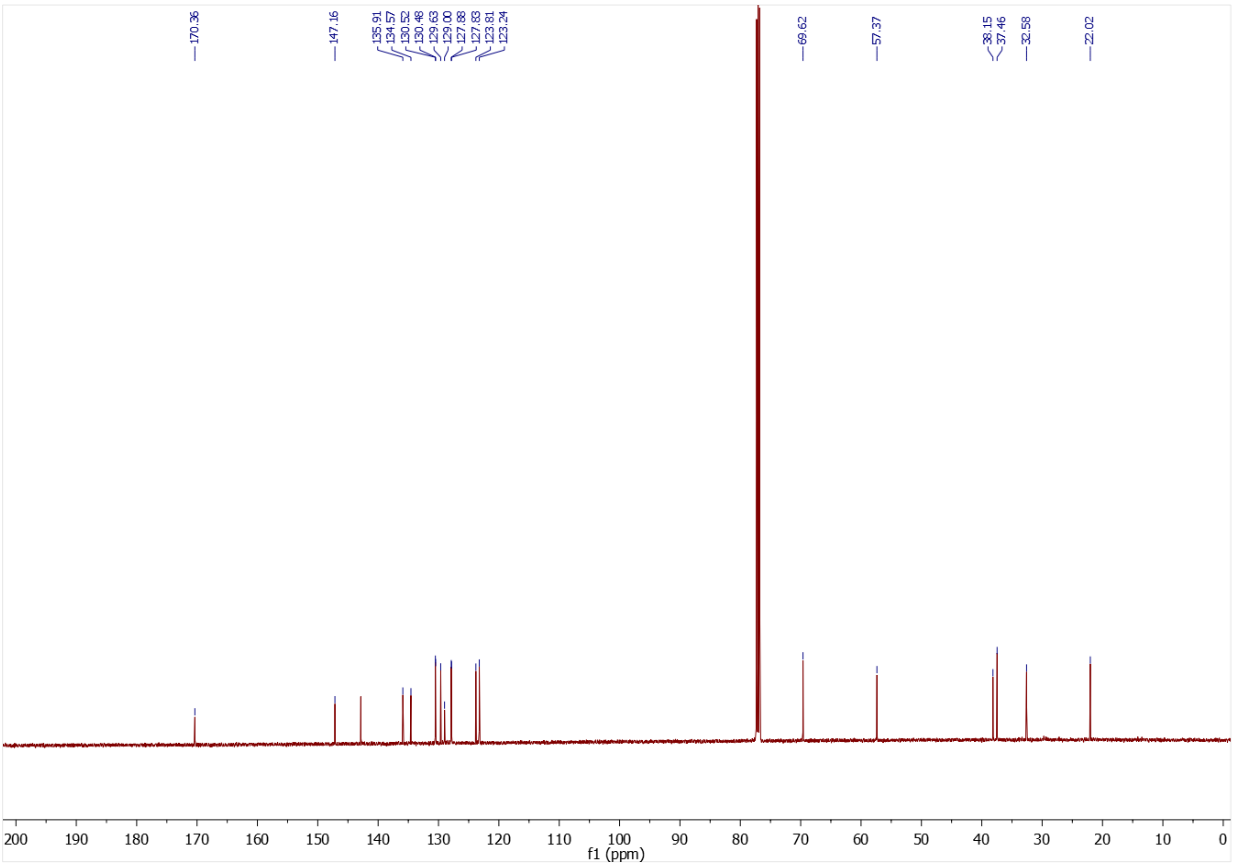

170.36
147.16
135.91
134.57
130.52
130.48
129.63
129.00
127.88
127.83
123.81
123.24
69.62
57.37
38.15
37.46
32.58
22.02
f1 (ppm)

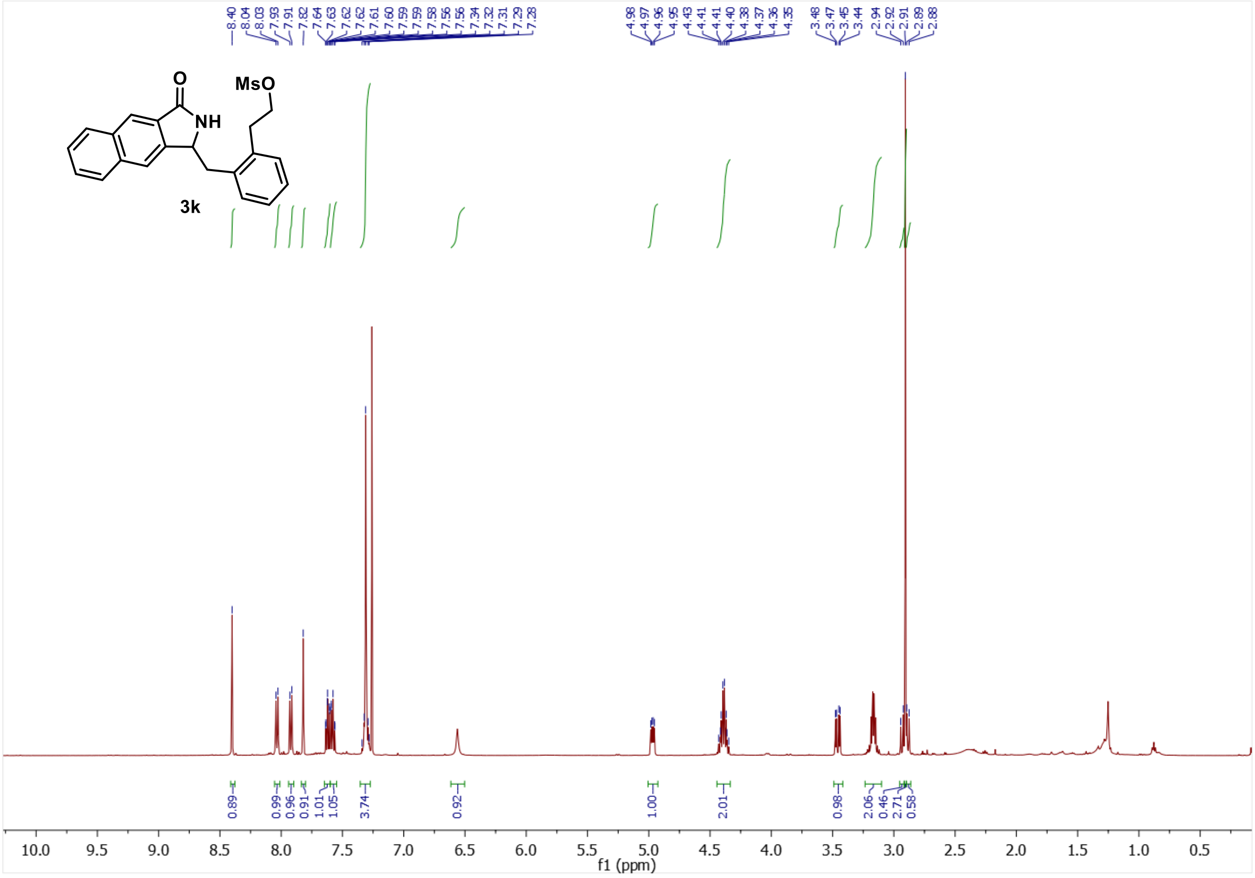
MsO
O
NH
3k
f1 (ppm)

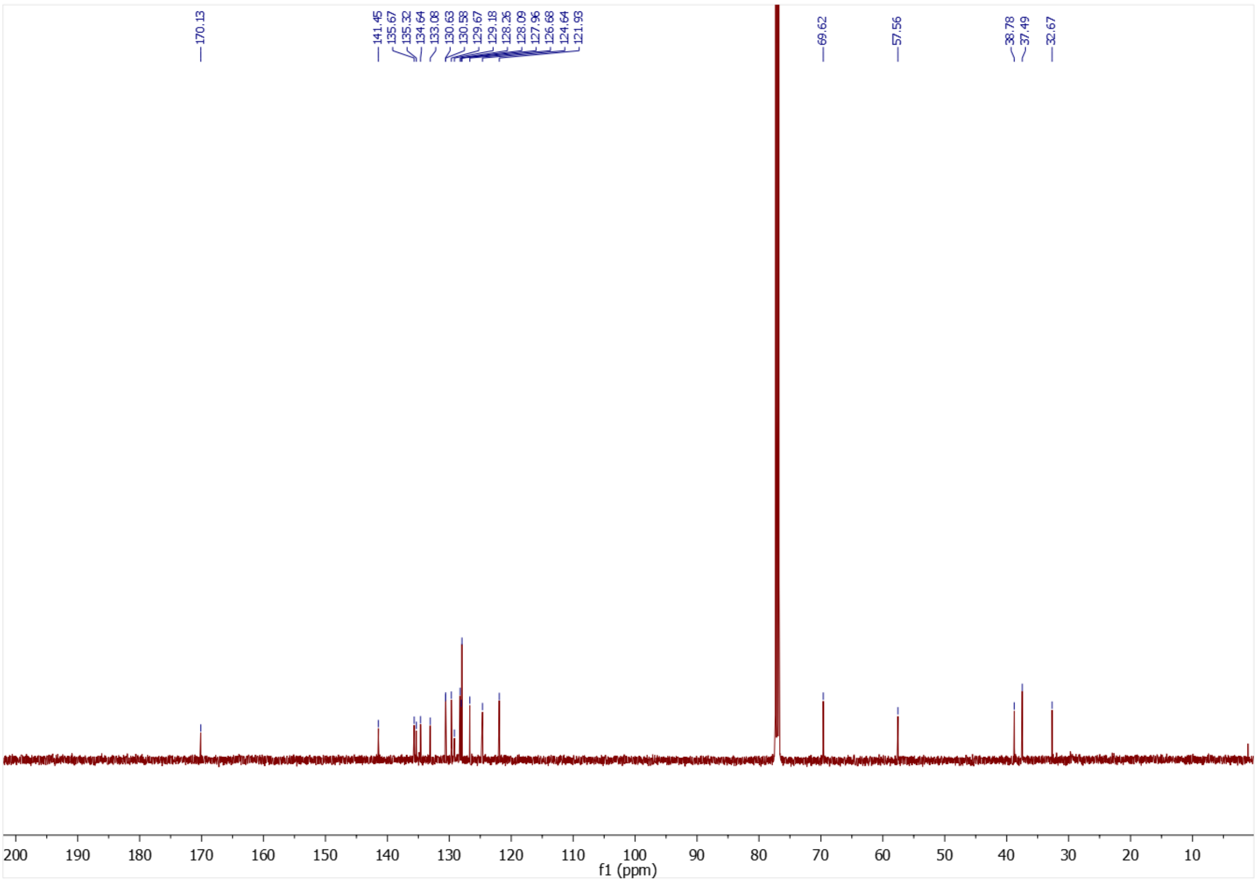
170.13
69.62
57.56
38.78
37.49
32.67
f1 (ppm)

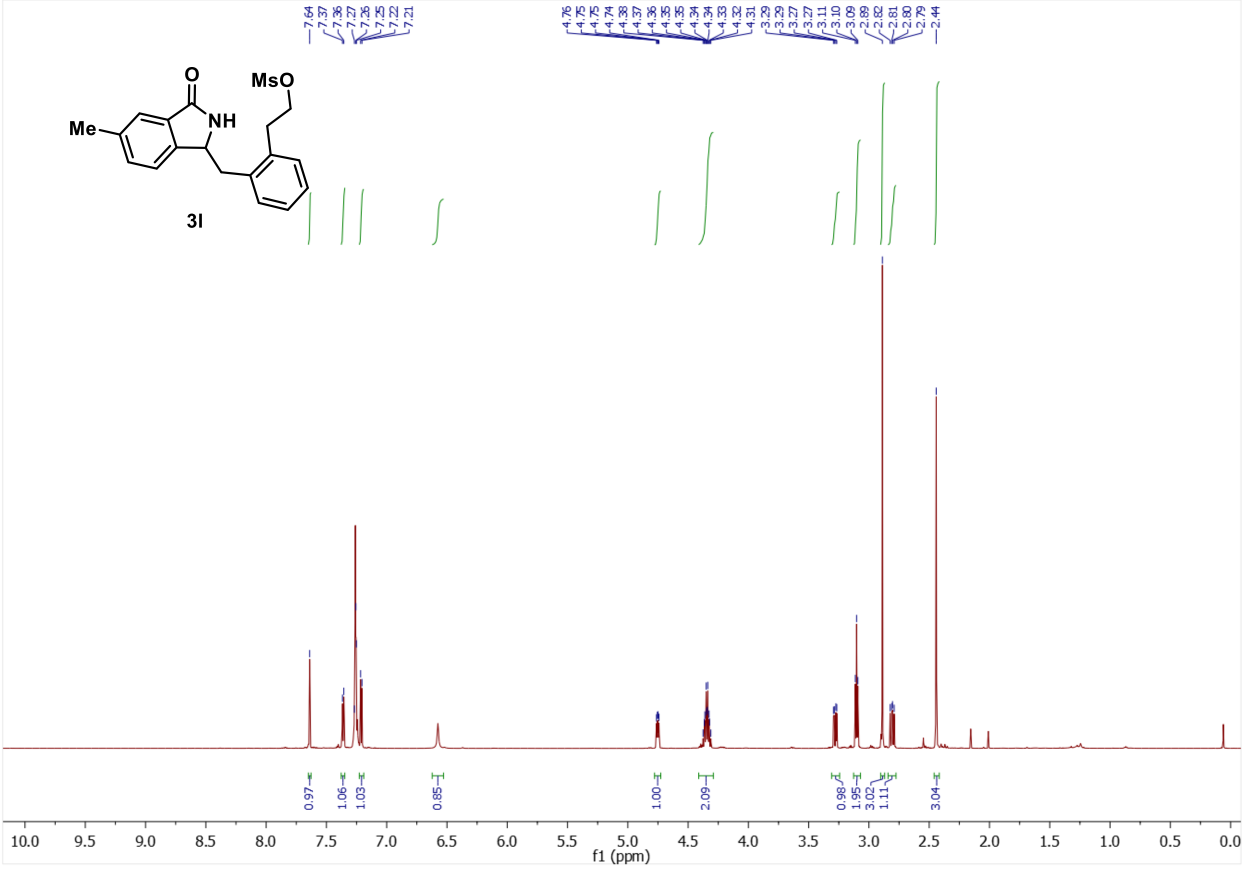

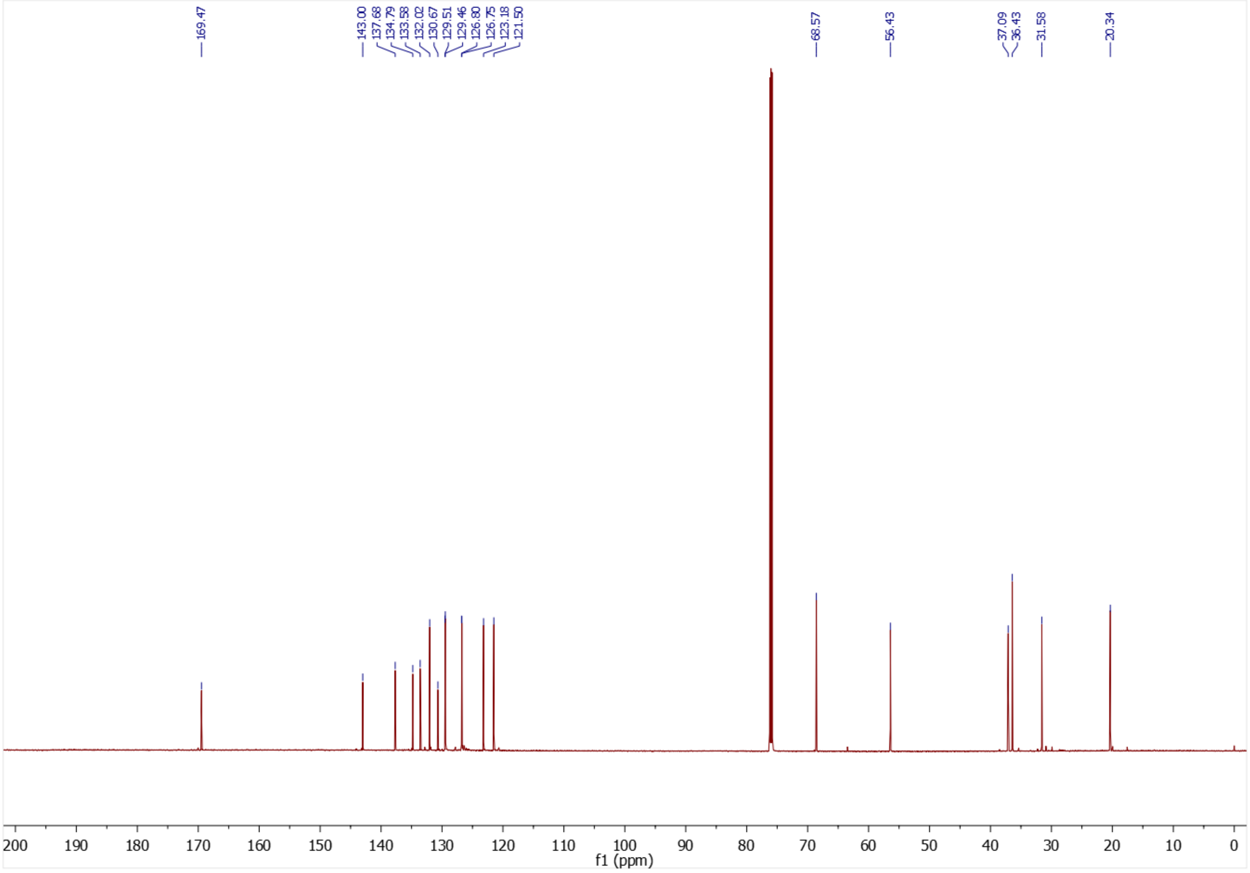

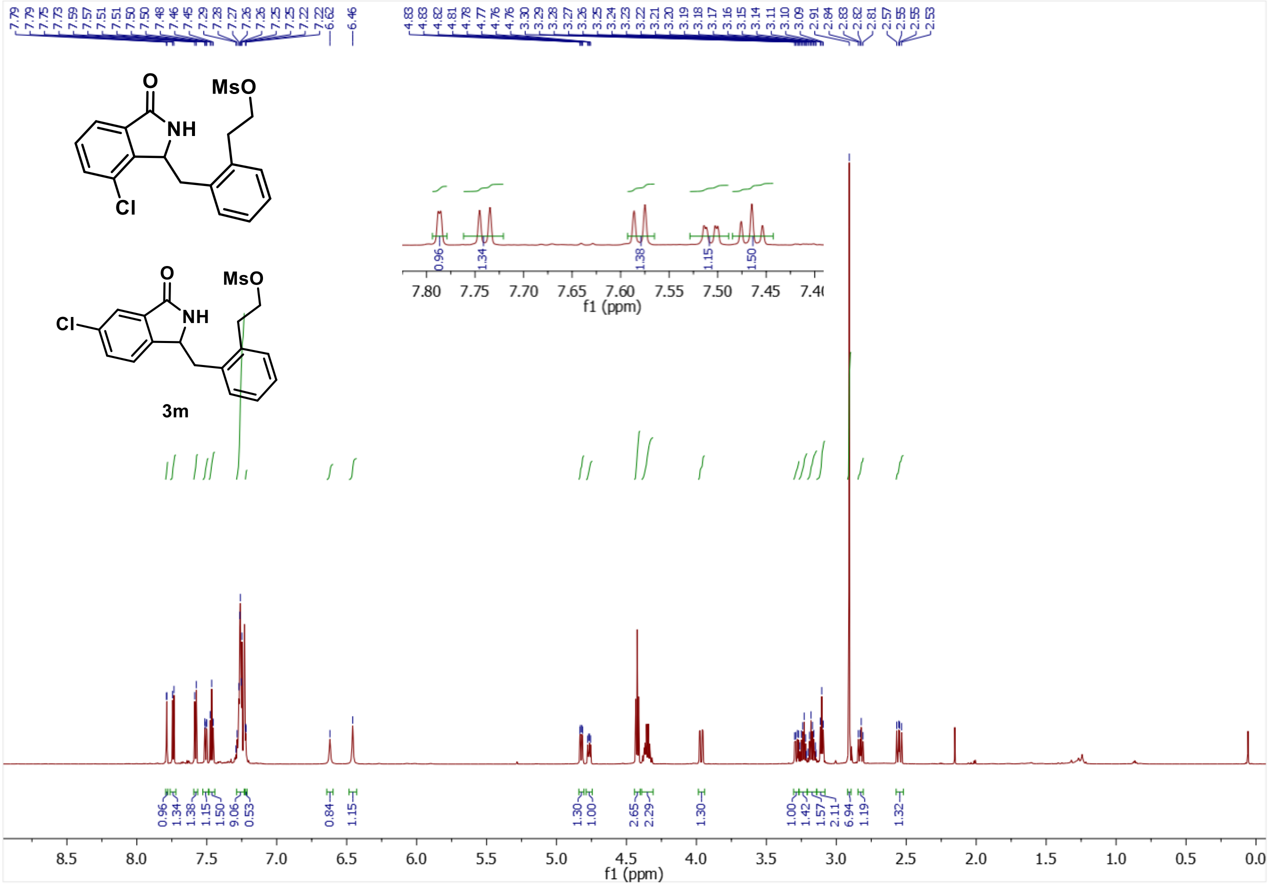
MsO
NH
O
Cl
3m
f1 (ppm)

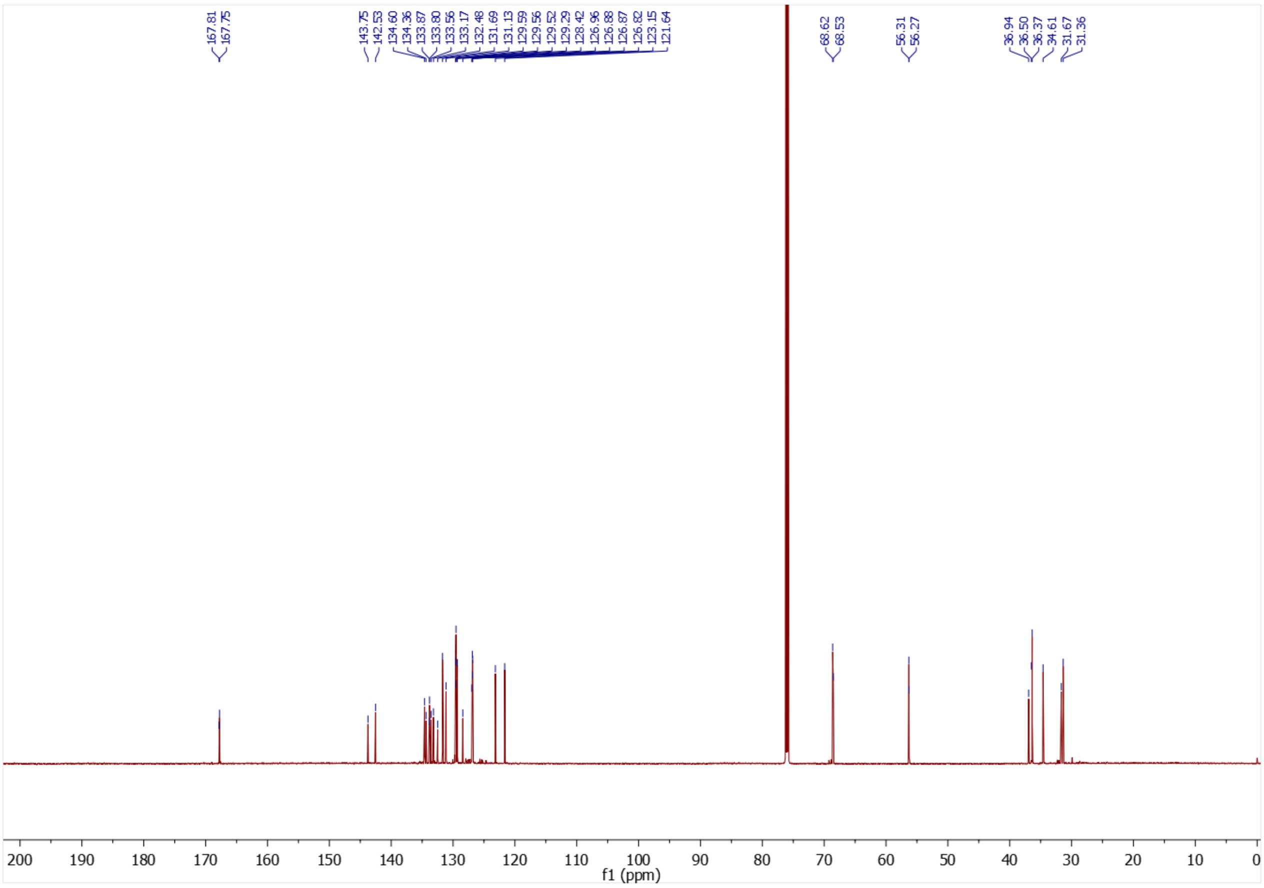
f1 (ppm)

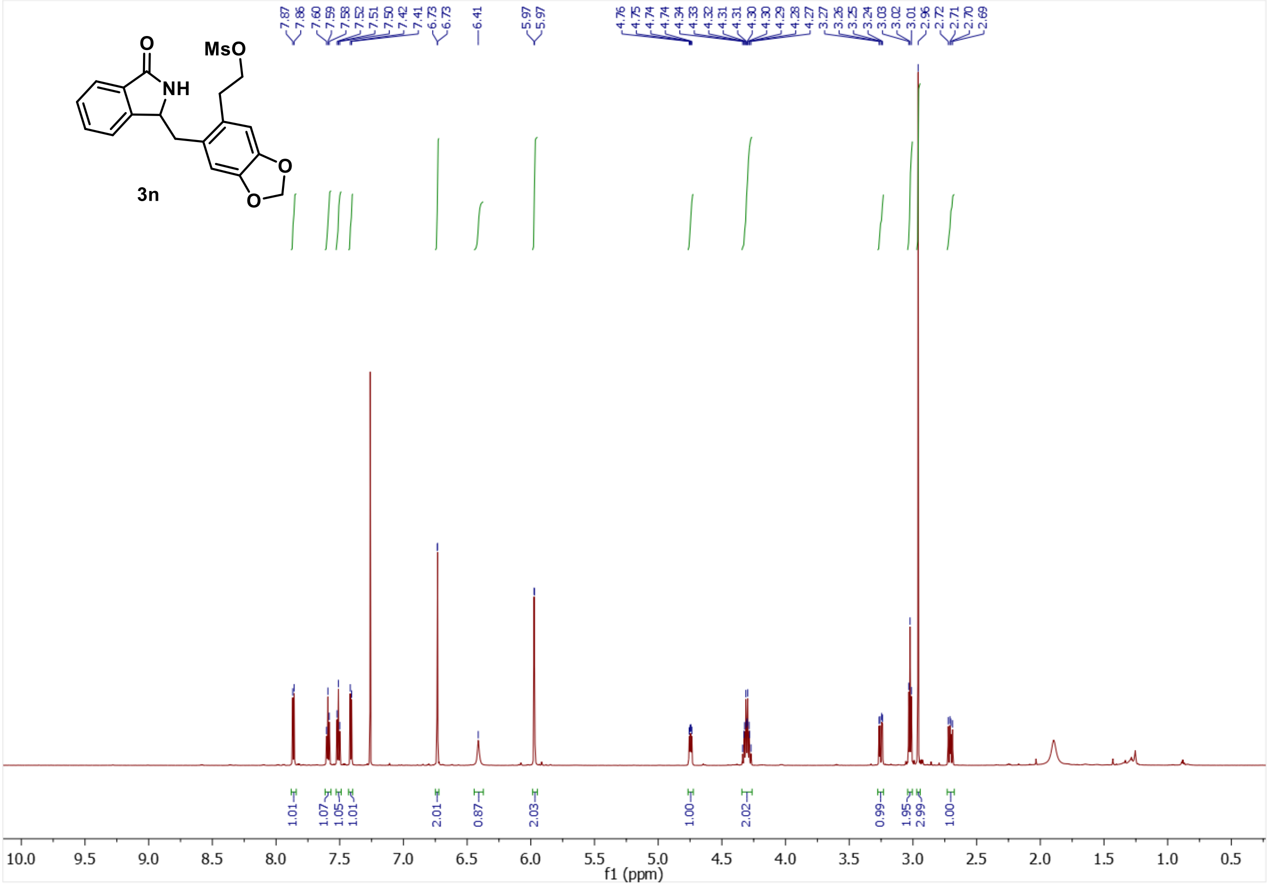
MsO
NH
O
3n
f1 (ppm)

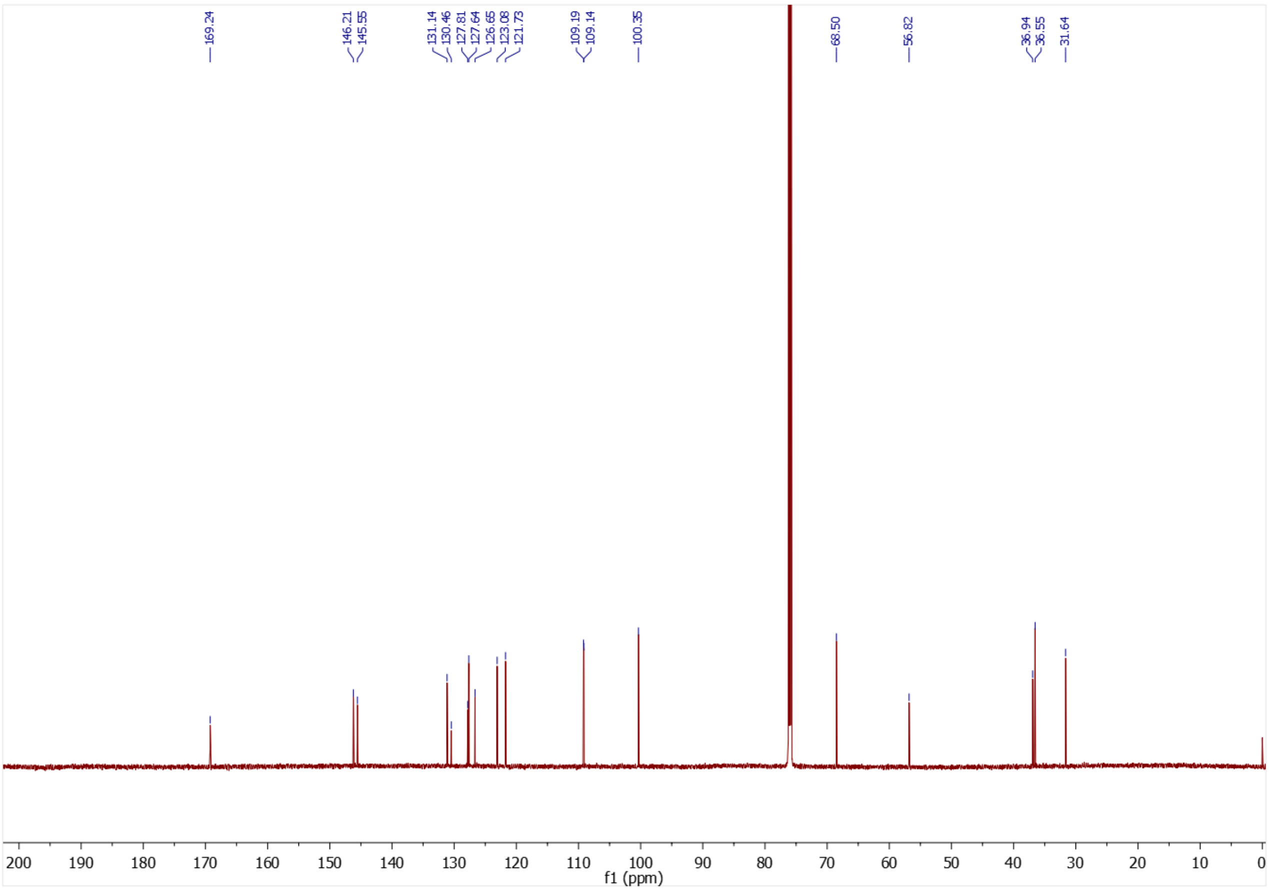
f1 (ppm)

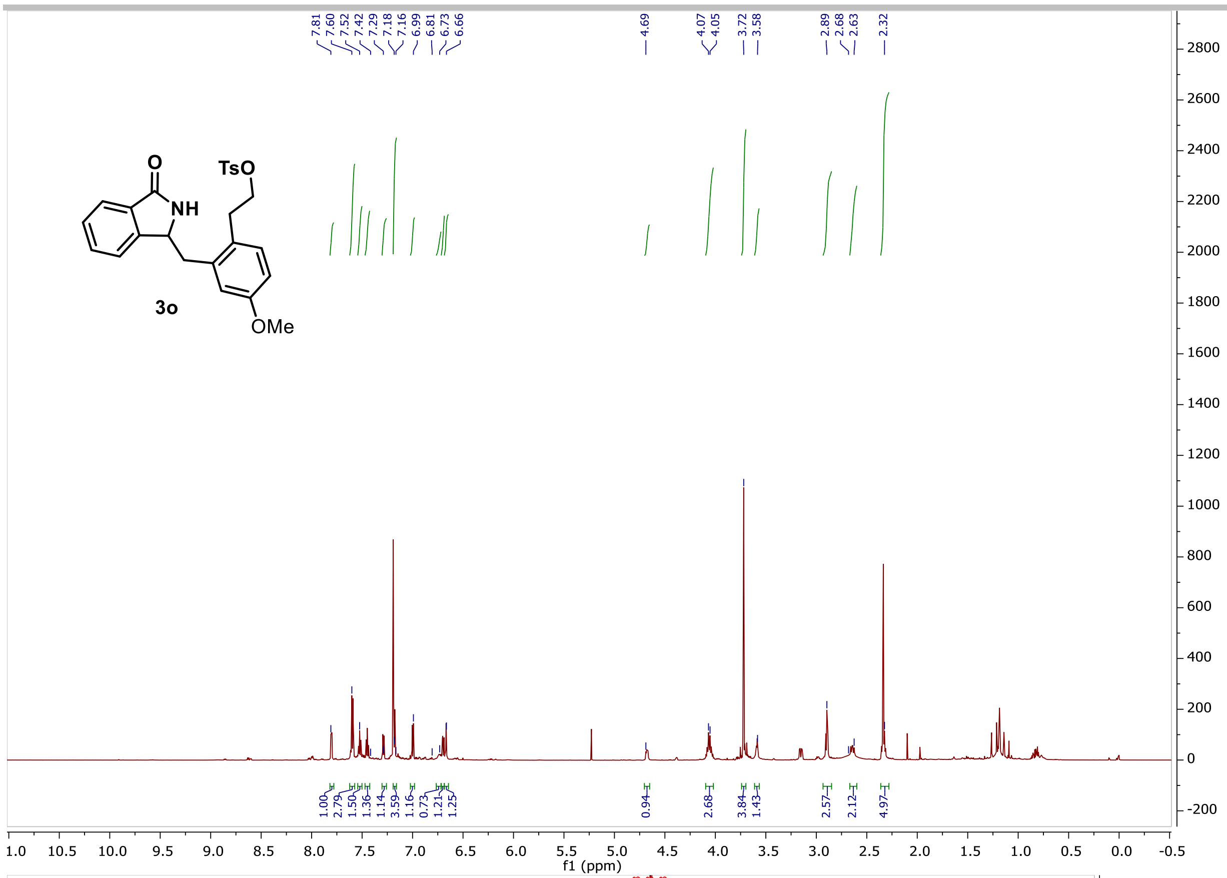
3o
TsO
NH
O
OMe
f1 (ppm)

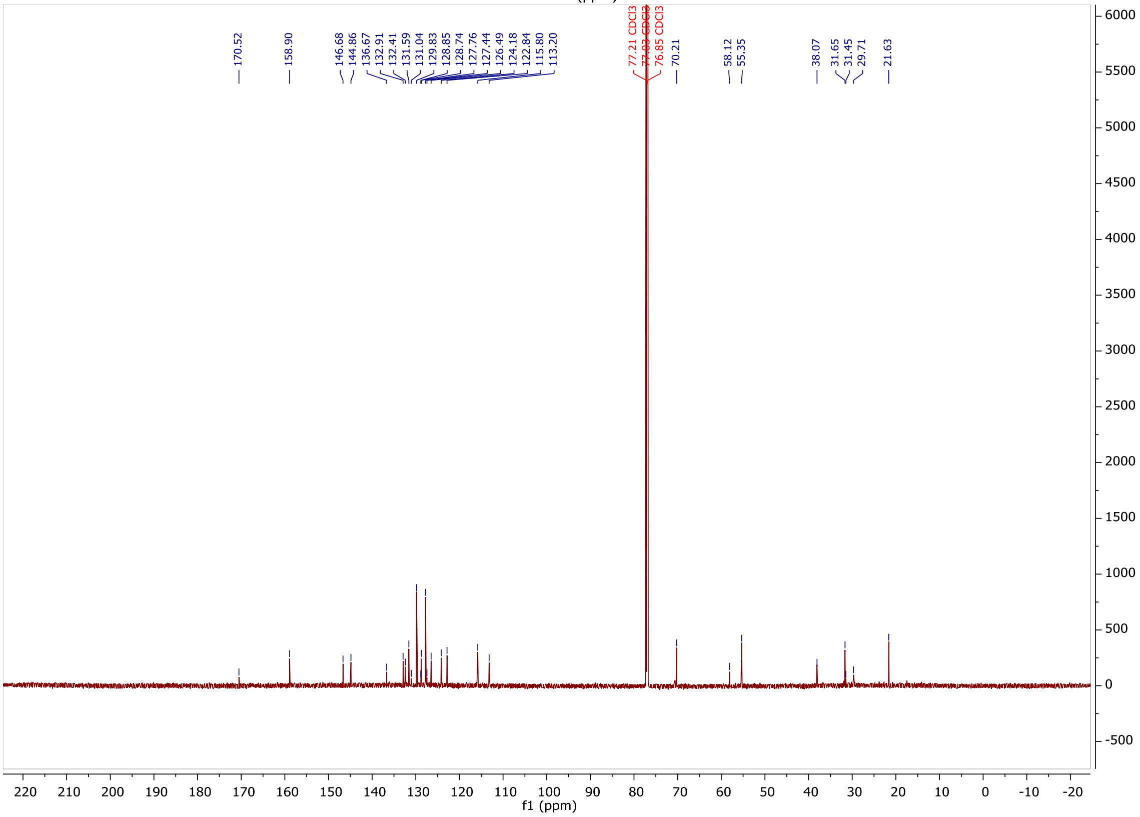
170.52
158.90
146.68
144.86
136.67
132.91
132.41
131.59
131.04
129.83
128.85
128.74
127.76
127.44
126.49
124.18
122.84
115.80
113.20
77.21 CDCl3
76.85 CDCl3
70.21
58.12
55.35
38.07
31.65
31.45
29.71
21.63
f1 (ppm)

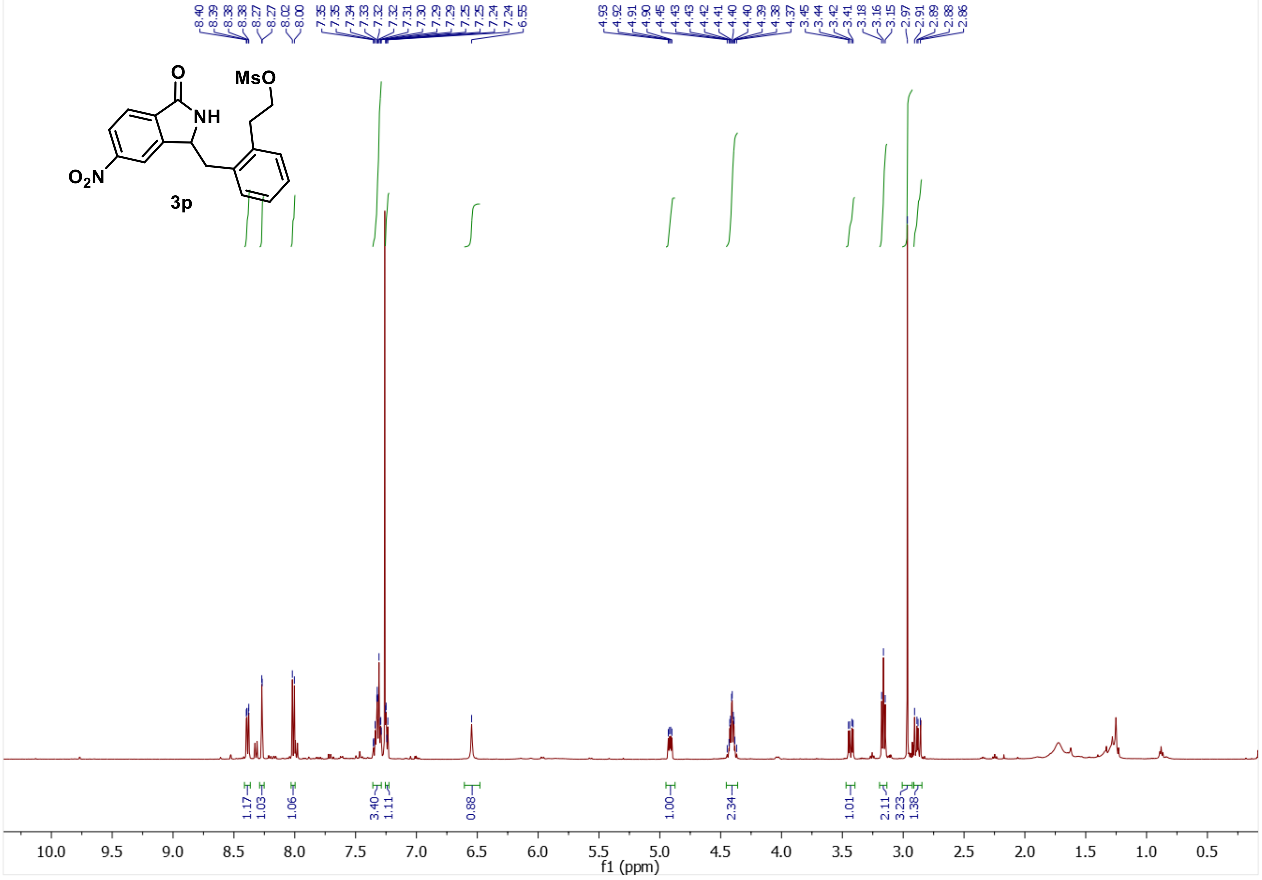
O
MsO
NH
O2N
3p
8.40 8.39 8.38 8.38 8.27 8.27 8.02 8.00 7.35 7.35 7.34 7.33 7.32 7.32 7.31 7.30 7.29 7.29 7.25 7.25 7.24 7.24 6.55
4.93 4.92 4.91 4.90 4.45 4.43 4.43 4.42 4.41 4.40 4.40 4.39 4.38 4.37 3.45 3.44 3.42 3.41 3.18 3.16 3.15 2.97 2.91 2.89 2.88 2.86
1.17 1.03 1.06 3.40 1.11 0.88 1.00 2.34 1.01 2.11 3.23 1.38
f1 (ppm)

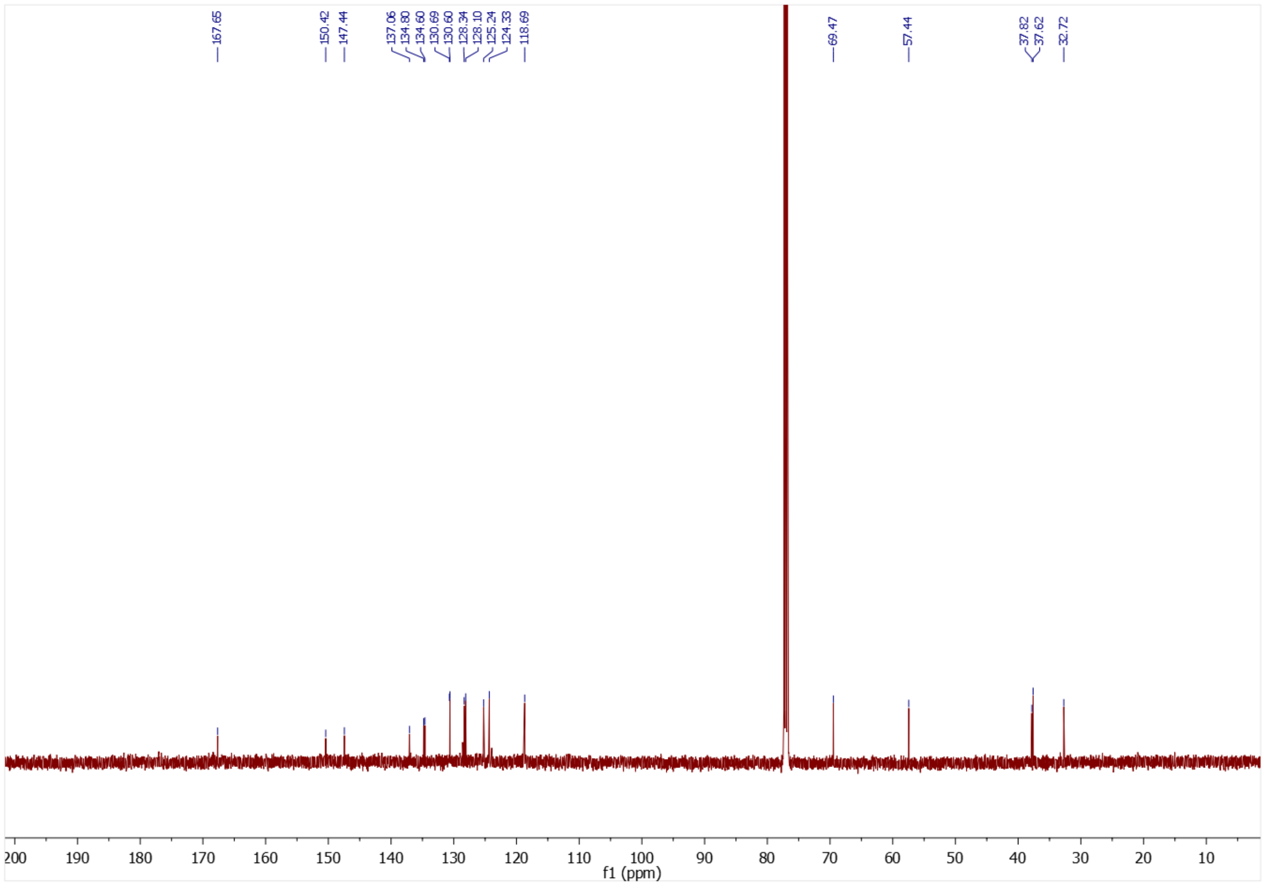
167.65
150.42 147.44
137.06 134.80 134.60 130.69 130.60 128.34 128.10 125.24 124.33 118.69
69.47
57.44
37.82 37.62 32.72
f1 (ppm)

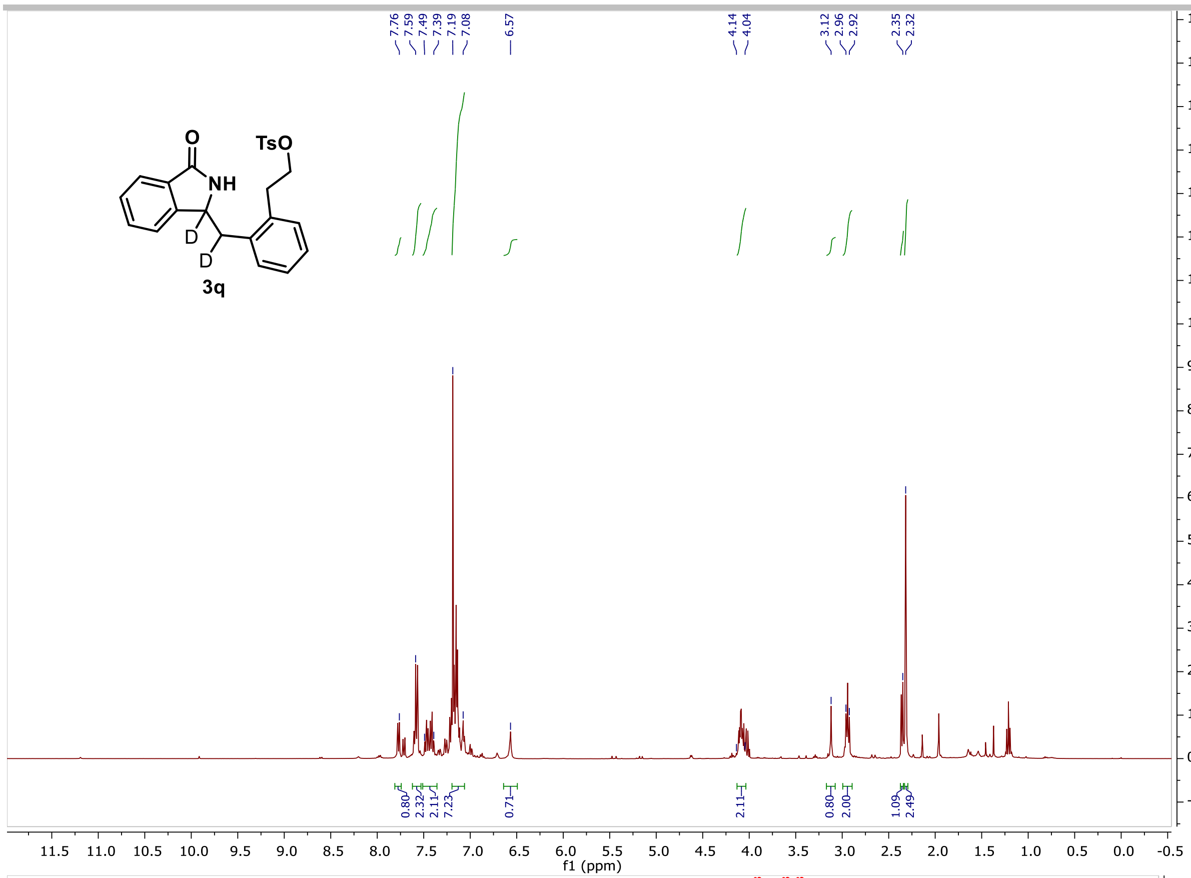

7.76
7.59
7.49
7.39
7.19
7.08
6.57
4.14
4.04
3.12
2.96
2.92
2.35
2.32
O
NH
TsO
D
D
3q
0.80
2.32
2.11
7.23
0.71
2.11
0.80
2.00
1.09
2.49
f1 (ppm)

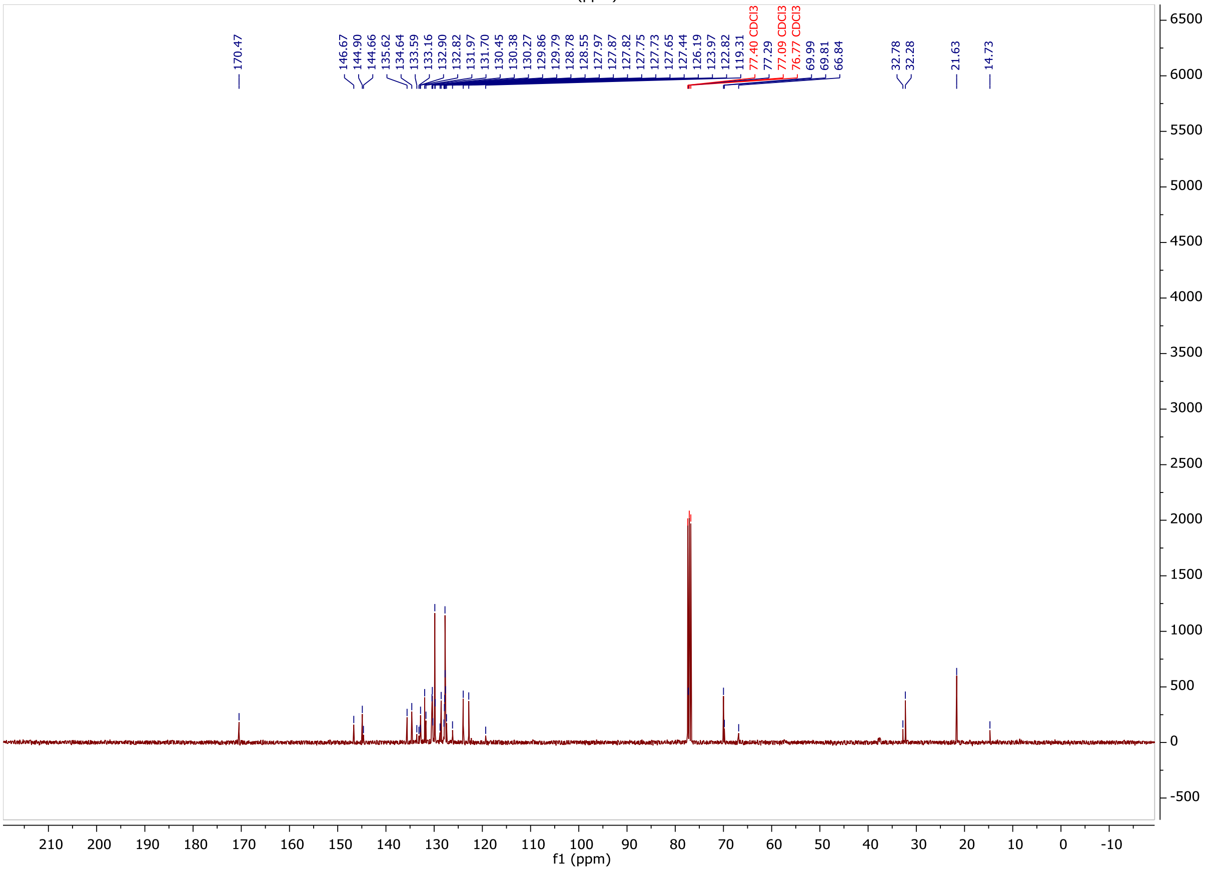

170.47
146.67
144.90
144.66
135.62
134.64
133.59
133.16
132.90
132.82
131.97
131.70
130.45
130.38
130.27
129.86
129.79
128.78
128.55
127.97
127.87
127.82
127.75
127.73
127.65
127.44
126.19
123.97
122.82
119.31
77.40 CDCl3
77.29
77.09 CDCl3
76.77 CDCl3
69.99
69.81
66.84
32.78
32.28
21.63
14.73
f1 (ppm)

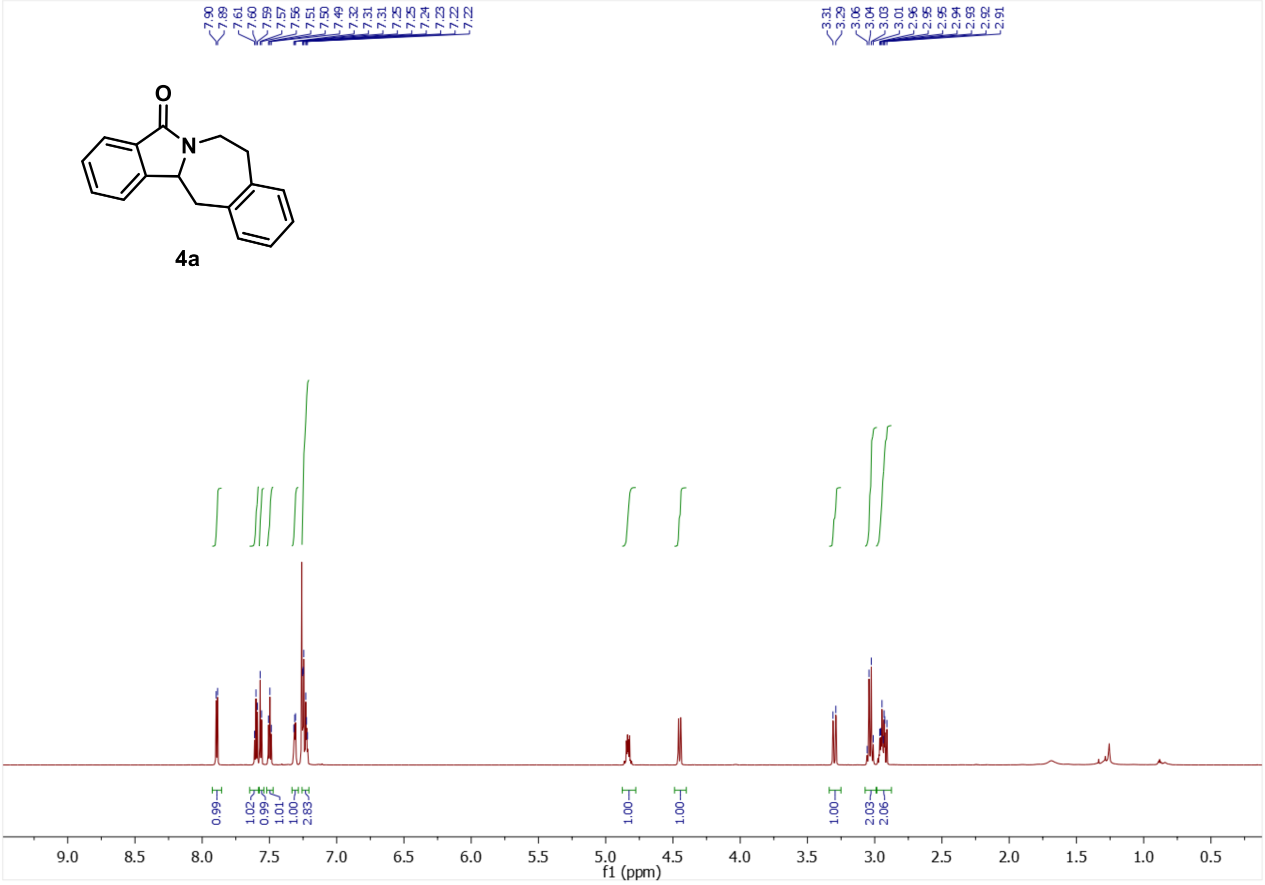
4a
f1 (ppm)

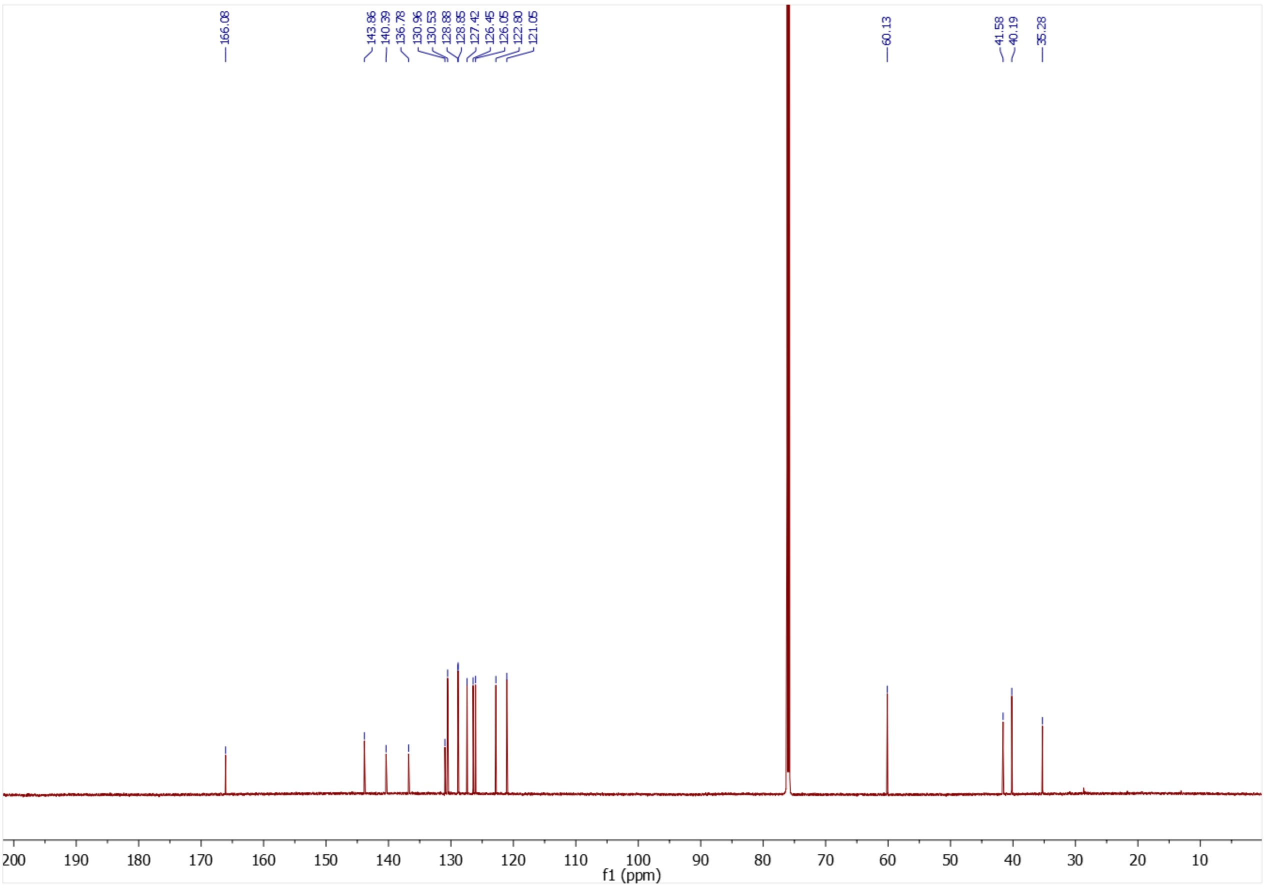
f1 (ppm)

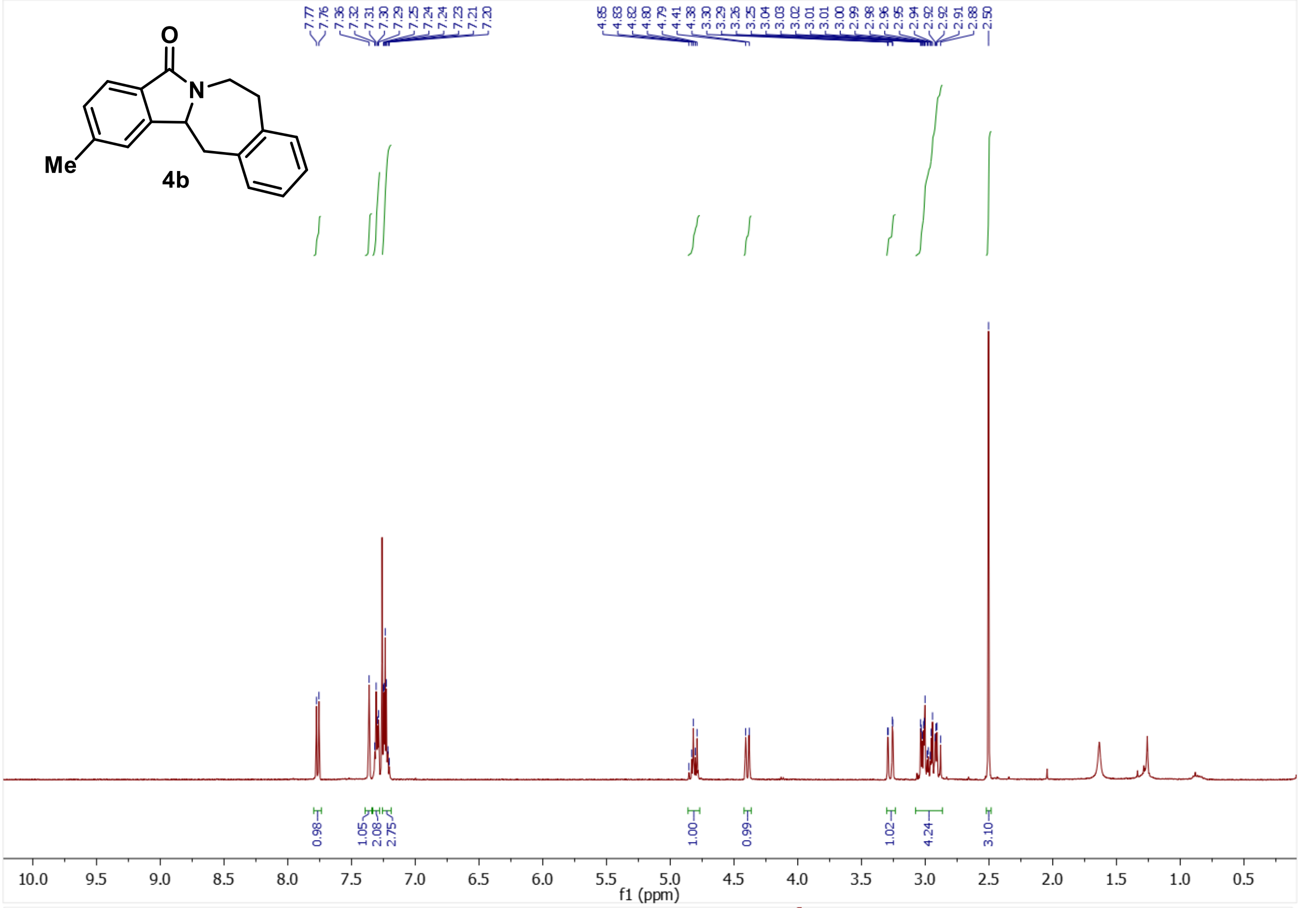
O
N
Me
4b
7.77 7.76 7.36 7.32 7.31 7.30 7.29 7.25 7.24 7.24 7.23 7.21 7.20
4.85 4.83 4.82 4.80 4.79 4.41 4.38 3.30 3.29 3.26 3.25 3.04 3.03 3.02 3.01 3.01 3.00 2.99 2.98 2.96 2.95 2.94 2.92 2.92 2.91 2.88 2.50
0.98 1.05 2.08 2.75 1.00 0.99 1.02 4.24 3.10
10.0 9.5 9.0 8.5 8.0 7.5 7.0 6.5 6.0 5.5 5.0 4.5 4.0 3.5 3.0 2.5 2.0 1.5 1.0 0.5
f1 (ppm)

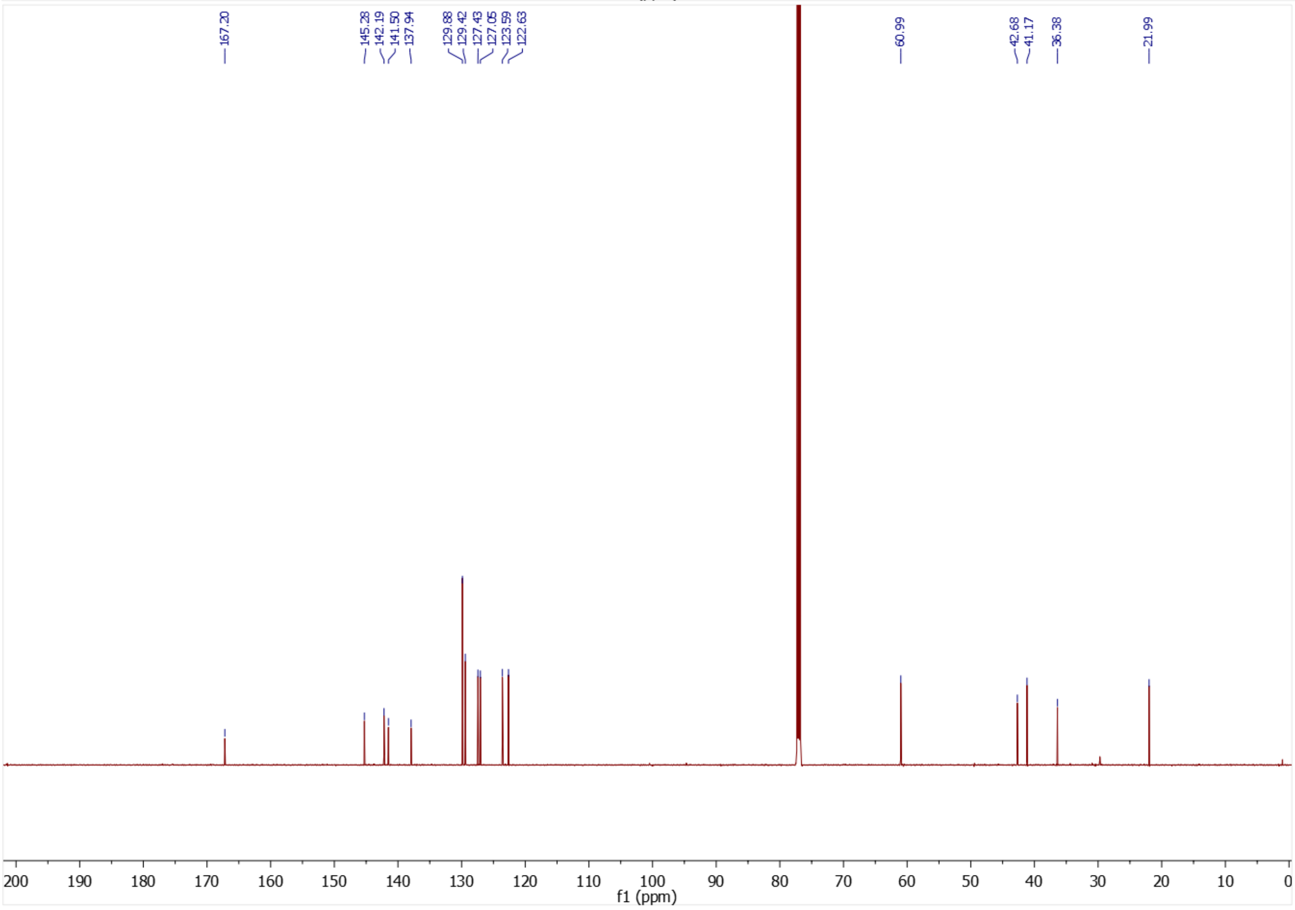
167.20
145.28 142.19 141.50 137.94
129.88 129.42 127.43 127.05 123.59 122.63
60.99
42.68 41.17 36.38
21.99
200 190 180 170 160 150 140 130 120 110 100 90 80 70 60 50 40 30 20 10 0
f1 (ppm)

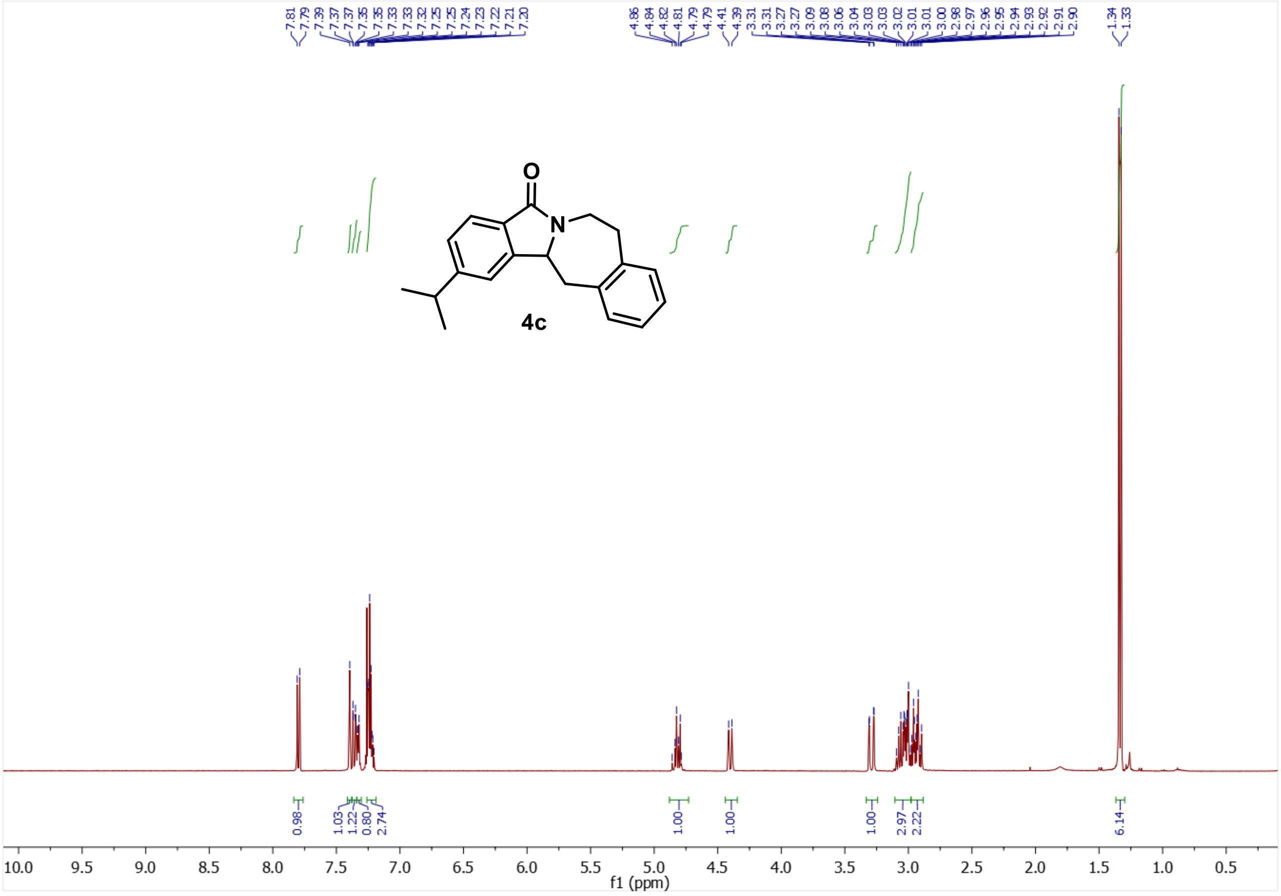
7.81
7.79
7.39
7.37
7.37
7.35
7.35
7.33
7.33
7.32
7.25
7.25
7.24
7.23
7.22
7.21
7.20
4.86
4.84
4.82
4.81
4.79
4.79
4.41
4.39
3.31
3.31
3.27
3.27
3.09
3.08
3.06
3.04
3.04
3.03
3.03
3.02
3.01
3.01
3.00
2.98
2.97
2.96
2.95
2.94
2.93
2.92
2.91
2.90
1.34
1.33
O
N
4c
0.98
1.03
1.22
0.80
2.74
1.00
1.00
1.00
2.97
2.22
6.14
10.0 9.5 9.0 8.5 8.0 7.5 7.0 6.5 6.0 5.5 5.0 4.5 4.0 3.5 3.0 2.5 2.0 1.5 1.0 0.5
f1 (ppm)

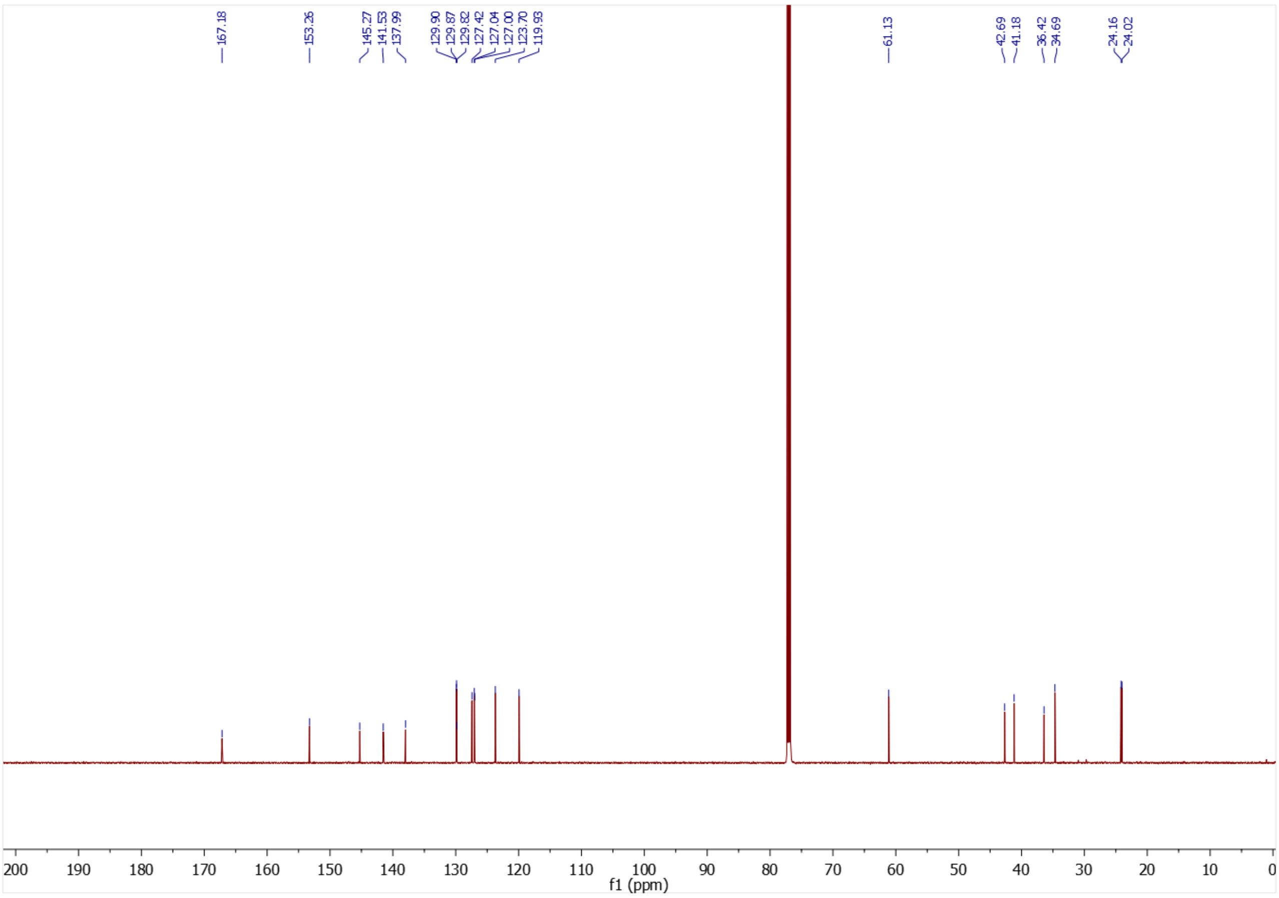
167.18
153.26
145.27
141.53
137.99
129.90
129.87
129.82
127.42
127.04
127.00
123.70
119.93
61.13
42.69
41.18
36.42
34.69
24.16
24.02
200 190 180 170 160 150 140 130 120 110 100 90 80 70 60 50 40 30 20 10 0
f1 (ppm)

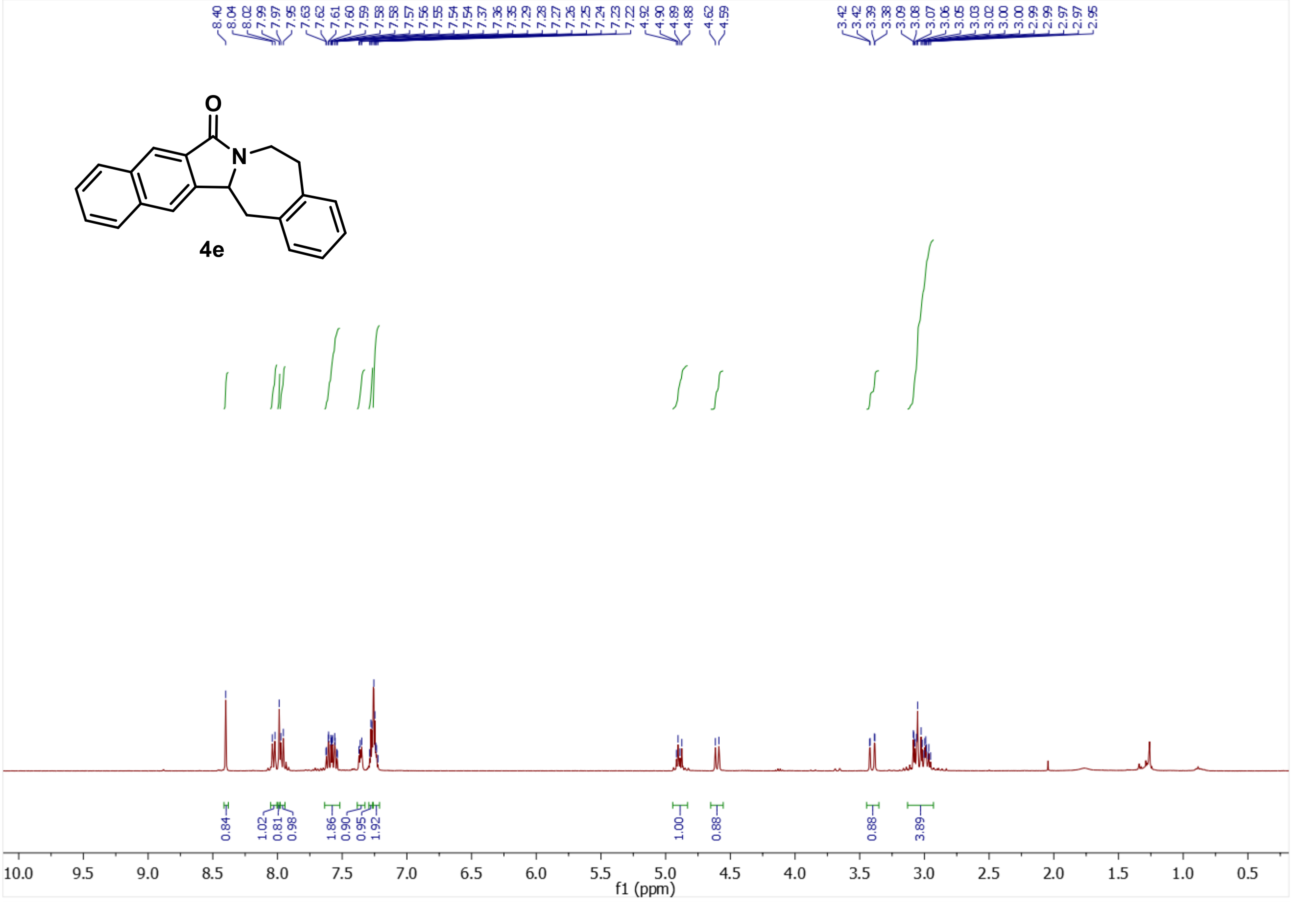
4e

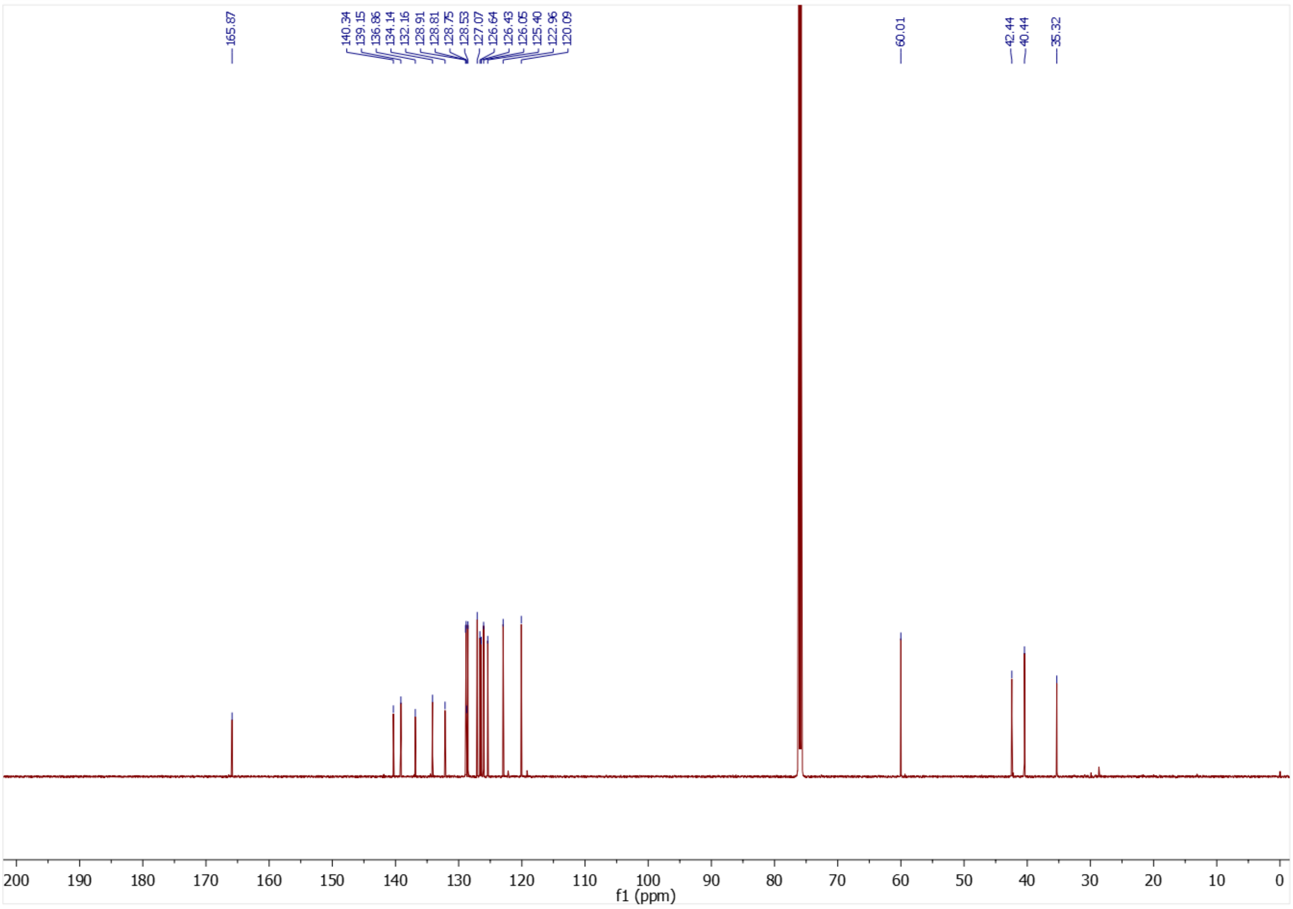

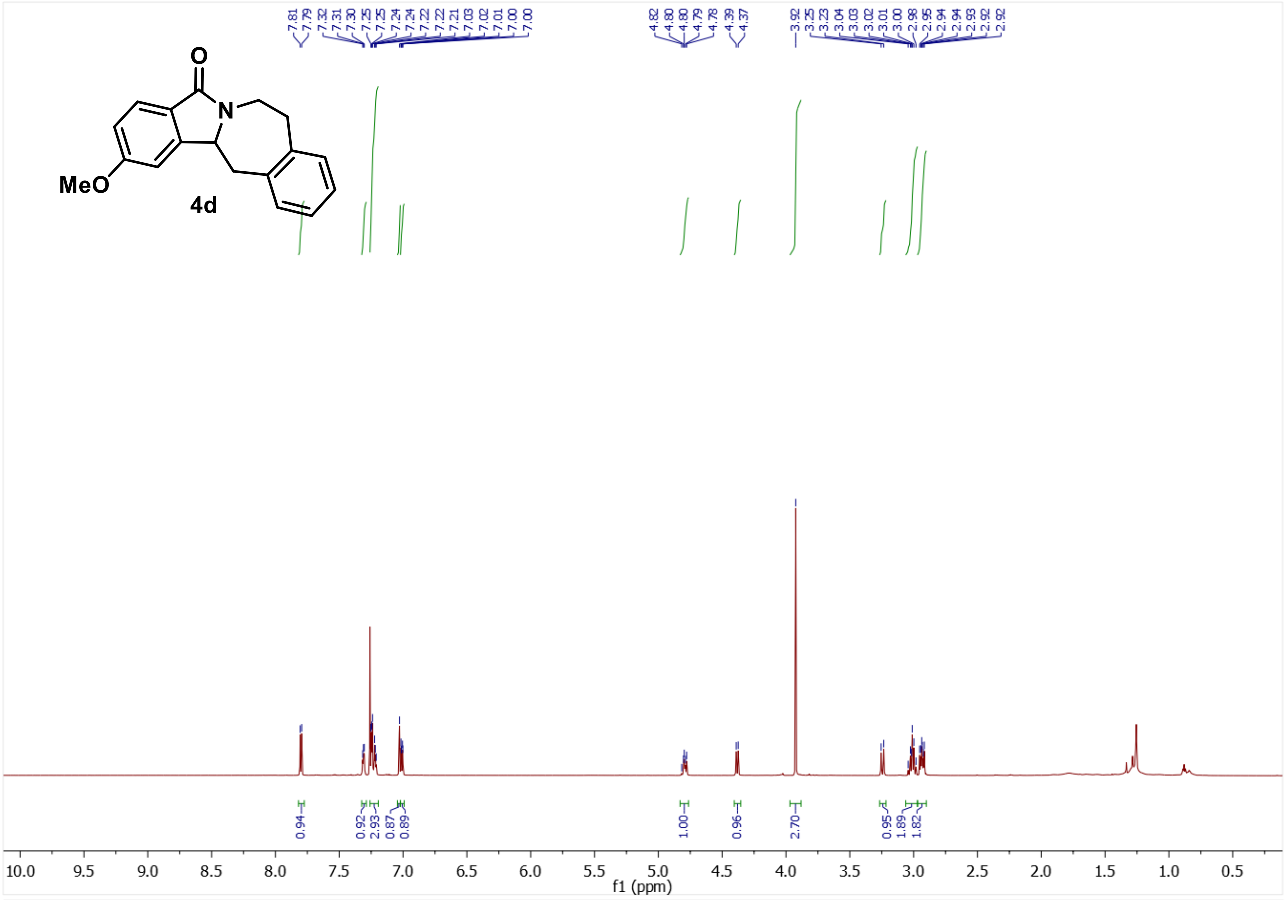
O
N
MeO
4d
7.81 7.79 7.32 7.31 7.30 7.25 7.25 7.24 7.24 7.22 7.22 7.21 7.03 7.02 7.01 7.00 7.00
4.82 4.80 4.80 4.79 4.78 4.39 4.37
3.92 3.25 3.23 3.04 3.03 3.02 3.01 3.00 2.98 2.95 2.94 2.94 2.93 2.92 2.92
0.94 0.92 2.93 0.87 0.89 1.00 0.96 2.70 0.95 1.89 1.82
10.0 9.5 9.0 8.5 8.0 7.5 7.0 6.5 6.0 5.5 5.0 4.5 4.0 3.5 3.0 2.5 2.0 1.5 1.0 0.5
f1 (ppm)

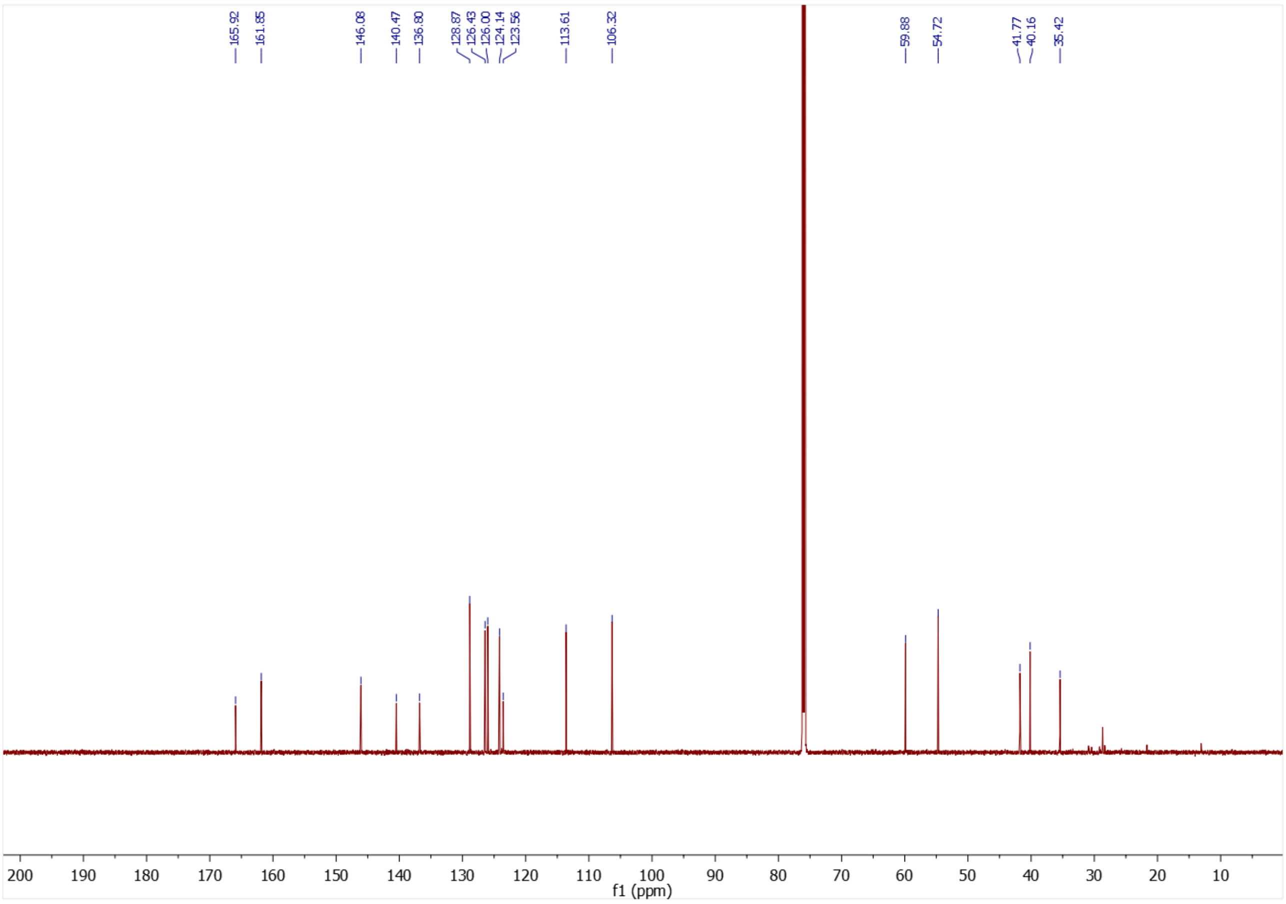
165.92 161.85 146.08 140.47 136.80 128.87 126.43 126.00 124.14 123.56 113.61 106.32 59.88 54.72 41.77 40.16 35.42
200 190 180 170 160 150 140 130 120 110 100 90 80 70 60 50 40 30 20 10
f1 (ppm)

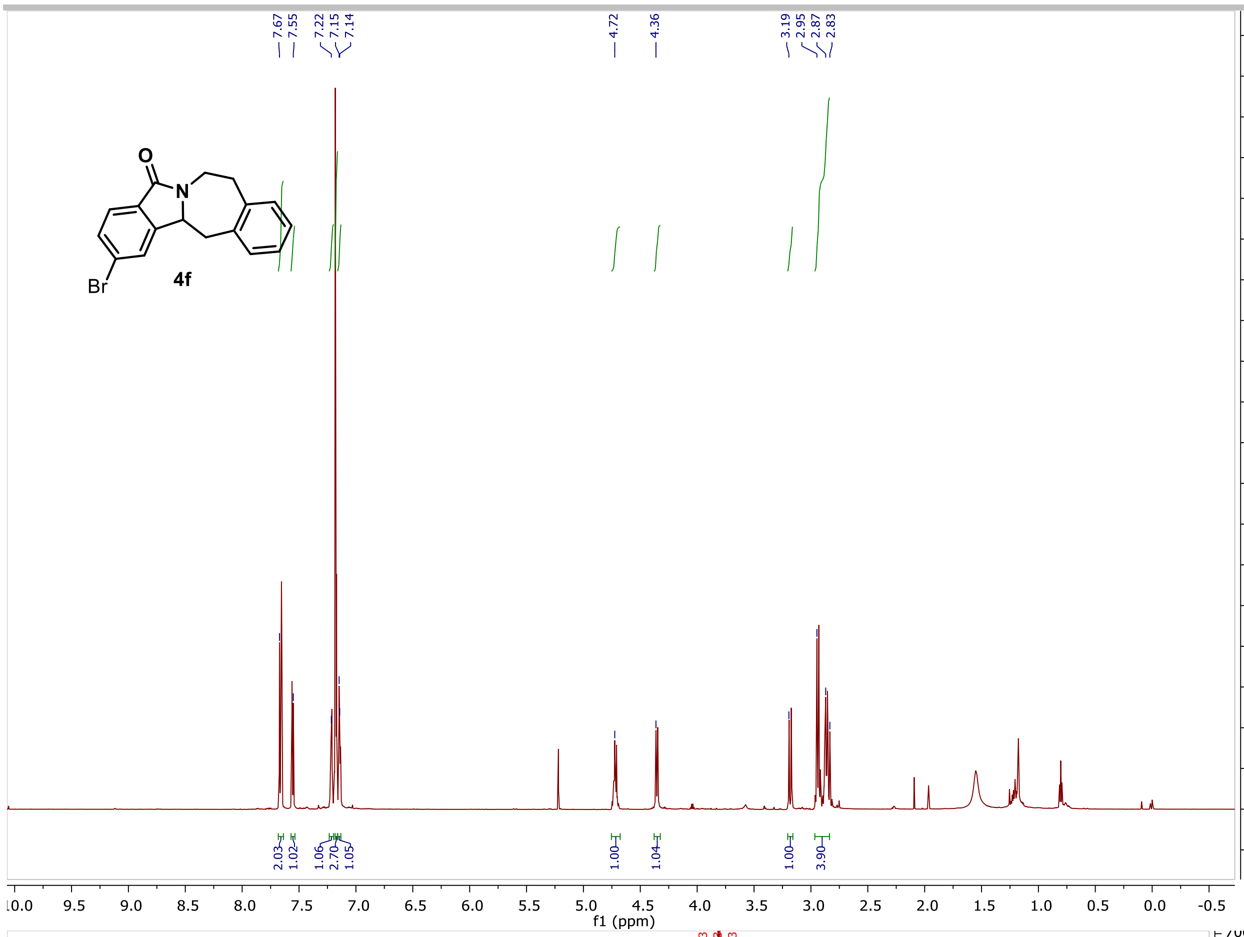
7.67
7.55
7.22
7.15
7.14
4.72
4.36
3.19
2.95
2.87
2.83
O
N
Br
4f
2.03
1.02
1.06
2.70
1.05
1.00
1.04
1.00
3.90
f1 (ppm)

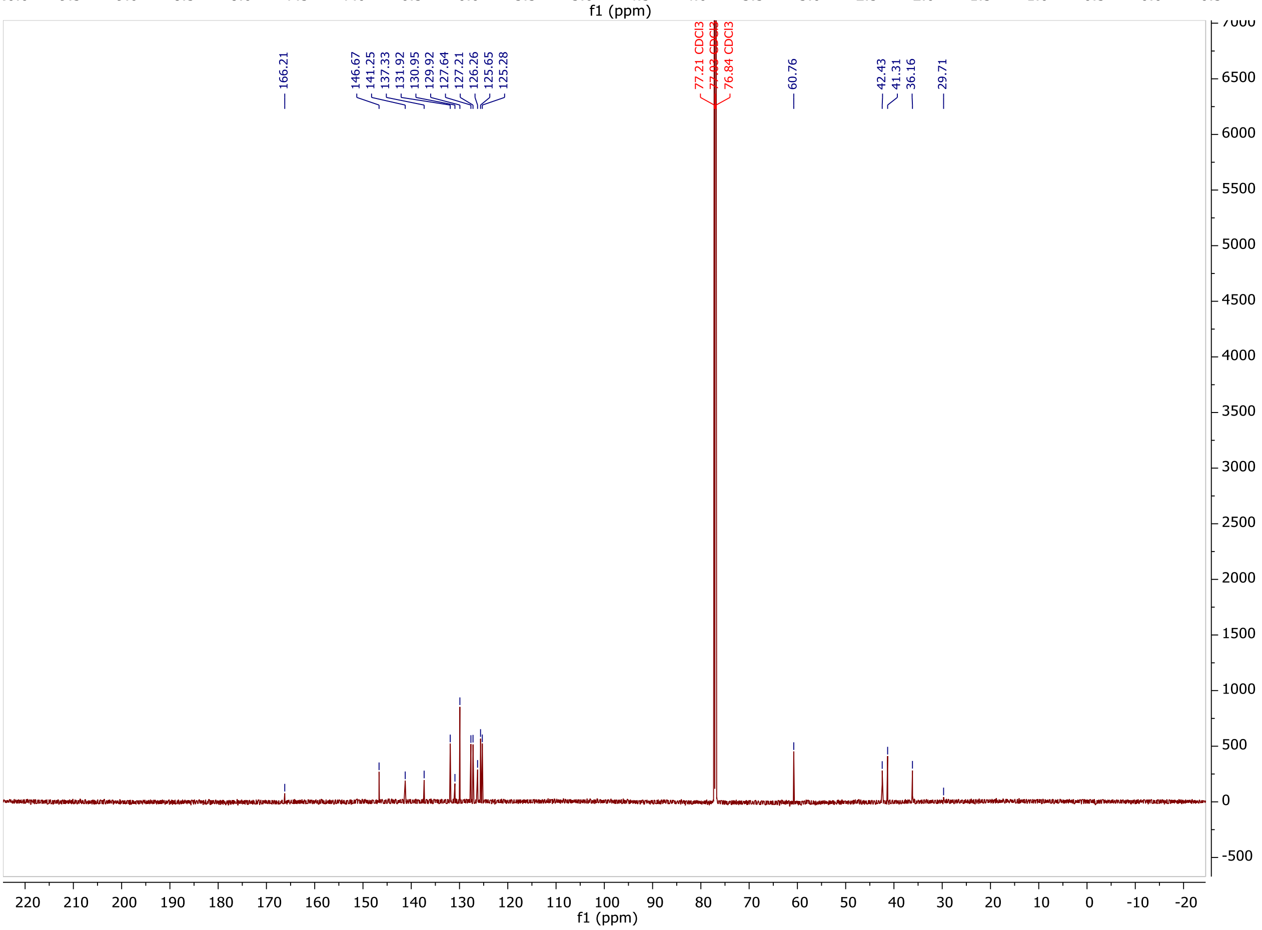
166.21
146.67
141.25
137.33
131.92
130.95
129.92
127.64
127.21
126.26
125.65
125.28
77.21 CDCl3
76.84 CDCl3
60.76
42.43
41.31
36.16
29.71
f1 (ppm)

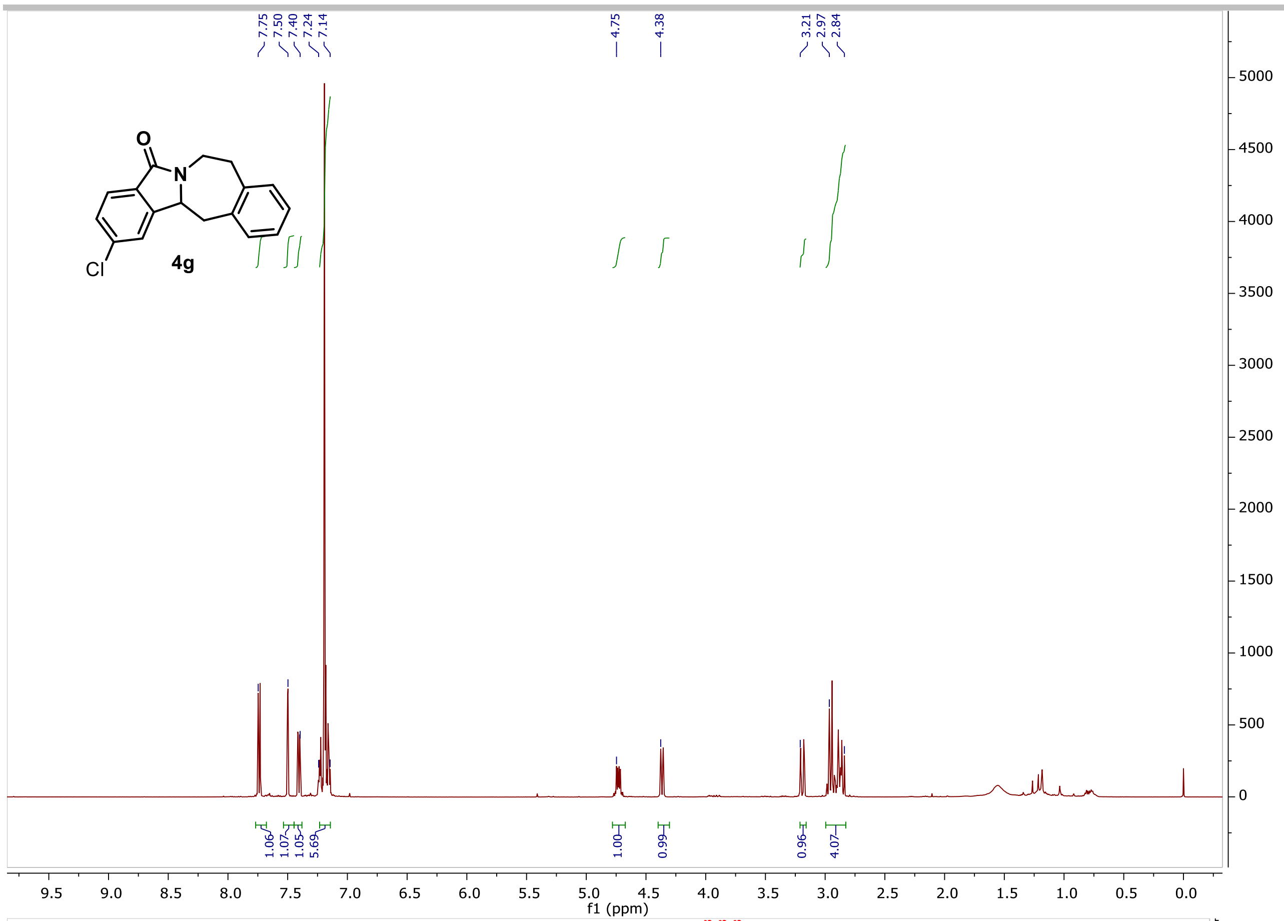
7.75
7.50
7.40
7.24
7.14
4.75
4.38
3.21
2.97
2.84
O
N
Cl
4g
1.06
1.07
1.05
5.69
1.00
0.99
0.96
4.07
f1 (ppm)

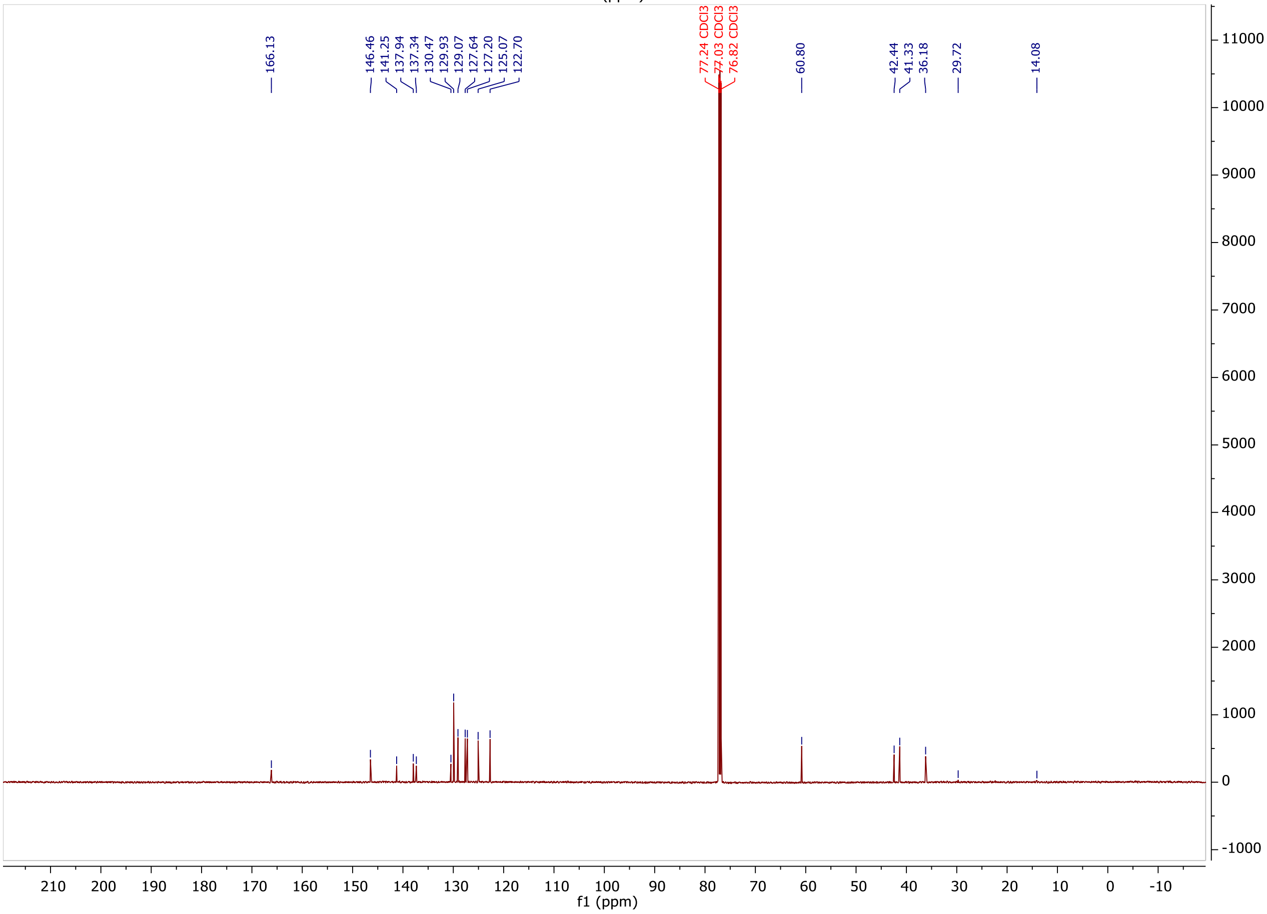
166.13
146.46
141.25
137.94
137.34
130.47
129.93
129.07
127.64
127.20
125.07
122.70
77.24 CDCl3
77.03 CDCl3
76.82 CDCl3
60.80
42.44
41.33
36.18
29.72
14.08
f1 (ppm)

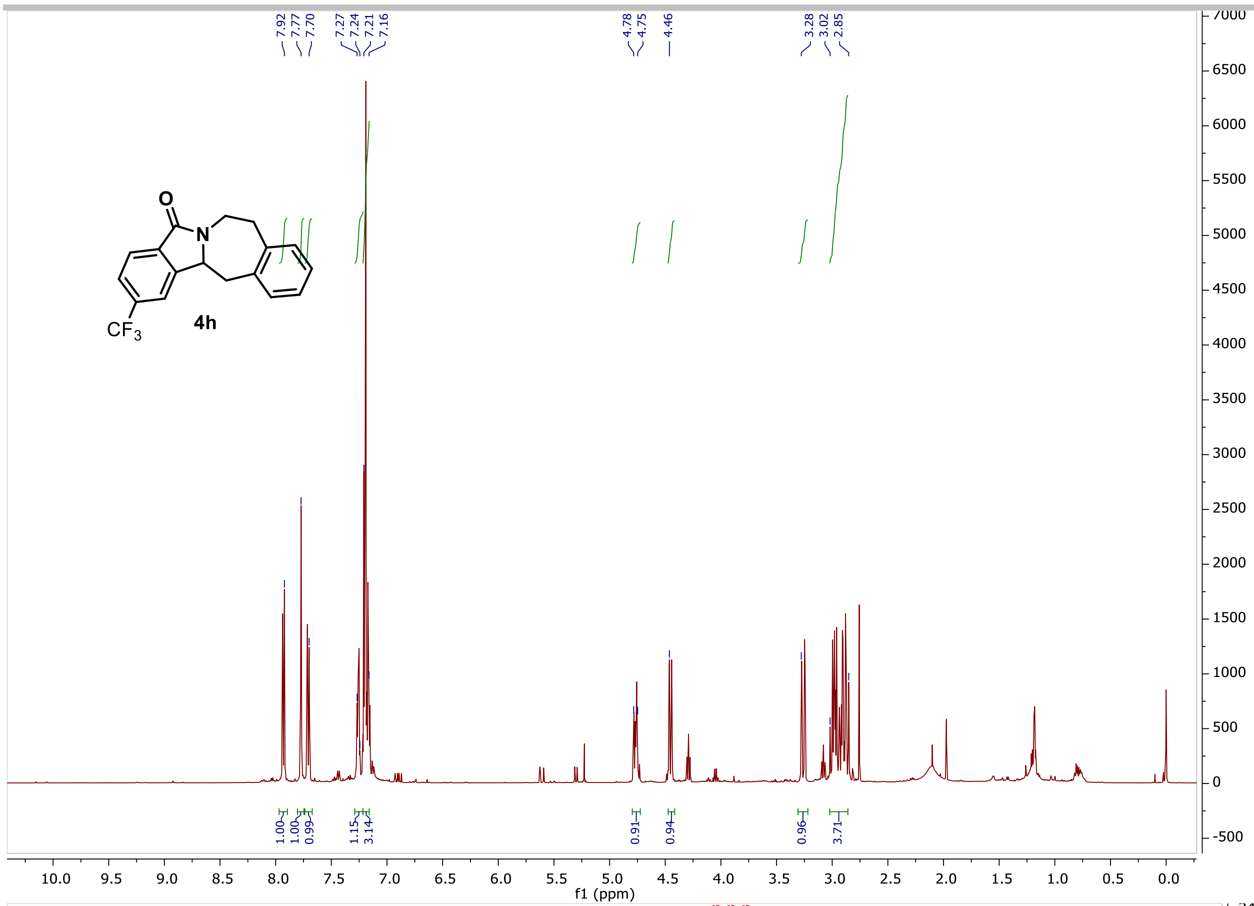
7.92
7.77
7.70
7.27
7.24
7.21
7.16
4.78
4.75
4.46
3.28
3.02
2.85
O
N
CF3
4h
1.00
1.00
0.99
1.15
3.14
0.91
0.94
0.96
3.71
f1 (ppm)

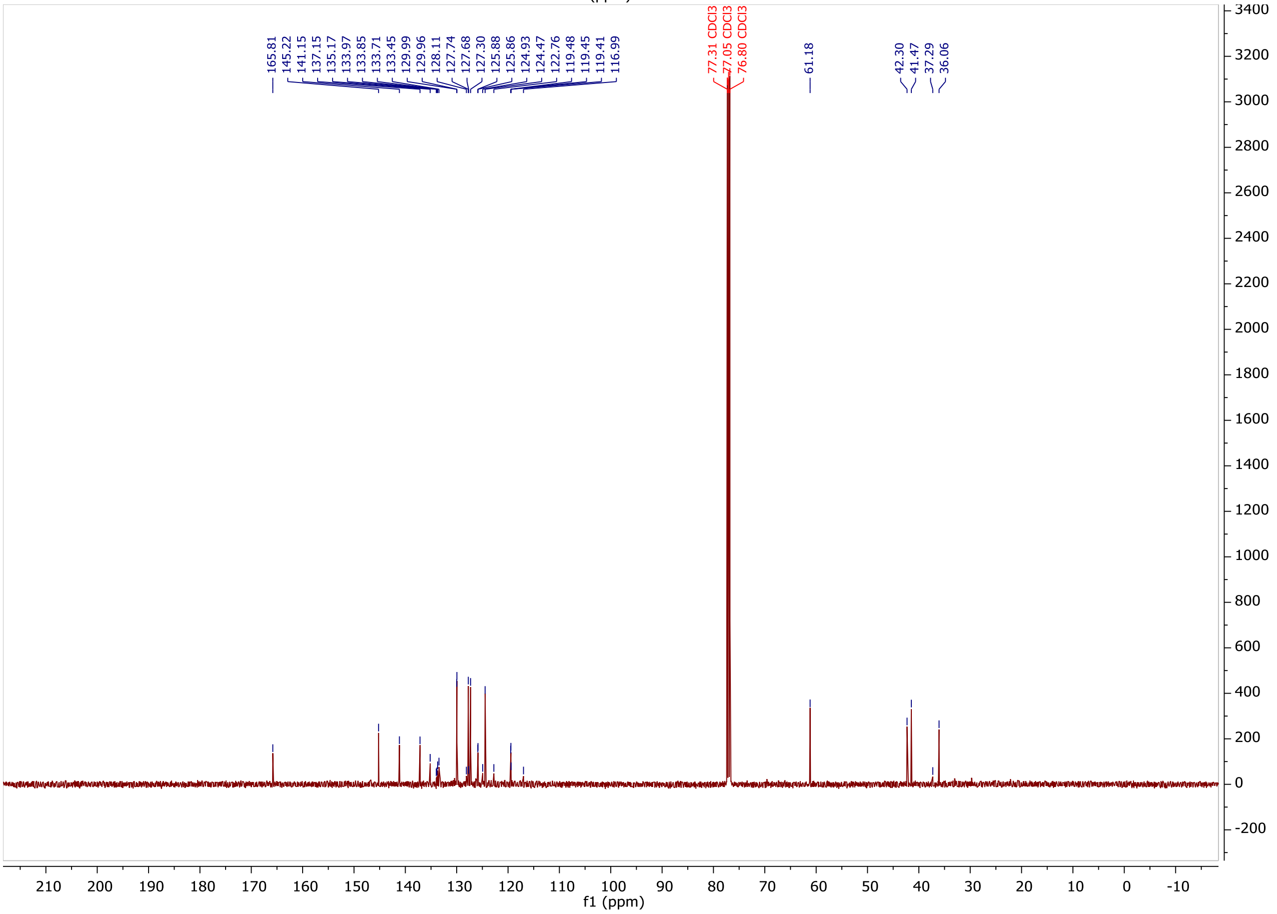
165.81
145.22
141.15
137.15
135.17
133.97
133.85
133.71
133.45
129.99
129.96
128.11
127.74
127.68
127.30
125.88
125.86
124.93
124.47
122.76
119.48
119.45
119.41
116.99
77.31 CDCl3
77.05 CDCl3
76.80 CDCl3
61.18
42.30
41.47
37.29
36.06
f1 (ppm)

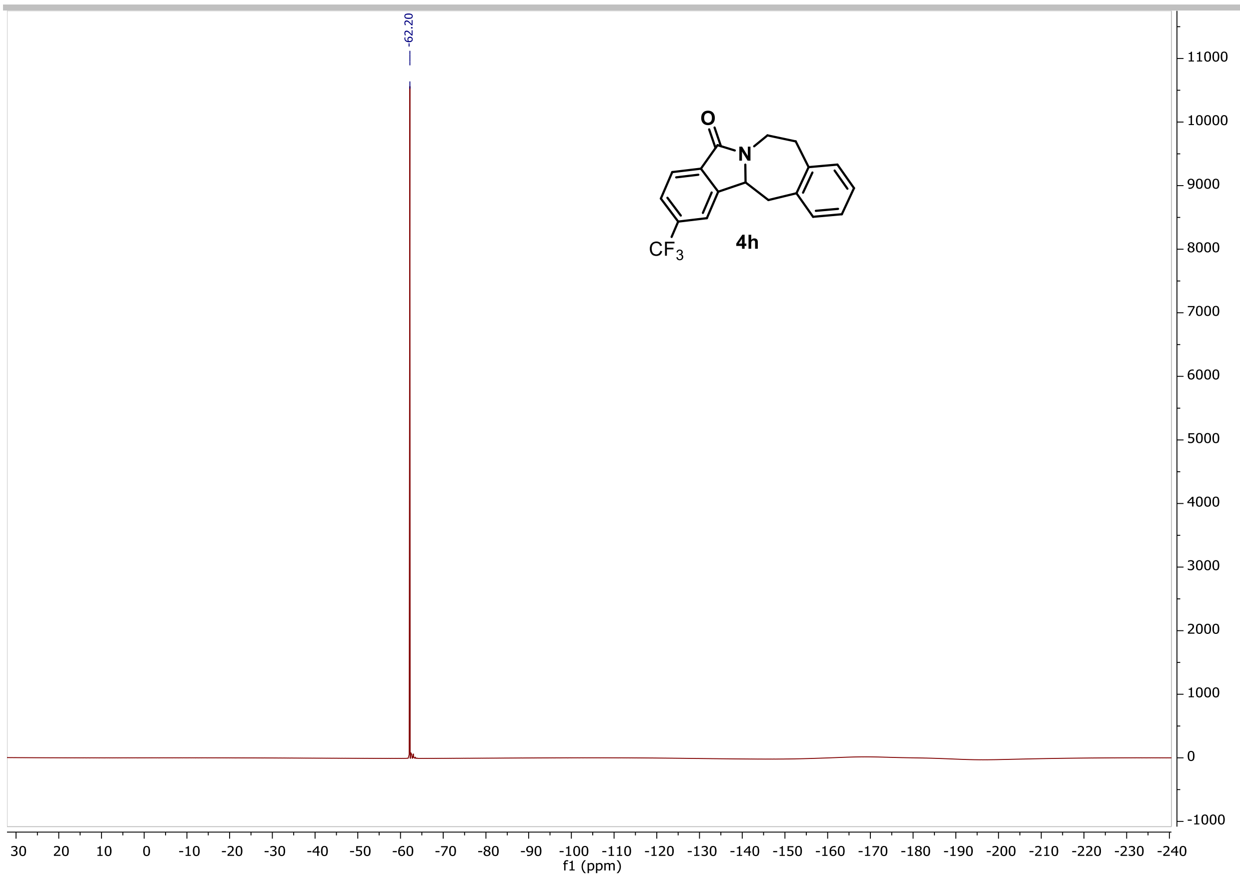
-62.20
O
N
CF3
4h
11000
10000
9000
8000
7000
6000
5000
4000
3000
2000
1000
0
-1000
30 20 10 0 -10 -20 -30 -40 -50 -60 -70 -80 -90 -100 -110 -120 -130 -140 -150 -160 -170 -180 -190 -200 -210 -220 -230 -240
f1 (ppm)

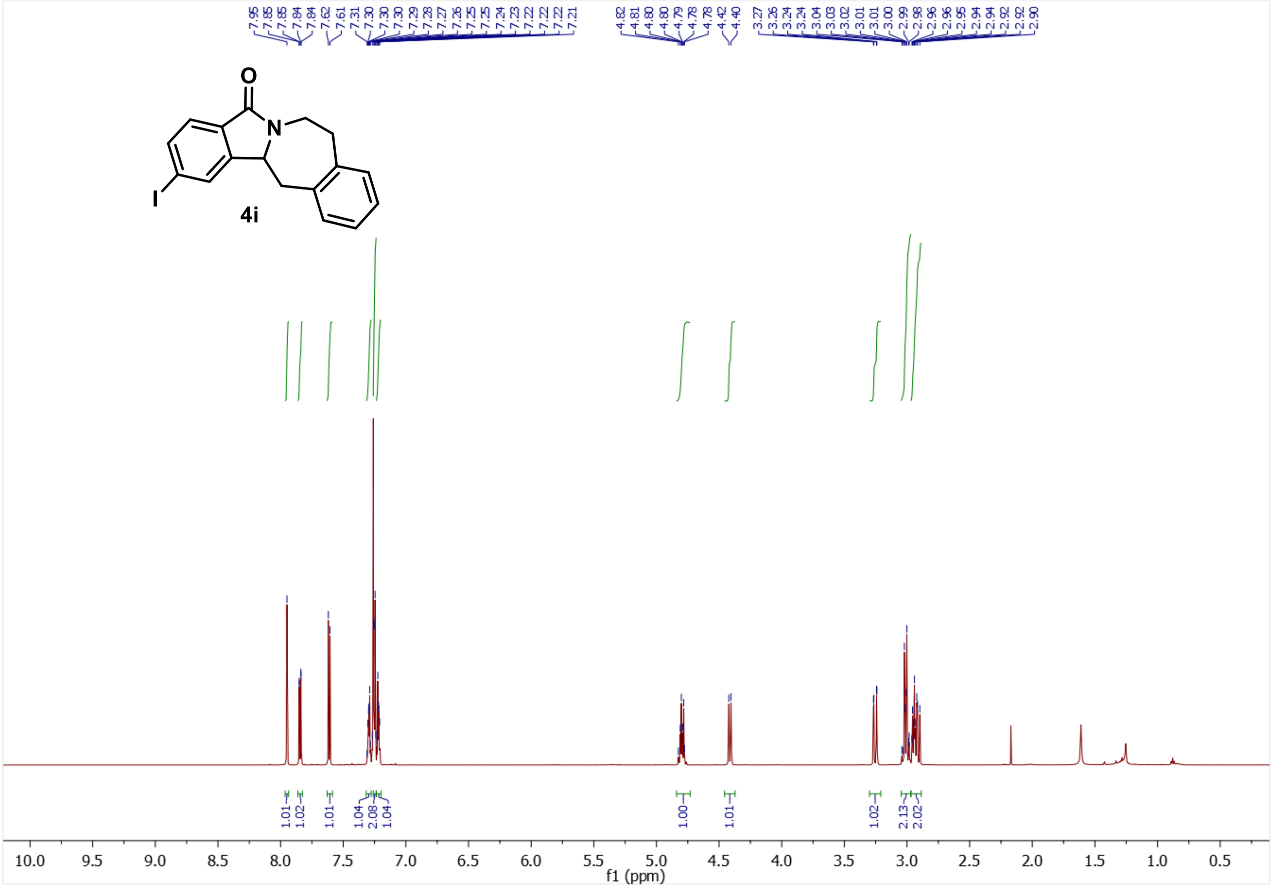
O
N
I
4i
f1 (ppm)

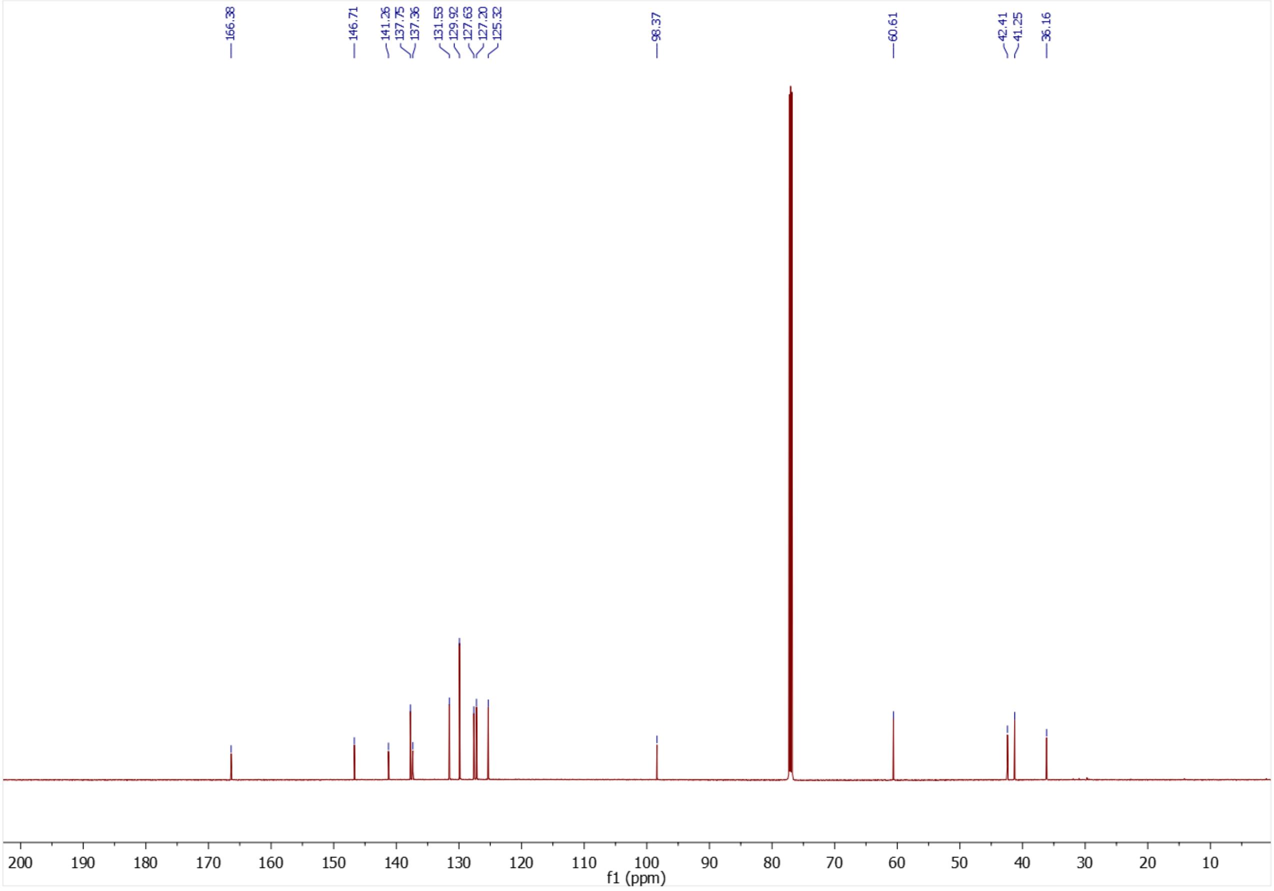
f1 (ppm)

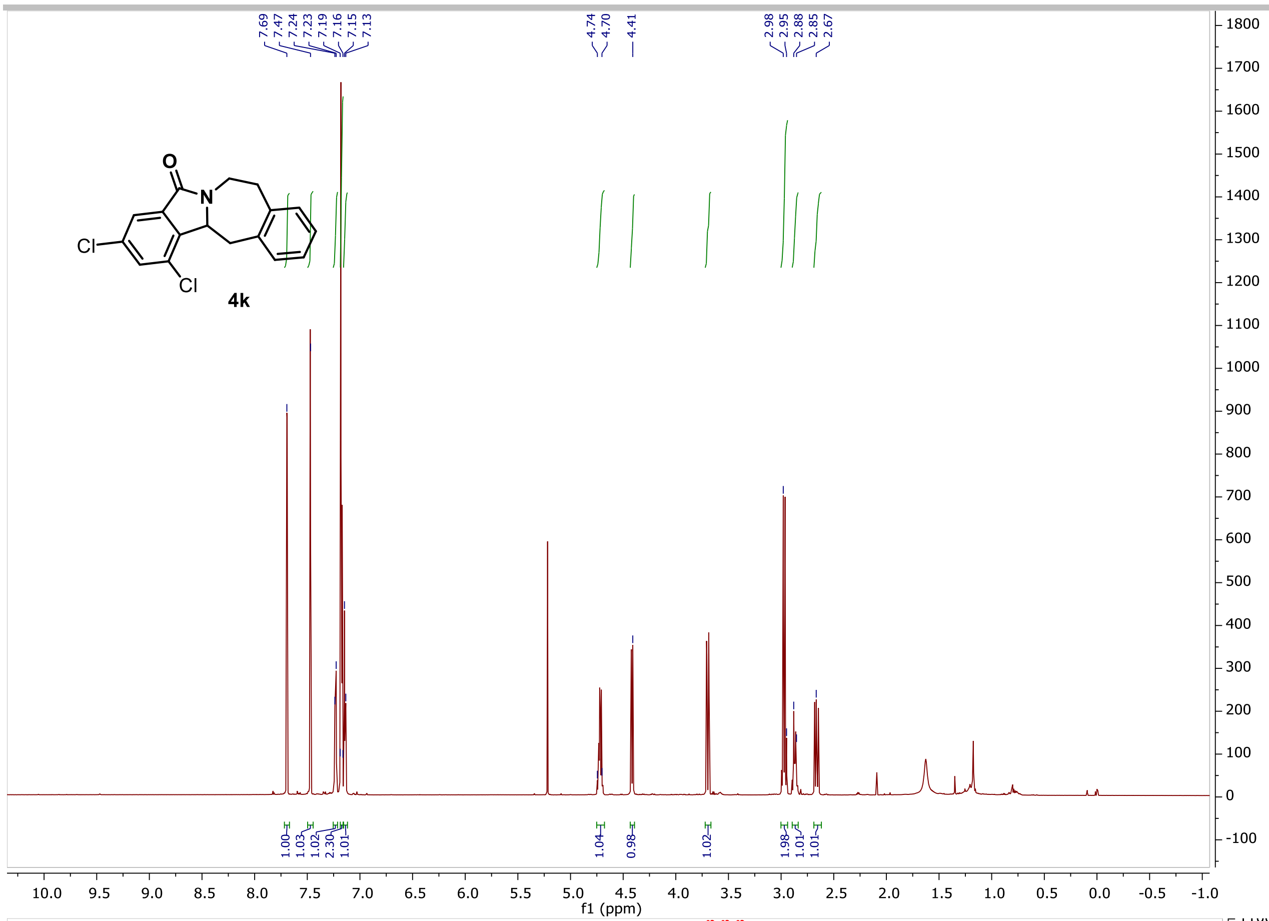
7.69
7.47
7.24
7.23
7.19
7.16
7.15
7.13
4.74
4.70
4.41
2.98
2.95
2.88
2.85
2.67
O
N
Cl
Cl
4k
1.00
1.03
1.02
2.30
1.01
1.04
0.98
1.02
1.98
1.01
1.01
f1 (ppm)

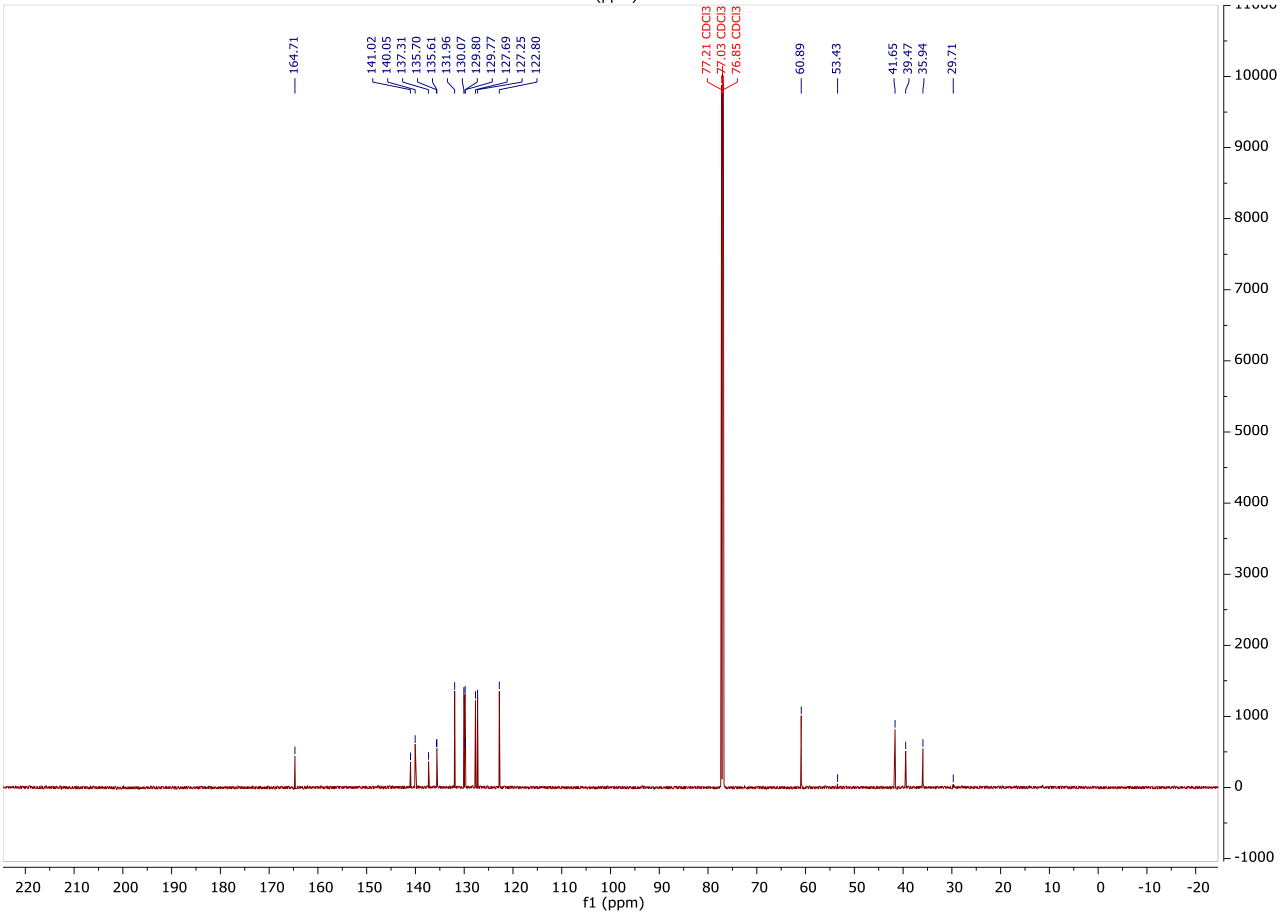
164.71
141.02
140.05
137.31
135.70
135.61
131.96
130.07
129.80
129.77
127.69
127.25
122.80
77.21 CDCl3
77.03 CDCl3
76.85 CDCl3
60.89
53.43
41.65
39.47
35.94
29.71
f1 (ppm)

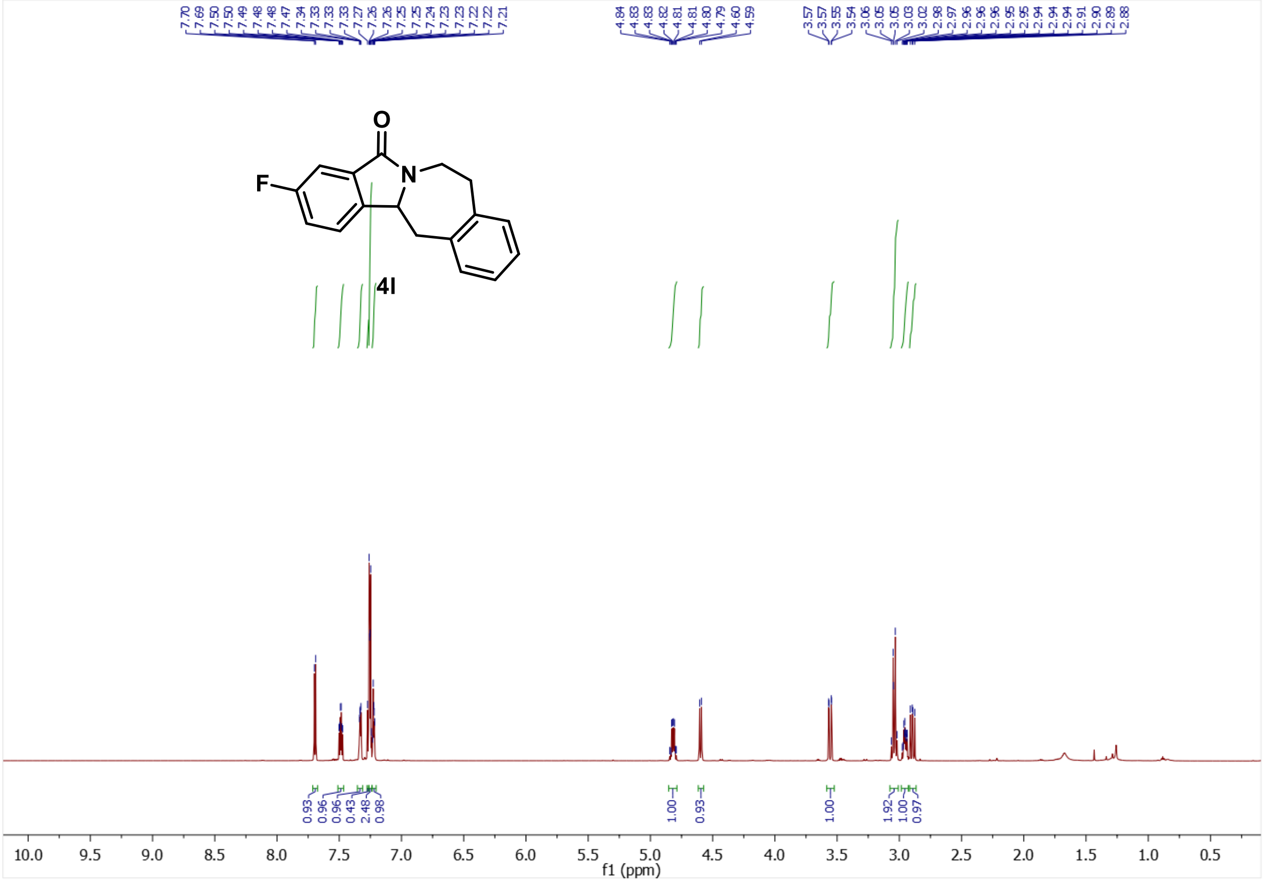
4l
f1 (ppm)

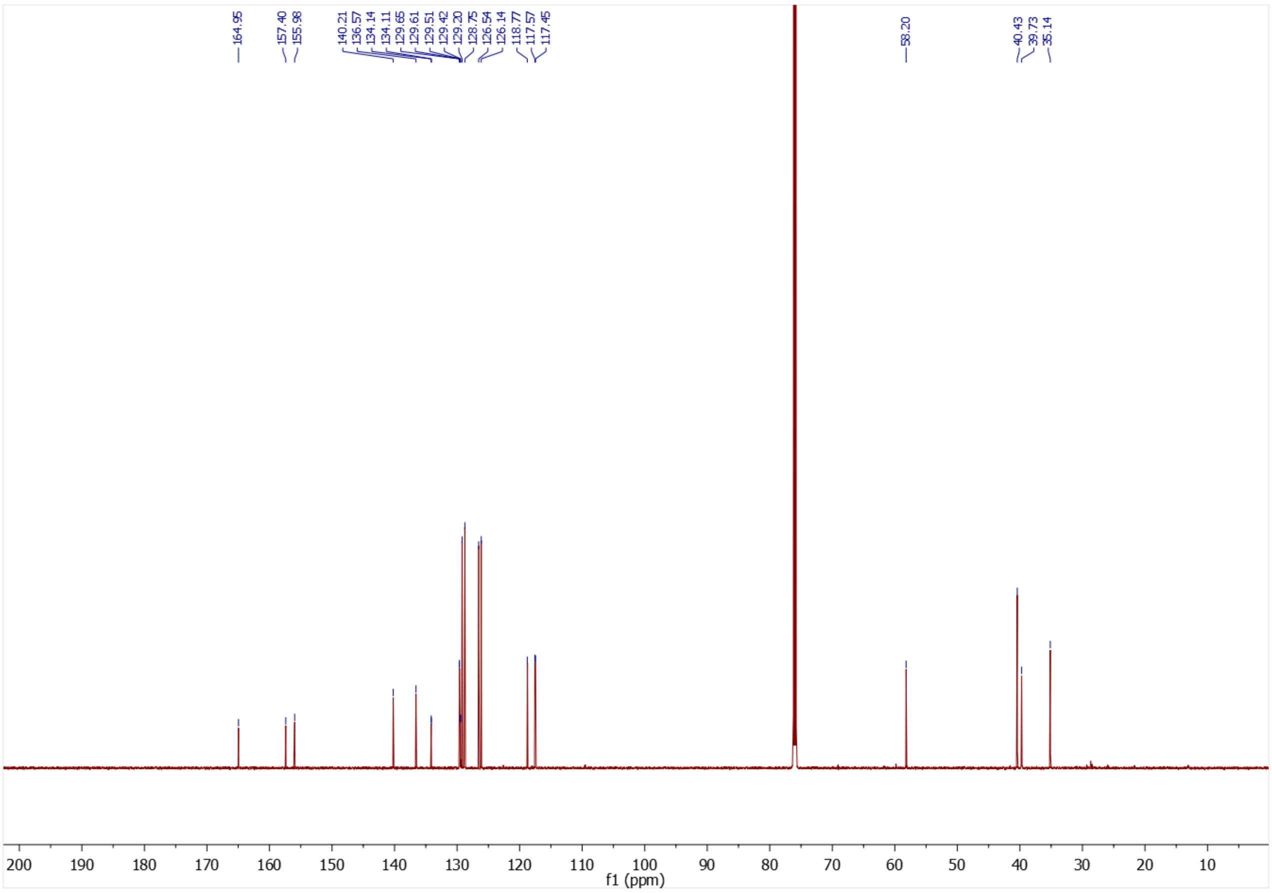
f1 (ppm)

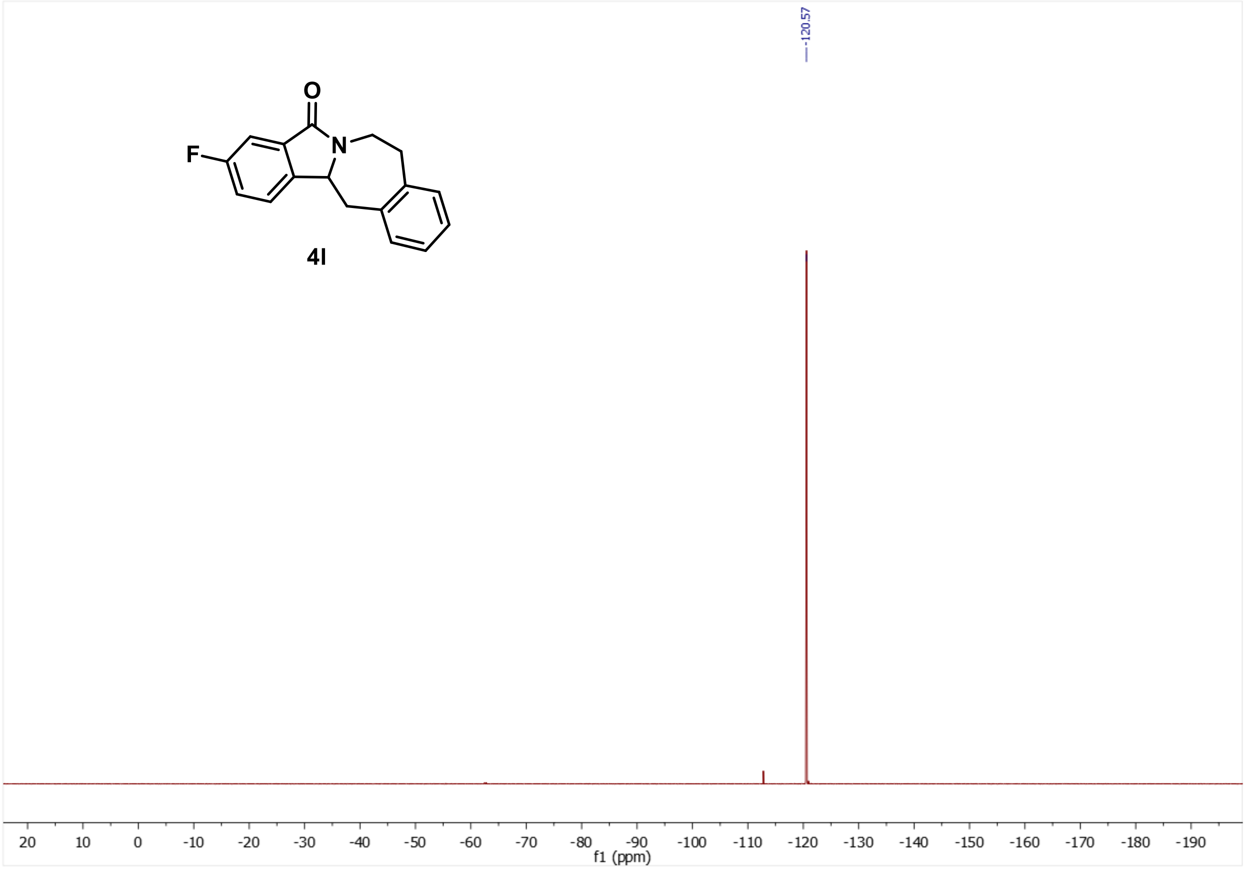
-120.57
F
O
N
4l
20
10
0
-10
-20
-30
-40
-50
-60
-70
-80
-90
-100
-110
-120
-130
-140
-150
-160
-170
-180
-190
f1 (ppm)

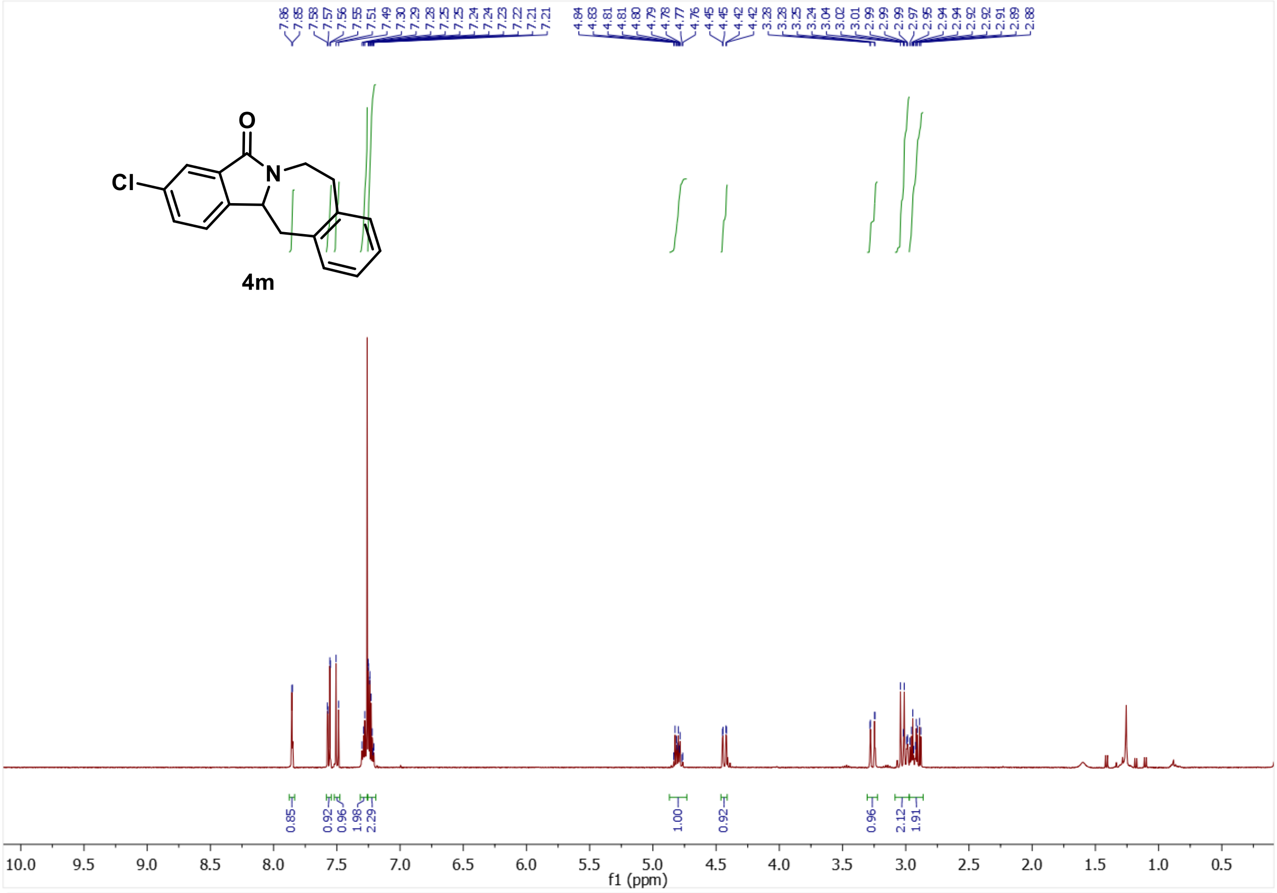
7.86
7.85
7.58
7.57
7.56
7.55
7.51
7.49
7.30
7.29
7.28
7.25
7.25
7.24
7.24
7.23
7.22
7.21
7.21
4.84
4.83
4.81
4.80
4.79
4.78
4.77
4.76
4.45
4.45
4.42
4.42
3.28
3.25
3.24
3.04
3.02
3.01
2.99
2.99
2.99
2.97
2.95
2.94
2.94
2.92
2.92
2.91
2.89
2.88
O
Cl
N
4m
0.85
0.92
0.96
1.98
2.29
1.00
0.92
0.96
2.12
1.91
10.0
9.5
9.0
8.5
8.0
7.5
7.0
6.5
6.0
5.5
5.0
4.5
4.0
3.5
3.0
2.5
2.0
1.5
1.0
0.5
f1 (ppm)

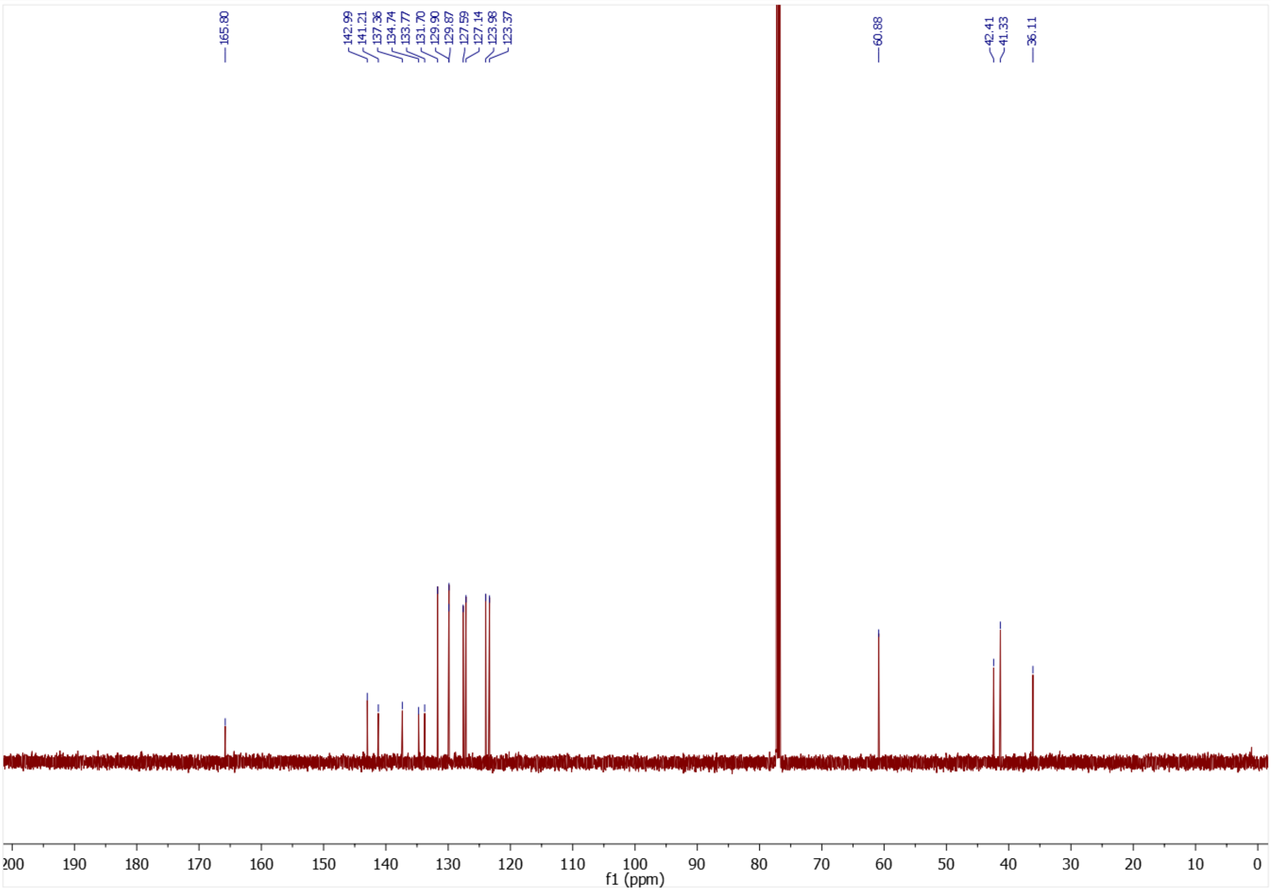
165.80
142.99
141.21
137.36
134.74
133.77
131.70
129.90
129.87
127.59
127.14
123.98
123.37
60.88
42.41
41.33
36.11
200
190
180
170
160
150
140
130
120
110
100
90
80
70
60
50
40
30
20
10
0
f1 (ppm)

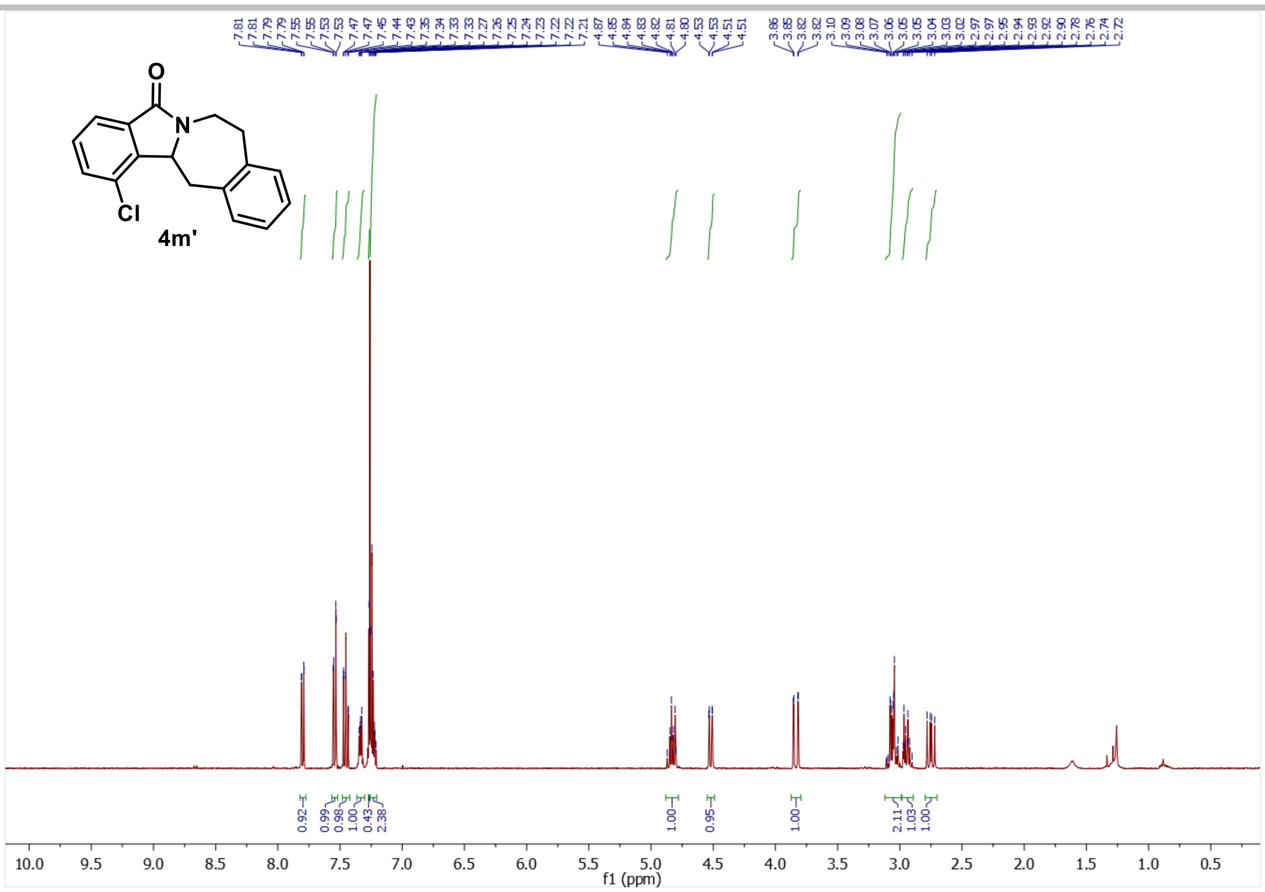
O
N
Cl
4m'
f1 (ppm)

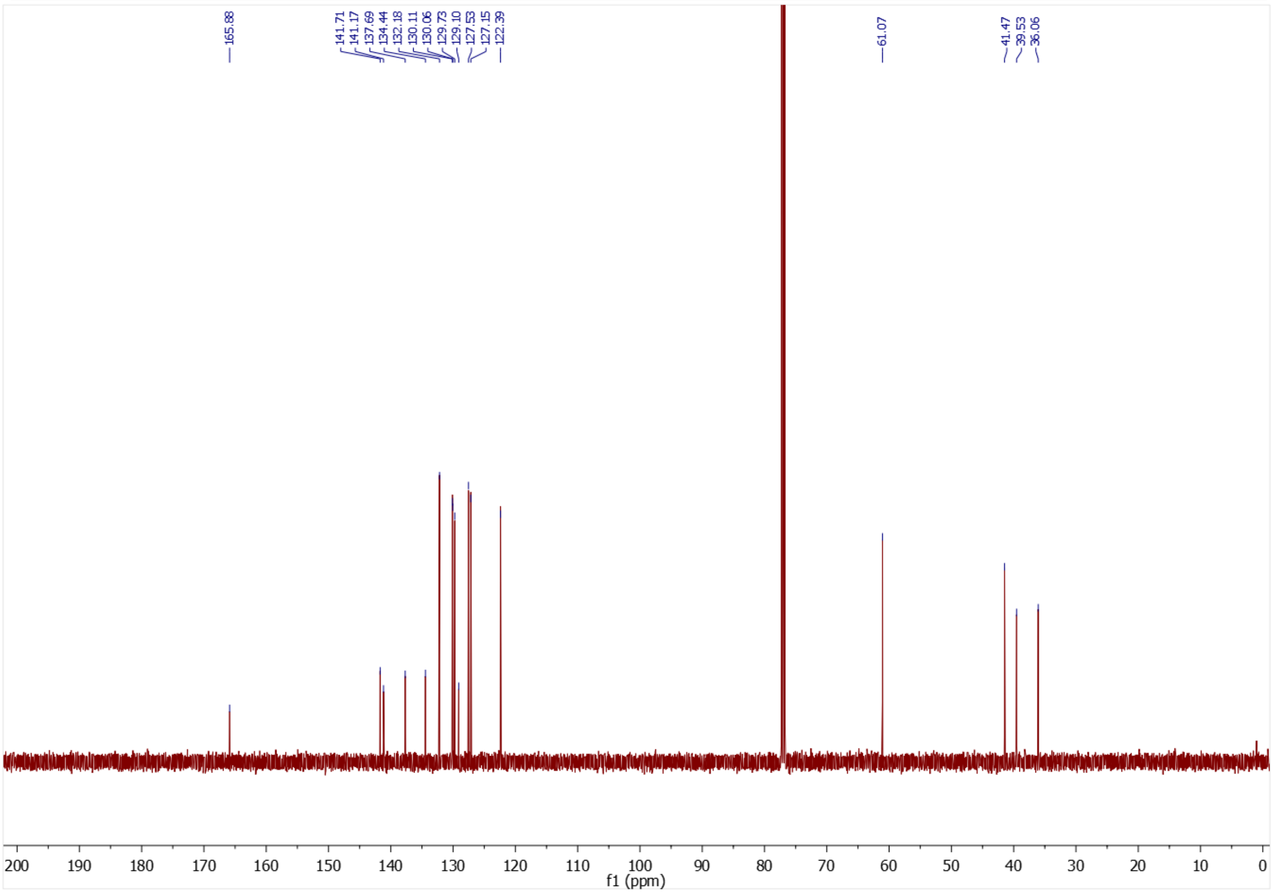
165.88
141.71
141.17
137.69
134.44
132.18
130.11
130.06
129.73
129.10
127.53
127.15
122.39
61.07
41.47
39.53
36.06
f1 (ppm)

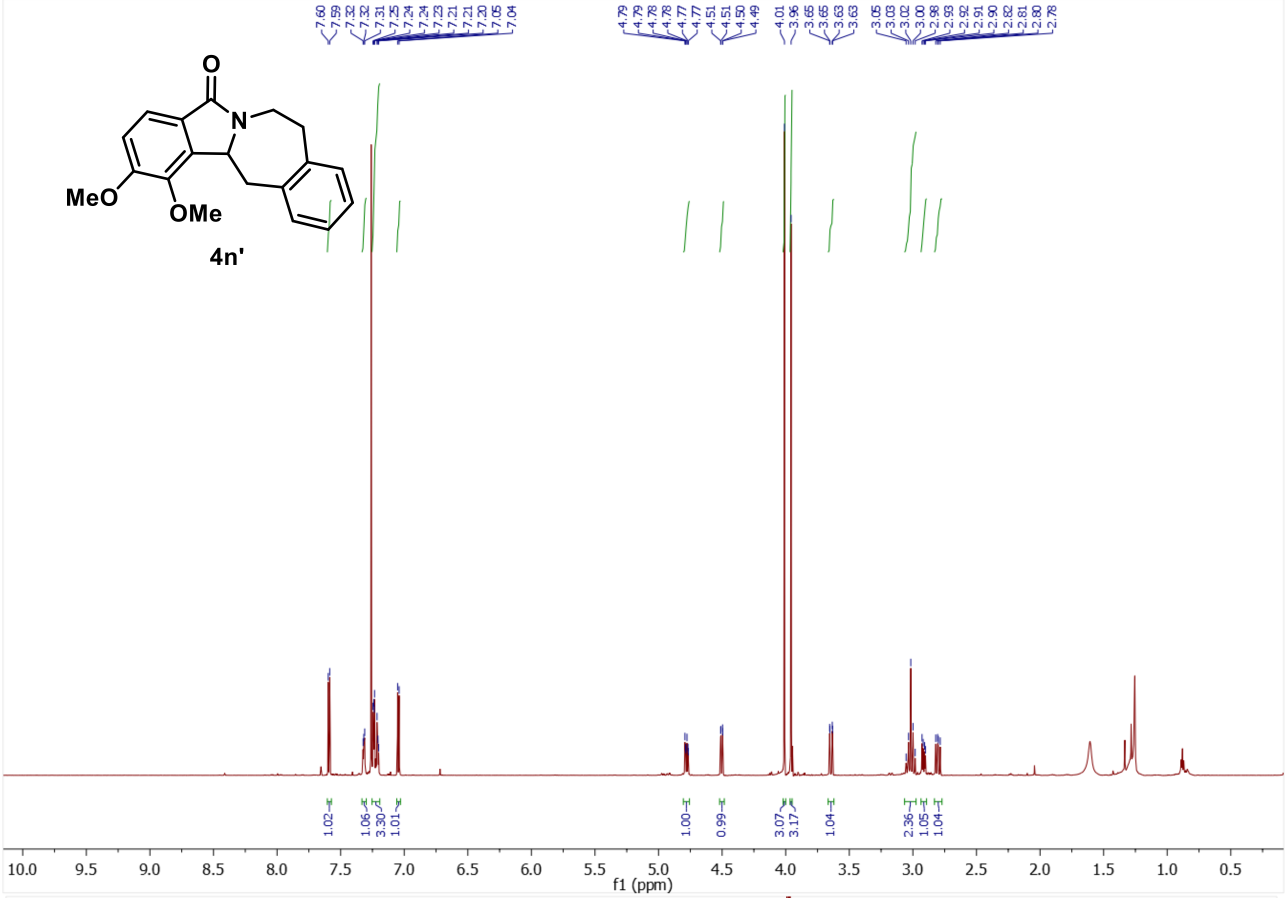
O
N
MeO
OMe
4n'
7.60
7.59
7.32
7.32
7.31
7.25
7.24
7.24
7.23
7.21
7.21
7.20
7.05
7.04
4.79
4.79
4.78
4.78
4.77
4.77
4.51
4.51
4.50
4.49
4.01
3.96
3.65
3.65
3.63
3.63
3.05
3.03
3.02
3.00
2.98
2.93
2.92
2.91
2.90
2.82
2.81
2.80
2.78
1.02
1.06
3.30
1.01
1.00
0.99
3.07
3.17
1.04
2.36
1.05
1.04
10.0 9.5 9.0 8.5 8.0 7.5 7.0 6.5 6.0 5.5 5.0 4.5 4.0 3.5 3.0 2.5 2.0 1.5 1.0 0.5
f1 (ppm)

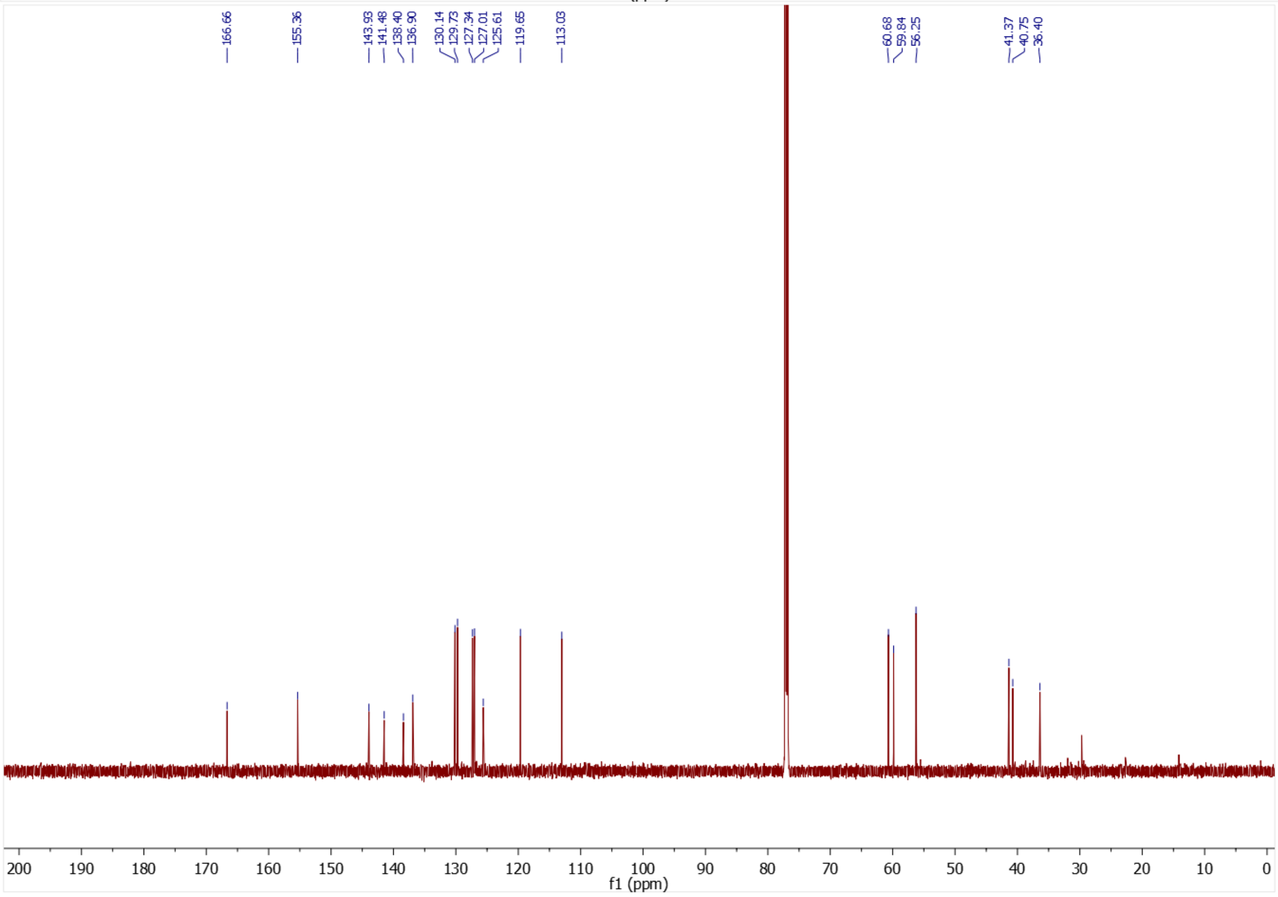
166.66
155.36
143.93
141.48
138.40
136.90
130.14
129.73
127.34
127.01
125.61
119.65
113.03
60.68
59.84
56.25
41.37
40.75
36.40
200 190 180 170 160 150 140 130 120 110 100 90 80 70 60 50 40 30 20 10 0
f1 (ppm)

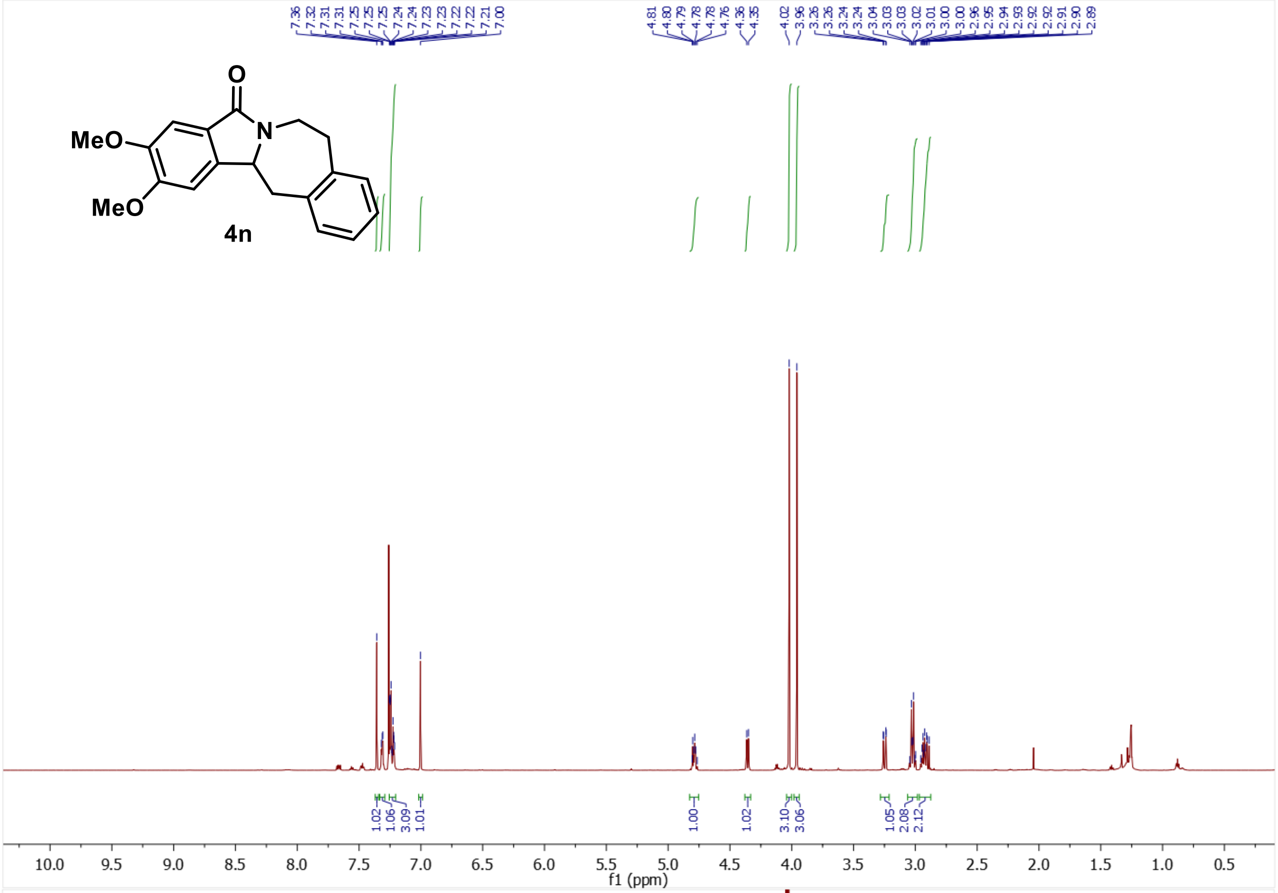
MeO
MeO
O
N
4n
7.36 7.32 7.31 7.25 7.25 7.25 7.24 7.24 7.23 7.23 7.22 7.22 7.21 7.00
4.81 4.80 4.79 4.78 4.78 4.76 4.36 4.35 4.02 3.96 3.26 3.26 3.24 3.24 3.04 3.03 3.03 3.02 3.01 3.00 3.00 2.96 2.95 2.94 2.93 2.92 2.92 2.91 2.90 2.89
1.02 1.06 3.09 1.01 1.00 1.02 3.10 3.06 1.05 2.08 2.12
10.0 9.5 9.0 8.5 8.0 7.5 7.0 6.5 6.0 5.5 5.0 4.5 4.0 3.5 3.0 2.5 2.0 1.5 1.0 0.5
f1 (ppm)

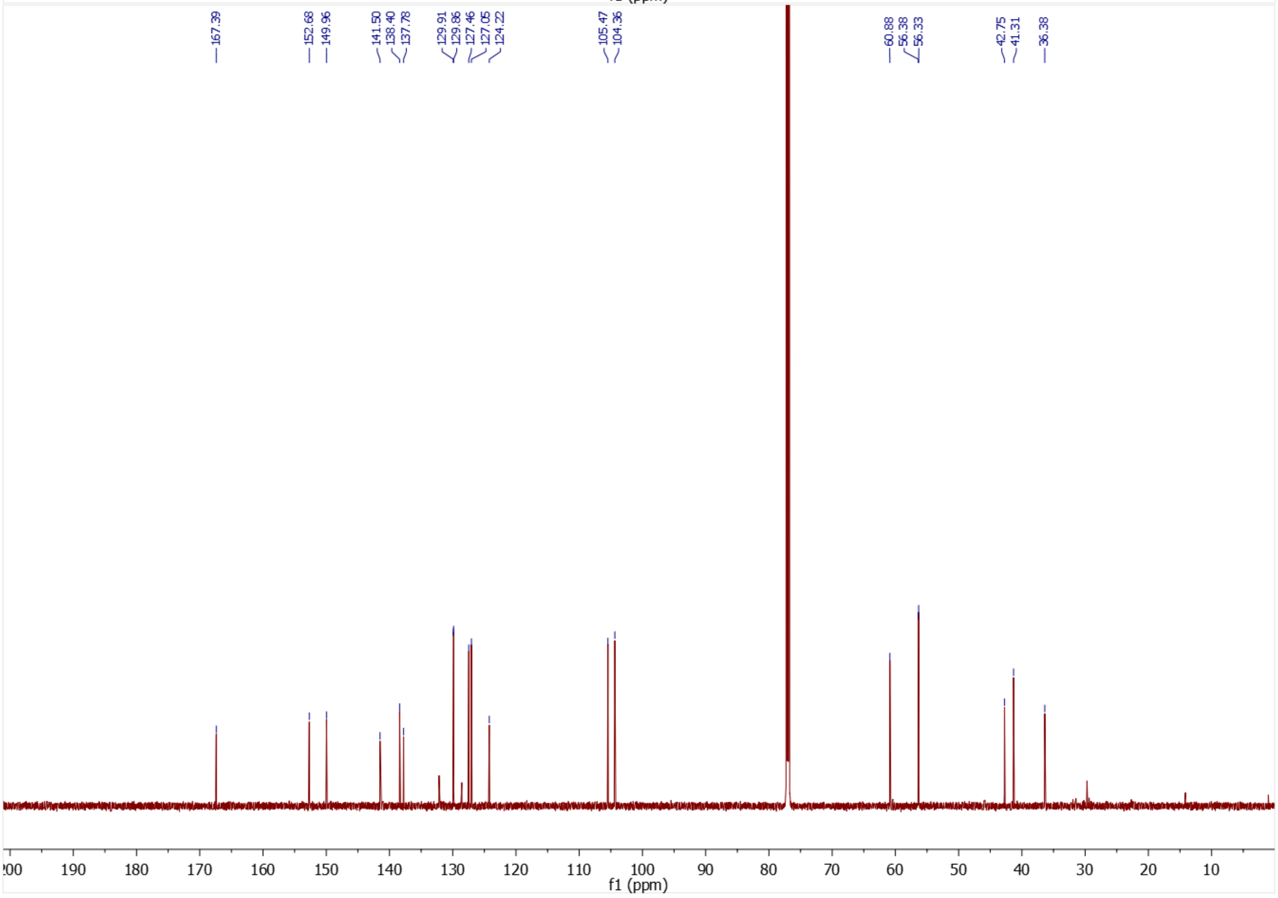
167.39
152.68 149.96
141.50 138.40 137.78
129.91 129.86 127.46 127.05 124.22
105.47 104.36
60.88 56.38 56.33
42.75 41.31
36.38
200 190 180 170 160 150 140 130 120 110 100 90 80 70 60 50 40 30 20 10
f1 (ppm)

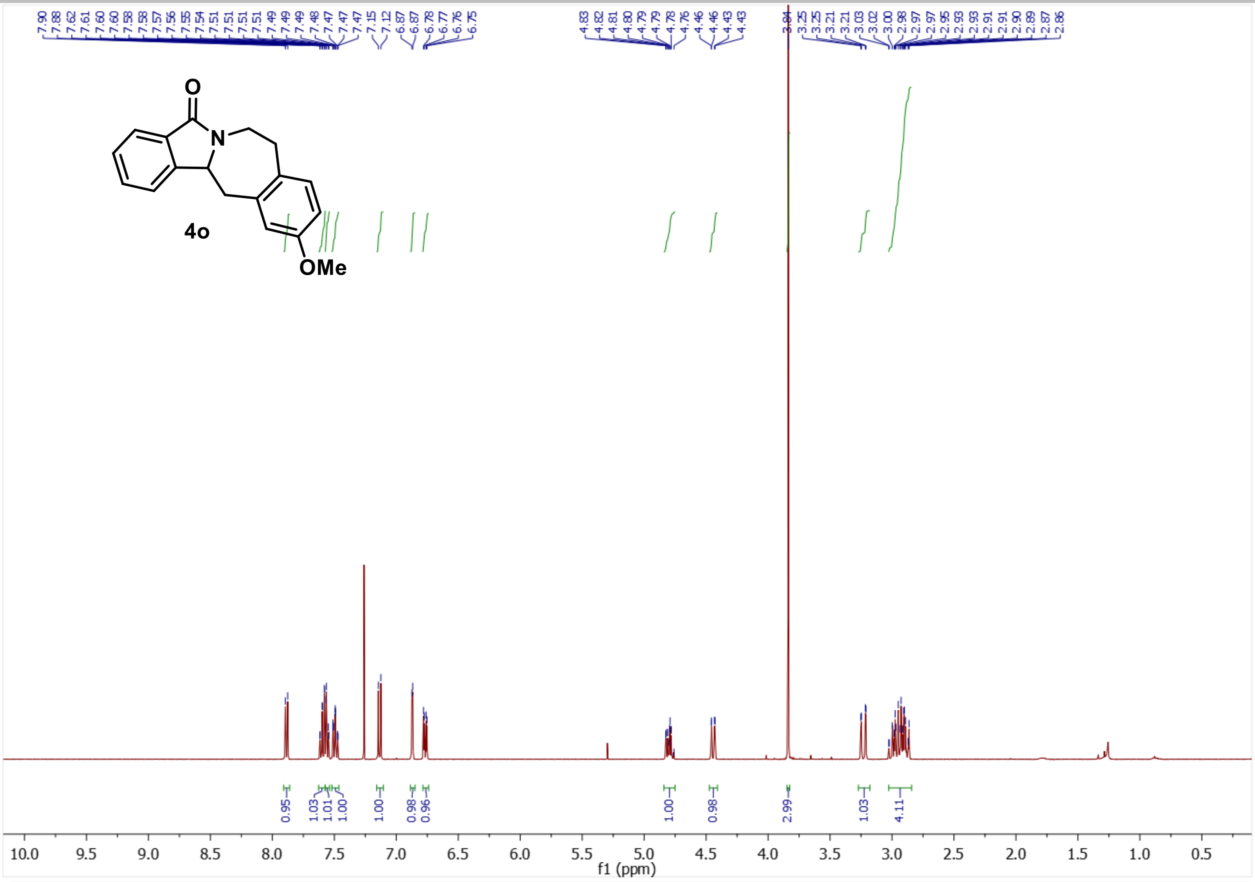
7.90 7.88 7.62 7.61 7.60 7.60 7.58 7.58 7.57 7.56 7.55 7.54 7.51 7.51 7.51 7.49 7.49 7.48 7.47 7.47 7.47 7.15 7.12 6.87 6.87 6.78 6.77 6.76 6.75
4.83 4.82 4.81 4.80 4.79 4.79 4.78 4.76 4.46 4.46 4.43 4.43
3.84 3.25 3.25 3.21 3.21 3.03 3.02 3.00 2.98 2.97 2.97 2.95 2.93 2.93 2.91 2.91 2.90 2.89 2.87 2.86
O
N
4o
OMe
0.95 1.03 1.01 1.00 1.00 0.98 0.96 1.00 0.98 2.99 1.03 4.11
f1 (ppm)

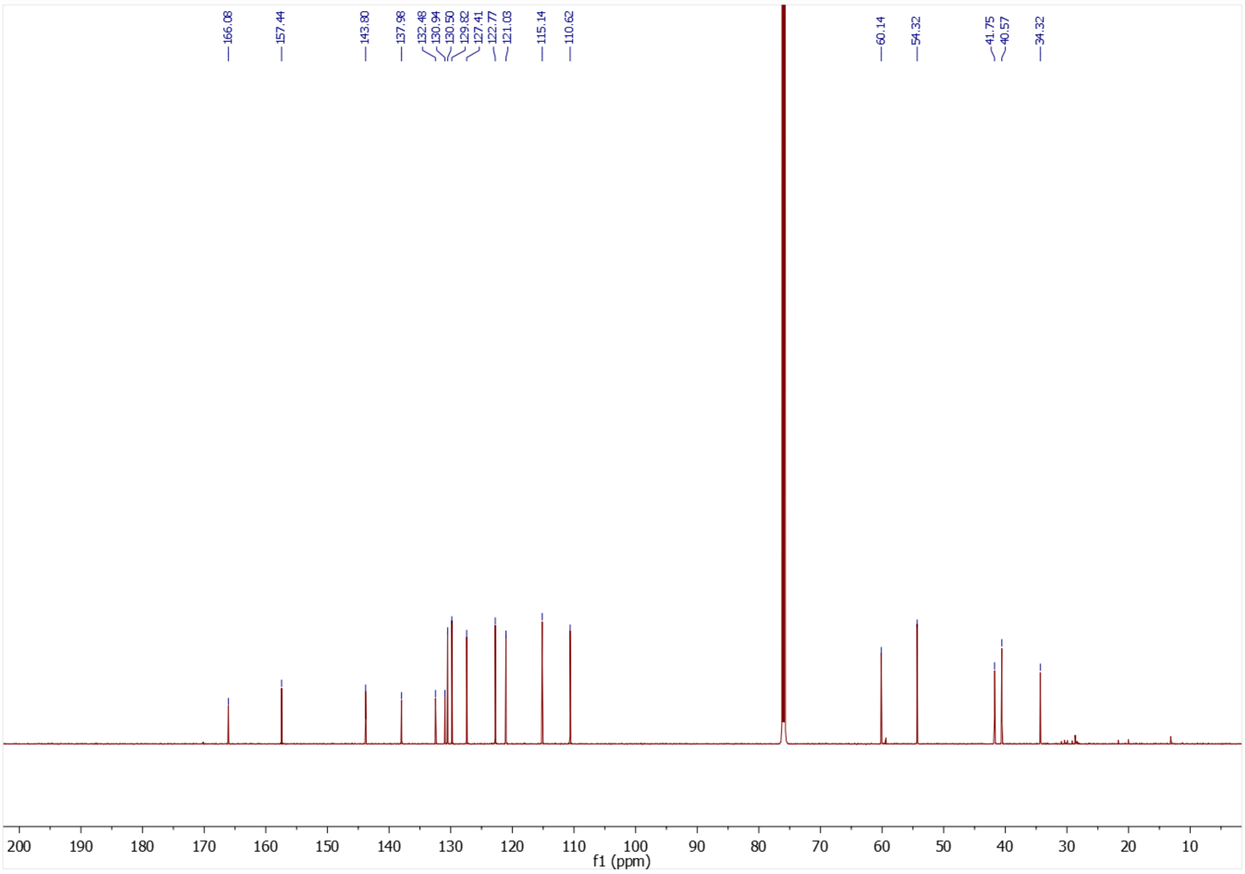
166.08 157.44 143.80 137.98 132.48 130.94 130.50 129.82 127.41 122.77 121.03 115.14 110.62 60.14 54.32 41.75 40.57 34.32
f1 (ppm)

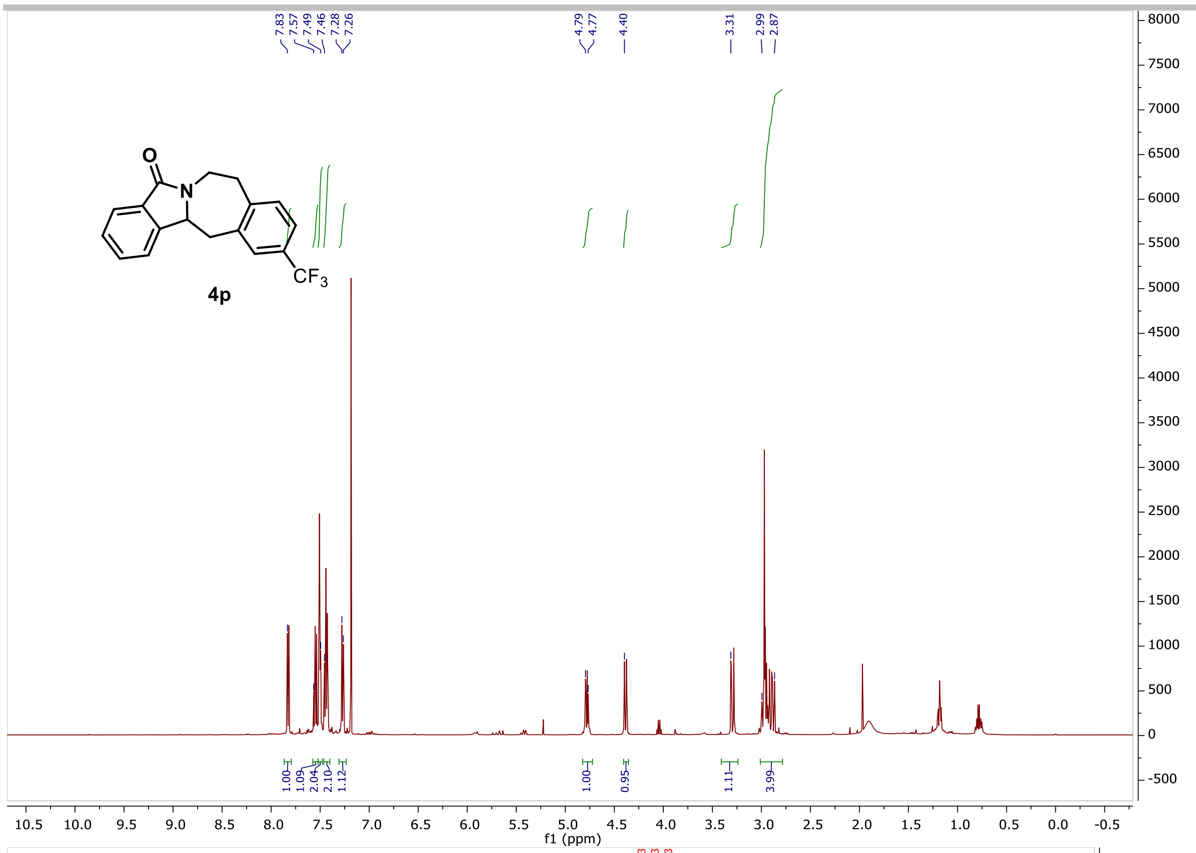
4p
CF3
O
N
7.83
7.57
7.49
7.46
7.28
7.26
4.79
4.77
4.40
3.31
2.99
2.87
1.00
1.09
2.04
2.10
1.12
1.00
0.95
1.11
3.99
f1 (ppm)

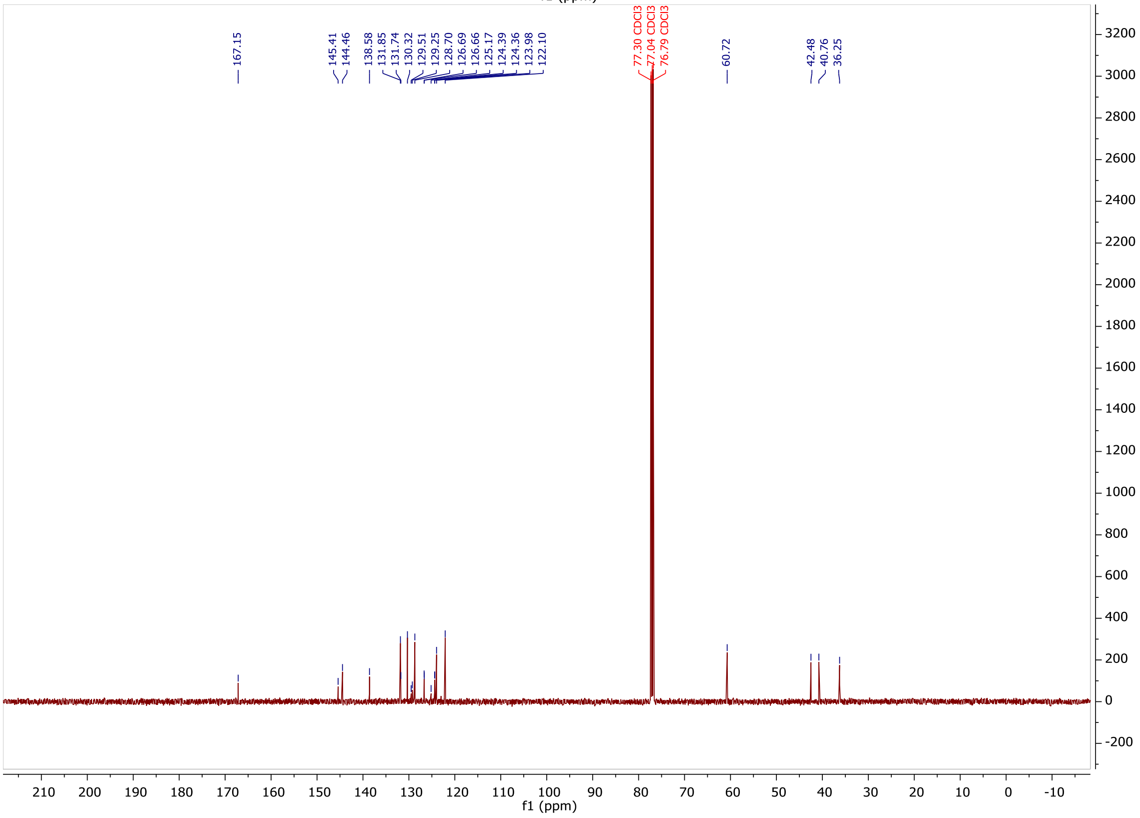
167.15
145.41
144.46
138.58
131.85
131.74
130.32
129.51
129.25
128.70
126.69
126.66
125.17
124.39
124.36
123.98
122.10
77.30 CDCl3
77.04 CDCl3
76.79 CDCl3
60.72
42.48
40.76
36.25
f1 (ppm)

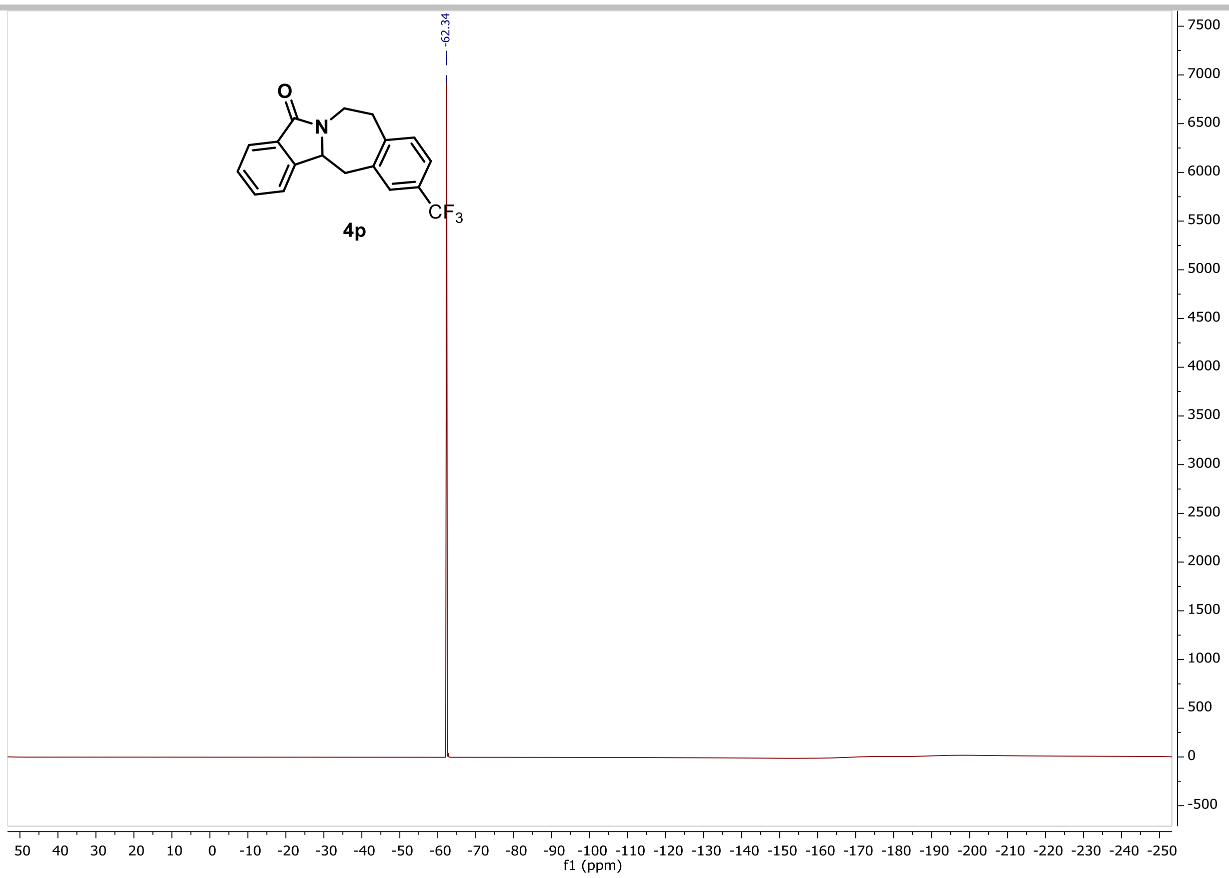
-62.34
O
N
CF3
4p
7500
7000
6500
6000
5500
5000
4500
4000
3500
3000
2500
2000
1500
1000
500
0
-500
50 40 30 20 10 0 -10 -20 -30 -40 -50 -60 -70 -80 -90 -100 -110 -120 -130 -140 -150 -160 -170 -180 -190 -200 -210 -220 -230 -240 -250
f1 (ppm)

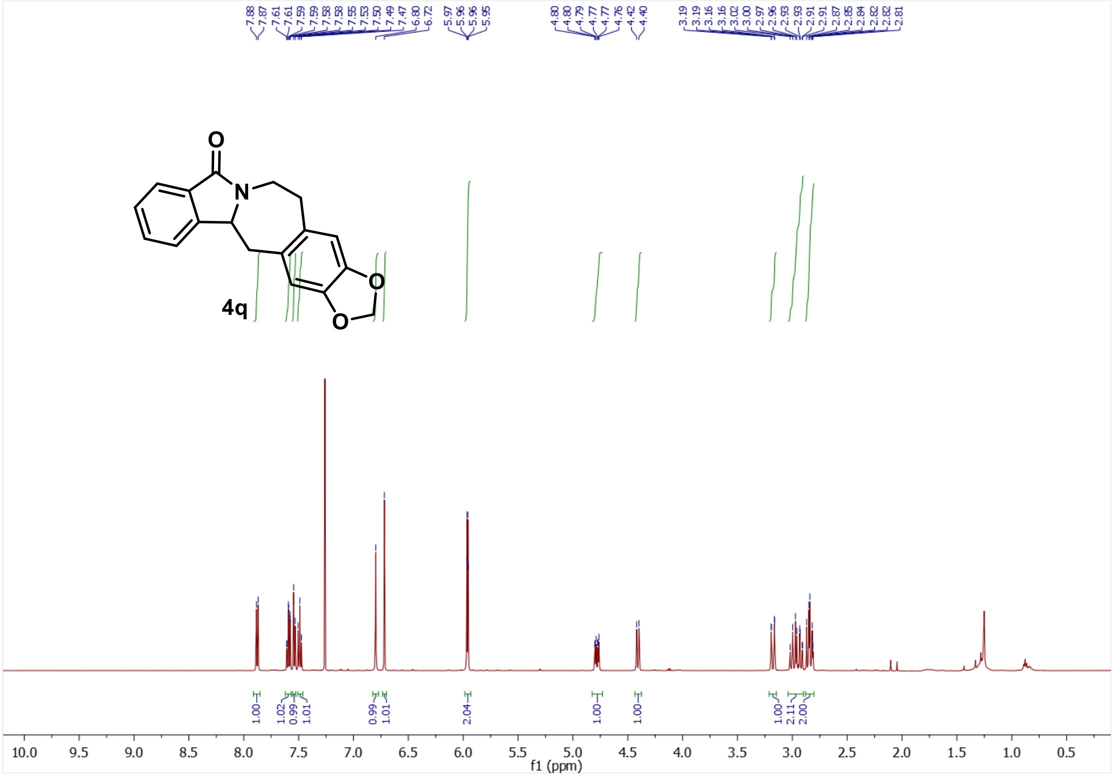
4q
7.88
7.87
7.61
7.61
7.59
7.59
7.58
7.58
7.55
7.53
7.50
7.49
7.47
6.80
6.72
5.97
5.96
5.96
5.95
4.80
4.80
4.79
4.77
4.77
4.76
4.42
4.40
3.19
3.19
3.16
3.16
3.02
3.00
2.97
2.96
2.93
2.93
2.91
2.91
2.87
2.85
2.84
2.82
2.82
2.81
1.00
1.02
0.99
1.01
0.99
1.01
2.04
1.00
1.00
1.00
2.11
2.00
f1 (ppm)

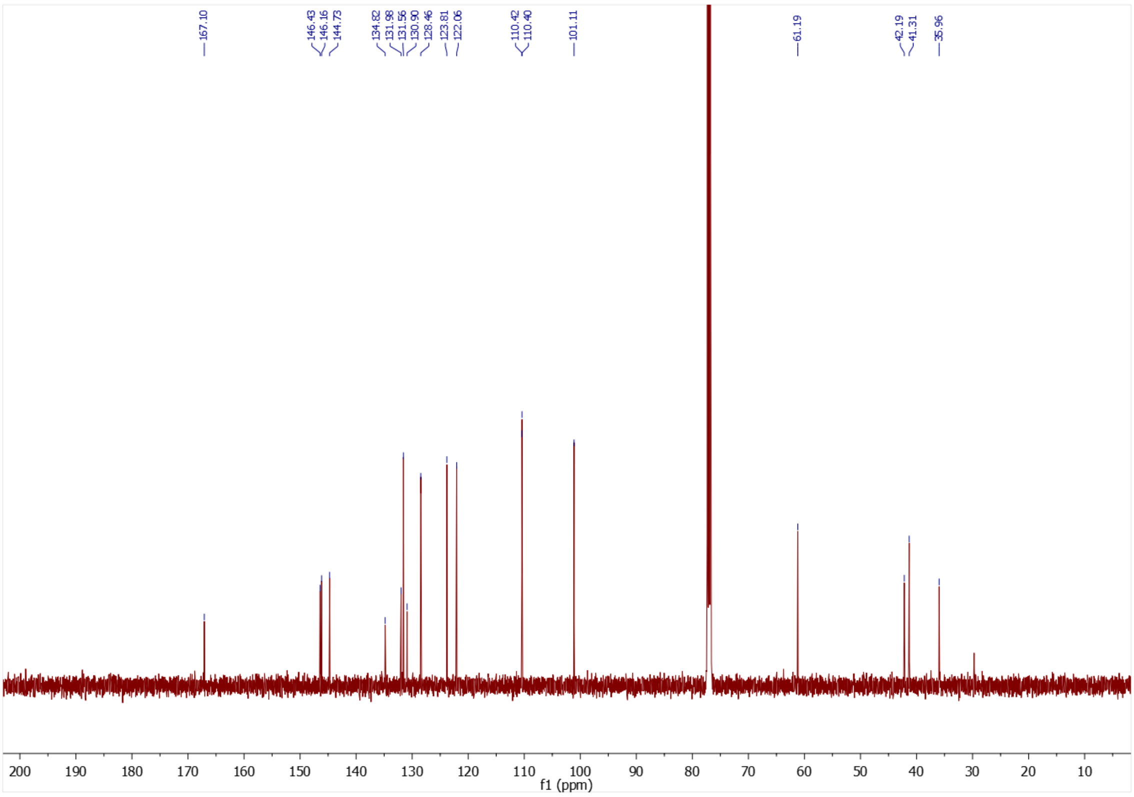
167.10
146.43
146.16
144.73
134.82
131.98
131.56
130.90
128.46
123.81
122.06
110.42
110.40
101.11
61.19
42.19
41.31
35.96
f1 (ppm)

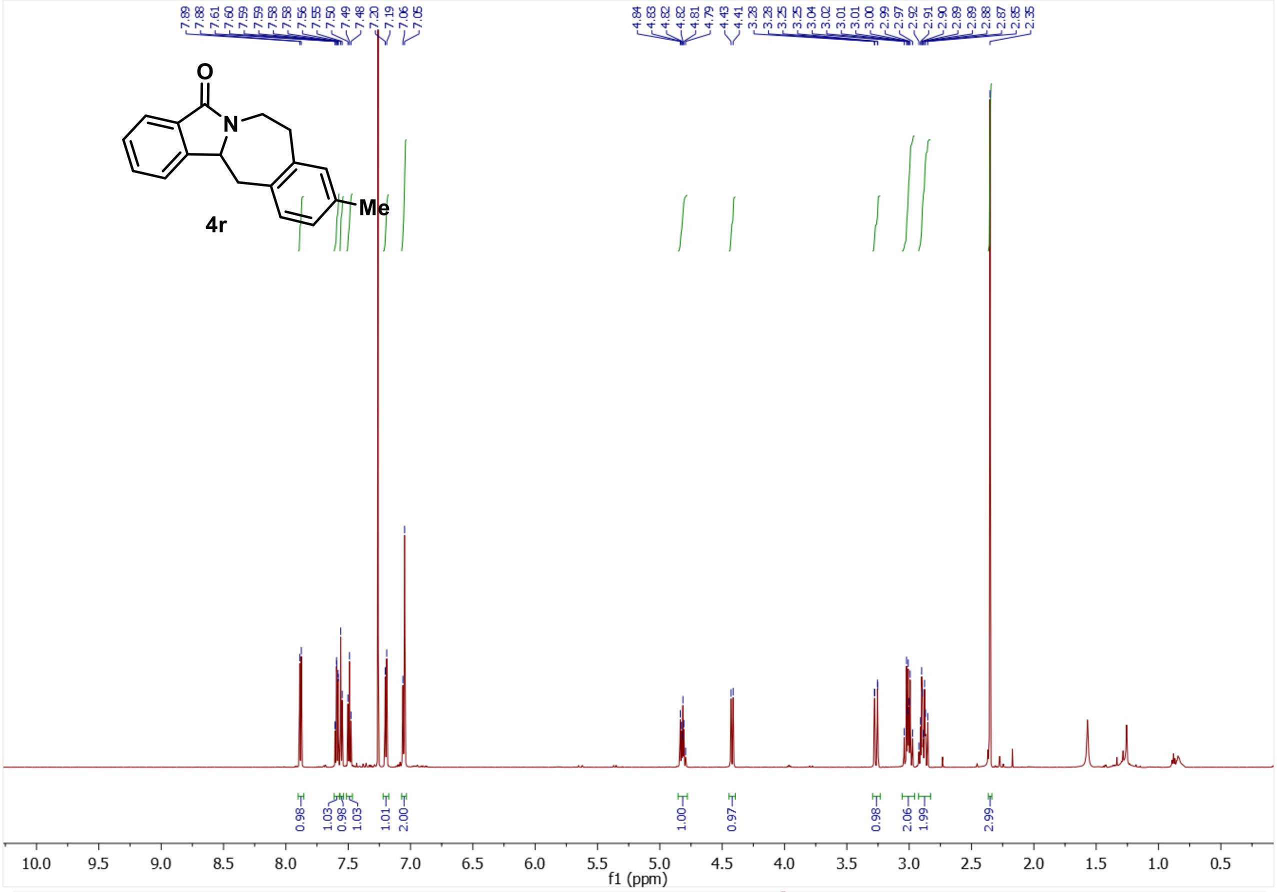
7.89
7.88
7.61
7.60
7.59
7.59
7.58
7.58
7.56
7.55
7.50
7.49
7.48
7.20
7.19
7.06
7.05
4.84
4.83
4.82
4.82
4.81
4.79
4.43
4.41
3.28
3.28
3.25
3.25
3.04
3.02
3.01
3.01
3.00
2.99
2.97
2.92
2.91
2.90
2.89
2.89
2.88
2.87
2.85
2.35
O
N
Me
4r
0.98
1.03
0.98
1.03
1.01
2.00
1.00
0.97
0.98
2.06
1.99
2.99
f1 (ppm)

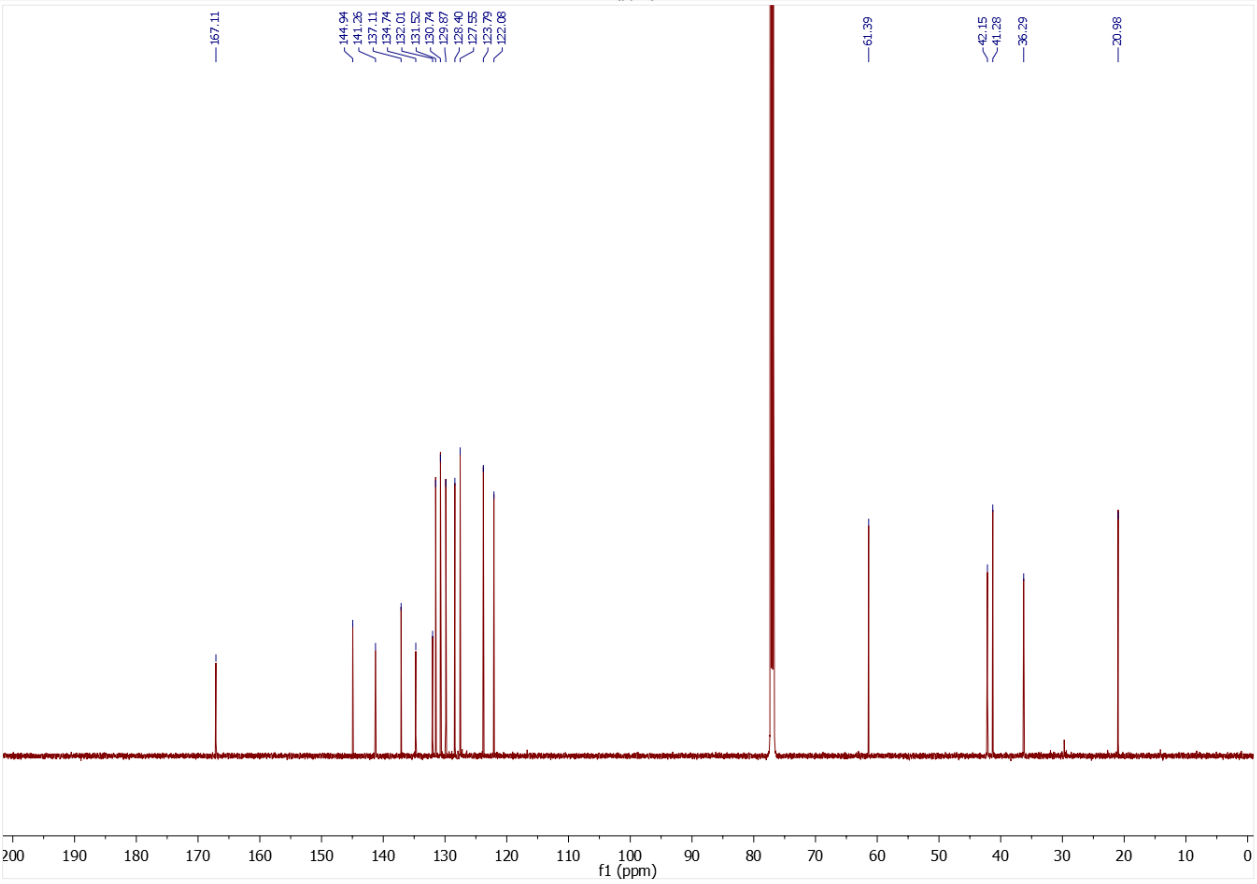
167.11
144.94
141.26
137.11
134.74
132.01
131.52
130.74
129.87
128.40
127.55
123.79
122.08
61.39
42.15
41.28
36.29
20.98
f1 (ppm)
